# Incidence of dengue and chikungunya viruses in mosquitoes and human patients in border provinces of Vietnam

**DOI:** 10.1186/s13071-017-2422-z

**Published:** 2017-11-09

**Authors:** Kim Lien Pham Thi, Laurence Briant, Laurent Gavotte, Pierrick Labbe, Marco Perriat-Sanguinet, Emmanuel Cornillot, Trong Duoc Vu, Thi Yen Nguyen, Vu Phong Tran, Van Soai Nguyen, Christian Devaux, Aneta Afelt, Chi Cuong Tran, Thi Nga Phan, Nhu Duong Tran, Roger Frutos

**Affiliations:** 1 National Institute of Hygiene and Epidemiology, 1 Yersin Street, Hanoi, 10000 Vietnam; 2 IRIM, University of Montpellier, CNRS, Montpellier, France; 3 Cirad, Intertryp, UMR 17, TA-A17/G, Campus International de Baillarguet, 34398 Cedex 5 Montpellier, France; 4 ISEM, University of Montpellier, CNRS, EPHE IRD, Montpellier, France; 5 Institut de Biologie Computationnelle (IBC), Montpellier, France; 6 IRCM, University of Montpellier, INSERM, ICM,, Montpellier, France; 7 Aix Marseille Université, CNRS, IRD, INSERM, AP-HM, URMITE, IHU-Méditerranée infection, 19-21 Boulevard Jean Moulin, 13005 Marseille, France; 8 University of Warsaw, Interdisciplinary Center for Mathematical and Computational Modelling, University of Warsaw, Prosta 69, 00-838 Warsaw, Poland; 9 IES, University of Montpellier, CNRS, Montpellier, France

**Keywords:** Dengue, Chikungunya, Vietnam, *Aedes aegypti*, *Aedes albopictus*

## Abstract

**Background:**

Dengue virus remains a major threat in Vietnam, while chikungunya virus is expected to become one. Surveillance was conducted from 2012 to 2014 in Vietnam to assess the presence of dengue and chikungunya viruses in patients hospitalized with acute fever in five Vietnam provinces neighboring Lao PDR and Cambodia. Surveillance was extended to mosquitoes present in the vicinity of the patients’ households.

**Results:**

A total 558 human serum samples were collected along with 1104 adult mosquitoes and 12,041 larvae from 2250 households. Dengue virus was found in 17 (3%) human serum samples and in 9 (0.8%) adult mosquitoes. Chikungunya virus was detected in 2 adult mosquitoes (0.18%) while no chikungunya virus was detected in humans. Differing densities of mosquito populations were found, with the highest in the Long An Province border with Cambodia. Long An Province also displayed the lowest rate of infection, despite a very high Breteau Index, high human population density and presence of the main cross border road system. The highest incidence was found in Dac Nong Province, where the Breteau and Container indices were the second lowest. Dengue virus was detected in five *Aedes albopictus*, three *Aedes aegypti* and one *Culex vishnui*. Chikungunya virus was detected in two *Ae. aegypti*. All infected mosquitoes belonged to haplotypes described in other parts of the world and a number of novel haplotypes were found among uninfected mosquitoes.

**Conclusions:**

Dengue is considered to be regularly introduced to Vietnam from Cambodia, mostly through human movement. The data reported here provides a complementary picture. Due to intensive international trade, long-distance transportation of mosquito populations may play a role in the regular importation of dengue in Vietnam through Ho Chi Minh City. It is important to decipher the movement of mosquitoes in Vietnam, not only at the Lao PDR and Cambodia borders but also through international trade routes. Mosquito surveillance programs should address and follow mosquito populations instead of mosquito species.

**Electronic supplementary material:**

The online version of this article (10.1186/s13071-017-2422-z) contains supplementary material, which is available to authorized users.

## Introduction

The current expansion of arbovirus diseases is largely due to mosquito vector range extension, driven by climate change, the globalization of transport, and to increased human movement. The absence of vaccines and efficient therapeutic drugs for the different viruses amplifies the problem. Among arboviruses, *Aedes*-borne viruses, such as dengue and chikungunya virus, are particularly targeted by national surveillance programs.

Dengue virus (DENV) infects about 390 million people per year worldwide, with 96 million asymptomatic cases [1]. Over the past 50 years dengue has spread inexorably from 9 countries prior to 1970 to over 124 today, with an increase of incidence multiplied by 30 [2]. Vietnam is one of the five countries in the Southeast Asia with the highest dengue burden [3]. First described in northern Vietnam in 1958, dengue expanded in the south during the 1960s [4]. Dengue remains a major health problem in Vietnam and the number of cases has increased over the past 15 years [5]. Vietnam is reported as a hyperendemic country [6–8], with DENV1 and DENV2 being the most predominant serotypes [9]. Dengue transmission occurs throughout the year with peaks (72% of total cases) between June and November [10] and a lower rate from December to March [10–12]. Dengue is now considered to be regularly introduced in Vietnam from neighboring countries, i.e. Cambodia and Lao PDR, and recently, dengue was shown to initiate in Ho Chi Minh City and to expand north by successive waves [13].

Chikungunya virus (CHIKV), an alphavirus first isolated in Tanzania in 1953 [14, 15], has been detected since the 1960s in Asia and was reported in Vietnam in 1967 [15]. In 2004, a new variant of CHIKV emerged in East Africa and quickly spread over the Indian Ocean, India and Thailand, causing major outbreaks, [14–19] and was described in Cambodia in 2011 and 2012 and in Lao PDR [20]. This new variant had a high affinity for *Aedes albopictus*, which is actively transported via international trade maritime routes [16]. However, until now no clinical case of chikungunya has been described in Vietnam. Dengue and chikungunya symptoms are very similar, in particular in the early stages [21], making it easy to confuse diseases and underestimate the burden of chikungunya. In addition, the same *Aedes* mosquito species are vectoring both viruses and are widely prevalent in the region. Therefore, the present work was conducted in connection with the National Vietnamese Programme for surveillance of dengue to investigate the presence of dengue and chikungunya in patients hospitalized with acute fever and in mosquito populations present around the patient’s households as well as communes located in five provinces of North, Central and South Vietnam bordering Lao PDR and Cambodia. A specific focus was put on the identification of circulating mosquito haplotypes in these regions.

## Methods

### Blood sampling

**Fig. 1:**
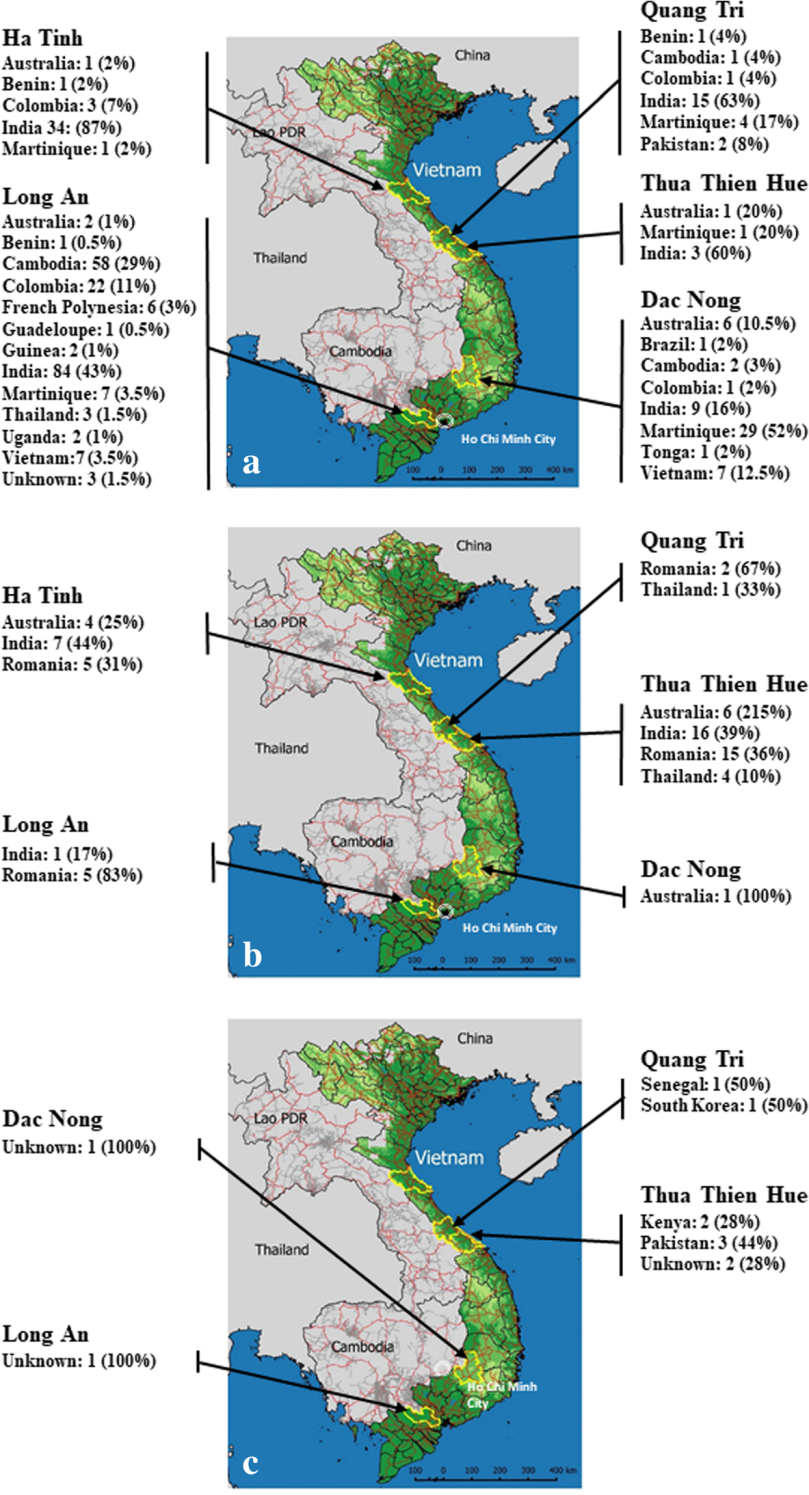
Map of sampling sites and mosquito haplotype distribution. **a** Location of *Aedes aegypti* haplotypes. **b** Location of *Aedes albopictus* haplotypes. **c** Location of haplotypes of other mosquitoes. Provinces where the sampling was conducted are surrounded in yellow. Country names indicate the countries where the haplotypes have been described. Numbers indicate the number of individuals belonging to the haplotype. Numbers in parentheses indicate the percentage

Active surveillance of acute febrile syndromes and collection of human blood samples were conducted from September 2012 to September 2014 by five preventive medicine centers in Ha Tinh, Thua Thien Hue, Quang Tri, Dac Nong and Long An (Fig. 1). A blood sample (3–5 ml) was collected from each patient during the acute phase. Serum samples were stored on dry ice and transferred the same day to final storage at -80 °C.

### Case definition

Enrolled hospitalized patients were selected when displaying a high fever (≥ 38.5 °C) and at least two other symptoms of either a rash, myalgia, joint pain, swelling of joints, nausea/vomiting or headache. Written informed consent was obtained before sample collection, either from the patient, from parents or from legal representative for minors. Sera of acute febrile cases were obtained within 7 days after the disease onset. Information collected for each patient included a unique identification number for anonymization and demographic data such as age, gender, residential address, date of symptom onset, diagnosis, and date of the first and second sample collection.

### Mosquito sampling

Sampling of larvae, pupae and adult mosquitoes was conducted four times over two years, i.e. once in the rain season (July-August) and once in the dry season (November-December) between September 2012 and September 2014. For each province, collections were conducted in four communes of a district bordering Lao PDR or Cambodia. A door-to-door entomological survey was also conducted. Adult mosquitoes were collected in and around households. A household was defined as a single residential building, including any storage building, kitchen, latrine hut and garden, as well as the outside areas up to the fenced partition separating a house from its neighbor. Households of dengue-positive inpatients were investigated when patients were at hospital, as well as other households in the commune for up to a total of 30 randomly selected households in each commune. If a selected house was impossible to reach (absence or refusal of the owner), the closest neighboring household was chosen instead. Mosquitoes were captured outdoor and indoor using a backpack aspirator between 5–10 am and 4–8 pm, during mosquito blood-feeding activity peak. Collected mosquitoes were immediately stored in RNAlater® (Qiagen, Hilden, Germany) and kept refrigerated at 4 °C prior to identification. All accessible potential larval developmental sites in and around households (discarded tires, water containers, fish water pot, flower pot, water jar/pot, water tank, vase, wet container, etc.) were searched for mosquito larvae and pupae. Potential sites were inspected using dippers, as previously described [22, 23]. All collected larvae and pupae were placed into labeled plastic vials containing RNAlater®. Species, sex (for adults only), location, date of collection and GPS coordinates of sampling points were recorded for all samples.

### DNA extraction and mosquito genotyping

DNA for mosquito genotyping was extracted from the head and thorax of adult mosquitoes using a QIAamp® DNA Micro Kit (Qiagen, Hilden, Germany). The extracted sample was eluted in 60 μl, according to the manufacturer’s protocols, and was stored at -80 °C until use. The mitochondrial gene cytochrome *c* oxidase subunit 1 (*cox*1) was used for genotyping. PCR primers are presented in Additional file 1: Table S1. PCR products were analyzed for quality in a 2% (w/v) agarose gel containing 10% SYBR safe DNA Gel Stain (Invitrogen, Carlsbad, USA) in 1% Tris-acetate-EDTA (TAE) buffer. A 100 bp ladder was used as a molecular weight marker. Sequences were obtained from a private service company (Biofidal, Lyon, France) using the dye terminator sequencing reaction method. All sequences are provided in .fasta format in Additional file 2: Table S2.

### RNA extraction, cDNA synthesis and PCR for virus identification

RNA was extracted from both human serum samples (clinical samples) and mosquitoes abdomens (field mosquito samples) using the QIAamp® viral RNA Mini kit (Qiagen). RNA templates were transcribed into cDNA using the SuperScript III reverse transcriptase (Invitrogen) and random primers. Briefly, RNA templates were mixed with RNase-free DNase solution and incubated at 37 °C for 30 min and then 75 °C for 15 min. Annealing was performed at 25 °C for 5 min using random primers, followed by extension at 42 °C for 60 min. Reactions were terminated by holding the mixture at 70 °C for 15 min. Dengue virus was detected using a one-step single tube serotype specific multiplex PCR, as previously described by Lanciotti et al. [24]. Chikungunya virus was detected using primers specific to the Nonstructural Protein 2 (*NSP2*) gene. Primers for PCR detection of dengue and chikungunya virus are presented in Additional file 1: Table S1. PCR products were analyzed for quality in a 2% (w/v) agarose gel containing 10% SYBR safe DNA Gel Stain in 1% Tris-acetate-EDTA (TAE) buffer. A 100 bp ladder was used as a molecular weight marker.

### Entomological indices

The following indices were used to describe mosquito data: (i) HI, House Index (Number of positive houses/Number of houses explored × 100); (ii) CI, Container Index (Number of positive containers/Number of containers explored × 100); and (iii) BI, Breteau Index (Number of positive containers/Number of houses explored × 100). The 95% confidence intervals were calculated using an exact binomial test.

### Sequence analysis

The *cox*1 genetic sequences were aligned using the MUSCLE algorithm [25] in the SeaView package [26]. Each sequence was compared to sequences present in the GenBank database using the online BLAST nucleotide search tool (Blastn) (https://blast.ncbi.nlm.nih.gov/Blast.cgi). The best hit for both score and coverage was retained, when existing, as the corresponding haplotype for each sample.

## Results

### Patient cohort

A total of 558 serum samples were collected from patients presenting with acute fever and symptoms compatible to DENV/CHIKV infection admitted during the 2012–2014 period in the five preventive medicine centers selected. The patient cohort consisted of 253 women and 305 men, ranging from 4 months to 74 years of age, with a median age of 24 years, and a mean age of 26 years (Table 1). The sex ratio was slightly biased (OR = 1.2) towards males, with 54.6% male patients and 45.4% female patients. The most frequent symptom recorded was myalgia (59%, Fisher’s exact test *P* = 0.0001), followed by headaches (44%, Fisher’s exact test *P* = 0.0003) and arthralgia (21%, Fisher’s exact test *P* = 0.0001) (Table 1). All symptoms were significantly more represented in the population of > 18 year-old (Fisher’s exact test *P* = 0.0001) with the exception of a rash, which was similarly represented in both populations, and nausea/vomiting, which was more frequent for patients < 18 year-old (Table 1).

**Table 1:** Frequency of symptoms upon admission

Patient data (*n* = 558)		Value^a^	Odds ratio	< 18 years (*n* = 269)	> 18 years (*n* = 289)	*P*-value^b^
Age (years)		26 (0.4–74)		(0.4–18)	(19–74)	
Sex (M/F) (%)		305/253 (54.6/45.4)	1.2			
Duration of febrile state (days)		3.6 (1–7)				
Signs and symptoms	Temperature on admission (°C)	38.8 (38–40)				
	Headache	248 (44)	0.8	36.4	51.9	0.0003
	Myalgia	328 (59)	1.4	30.8	64.8	0.0001
	Arthralgia	117 (21)	0.3	10.0	31.1	0.0001
	Rash	189 (16)	0.5	35.3	32.5	0.5311
	Petechia	156 (10)	0.4	15.6	38.8	0.0001
	Nausea/ Vomiting	68 (3.2)	0.13	13.7	1.04	0.0001
	Positive tourniquet test	53 (9.5)	0.1	4.5	14.2	0.0001
Warning sign	Back pain	56 (10)	0.11	1.85	17.7	0.0001
	Bleeding gums	18 (3.2)	0.03	0.74	5.54	0.0001
	Abdominal pain	38 (6.8)	0.07	3.34	10.03	0.001

^a^Mean (range) or number (%)

^b^Chi-square and Fisher’s exact test

### Frequency of DENV and CHIKV in the human cohort

Of the 558 acute-phase serum samples collected, 17 (3.05%) were found positive for dengue and none for chikungunya (Table 2). Dengue-positive samples were detected in all five provinces. The percentage of positive samples ranged between 2.06–5.47%, with the highest rate found in the southern Province of Dac Nong in which all four serotypes were detected. The serotype DENV1 was present in all provinces, whereas DENV2 was present only in the three most southern provinces. DENV3 was found in the northern provinces and Dac Nong while DENV4 was present only in Dac Nong. The most frequently detected serotype was DENV1 (1.25%), followed by DENV2 (0.90%); DENV3 (0.72%) and DENV4 (0.18%).

**Table 2:** Frequency of DENV and CHIKV in human sera

Province	No. of patients	Dengue serotype				Chikungunya
		D1	D2	D3	D4	
Ha Tinh	100	1	0	1	0	0
Quang Tri	97	1	0	1	0	0
Thua Thien Hue	91	1	2	0	0	0
Dac Nong	128	2	2	2	1	0
Long An	142	2	1	0	0	0
Total	558	7	5	4	1	0

### Distribution of mosquito larval populations

A total 8269 water containers from 2698 households were sampled from five provinces from September 2012 to September 2014. A total of 12,041 larvae were collected, of which 80% (9588 larvae) were identified based on morphology as *Ae. aegypti*, whereas 20% (2453 larvae) were identified as *Ae. albopictus* (Table 3). *Aedes aegypti* and *Ae. albopictus* were found in all provinces but *Ae. aegypti* was the dominant species everywhere, with a species ratio ranging from 0.53 in Thua Thien Hue to 0.99 in Long An (Table 3). Entomological indices indicated some disparities between the provinces, but no correlation was found between the number of infected houses, number of containers and mosquito density (Table 3). A high container presence in Long An (5128, when the other provinces displayed a maximum of 936 containers) with a Breteau index of 205 was linked with the lowest mosquito presence (House index of 3.02). Conversely, in Dac Nong, the Breteau index (35.71) was six times lower than in Long An and the number of larvae 10 times lower (1693 *vs* 16,480) while the House index (9.21) was three times higher.

**Table 3:** Distribution of *Aedes* larvae and entomological indices

Province	No. of houses explored	No. of positive houses (%)	No. of containers explored	No. of positive containers (%)	*Ae. aegypti* larvae	*Ae. albopictus* larvae	Species ratio	Entomological indices		
								BI^a^	CI^b^	HI^c^
Ha Tinh	450	41 (8.1)	936	79 (8.4)	1406	551	0.65	17.56	8.44	8.12
Quang Tri	420	24 (4.9)	778	170 (21.9)	1147	423	0.73	40.48	21.85	4.82
Thua Thien Hue	480	40 (6.8)	730	202 (27.7)	1147	1022	0.53	42.08	27.67	6.77
Dac Nong	420	47 (9.2)	807	150 (18.6)	760	411	0.65	35.71	18.59	9.21
Long An	480	18 (3.0)	5128	984 (19.2)	5128	46	0.99	205	19.19	3.02
Total	2250	170 (7.5)	8549	1585 (18.9)	9588	2453	0.78	7.044	18.54	1.99

^a^BI: Breteau Index = No. of positive containers / No. of houses explored × 100

^b^CI: Container Index = No. of positive containers / No. of containers explored × 100

^c^HI: House Index = No. of positive houses / No. of houses explored × 100

### Frequency of DENV and CHIKV in adult mosquitoes

A total of 1104 adult *Aedes* mosquitoes were captured from 2268 households. *Aedes aegypti* represented 89.6% (989 individuals) of collected mosquitoes and *Ae. albopictus* 10.4% (115 individuals), according to field-identification from morphological traits (Table 4). *Aedes* spp. density displayed a 10-fold increasing gradient from North (Ha Tinh) to South (Long An). Conversely, 5 *Ae. aegypti* and 80 individuals initially identified as *Ae. albopictus* adults were captured in the central Province of Hue. For *Ae. albopictus*, the highest density was observed in Thua Thien Hue (63.5% of all *Ae. albopictus* samples), whereas only a few individuals were captured in the other provinces. Of the 1104 adult *Aedes* mosquitoes collected, 9 (0.8%) were found to be infected with DENV and 2 (0.18%) were infected with CHIKV. No infected mosquitoes were found in Thua Thien Hue. Five out of nine dengue-infected mosquitoes were *Ae. albopictus* individuals and three were *Ae. aegypti* individuals. A DENV4-infected *Culex vishnui* adult, misidentified in the field as *Ae. albopictus*, was captured in the southern Province of Dac Nong (Table 4). The two CHIKV-positive *Ae. aegypti* individuals were detected in Dac Nong and Long An (Table 4). DENV-positive adult mosquitoes were detected in four of the five provinces studied (Table 4). Altogether, three species were found to be infected: *Ae. albopictus* (5 DENV-positive), *Ae. aegypti* (2 CHIKV- and 3 DENV-positive) and *C. vishnui* (1 DENV-positive) (Table 5). One individual infected with DENV2 recovered from Quang Tri was a male, indicating a vertical transmission.

**Table 4:** Presence of dengue and chikungunya viruses in sequenced adult mosquitoes

Province	Number of mosquitoes^b^			Dengue-positive			Chikunguya-positive		
	*Ae. aegypti*	*Ae. albopictus*	Other^a^	*Ae. aegypti*	*Ae. albopictus*	Other^a^	*Ae. aegypti*	*Ae. albopictus*	Other^a^
Ha Tinh	40 (55)	16 (19)	0	0	1 (DENV1)	0	0	0	0
Quang Tri	24 (64)	3 (3)	2 (2)	0	2 (DENV2)	0	0	0	0
Thua Thien Hue	5 (5)	41 (73)	7 (7)	0	0	0	0	0	0
Dac Nong	56 (285)	1 (2)	1 (1)	1 (DENV1)	0	1 (DENV4)	1	0	0
Long An	196 (580)	6 (6)	2 (2)	2 (DENV2)	1 (DENV2) 1 (DENV4)	0	1	0	0

^a^Mosquito species other than *Ae. aegypti* and *Ae. albopictus* are presented in Table 5

^b^Number of individuals of the species sequenced (total number of individuals of the species captured)

**Table 5:** Distribution of haplotypes of infected adult mosquitoes

Strain	Virus	Species	Haplotype (GenBank ID)	Country
DN_DL_01F4	DENV1	*Aedes aegypti*	Martinique 1 (JQ926696)	Martinique (French Caribbean)
DN_DL_01F8	DENV4	*Culex vishnui*	564IRI2008 (AB738195)	Unknown
DN_DMi_06F2	CHIKV	*Aedes aegypti*	Martinique 1 JQ926696)	Martinique (French Caribbean)
LA_BHT_17F6	DENV4	*Aedes albopictus*	None (HF536717)	Romania
LA_TT_22F5	DENV2	*Aedes albopictus*	None (HF536717)	Romania
LA_TT_24F1	DENV2	*Aedes aegypti*	BU-Zoo-Ae.a-31 (KT339683)	India
LA_TT_26F3	CHIKV	*Aedes aegypti*	BU-Zoo-Ae.a-31 (KT339683)	India
LA_BT_28F5	DENV2	*Aedes aegypti*	aeg7 (KP843388)	Thailand
HT_Hv_37F2	DENV1	*Aedes albopictus*	None (HF536717)	Romania
QT_LB_55M1	DENV1	*Aedes albopictus*	alb9 (KP843400)	Thailand
QT_LB_56F1	DENV1	*Aedes albopictus*	None (HF536717)	Romania

### Genetic structure of mosquito populations

Sequencing of the *cox*1 mitochondrial gene for 400 of 1104 adult mosquitoes showed that 12 individuals had been misidentified using morphological traits, corresponding to a 3% rate of misidentified mosquitoes. *Cox*1 gene analysis revealed the simultaneous presence in all sites of diverse haplotypes (Fig. 1, Additional file 3: Table S3). Attempts to assign individuals to previously identified haplotypes were difficult. *Aedes aegypti* (Fig. 1a) and *Ae. albopictus* (Fig. 1b) populations displayed high levels of diversity, with very few of the detected haplotypes being previously described in Vietnam or even in Southeast Asia. Instead, most haplotypes corresponded to those previously described in India, Australia, Africa, South America, the Caribbean islands, Pacific islands, or Europe. As an example, among the 40 *Ae. aegypti* adults captured in Ha Tinh, none belonged to a haplotype previously described in Southeast Asia. They corresponded instead to haplotypes previously identified in Australia, Benin, Colombia, India and Martinique (Fig. 1a). The Cambodia 3 haplotype of *Ae. aegypti* was found in Long An (29%), Dac Nong (3%) and Quang Tri (4%) but no individual from this haplotype was positive for dengue or chikungunya virus (Fig. 1a, Table 5, Additional file 3: Table S3). Haplotypes initially described in Vietnam were found only in Long An and Dac Nong and corresponded to 3.5 and 12.5% of the collected samples, respectively. None of the individuals belonging to the Vietnam haplotype were positive for viruses (Fig. 1a, Table 5, Additional file 3: Table S3). Several mosquito individuals corresponded to novel haplotypes and did not match any known haplotypes in GenBank. In addition, some mosquitoes misidentified as *Ae. albopictus*, based on morphological traits, were determined to be *Aedes w-albus*, *Aedes mcintoshi*, *Aedes cogilli*, *Culex vishnui* and *Culex bitaeniorhynchus* (Fig. 1c, Additional file 3: Table S3). The *cox*1 haplotypes of all infected mosquitoes have been described in other parts of the world (Table 5).

## Discussion

To our knowledge, this study is the first to investigate, in a comparative way, vector distribution, DENV and CHIKV circulation and occurrence of both inpatients hospitalized with acute febrile symptoms and mosquitoes across several provinces throughout Vietnam. The average rate of dengue among acute fever patients was about 3% and ranged between 2.06–5.70%, depending on the province. This is 10- to 5-times less than previously reported among acute fever inpatients [27, 28]. Hypotheses to explain this lower rate could be that this study focused on regional health centers, while suspected dengue cases might be directed to major hospitals not covered in this study. One cannot exclude that, since the previous studies in 2010 and 2013 used as reference were conducted [27, 28], the primary diagnostic for dengue has improved, leading to less undiagnosed dengue cases within acute fever inpatients. The presence of all four serotypes of dengue is in agreement with previous reports of the hyperendemicity status of Vietnam [8, 29].

Dengue is considered to be regularly introduced to Vietnam from Cambodia, mostly through human movement [13]. Furthermore, Raghwani et al. [13] demonstrated that infections initially started in Ho Chi Minh City and were moving north in successive waves. The higher incidence was observed in Ho Chi Minh City and urban areas, whereas a lower incidence was observed in rural areas. The low incidence reported in this work, which was conducted in rural areas, is in agreement with this previous report. This work brings a different and complementary insight by addressing, in addition to the rate of infection in acute fever inpatients, the rate of mosquito infection at, and in the vicinity of, the living places of infected patients. Under the hypothesis of a recurrent introduction of dengue from Cambodia, the Province of Long An, neighbor to Cambodia on one side and to Ho Chi Minh City on the other, was expected to display a higher rate of incidence. In fact, the incidence in human patients is one of the lowest here, despite a very high Breteau index, a proximity to high human population density and presence of the main cross border road system (Fig. 1). The province with the highest incidence was the southern province of Dac Nong. However, this province also displayed the lowest mosquito population density. The Breteau and Container indices here were the second lowest, whereas the House index was the highest, in agreement with the observed incidence rate. This indicates that, although widely used, the density of *Aedes* mosquitoes is not a reliable indicator of the risk of dengue. Although *Ae. aegypti* outnumbered *Ae. albopictus*, the latter appeared to represent a higher proportion of infected individuals. This is particularly true for the southern Province of Long An, where *Ae. albopictus* made up 50% of the infected mosquitoes while they only represented 1.2% of the mosquitoes sampled. In the northern/central provinces of Ha Tinh and Quang Tri, where *Ae. albopictus* made up 27–28% of the mosquitoes sampled, this species was the only one found to harbor dengue viruses. Interestingly, in Thua Thien Hue, where *Ae. albopictus* represented 47% of the captured mosquitoes, no infected individuals were found. These data emphasize the need to take these entomological indices with caution, as they can be misleading and do not reflect the risk of virus transmission. Furthermore, these indices are referring to the presence of species, whereas the ability of mosquitoes to efficiently transfer a virus is a trait linked to populations, i.e. subspecies [30–33]. Some specific populations, perhaps less numerous but more actively involved in the effective transmission of the virus, may have a higher impact. This would not be reflected by the entomological indices.

Another outcome of this work is the demonstration of the presence of multiple populations, i.e. maternally inherited mitochondrial *cox*1 lineages. All virus-infected mosquitoes described in this work, as well as most of the mosquitoes analyzed in this work, belonged to mitochondrial lineages, i.e. *cox*1 haplotypes, which have been described outside Southeast Asia. This feature was found in all provinces. Furthermore, a number of unknown *Ae. aegypti cox*1 haplotypes were sequenced, along with other species mistakenly field-identified as *Ae. albopictus* based on morphological traits. This simultaneous presence of several separate lineages in Vietnam indicates that there exists a high diversity of potential vectors. In addition, the description of the same haplotypes (populations) in other parts of the world indicates that a worldwide circulation of *Aedes* mosquito populations (or lineages) is taking place, and that these mosquito populations are transported worldwide including in Vietnam. Worldwide transportation and introduction of *Aedes* vectors is a wide spread phenomenon [30, 34, 35]. It is not possible to state whether these mosquito populations originated in Vietnam and were moved to other places where they were identified or, conversely, were moved to Vietnam from other parts of the world. Nevertheless, this worldwide description of the same haplotypes indicates a long distance transportation of these mosquitoes, and perhaps also of the viruses they carry, most likely due to international maritime trade [34].

This conclusion must be linked to elements previously reported. First, dengue virus was shown to undergo a complex dynamic involving both serotype and lineage replacements [8, 27, 28, 36]. Although many parameters could be involved in this mechanism, lineage replacement was thought to be more stochastic than selective [8, 36]. The worldwide circulation of mosquito populations, and the potential introduction of new virus lineages, could partly explain the regular virus replacement [8, 27, 28, 36] and the stochastic determinism of these replacements [8, 36]. Secondly, dengue virus has been shown in Vietnam to display a higher incidence in urban areas and, in particular, in Ho Chi Minh City which was described as the starting point of the north-bound expansion of dengue in Vietnam [13]. Ho Chi Minh City is located close to Cambodia, and the regular introduction of dengue virus from Cambodia to Ho Chi Minh City and then to the rest of Vietnam is very plausible. However, Ho Chi Minh City is also the economic capital of Vietnam and a main hub for the international transportation of goods. The network of ports in Ho Chi Minh City, known as Saigon Port, is the first one in size in Vietnam and 24th worldwide for containers. Introduction of mosquitoes through maritime international trade routes, in addition to introduction from Cambodia, is therefore also very plausible. Furthermore, no individual of the Cambodia 3 haplotype, described in Cambodia where the dengue virus is thought to have come from, was found to be infected with DENV or CHIKV. This work demonstrates that there is, therefore, no single local endemic population of mosquitoes involved in the transmission of dengue and chikungunya in Vietnam, but rather a countrywide co-circulation of several distinct populations of both *Ae. aegypti* and *Ae. albopictus* belonging to lineages distributed worldwide. Dengue-infected *Ae. aegypti* mosquitoes belonged to lineages described in Martinique, India and Thailand. With the exception of QT_LB_albo_55M1 which belonged to the haplotype alb9 of *A. albopictus* described in Thailand, all the other dengue-infected *Ae. albopictus* individuals belonged to the same population, which has no haplotype name and which was characterized in Romania and described as an invasive population in Europe [37]. The alb9 dengue-infected mosquito was a male, indicating the presence of vertical transmission, a trait already known for dengue [38–40].

One dengue-infected mosquito was found not to be *Ae. albopictus* but instead *Culex vishnui*. This is, to our knowledge, the first time *C. vishnui*, a species known for transmitting Japanese encephalitis, has been found infected with dengue. There is no evidence from the data reported here that *C. vishnui* can efficiently transmit dengue to humans through a blood meal. However, there is at least the demonstration that *C. vishnui* can take up the dengue virus and therefore be considered as a potential vector. Further research should be considered to investigate the occurrence of efficient transmission of dengue in this already medically-important mosquito group. It is also an indication that vector population studies must be extended to all mosquitoes species present, not *Ae. aegypti* and *Ae. albopictus*, when surveying the actual virus circulation in an ecosystem.

## Conclusions

This work stresses the need to establish a genotype-based survey of circulating mosquitoes in Vietnam, not based on the species level as is currently done, but rather on the population at the infra-species level, with an extension to other local mosquito species. The entomological indices currently in use are misleading and other sets of indices reflecting the involvement of specific populations in the transmission must be developed. An integrative analysis encompassing the genetic study of viral lineages in human patients and in mosquitoes, along with the genotyping of mosquito populations, should be undertaken to provide clear information on the dynamics of dengue and chikungunya. Although no clinical chikungunya case was declared and no CHIKV was found in human febrile patients in this study, the detection of CHIKV in mosquito haplotypes bound to worldwide movements, to areas with major chikungunya outbreaks, indicate that the threat should be taken seriously and a dedicated surveillance program should be implemented. Dengue and chikungunya, and perhaps other *Aedes*-borne diseases, appear as global threats that should not be addressed at a national or even regional scale but rather at a global scale, with worldwide dimension characterized by permanent exchanges and movements.

## Additional files

Additional file 1: Table S1. Oligonucleotide primers used for polymerase chain reaction (DOCX 13 kb)

Additional file 2: Table S2. Mosquito *cox*1 sequences (DOCX 29 kb)

Additional file 3: Table S3. Distribution of haplotypes of captured adult mosquitoes (DOCX 31 kb)

## Abbreviations

BI: Breteau index
cDNA: complementary deoxyribonucleic acid
CHIKV: chikungunya virus
CI: container index
*cox*1: cytochrome *c* oxidase subunit 1
DENV: Dengue virus
DF: Dengue fever
DHF: dengue hemorrhagic fever
HI: house index
Lao PDR: Lao People Democratic Republic
NIHE: National Institute of Hygiene and Epidemiology
RT-PCR: reverse transcription-polymerase chain reaction
WHO: World Health Organization
