# Supplementary material for: Incidence of dengue and chikungunya viruses in mosquitoes and human patients in border provinces of Vietnam
Source: Parasit Vectors. 2017 Nov 9;10:556. doi: 10.1186/s13071-017-2422-z (PMC5680899; doi:10.1186/s13071-017-2422-z)
Supplement: Supplementary file 1 — Oligonucleotide primers used for polymerase chain reaction (DOCX 13 kb) [file 13071_2017_2422_MOESM1_ESM.docx]

**Additional file 1: Table S1:** Oligonucleotide primers used for polymerase chain reaction

|  | **Primer** | **Sequence (5'-3')** | **Position^1^** | **Size (bp)** | **Hybridization temperature** | |
| --- | --- | --- | --- | --- | --- | --- |
| DENV | D1 | TCAATATGCTGAAACGCGCGAGAA ACCG | 616-644 | 511 | | 55°C |
|  | TS1 | CGTCTCAGTGATCCG GGGG | 568-586 | 482 (D1 and TS 1) | |  |
|  | TS2 | CGC CAC AAG GGC CAT GAA CAG | 232-252 | 119 (D1 and TS 2) | |  |
|  | TS3 | TAA CAT CAT CAT GAG ACA GAGC | 400-421 | 290 (D1 and TS 3) | |  |
|  | TS4 | TGTTGTCTTAAACAAGAGAGGTC | 506-527 | 392 (D1 and TS 4) | |  |
| CHIKV | NSP2-F | GGCACTGGTCCCAGATAATTCAAG | 3181-3241 | 120 | | 55°C |
|  | NSP2-R | GCTGTCTAGTACCACCCCATACATG |  |  | |  |
| *cox*1 gene | COI F | TATCGCCTAAACTTCAGCC |  | 1651 | | 56°C |
|  | COI R | CCTAAATTTGCTCATGTTGCC |  |  | |  |
