# Supplementary material for: Incidence of dengue and chikungunya viruses in mosquitoes and human patients in border provinces of Vietnam
Source: Parasit Vectors. 2017 Nov 9;10:556. doi: 10.1186/s13071-017-2422-z (PMC5680899; doi:10.1186/s13071-017-2422-z)
Supplement: Supplementary file 2 — Mosquito cox1 sequences (DOCX 29 kb) [file 13071_2017_2422_MOESM2_ESM.docx]

**Additional file 2: Table S2.** Mosquito *cox*1 sequences

>DN_DL_01F1

GGTGGGGTCAGATCAGATAAGTGTTGGTATAAAATAGGGTCTCCCCCTCCGATTGGGTCAAAGAAAGATGTATTTAAGTTTCGGTCTGTTAATAATATAGTAATAGCTCCAGCTAAAACAGGAAGAGAAAGAAGTAATAAGATAGCTGTAATAACTACAGATCAAACAAATAAGGGTAGTTGATCTAAAGTAATTCCTGACGATCGTATATTAATTACAGTTGTAATAAAATTTACTGCCCCTAAAATTGAGGAAATTCCAGCTAAATGAAGAGAAAAAATAGCTTAATCAACAGAAGCTCCAGCATGAGCTGTTCCTGAAGAGAGAGGAGGATAAACTGTTCACCCAGTTCCTGCCCCATTTTCTACTATTGAGCTTGATAATAGAAGAGTCAATGAAGGAGGTAGTATTCAAAAACTTATATTATTCATTCGAGGGAAAGCTATATCAGGGGCTCCTAATATTAAAGGAACTAATCAA

>DN_DL_01F2

TTGGGGTCAGATCAGATAAGTGTTGGTATAAAATAGGATCTCCTCCTCCGATTGGATCAAAGAAAGATGTATTTAAGTTTCGGTCTGTTAATAACATAGTAATAGCTCCAGCTAAAACAGGAAGAGAAAGAAGTAATAAGATAGCTGTAATAACTACAGATCAAACAAATAAAGGTAGTCGATCTAAAGTAATTCCTGACGATCGTATATTAATTACAGTTGTAATAAAATTTACTGCCCCTAAAATTGAGGAAATTCCAGCTAAATGAAGAGAAAAAATAGCTAAATCAACAGAAGCTCCAGCATGAGCTGTTCCTGAAGAGAGAGGAGGATAAACTGTTCACCCAGTTCCTGCTCCATTTTCTACTATTGAGCTTGATAATAGAAGAGTCAATGAAGGAGGTAGTATTCAAAAACTTATATTATTTATTCGAGGAAAGGCTATATCAGGGGCTCCTAATAT

>DN_DL_01F3

GTGGGGTTCAGATCAAATAAGTGTTGGTATAAAATAGGGTCTCCCCCTCCGATTGGATCAAAGAAAGATGTATTTAAGTTTCGGTCTGTTAATAATATAGTAATAGCTCCAGCTAAAACAGGAAGAGAAAGAAGTAATAAGATAGCTGTAATAACTACAGATCAAACAAATAAGGGTAGTCGATCTAAAGTAATCCCTGACGATCGCATATTAATCACAGTTGTAATAAAATTTACTGCCCCTAAAATTGAGGAAATTCCAGCTAAATGAAGAGAAAAAATAGCTAAATCAACAGAAGCTCCAGCATGAGCTGTTCCTGAAGAGAGAGGAGGATAAACTGTTCACCCAGTCCCTGCCCCATTTTCTACTATTGAGCTTGATAATAGAAGAGTCAATGAAGGAGGTAGTATTCAAAAACTTATATTATTCATTCGAGGGAAAGCTATATCAGGGGCTCCTAATATTAAAGGAACTAATCACCTTCCAAATCCTCCCACA

>DN_DL_01F4

GGGCGTCAGATCAGATAAGTGTTGGTATAAAATAGGGTCTCCCCCTCCGATTGGATCAAAGAAAGATGTATTTAAGTTTCGGTCTGTTAATAATATAGTAATAGCTCCAGCTAAAACAGGAAGAGAAAGAAGTAATAAGATAGCTGTAATAACTACAGATCAAACAAATAAGGGTAGTCGATCTAAAGTAATCCCTGACGATCGCATATTAATCACAGTTGTAATAAAATTTACTGCCCCTAAAATTGAGGAAATTCCAGCTAAATGAAGAGAAAAAATAGCTAAATCAACAGAAGCTCCAGCATGAGCTGTTCCTGAAGAGAGAGGAGGATAAACTGTTCACCCAGTCCCTGCCCCATTTTCTACTATTGAGCTTGATAATAGAAGAGTCAATGAAGGAGGTAGTATTCAAAAACTTATATTATTCATTCGAGGGAAAGCTATATCAGGGGCTCCTAATATTAAAGGAACTAATCAATTCCAAAATTTCCTCCATA

>DN_DL_01F5

GGGGGGGTCAGATCAGATAGTGTTGGTATAAAATAGGATCTCCTCCTCCGATTGGATCAAAGAAAGATGTATTTAAGTTTCGGTCTGTTAATAACATAGTAATAGCTCCAGCTAAAACAGGAAGAGAAAGAAGTAATAAGATAGCTGTAATAACTACAGATCAAACAAATAAAGGTAGTCGATCTAAAGTAATTCCTGACGATCGTATATTAATTACAGTTGTAATAAAATTTACTGCCCCTAAAATTGAGGAAATTCCAGCTAAATGAAGAGAAAAAATAGCTAAATCAACAGAAGCTCCAGCATGAGCTGTTCCTGAAGAGAGAGGAGGATAAACTGTTCACCCAGTTCCTGCCCCATTTTCTACTATTGAGCTTGATAATAGAAGAGTCAATGAAGGAGGTAGTATTCAAAAACTTATATTATTTATTCGAGGAAAGGCTATATCAGGGGCTCCTAATATTAAAGGAACTAATCAATTCCAAAATTCCCTCCCGGA

>DN_DL_01F6

GGTGGGTCAAGATCAAATAAGTGTTGGTATAAAATAGGGTCTCCCCCTCCGATTGGATCAAAGAAAGATGTATTTAAGTTTCGGTCTGTTAATAATATAGTAATAGCTCCAGCTAAAACAGGAAGAGAAAGAAGTAATAAGATAGCTGTAATAACTACAGATCAAACAAATAAGGGTAGTCGATCTAAAGTAATCCCTGACGATCGCATATTAATCACAGTTGTAATAAAATTTACTGCCCCTAAAATTGAGGAAATTCCAGCTAAATGAAGAGAAAAAATAGCTAAATCAACAGAAGCTCCAGCATGAGCTGTTCCTGAAGAGAGAGGAGGATAAACTGTTCACCCAGTTCCTGCCCCATTTTCTACTATTGAGCTTGATAATAGAAGAGTCAATGAAGGAGGTAGTATTCAAAAACTTATATTATTCATTCGAGGGAAAGCTATATCAGGGGCTCCTAATATTAAAGGAACTAATCAATTTCCAAATTCCTCC

>DN_DL_01F7

GGTGGGGCAAGGATCAAAATAAGTGTTGGTATAAAATAGGGTCTCCCCCTCCGATTGGGTCAAAGAAAGATGTATTTAAGTTTCGGTCTGTTAATAATATAGTAATAGCTCCAGCTAAAACAGGAAGAGAAAGAAGTAATAAGATAGCTGTAATAACTACAGATCAAACAAATAAGGGTAGTTGATCTAAAGTAATTCCTGACGATCGTATATTAATTACAGTTGTAATAAAATTTACTGCCCCTAAAATTGAGGAAATTCCAGCTAAATGAAGAGAAAAAATAGCTAAATCAACAGAAGCTCCAGCATGAGCTGTTCCTGAAGAGAGAGGAGGATAAACTGTTCACCCAGTTCCTGCCCCATTTTCTACTATTGAGCTTGATAATAGAAGAGTCAATGAAGGAGGTAGTATTCAAAAACTTATATTATTCATTCGAGGGAAAGCTATATCAGGGGCTCCTAATATTAAAGGAACTAATCAATTTCCAAAATCCT

CC

>DN_DL_01F8

GGGGAAGTCAAGATCAAATAAGTGTTGATAAAGAATTGGGTCTCCTCCTCCAATTGGGTCAAAGAATGAAGTATTTAAATTTCGATCTGTTAATAATATAGTAATAGCTCCGGCTAATACAGGTAATGAAAGAAGTAATAAAACAGCAGTAATTACTACTGATCAAACAAATAATGGTATTCGATCAAGTGTAATTCCTGAAGATCGTATATTAATAACTGTAGTAATAAAATTTACTGCCCCTAAAATTGATGAAATCCCTGCTAAATGTAAAGAAAAAATAGCTAAATCAACTGAGGCCCCAGCATGAGCTGTTCCAGATGATAAAGGAGGATAAACTGTTCATCCTGTCCCAGCTCCATTTTCTACTAAACTACTTGAAAGTAGTAAAGTTAATGAAGGTGGAAGTATTCAAAAACTTATATTATTTATTCGTGGAAAAGCTATATCAGGAGCTCCTAGTATTAAAGGAACTAATCAATTTCCAAAATCCTCC

>DN_DL_01F9

GTGGGGTCAAGATCAGATAAGTGTTGGTATAAAATAGGGTCTCCTCCTCCGATTGGATCAAAGAAAGATGTATTTAAGTTTCGGTCTGTTAATAACATAGTAATAGCTCCAGCTAAAACAGGAAGAGAAAGAAGTAATAAGATAGCTGTAATAACTACAGATCAAACAAATAAAGGTAGTCGATCTAAAGTAATTCCTGACGATCGTATATTAATTACAGTTGTAATAAAATTTACTGCCCCTAAAATTGAGGAAATTCCAGCTAAATGAAGAGAAAAAATAGCTAAATCAACAGAAGCTCCAGCATGAGCTGTTCCTGAAGAGAGAGGAGGATAAACTGTTCACCCAGTTCCTGCCCCATTTTCTACTATTGAGCTTGATAATAGAAGAGTCAATGAAGGAGGTAGTATTCAAAAACTTATATTATTTATTCGAGGAAAGGCTATATCAGGGGCTCCTAATATTAAAGGAACTAATCACCATACAAATTTCCTCCCCA

>DN_DL_01F10

GGGGGGTCAAGATCAGATAAGTGTTGGTATAAAATAGGGTCTCCCCCTCCGATTGGGTCAAAGAAAGATGTATTTAAGTTTCGGTCTGTTAATAATATAGTAATAGCTCCAGCTAAAACAGGAAGAGAAAGAAGTAATAAGATAGCTGTAATAACTACAGATCAAACAAATAAGGGTAGTTGATCTAAAGTAATTCCTGACGATCGTATATTAATTACAGTTGTAATAAAATTTACTGCCCCTAAAATTGAGGAAATTCCAGCTAAATGAAGAGAAAAAATAGCTAAATCAACAGAAGCTCCAGCATGAGCTGTTCCTGAAGAGAGAGGAGGATAAACTGTTCACCCAGTTCCTGCCCCATTTTCTACTATTGAGCTTGATAATAGAAGAGTCAATGAAGGAGGTAGTATTCAAAAACTTATATTATTCATTCGAGGGAAAGCTATATCAGGGGCTCCTAATATTAAAGGAACTAATCAATACCAAATTTCCTCACAGAG

>DN_DL_02F1

GGGGGGTCAAGGATCAGATAAGTGTTGGTATAAAATAGGGTCTCCCCCTCCGATTGGATCAAAGAAAGATGTATTTAAGTTTCGGTCTGTTAATAATATAGTAATAGCTCCAGCTAAAACAGGAAGAGAAAGAAGTAATAAGATAGCTGTAATAACTACAGATCAAACAAATAAGGGTAGTCGATCTAAAGTAATCCCTGACGATCGCATATTAATCACAGTTGTAATAAAATTTACTGCCCCTAAAATTGAGGAAATTCCAGCTAAATGAAGAGAAAAAATAGCTAAATCAACAGAAGCTCCAGCATGAGCTGTTCCTGAAGAGAGAGGAGGATAAACTGTTCACCCAGTCCCTGCCCCATTTTCTACTATTGAGCTTGATAATAGAAGAGTCAATGAAGGAGGTAGTATTCAAAAACTTATATTATTCATTCGAGGGAAAGCTATATCAGGGGCTCCTAATATTAAAGGAACTAATCAATTAACAAAATTCCCTCCAAGA

>DN_DL_02F2

GTGGGTCAGATCAAATAAGTGTTGGTATAAAATAGGGTCTCCCCCTCCGATTGGATCAAAGAAAGATGTATTTAAGTTTCGGTCTGTTAATAATATAGTAATAGCTCCAGCTAAAACAGGAAGAGAAAGAAGTAATAAGATAGCTGTAATAACTACAGATCAAACAAATAAGGGTAGTCGATCTAAAGTAATCCCTGACGATCGCATATTAATCACAGTTGTAATAAAATTTACTGCCCCTAAAATTGAGGAAATTCCAGCTAAATGAAGAGAAAAAATAGCTAAATCAACAGAAGCTCCAGCATGAGCTGTTCCTGAAGAGAGAGGAGGATAAACTGTTCACCCAGTCCCTGCCCCATTTTCTACTATTGAGCTTGATAATAGAAGAGTCAATGAAGGAGGTAGTATTCAAAAACTTATATTATTCATTCGAGGGAAAGCTATATCAGGGGCTCCTAATATTAAAGGAACTAATCAACTACAAAATTCCCTCCTACAGT

>DN_DL_02F3

GGGCCGTCCAAGTATCAAAATAAGTGTTCCGTACAAAATAGGGTCTCCCCCTCCGATTGGGTCAAAGAAAGATGTATTTAAGTTTCGGTCTGTTAATAATATAGTAATAGCTCCAGCTAAAACAGGAAGAGAAAGAAGTAATAAGATAGCTGTAATAACTACAGATCAAACAAATAAGGGTAGTTGATCTAAAGTAATTCCTGACGATCGTATATTAATTACAGTTGTAATAAAATTTACTGCCCCTAAAATTGAGGAAATTCCAGCTAAATGAAGAGAAAAAATAGCTAAATCAACAGAAGCTCCAGCATGAGCTGTTCCTGAAGAGAGAGGAGGATAAACTGTTCACCCAGTTCCTGCCCCATTTTCTACTATTGAGCTTGATAATAGAAGAGTCAATGAAGGAGGTAGTATTCAAAAACTTATATTATTCATTCGAGGGAAAGCTATATCAGGGGCTCCTAATATTAAAGGAACTAATCAATTTCCAAATTCCTCCCA

>DN_DL_02F4

GGGGGGGGTCAAGATCAAAATAAGTGTTGGTATAAAATAGGGTCTCCCCCTCCGATTGGATCAAAGAAAGATGTATTTAAGTTTCGGTCTGTTAATAATATAGTAATAGCTCCAGCTAAAACAGGAAGAGAAAGAAGTAATAAGATAGCTGTAATAACTACAGATCAAACAAATAAGGGTAGTCGATCTAAAGTAATCCCTGACGATCGCATATTAATCACAGTTGTAATAAAATTTACTGCCCCTAAAATTGAGGAAATTCCAGCTAAATGAAGAGAAAAAATAGCTAAATCAACAGAAGCTCCAGCATGAGCTGTTCCTGAAGAGAGAGGAGGATAAACTGTTCACCCAGTCCCTGCCCCATTTTCTACTATTGAGCTTGATAATAGAAGAGTCAATGAAGGAGGTAGTATTCAAAAACTTATATTATTCATTCGAGGGAAAGCTATATCAGGGGCTCCTAATATTAAAGGAACTAATCAATTTCCAAATTCCTCCCATA

>DN_DMi_03F1

GTGGGTCAAGATCAATAAGTGTTGGTATAAAATAGGGTCTCCCCCTCCGATTGGATCAAAGAAAGATGTATTTAAGTTTCGGTCTGTTAATAATATAGTAATAGCTCCAGCTAAAACAGGAAGAGAAAGAAGTAATAAGATAGCTGTAATAACTACAGATCAAACAAATAAGGGTAGTCGATCTAAAGTAATCCCTGACGATCGCATATTAATCACAGTTGTAATAAAATTTACTGCCCCTAAAATTGAGGAAATTCCAGCTAAATGAAGAGAAAAAATAGCTAAATCAACAGAAGCTCCAGCATGAGCTGTTCCTGAAGAGAGAGGAGGATAAACTGTTCACCCAGTCCCTGCCCCATTTTCTACTATTGAGCTTGATAATAGAAGAGTCAATGAAGGAGGTAGTATTCAAAAACTTATATTATTCATTCGAGGGAAAGCTATATCAGGGGCTCCTAATATTAAAGGAACTAATCAATTTCCAAAATTCCTCCCA

>DN_DMi_03F2

CTGCGATCACTATCCAATTAACTAGTTCSTCTAAATAGGGTCTCCCCCTCCGATTGGATCAAAGAAAGATGTATTTAAGTTTCGGTCTGTTAATAATATAGTAATAGCTCCAGCTAAAACAGGAAGAGAAAGAAGTAATAAGATAGCTGTAATAACTACAGATCAAACAAATAAGGGTAGTCGATCTAAAGTAATCCCTGACGATCGCATATTAATCACAGTTGTAATAAAATTTACTGCCCCTAAAATTGAGGAAATTCCAGCTAAATGAAGAGAAAAAATAGCTAAATCAACAGAAGCTCCAGCATGAGCTGTTCCTGAAGAGAGAGGAGGATAAACTGTTCACCCAGTTCCTGCCCCATTTTCTACTATTGAGCTTGATAATAGAAGAGTCAATGAAGGAGGTAGTATTCAAAAACTTATATTATTCATTCGAGGGAAAGCTATATCAGGGGCTCCTAATATTAAAGGAACTAATCAATTTCAAAATCCTCC

>DN_DMi_03F3

GTGGGGGTCAAGTATCAGATAAGTGTTGGTATAAAATAGGGTCTCCCCCTCCGATTGGATCAAAGAAAGATGTATTTAAGTTTCGGTCTGTTAATAATATAGTAATAGCTCCAGCTAAAACAGGAAGAGAAAGAAGTAATAAGATAGCTGTAATAACTACAGATCAAACAAATAAGGGTAGTCGATCTAAAGTAATCCCTGACGATCGCATATTAATCACAGTTGTAATAAAATTTACTGCCCCTAAAATTGAGGAAATTCCAGCTAAATGAAGAGAAAAAATAGCTAAATCAACAGAAGCTCCAGCATGAGCTGTTCCTGAAGAGAGAGGAGGATAAACTGTTCACCCAGTTCCTGCCCCATTTTCTACTATTGAGCTTGATAATAGAAGAGTCAATGAAGGAGGTAGTATTCAAAAACTTATATTATTCATTCGAGGGAAAGCTATATCAGGGGCTCCTAATATTAAAGGAACTAATCAACTACAAAATTCCCCCACACA

>DN_DMi_03F4

GGGGGGTCAAGATCAGATAAGTGTTGGTATAAAATAGGGTCTCCCCCTCCGATTGGATCAAAGAAAGATGTATTTAAGTTTCGGTCTGTTAATAATATAGTAATAGCTCCAGCTAAAACAGGAAGAGAAAGAAGTAATAAGATAGCTGTAATAACTACAGATCAAACAAATAAGGGTAGTCGATCTAAAGTAATCCCTGACGATCGCATATTAATCACAGTTGTAATAAAATTTACTGCCCCTAAAATTGAGGAAATTCCAGCTAAATGAAGAGAAAAAATAGCTAAATCAACAGAAGCTCCAGCATGAGCTGTTCCTGAAGAGAGAGGAGGATAAACTGTTCACCCAGTTCCTGCCCCATTTTCTACTATTGAGCTTGATAATAGAAGAGTCAATGAAGGAGGTAGTATTCAAAAACTTATATTATTCATTCGAGGGAAAGCTATATCAGGGGCTCCTAATATTAAAGGAACTAATCAATTACCAAATTCCCTCCACACA

>DN_DMi_03F5

GTGCGGTCAGTATCAGATAAGTGTTGCGTATAAAATAGGGTCTCCCCCTCCGATTGGATCAAAGAAAGATGTATTTAAGTTTCGGTCTGTTAATAATATAGTAATAGCTCCAGCTAAAACAGGAAGAGAAAGAAGTAATAAGATAGCTGTAATAACTACAGATCAAACAAATAAGGGTAGTCGATCTAAAGTAATCCCTGACGATCGCATATTAATCACAGTTGTAATAAAATTTACTGCCCCTAAAATTGAGGAAATTCCAGCTAAATGAAGAGAAAAAATAGCTAAATCAACAGAAGCTCCAGCATGAGCTGTTCCTGAAGAGAGAGGAGGATAAACTGTTCACCCAGTCCCTGCCCCATTTTCTACTATTGAGCTTGATAATAGAAGAGTCAATGAAGGAGGTAGTATTCAAAAACTTATATTATTCATTCGAGGGAAAGCTATATCAGGGGCTCCTAATATTAAAGGAACTAATCAATAACCAAATTCCCCCCCAGA

>DN_DMi_03F6

GTGGGGTCAAGATCAAATAAGTGTTGGTATAAAATAGGGTCTCCCCCTCCGATTGGATCAAAGAAAGATGTATTTAAGTTTCGGTCTGTTAATAATATAGTAATAGCTCCAGCTAAAACAGGAAGAGAAAGAAGTAATAAGATAGCTGTAATAACTACAGATCAAACAAATAAGGGTAGTCGATCTAAAGTAATCCCTGACGATCGCATATTAATCACAGTTGTAATAAAATTTACTGCCCCTAAAATTGAGGAAATTCCAGCTAAATGAAGAGAAAAAATAGCTAAATCAACAGAAGCTCCAGCATGAGCTGTTCCTGAAGAGAGAGGAGGATAAACTGTTCACCCAGTCCCTGCCCCATTTTCTACTATTGAGCTTGATAATAGAAGAGTCAATGAAGGAGGTAGTATTCAAAAACTTATATTATTCATTCGAGGGAAAGCTATATCAGGGGCTCCTAATATTAAAGGAACTAATCAATTACAAAATTCCCTCCACGA

>DN_DMi_03F7

GGGGGGTTCAAAGATCAAATAAGTGTTGGTATAAAATAGGGTCTCCCCCTCCGATTGGATCAAAGAAAGATGTATTTAAGTTTCGGTCTGTTAATAATATAGTAATAGCTCCAGCTAAAACAGGAAGAGAAAGAAGTAATAAGATAGCTGTAATAACTACAGATCAAACAAATAAGGGTAGTCGATCTAAAGTAATCCCTGACGATCGCATATTAATCACAGTTGTAATAAAATTTACTGCCCCTAAAATTGAGGAAATTCCAGCTAAATGAAGAGAAAAAATAGCTAAATCAACAGAAGCTCCAGCATGAGCTGTTCCTGAAGAGAGAGGAGGATAAACTGTTCACCCAGTTCCTGCCCCATTTTCTACTATTGAGCTTGATAATAGAAGAGTCAATGAAGGAGGTAGTATTCAAAAACTTATATTATTCATTCGAGGGAAAGCTATATCAGGGGCTCCTAATATTAAAGGAACTAATCAATTACAAAATTCCCTCCCGAT

>DN_DMi_03F8

GGTGGGTCAAGGATCAAAATAAGTGTTGGTATAAAATAGGGTCTCCCCCTCCGATTGGATCAAAGAAAGATGTATTTAAGTTTCGGTCTGTTAATAATATAGTAATAGCTCCAGCTAAAACAGGAAGAGAAAGAAGTAATAAGATAGCTGTAATAACTACAGATCAAACAAATAAGGGTAGTCGATCTAAAGTAATCCCTGACGATCGCATATTAATCACAGTTGTAATAAAATTTACTGCCCCTAAAATTGAGGAAATTCCAGCTAAATGAAGAGAAAAAATAGCTAAATCAACAGAAGCTCCAGCATGAGCTGTTCCTGAAGAGAGAGGAGGATAAACTGTTCACCCAGTCCCTGCCCCATTTTCTACTATTGAGCTTGATAATAGAAGAGTCAATGAAGGAGGTAGTATTCAAAAACTTATATTATTCATTCGAGGGAAAGCTATATCAGGGGCTCCTAATATTAAAGGAACTAATCAATTTCCAAATTCCTCCA

>DN_DMi_03F9

GAGGGGGTAAAGATCAAATAAGTGTTGGTATAAAATAGGGTCTCCCCCTCCGATTGGATCAAAGAAAGATGTATTTAAGTTTCGGTCTGTTAATAATATAGTAATAGCTCCAGCTAAAACAGGAAGAGAAAGAAGTAATAAGATAGCTGTAATAACTACAGATCAAACAAATAAGGGTAGTCGATCTAAAGTAATCCCTGACGATCGCATATTAATCACAGTTGTAATAAAATTTACTGCCCCTAAAATTGAGGAAATTCCAGCTAAATGAAGAGAAAAAATAGCTWAATCAACAGAAGCTCCAGCATGAGCTGTTCCTGAAGAGAGAGGAGGATAAACTGTTCACCCAGTTCCTGCCCCATTTTCTACTATTGAGCTTGATAATAGAAGAGTCAATGAAGGAGGTAGTATTCAAAAACTTATATTATTCATTCGAGGGAAAGCTATATCAGGGGCTCCTAATATTAAAGGAACTAATCAATTTCCAAATCCTCCCA

>DN_DMi_04F1

GGTGGGGTCAAGATCAATAAGTGTTGGTATAAAATAGGGTCTCCCCCTCCGATTGGATCAAAGAAAGATGTATTTAAGTTTCGGTCTGTTAATAATATAGTAATAGCTCCAGCTAAAACAGGAAGAGAAAGAAGTAATAAGATAGCTGTAATAACTACAGATCAAACAAATAAGGGTAGTCGATCTAAAGTAATCCCTGACGATCGCATATTAATCACAGTTGTAATAAAATTTACTGCCCCTAAAATTGAGGAAATTCCAGCTAAATGAAGAGAAAAAATAGCTAAATCAACAGAAGCTCCAGCATGAGCTGTTCCTGAAGAGAGAGGAGGATAAACTGTTCACCCAGTCCCTGCCCCATTTTCTACTATTGAGCTTGATAATAGAAGAGTCAATGAAGGAGGTAGTATTCAAAAACTTATATTATTCATTCGAGGGAAAGCTATATCAGGGGCTCCTAATATTAAAGGAACTAATCAATTTCCAAATTCCTCC

>DN_DMi_04F2

GGGGGTCAAGATCAAATAAGTGTTGGTATAAAATAGGGTCTCCCCCTCCGATTGGATCAAAGAAAGATGTATTTAAGTTTCGGTCTGTTAATAATATAGTAATAGCTCCAGCTAAAACAGGAAGAGAAAGAAGTAATAAGATAGCTGTAATAACTACAGATCAAACAAATAAGGGTAGTCGATCTAAAGTAATCCCTGACGATCGCATATTAATCACAGTTGTAATAAAATTTACTGCCCCTAAAATTGAGGAAATTCCAGCTAAATGAAGAGAAAAAATAGCTAAATCAACAGAAGCTCCAGCATGAGCTGTTCCTGAAGAGAGAGGAGGATAAACTGTTCACCCAGTCCCTGCCCCATTTTCTACTATTGAGCTTGATAATAGAAGAGTCAATGAAGGAGGTAGTATTCAAAAACTTATATTATTCATTCGAGGGAAAGCTATATCAGGGGCTCCTAATATTAAAGGAACTAATCAATTTCCAAACTCCTCCCATA

>DN_DMi_04F3

GGGGGTCAGATCAGATAAGTGTTGGTATAAAATAGGGTCTCCCCCTCCGATTGGATCAAAGAAAGATGTATTTAAGTTTCGGTCTGTTAATAATATAGTAATAGCTCCAGCTAAAACAGGAAGAGAAAGAAGTAATAAGATAGCTGTAATAACTACAGATCAAACAAATAAGGGTAGTCGATCTAAAGTAATCCCTGACGATCGCATATTAATCACAGTTGTAATAAAATTTACTGCCCCTAAAATTGAGGAAATTCCAGCTAAATGAAGAGAAAAAATAGCTAAATCAACAGAAGCTCCAGCATGAGCTGTTCCTGAAGAGAGAGGAGGATAAACTGTTCACCCAGTTCCTGCCCCATTTTCTACTATTGAGCTTGATAATAGAAGAGTCAATGAAGGAGGTAGTATTCAAAAACTTATATTATTCATTCGAGGGAAAGCTATATCAGGGGCTCCTAATATTAAAGGAACTAATCAATCTACAAAATTCCCTCCCACA

>DN_DMi_04F4

GGGGGTCAAGATCAAATAAGTGTTGGTATAAAATAGGGTCTCCCCCTCCGATTGGATCAAAGAAAGATGTATTTAAGTTTCGGTCTGTTAATAATATAGTAATAGCTCCAGCTAAAACAGGAAGAGAAAGAAGTAATAAGATAGCTGTAATAACTACAGATCAAACAAATAAGGGTAGTCGATCTAAAGTAATCCCTGACGATCGCATATTAATCACAGTTGTAATAAAATTTACTGCCCCTAAAATTGAGGAAATTCCAGCTAAATGAAGAGAAAAAATAGCTAAATCAACAGAAGCTCCAGCATGAGCTGTTCCTGAAGAGAGAGGAGGATAAACTGTTCACCCAGTTCCTGCCCCATTTTCTACTATTGAGCTTGATAATAGAAGAGTCAATGAAGGAGGTAGTATTCAAAAACTTATATTATTCATTCGAGGGAAAGCTATATCAGGGGCTCCTAATATTAAAGGAACTAATCAATCACCAAATTTCCTCCCCGA

>DN_DMi_04F5

GTGGGGTCAGATCAGATAAGTGTTGGTATAAAATAGGGTCTCCCCCTCCGATTGGATCAAAGAAAGATGTATTTAAGTTTCGGTCTGTTAATAATATAGTAATAGCTCCAGCTAAAACAGGAAGAGAAAGAAGTAATAAGATAGCTGTAATAACTACAGATCAAACAAATAAGGGTAGTCGATCTAAAGTAATCCCTGACGATCGCATATTAATCACAGTTGTAATAAAATTTACTGCCCCTAAAATTGAGGAAATTCCAGCTAAATGAAGAGAAAAAATAGCTAAATCAACAGAAGCTCCAGCATGAGCTGTTCCTGAAGAGAGAGGAGGATAAACTGTTCACCCAGTTCCTGCCCCATTTTCTACTATTGAGCTTGATAATAGAAGAGTCAATGAAGGAGGTAGTATTCAAAAACTTATATTATTCATTCGAGGGAAAGCTATATCAGGGGCTCCTAATATTAAAGGAACTAATCAATTTCAAAATTCCCTCCCACA

>DN_DMi_04F6

GTGGGGTCAAGATCAGATAAGTGTTGGTATAAAATAGGGTCTCCCCCTCCGATTGGATCAAAGAAAGATGTATTTAAGTTTCGGTCTGTTAATAATATAGTAATAGCTCCAGCTAAAACAGGAAGAGAAAGAAGTAATAAGATAGCTGTAATAACTACAGATCAAACAAATAAGGGTAGTCGATCTAAAGTAATCCCTGACGATCGCATATTAATCACAGTTGTAATAAAATTTACTGCCCCTAAAATTGAGGAAATTCCAGCTAAATGAAGAGAAAAAATAGCTAAATCAACAGAAGCTCCAGCATGAGCTGTTCCTGAAGAGAGAGGAGGATAAACTGTTCACCCAGTCCCTGCCCCATTTTCTACTATTGAGCTTGATAATAGAAGAGTCAATGAAGGAGGTAGTATTCAAAAACTTATATTATTCATTCGAGGGAAAGCTATATCAGGGGCTCCTAATATTAAAGGAACTAATCAATTTCCAAATTCCCTCCCAGA

>DN_DMi_04F7

ACTGGCGTTCAGTATCCAATAAGTGTTCCGTACAAAATAGGGTCTCCCCCTCCGATTGGATCAAAGAAAGATGTATTTAAGTTTCGGTCTGTTAATAATATAGTAATAGCTCCAGCTAAAACAGGAAGAGAAAGAAGTAATAAGATAGCTGTAATAACTACAGATCAAACAAATAAGGGTAGTCGATCTAAAGTAATCCCTGACGATCGCATATTAATCACAGTTGTAATAAAATTTACTGCCCCTAAAATTGAGGAAATTCCAGCTAAATGAAGAGAAAAAATAGCTAAATCAACAGAAGCTCCAGCATGAGCTGTTCCTGAAGAGAGAGGAGGATAAACTGTTCACCCAGTTCCTGCCCCATTTTCTACTATTGAGCTTGATAATAGAAGAGTCAATGAAGGAGGTAGTATTCAAAAACTTATATTATTCATTCGAGGGAAAGCTATATCAGGGGCTCCTAATATTAAAGGAACTAATCAATTTACAAATTCCTCCCACAC

>DN_DMi_04F8

GGTGGGGTTCAAGATCAAAATAAGTGTTGGTATAAAATAGGGTCTCCCCCTCCGATTGGATCAAAGAAAGATGTATTTAAGTTTCGGTCTGTTAATAATATAGTAATAGCTCCAGCTAAAACAGGAAGAGAAAGAAGTAATAAGATAGCTGTAATAACTACAGATCAAACAAATAAGGGTAGTCGATCTAAAGTAATCCCTGACGATCGCATATTAATCACAGTTGTAATAAAATTTACTGCCCCTAAAATTGAGGAAATTCCAGCTAAATGAAGAGAAAAAATAGCTAAATCAACAGAAGCTCCAGCATGAGCTGTTCCTGAAGAGAGAGGAGGATAAACTGTTCACCCAGTTCCTGCCCCATTTTCTACTATTGAGCTTGATAATAGAAGAGTCAATGAAGGAGGTAGTATTCAAAAACTTATATTATTCATTCGAGGGAAAGCTATATCAGGGGCTCCTAATATTAAAGGAACTAATCAATTTCCAAATTCCTCACA

>DN_DMi_05F1

CTTGGTACAAGTATTCAATTTACCCGATCCTCTAAAATAGGATCTCCTCCTCCGATTGGATCAAAGAAAGATGTATTTAAGTTTCGGTCTGTTAATAACATAGTATTAGCTCCAGCTAAAACAGGAAGAGAAAGAAGTAATAAGATAGCTGTAATAACTACAGATCAAACAAATAAAGGTAGTCGATCTAAAGTAATTCCTGATGATCGTATATTAATTACAGTTGTAATAAAATTTACTGCCCCTAAAATTGAGGAAATTCCCGCTAAATGAAGAGAAAAAATAGCTAAATCAACAGAAGCTCCAGCATGAGCTGTTCCTGAAGAGAGAGGAGGATAAACTGTTCACCCAGTTCCTGCCCCATTTTCTACTATTGAGCTAGATAATAGAAGAGTCAATGAGGGAGGTAGTATTCAAAAACTTATATTATTTATTCGAGGAAAGGCTATATCAGGGGCTCCTAATATTAAAGGAACTAATCAATTTCAAAATCCTCCA

>DN_DMi_05M1

GATGCGGTCAGTTCAGATTACCTGTTCCGTATAAAATAGGATCTCCTCCTCCGATTGGATCAAAGAAAGATGTATTTAAGTTTCGGTCTGTTAATAACATAGTAATAGCTCCAGCTAAAACAGGAAGAGAAAGAAGTAATAAGATAGCTGTAATAACTACAGATCAAACAAATAAAGGTAGTCGATCTAAAGTAATTCCTGACGATCGTATATTAATTACAGTTGTAATAAAATTTACTGCCCCTAAAATTGAGGAAATTCCAGCTAAATGAAGAGAAAAAATAGCTAAATCAACAGAAGCTCCAGCATGAGCTGTTCCTGAAGAGAGAGGAGGATAAACTGTTCACCCAGTTCCTGCCCCATTTTCTACTATTGAGCTCGATAATAGAAGAGTCAATGAAGGAGGTAGTATTCAAAAACTTATATTATTTATTCGAGGAAAGGCTATATCAGGGGCTCCTAATATTAAAGGAACTAATCAATTTCAAATTCCCCCCCACA

>DN_DMi_06F1

GGGGGGTCAGATCAGATAAGTGTTGGTATAAAATAGGGTCTCCCCCTCCGATTGGATCAAAGAAAGATGTATTTAAGTTTCGGTCTGTTAATAATATAGTAATAGCTCCAGCTAAAACAGGAAGAGAAAGAAGTAATAAGATAGCTGTAATAACTACAGATCAAACAAATAAGGGTAGTCGATCTAAAGTAATCCCTGACGATCGCATATTAATCACAGTTGTAATAAAATTTACTGCCCCTAAAATTGAGGAAATTCCAGCTAAATGAAGAGAAAAAATAGCTAAATCAACAGAAGCTCCAGCATGAGCTGTTCCTGAAGAGAGAGGAGGATAAACTGTTCACCCAGTCCCTGCCCCATTTTCTACTATTGAGCTTGATAATAGAAGAGTCAATGAAGGAGGTAGTATTCAAAAACTTATATTATTCATTCGAGGGAAAGCTATATCAGGGGCTCCTAATATTAAAGGAACTAATCAATTACCAAATTTCCCCCAACA

>DN_DMi_06F2

GGGGGGTTCAGATCAAATAAGTGTTGGTATAAAATAGGGTCTCCCCCTCCGATTGGATCAAAGAAAGATGTATTTAAGTTTCGGTCTGTTAATAATATAGTAATAGCTCCAGCTAAAACAGGAAGAGAAAGAAGTAATAAGATAGCTGTAATAACTACAGATCAAACAAATAAGGGTAGTCGATCTAAAGTAATCCCTGACGATCGCATATTAATCACAGTTGTAATAAAATTTACTGCCCCTAAAATTGAGGAAATTCCAGCTAAATGAAGAGAAAAAATAGCTAAATCAACAGAAGCTCCAGCATGAGCTGTTCCTGAAGAGAGAGGAGGATAAACTGTTCACCCAGTCCCTGCCCCATTTTCTACTATTGAGCTTGATAATAGAAGAGTCAATGAAGGAGGTAGTATTCAAAAACTTATATTATTCATTCGAGGGAAAGCTATATCAGGGGCTCCTAATATTAAAGGAACTAATCAATTACCAAATTCCCTCCCCAGGGGG

>DN_DMi_06F3

CCTCGTTCGAACTAATCATTTCCAGATCCTACCGACTAATCGATTTCCCAATCCTCCGATCTACTCAATTTCCAAATCCTCCTCGGTCTGTTAATTACATAGTATTAGCTCCCGCTAAAACAGGAAGAGAAAGAAGTAATAAGATAGCTTTTATAACTACCGATTAAACAAATTAGGGTAGTCGATCTTAAGTAATCCCTGACCATTGCAAATTAATCACAGTTGTAATAAAATTTACTGGCCCTCCATTGAGGAAATTCCCGCTTAATTAAGAGAAAAAATAGCTTAATTAACAGAAGCTCCCGCATGAGCTGTTCCTGAAGAGAGAGGAGGATAAACTGTTCACCCAGTTCCTGCCCCATTTTCTACTATTGAGCTTGATAATAGAAGAGTCAATGAAGGAGGGGGTATTCACAAACTTATATCATTCATTCGAGGGAGAGCTATATCAGGGGCTCCTAATATTAAAGGAGCTGATCCGTTTCCAAATCCTCCCCCAAAAA

>DN_DMi_06F4

GGGGGGTCAGATCAGATAAGTGTTGGTATAAAATAGGGTCTCCCCCTCCGATTGGATCAAAGAAAGATGTATTTAAGTTTCGGTCTGTTAATAATATAGTAATAGCTCCAGCTAAAACAGGAAGAGAAAGAAGTAATAAGATAGCTGTAATAACTACAGATCAAACAAATAAGGGTAGTCGATCTAAAGTAATCCCTGACGATCGCATATTAATCACAGTTGTAATAAAATTTACTGCCCCTAAAATTGAGGAAATTCCAGCTAAATGAAGAGAAAAAATAGCTAAATCAACAGAAGCTCCAGCATGAGCTGTTCCTGAAGAGAGAGGAGGATAAACTGTTCACCCAGTCCCTGCCCCATTTTCTACTATTGAGCTTGATAATAGAAGAGTCAATGAAGGAGGTAGTATTCAAAAACTTATATTATTCATTCGAGGGAAAGCTATATCAGGGGCTCCTAATATTAAAGGAACTAATCAATTACCAAATTTCCTCCCCACA

>DN_DMi_06F5

GTGGGGTCAGTATCAGATAAGTGTTGGTATAAAATAGGGTCTCCCCCTCCGATTGGATCAAAGAAAGATGTATTTAAGTTTCGGTCTGTTAATAATATAGTAATAGCTCCAGCTAAAACAGGAAGAGAAAGAAGTAATAAGATAGCTGTAATAACTACAGATCAAACAAATAAGGGTAGTCGATCTAAAGTAATCCCTGACGATCGCATATTAATCACAGTTGTAATAAAATTTACTGCCCCTAAAATTGAGGAAATTCCAGCTAAATGAAGAGAAAAAATAGCTAAATCAACAGAAGCTCCAGCATGAGCTGTTCCTGAAGAGAGAGGAGGATAAACTGTTCACCCAGTTCCTGCCCCATTTTCTACTATTGAGCTTGATAATAGAAGAGTCAATGAAGGAGGTAGTATTCAAAAACTTATATTATTCATTCGAGGGAAAGCTATATCAGGGGCTCCTAATATTAAAGGAACTAATCAATTACCAAATTCCCCCCAACA

>DN_DL_07M1

GGCGCGGTCAAGTATCAGATTAGTGTTCCGTATAAAATAGGATCTCCTCCTCCGATTGGATCAAAGAAAGATGTATTTAAGTTTCGGTCTGTTAATAACATAGTAATAGCTCCAGCTAAAACAGGAAGAGAAAGAAGTAATAAGATAGCTGTAATAACTACAGATCAAACAAATAAAGGTAGTCGGTCTAAAGTAATTCCTGACGATCGTATATTAATTACAGTTGTAATAAAATTTACTGCCCCTAAAATTGAGGAAATTCCAGCTAAATGAAGAGAAAAAATAGCTAAATCAACAGAAGCTCCAGCATGAGCTGTTCCTGAAGAGAGAGGAGGATAAACTGTTCACCCAGTTCCTGCCCCATTTTCTACTATTGAGCTTGATAATAGAAGAGTCAATGAAGGAGGTAGTATTCAAAAACTTATATTATTTATTCGAGGAAAGGCTATATCAGGGGCTCCTAATATTAAAGGAACTAATCAATTTCCAAATTCCTCCCACA

>DN_DL_7M2

ACTACGTCAAAGTATCATATACGTGTTGGTACAAAATAGGGTCTCCCCCTCCGATTGGATCAAAGAAAGATGTATTTAAGTTTCGGTCTGTTAATAATATAGTAATAGCTCCAGCTAAAACAGGAAGAGAAAGAAGTAATAAGATAGCTGTAATAACTACAGATCAAACAAATAAGGGTAGTCGATCTAAAGTAATCCCTGACGATCGCATATTAATCACAGTTGTAATAAAATTTACTGCCCCTAAAATTGAGGAAATTCCAGCTAAATGAAGAGAAAAAATAGCTAAATCAACAGAAGCTCCAGCATGAGCTGTTCCTGAAGAGAGAGGAGGATAAACTGTTCACCCAGTCCCTGCCCCATTTTCTACTATTGAGCTTGATAATAGAAGAGTCAATGAAGGAGGTAGTATTCAAAAACTTATATTATTCATTCGAGGAAAAGCTATATCAGGGGCTCCTAATATTAAAGGAACTAATCAATTTCCAAATCCCTCCA

>DN_DL_7M3

GGGGGGTTTAAGTAATCAGATAAGTGTTGGTATAAAATAGGATCTCCTCCTCCGATTGGATCAAAGAAAGATGTATTTAAGTTTCGGTCTGTTAATAACATAGTAATAGCTCCAGCTAAAACAGGAAGAGAAAGAAGTAATAAGATAGCTGTAATAACTACAGATCAAACAAATAAAGGTAGTCGATCTAAAGTAATTCCTGACGATCGTATATTAATTACAGTTGTAATAAAATTTACTGCCCCTAAAATTGAGGAAATTCCAGCTAAATGAAGAGAAAAAATAGCTAAATCAACAGAAGCTCCAGCATGAGCTGTTCCTGAAGAGAGAGGAGGATAAACTGTTCACCCAGTTCCTGCTCCATTTTCTACTATTGAGCTTGATAATAGAAGAGTCAATGAAGGAGGTAGTATTCAAAAACTTATATTATTTATTCGAGGAAAGGCTATATCAGGGGCTCCTAATATTAAAGGAACTAATCAATTTCCAAATTCCCCCCA

>DN_DL_7M4

GGTGCGTTCAGTATCACGATCACCTGATCCGTACAAAATAGGGTCTCCCCCTCCGATTGGATCAAAGAAAGATGTATTTAAGTTTCGGTCTGTTAATAATATAGTAATAGCTCCAGCTAAAACAGGAAGAGAAAGAAGTAATAAGATAGCTGTAATAACTACAGATCAAACAAATAAGGGTAGTCGATCTAAAGTAATCCCTGACGATCGCATATTAATCACAGTTGTAATAAAATTTACTGCCCCTAAAATTGAGGAAATTCCAGCTAAATGAAGAGAAAAAATAGCTAAATCAACAGAAGCTCCAGCATGAGCTGTTCCTGAAGAGAGAGGAGGATAAACTGTTCACCCAGTCCCTGCCCCATTTTCTACTATTGAGCTTGATAATAGAAGAGTCAATGAAGGAGGTAGTATTCAAAAACTTATATTATTCATTCGAGGGAAAGCTATATCAGGGGCTCCTAATATTAAAGGAACTAATCAATTTCCAAATTCCCCCCA

>DN_DL_7M6

ACGTCTGCAACTATCATTTCCAATCCTACCCACTAGTACTTTCCAAATCCTCCGAACTAATCATTTTYCAAATCCTCCCGGTCAGTTATAATATAATCCTMCACTCCGGCTAATACGGGTAGAGAAAGAAGTAATAAAATAGCTGTAATTACTACTGATCACACAAATAAAGGTAGTCGATCAAGAGTAATACCAGCTGATCGTATATTAATTACAGTTGTAATAAAATTTACTGCTCCTAAAATAGATGAGATTCCCGCTAAATGTAAAGAAAAAATTGCTAAATCAACTGAAGCCCCAGCATGAGCTGTTCCAGAAGAAAGGGGGGGATAAACCGTTCACCCTGTTCCAGCTCCGTTTTCTACTATAGAACTAGAAAGCAGCAGTGTTAAAGGGGGGGGTAATATTCAAAAACTTATATTATTTATTCGAGGAAAAGCTATATCAGGGGCTCCTAGTATTAAGGGAACTAATCAATTTCCAAATCCTCCA

>DN_DL_7M7

GTTGGGTTACAGTAATSAGATAAGTGTTGGTATAAAATAGGGTCTCCCCCTCCGATTGGGTCAAAGAAAGATGTATTTAAGTTTCGGTCTGTTAATAATATAGTAATAGCTCCAGCTAAAACAGGAAGAGAAAGAAGTAATAAGATAGCTGTAATAACTACAGATCAAACAAATAAGGGTAGTTGATCTAAAGTAATTCCTGACGATCGTATATTAATTACAGTTGTAATAAAATTTACTGCCCCTAAAATTGAGGAAATTCCAGCTAAATGAAGAGAAAAAATAGCTAAATCAACAGAAGCTCCAGCATGAGCTGTTCCTGAAGAGAGAGGAGGATAAACTGTTCACCCAGTTCCTGCCCCATTTTCTACTATTGAGCTTGATAATAGAAGAGTCAATGAAGGAGGTAGTATTCAAAAACTTATATTATTCATTCGAGGGAAAGCTATATCAGGGGCTCCTAATATTAAAGGAACTAATCAATTTCCAAATTCCCCCCCA

>DN_DL_7M8

GATGGGTTCAGATCAGAATAAGTGTTGGTATAAAATAGGATCTCCTCCTCCGATTGGATCAAAGAAAGATGTATTTAAGTTTCGGTCTGTTAATAACATAGTAATAGCTCCAGCTAAAACAGGAAGAGAAAGAAGTAATAAGATAGCTGTAATAACTACAGATCAAACAAATAAAGGTAGTCGGTCTAAAGTAATTCCTGACGATCGTATATTAATTACAGTTGTAATAAAATTTACTGCCCCTAAAATTGAGGAAATTCCAGCTAAATGAAGAGAAAAAATAGCTAAATCAACAGAAGCTCCAGCATGAGCTGTTCCTGAAGAGAGAGGAGGATAAACTGTTCACCCAGTTCCTGCCCCATTTTCTACTATTGAGCTTGATAATAGAAGAGTCAATGAAGGAGGTAGTATTCAAAAACTTATATTATTTATTCGAGGAAAGGCTATATCAGGGGCTCCTAATATTAAAGGAACTAATCAATTCCAAATTCCCCCACA

>DN_DL_7M9

GATGCGTCAGTATCAGATTGTGTTGCGTACAAAATAGGGTCTCCCCCTCCGATTGGATCAAAGAAAGATGTATTTAAGTTTCGGTCTGTTAATAATATAGTAATAGCTCCAGCTAAAACAGGAAGAGAAAGAAGTAATAAGATAGCTGTAATAACTACAGATCAAACAAATAAGGGTAGTCGATCTAAAGTAATCCCTGACGATCGCATATTAATCACAGTTGTAATAAAATTTACTGCCCCTAAAATTGAGGAAATTCCAGCTAAATGAAGAGAAAAAATAGCTAAATCAACAGAAGCTCCAGCATGAGCTGTTCCTGAAGAGAGAGGAGGATAAACTGTTCACCCAGTCCCTGCCCCATTTTCTACTATTGAGCTTGATAATAGAAGAGTCAATGAAGGAGGTAGTATTCAAAAACTTATATTATTCATTCGAGGGAAAGCTATATCAGGGGCTCCTAATATTAAAGGAACTAATCAATTCCAAAATTCCCCCCA

>DN_DL_7M10

GTTGGGTTCAAGTAATCAGATAAGTGTTGGTATAAAATAGGGTCTCCCCCTCCGATTGGATCAAAGAAAGATGTATTTAAGTTTCGGTCTGTTAATAATATAGTAATAGCTCCAGCTAAAACAGGAAGAGAAAGAAGTAATAAGATAGCTGTAATAACTACAGATCAAACAAATAAGGGTAGTCGATCTAAAGTAATCCCTGACGATCGCATATTAATCACAGTTGTAATAAAATTTACTGCCCCTAAAATTGAGGAAATTCCAGCTAAATGAAGAGAAAAAATAGCTAAATCAACAGAAGCTCCAGCATGAGCTGTTCCTGAAGAGAGAGGAGGATAAACTGTTCACCCAGTCCCTGCCCCATTTTCTACTATTGAGCTTGATAATAGAAGAGTCAATGAAGGAGGTAGTATTCAAAAACTTATATTATTCATTCGAGGGAAAGCTATATCAGGGGCTCCTAATATTAAAGGAACTAATCAATTTCCAAATTCCCCCCA

>DN_DM_8F1

GGGGGGTCACGATCAGATAAGTGTTGGTATAAAATAGGATCTCCTCCTCCGATTGGATCGAAGAAAGATGTATTTAAGTTTCGGTCTGTTAATAACATAGTAATAGCTCCGCTAAAACAGGAAGAGAAAGAAGTAATAAGATAGCTGTAATAACTACAGATCAAACAAATAAAGGTAGTCGGTCTAAAGTAATTCCTGACGATCGTATATTAATTACAGTTGTAATAAAATTTACTGCCCCTAAAATTGAGGAAATTCCAGCTAAATGGAGAGAAAAAATAGCTAAATCAACAGAAGCTCCCGCATGAGCTGTTCCTGAAGAGAGAGGAGGATACACTGTTCACCCAGTTCCTGCCCCATTTTCTACTATTGAGCTTGATAATAGAAGAGTCAATGAAGGAGGTAGTATTCAAAAACTTATATTATTTATTCGAGGAAAGGCTATATCAGGGGCTCCTAATATTAAAGGAACTAATCAATTTCCTAAATCCTCC

>DN_DM_8F2

ATTCGGCGACTATCAATTTCCTAGATCCGTCTCAATAGGGTCTCCCCCTCTGCATTGAGATCAAAGAAAGATGTATTTCAGTTACGGTCTGTTAATAATATAGTAATAGCTCCAGCTAAAACAGGAAGAGAAAGAAGTAATAAGATAGCTGTAATAACTACAGATCAAACAAATAAGGGTAGTCGATCTAAAGTAATCCCTGACGATCGCATATTAATCACAGTTGTAATAAAATTTACTGCCCCTAAAATTGAGGAAATTCCAGCTAAATGAAGAGAAAAAATAGCTAAATCAACAGAAGCTCCAGCATGAGCTGTTCCTGAAGAGAGAGGAGGATAAACTGTTCACCCAGTCCCTGCCCCATTTTCTACTATTGAGCTTGATAATAGAAGAGTCAATGAAGGAGGTAGTATTCAAAAACTTATATTATTCATTCGAGGGAAAGCTATATCAGGGGCTCCTAATATTAAAGGAACTAATCAATTTCCAAATTCCTCCAA

>DN_DM_8F3

GGGGGGTTCAAGTATCAGATAAGTGTTGGTATAAAATAGGATCTCCTCCTCCGATTGGATCGAAGAAAGATGTATTTAAGTTTCGGTCTGTTAATAACATAGTAATAGCTCCAGCTAAAACAGGAAGAGAAAGAAGTAATAAGATAGCTGTAATAACTACAGATCAAACAAATAAAGGTAGTCGGTCTAAAGTAATTCCTGACGATCGTATATTAATTACAGTTGTAATAAAATTTACTGCCCCTAAAATTGAGGAAATTCCAGCTAAATGAAGAGAAAAAATAGCTAAATCAACAGAAGCTCCAGCATGAGCTGTTCCTGAAGAGAGAGGAGGATACACTGTTCACCCAGTTCCTGCCCCATTTTCTACTATTGAGCTTGATAATAGAAGAGTCAATGAAGGAGGTAGTATTCAAAAACTTATATTATTTATTCGAGGAAAGGCTATATCAGGGGCTCCTAATATTAAAGGAACTAATCAATTTCAAAATTCCCCCCA

>DN_DM_8F4

GTGGGGTCAAGTAATCAGATAAGTGTTGGTATAAAATAGGGTCTCCTCCTCCGATTGGATCAAAGAAAGATGTATTTAAGTTTCGGTCTGTTAATAACATAGTAATAGCTCCAGCTAAAACAGGAAGAGAAAGAAGTAATAAGATAGCTGTAATAACTACAGATCAAACAAATAAAGGTAGTCGATCTAAAGTAATTCCTGACGATCGTATATTAATTACAGTTGTAATAAAATTTACTGCCCCTAAAATTGAGGAAATTCCAGCTAAATGAAGAGAAAAAATAGCTAAATCAACAGAAGCTCCAGCATGAGCTGTTCCTGAAGAGAGAGGAGGATAAACTGTTCACCCAGTTCCTGCCCCATTTTCTACTATTGAGCTTGATAATAGAAGAGTCAATGAAGGAGGTAGTATTCAAAAACTTATATTATTTATTCGAGGAAAGGCTATATCAGGGGCTCCTAATATTAAAGGAACTAATCATTTACAAATTCCCCCCA

>DN_DM_8F5

GTTGGCGTTCAGTAATGAGATAAGTGTTGGTATAAAATAGGGTCTCCTCCTCCGATTGGATCAAAGAAAGATGTATTTAAGTTTCGGTCTGTTAATAACATAGTAATAGCTCCAGCTAAAACAGGAAGAGAAAGAAGTAATAAGATAGCTGTAATAACTACAGATCAAACAAATAAAGGTAGTCGATCTAAAGTAATTCCTGACGATCGTATATTAATTACAGTTGTAATAAAATTTACTGCCCCTAAAATTGAGGAAATTCCAGCTAAATGAAGAGAAAAAATAGCTAAATCAACAGAAGCTCCAGCATGAGCTGTTCCTGAAGAGAGAGGAGGATAAACTGTTCACCCAGTTCCTGCCCCATTTTCTACTATTGAGCTTGATAATAGAAGAGTCAATGAAGGAGGTAGTATTCAAAAACTTATATTATTTATTCGAGGAAAG

GCTATATCAGGGGCTCCTAATATTAAAGGAACTAATCAATTTCCAAATTCCCCCCA

>DN_DM_8F6

GTGGGTCAAGATCAGATAAGTGTTGGTATAAAATAGGGTCTCCTCCTCCGATTGGATCAAAGAAAGATGTATTTAAGTTTCGGTCTGTTAATAACATAGTAATAGCTCCAGCTAAAACAGGAAGAGAAAGAAGTAATAAGATAGCTGTAATAACTACAGATCAAACAAATAAAGGTAGTCGATCTAAAGTAATTCCTGACGATCGTATATTAATTACAGTTGTAATAAAATTTACTGCCCCTAAAATTGAGGAAATTCCAGCTAAATGAAGAGAAAAAATAGCTAAATCAACAGAAGCTCCAGCATGAGCTGTTCCTGAAGAGAGAGGAGGATAAACTGTTCACCCAGTTCCTGCCCCATTTTCTACTATTGAGCTTGATAATAGAAGAGTCAATGAAGGAGGTAGTATTCAAAAACTTATATTATTTATTCGAGGAAAGGCTATATCAGGGGCTCCTAATATTAAAGGAACTAATCAATTTCCAAACCCCTCCCA

>DN_DM_9M1

TGTGGGGTCAGATCAAATAAGTGTTGGTATAAAATAGGGTCTCCCCCTCCGATTGGATCAAAGAAAGATGTATTTAAGTTTCGGTCTGTTAATAATATAGTAATAGCTCCAGCTAAAACAGGAAGAGAAAGAAGTAATAAGATAGCTGTAATAACTACAGATCAAACAAATAAGGGTAGTCGATCTAAAGTAATCCCTGACGATCGCATATTAATCACAGTTGTAATAAAATTTACTGCCCCTAAAATTGAGGAAATTCCAGCTAAATGAAGAGAAAAAATAGCTAAATCAACAGAAGCTCCAGCATGAGCTGTTCCTGAAGAGAGAGGAGGATAAACTGTTCACCCAGTCCCTGCCCCATTTTCTACTATTGAGCTTGATAATAGAAGAGTCAATGAAGGAGGTAGTATTCAAAAACTTATATTATTCATTCGAGGGAAAGCTATATCAGGGGCTCCTAATATTAAAGGAACTAATCAATTTCCAAATTCCTCCCA

>DN_DM_9M2

GTTGGGGTTCAGATCAGATAAGTGTTGGTATAAAATAGGGTCTCCTCCTCCGATTGGATCAAAGAAAGATGTATTTAAGTTTCGGTCTGTTAATAACATAGTAATAGCTCCAGCTAAAACAGGAAGAGAAAGAAGTAATAAGATAGCTGTAATAACTACAGATCAAACAAATAAAGGTAGTCGATCTAAAGTAATTCCTGACGATCGTATATTAATTACAGTTGTAATAAAATTTACTGCCCCTAAAATTGAGGAAATTCCAGCTAAATGAAGAGAAAAAATAGCTAAATCAACAGAAGCTCCAGCATGAGCTGTTCCTGAAGAGAGAGGAGGATAAACTGTTCACCCAGTTCCTGCCCCATTTTCTACTATTGAGCTTGATAATAGAAGAGTCAATGAAGGAGGTAGTATTCAAAAACTTATATTATTTATTCGAGGAAAGGCTATATCAGGGGCTCCTAATATTAAAGGAACTAATCAAAAACAAAATTCCCCCAA

>DN_DM_9M3

CCTCGGTCAGTATTACGATTACCTGATCCGTCCCAAATAGGGTCTCCTCCTCCGATTGAGATCAAAGAAAGATGTATTTAAGTTTCGGTCTGTTAATAACATAGTAATAGCTCCAGCTAAAACAGGAAGAGAAAGAAGTAATAAGATAGCTGTAATAACTACAGATCAAACAAATAAAGGTAGTCGATCTAAAGTAATTCCTGACGATCGTATATTAATTACAGTTGTAATAAAATTTACTGCCCCTAAAATTGAGGAAATTCCAGCTAAATGAAGAGAAAAAATAGCTAAATCAACAGAAGCTCCAGCATGAGCTGTTCCTGAAGAGAGAGGAGGATAAACTGTTCACCCAGTTCCTGCCCCATTTTCTACTATTGAGCTTGATAATAGAAGAGTCAATGAAGGAGGTAGTATTCAAAAACTTATATTATTTATTCGAGGAAAGGCTATATCAGGGGCTCCTAATATTAAAGGAACTAATCAATTTCCAAATTCCTCCA

>DN_DM_9M4

GTGCGTCAGATCAGATTAGTGTTCCGTATAAAATAGGGTCTCCTCCTCCGATTGGATCAAAGAAAGATGTATTTAAGTTTCGGTCTGTTAATAACATAGTAATAGCTCCAGCTAAAACAGGAAGAGAAAGAAGTAATAAGATAGCTGTAATAACTACAGATCAAACAAATAAAGGTAGTCGATCTAAAGTAATTCCTGACGATCGTATATTAATTACAGTTGTAATAAAATTTACTGCCCCTAAAATTGAGGAAATTCCAGCTAAATGAAGAGAAAAAATAGCTAAATCAACAGAAGCTCCAGCATGAGCTGTTCCTGAAGAGAGAGGAGGATAAACTGTTCACCCAGTTCCTGCCCCATTTTCTACTATTGAGCTTGATAATAGAAGAGTCAATGAAGGAGGTAGTATTCAAAAACTTATTTAAAATTCCCTCCCA

>DN_DM_9M5

GGGGCTCAAGATCAGATAAGTGTTGGTATAAAATAGGGTCTCCTCCTCCGATTGGATCAAAGAAAGATGTATTTAAGTTTCGGTCTGTTAATAAYATAGTAATAGCTCCAGCTAAAACAGGAAGAGAAAGAAGTAATAAGATAGCTGTAATAACTACAGATCAAACAAATAARGGTAGTCGATCTAAAGTAATYCCTGACGATCGYATATTAATYACAGTTGTAATAAAATTTACTGCCCCTAAAATTGAGGAAATTCCAGCTAAATGAAGAGAAAAAATAGCTAAATCAACAGAAGCTCCAGCATGAGCTGTTCCTGAAGAGAGAGGAGGATAAACTGTTCACCCAGTYCCTGCCCCATTTTCTACTATTGAGCTTGATAATAGAAGAGTCAATGAAGGAGGTAGTATTCAAAAACTTATATTATTYATTCGAGGRAARGCTATATCAGGGGCTCCTAATATTAAAGGAACTAATCAATTTCCAAAATTCCTTCCA

>LA_BHT_13F1

AGATCAGAATAAGTGTTGGTATAAAATAGGATCTCCTCCTCCGATTGGATCAAAGAAAGATGTATTTAAGTTTCGGTCTGTTAATAACATAGTAATAGCTCCAGCTAAAACAGGAAGAGAAAGAAGTAATAAGATAGCTGTAATAACTACAGATCAAACAAATAAAGGTAGTCGATCTAAAGTAATTCCTGACGATCGTATATTAATTACAGTTGTAATAAAATTTACTGCCCCTAAAATTGAGGAAATTCCAGCTAAATGAAGAGAAAAAATAGCTAAATCAACAGAAGCTCCAGCATGAGCTGTTCCTGAAGAGAGAGGAGGATAAACTGTTCACCCAGTTCCTGCTCCATTTTCTACTATTGAGCTTGATAATAGAAGAGTCAATGAAGGAGGTAGTATTCAAAAACTTATATTATTTATTCGAGGAAAGGCTATATCAGGGGCTCCTAATATTAAAGGAACTAATCAATTTCAAAATCCCTCCA

>LA_BHT_13F2

GAATAAGTGTTGGTATAAAATAGGATCTCCTCCTCCGATTGGATCAAAGAAAGATGTATTTAAGTTTCGGTCTGTTAATAACATAGTAATAGCTCCAGCTAAAACAGGAAGAGAAAGAAGTAATAAGATAGCTGTAATAACTACAGATCAAACAAATAAAGGTAGTCGATCTAAAGTAATTCCTGACGATCGTATATTAATTACAGTTGTAATAAAATTTACTGCCCCTAAAATTGAGGAAATTCCAGCTAAATGAAGAGAAAAAATAGCTAAATCAACAGAAGCTCCAGCATGAGCTGTTCCTGAAGAGAGAGGAGGATAAACTGTTCACCCAGTTCCTGCTCCATTTTCTACTATTGAGCTTGATAATAGAAGAGTCAATGAAGGAGGTAGTATTCAAAAACTTATATTATTTATTCGAGGAAAGGCTATATCAGGGGCTCCTAATATTAAAGGAACTAATCAATTCCAAATTTCCCTCCCA

>LA_BH_13F3

GTGGCGTCAGATCAGATAAGTGTTGGTATAAAATAGGATCTCCTCCTCCGATTGGATCAAAGAAAGATGTATTTAAGTTTCGGTCTGTTAATAACATAGTAATAGCTCCAGCTAAAACAGGAAGAGAAAGAAGTAATAAGATAGCTGTAATAACTACAGATCAAACAAATAAAGGTAGTCGGTCTAAAGTAATTCCTGACGATCGTATATTAATTACAGTTGTAATAAAATTTACTGCCCCTAAAATTGAGGAAATTCCAGCTAAATGAAGAGAAAAAATAGCTAAATCAACAGAAGCTCCAGCATGAGCTGTTCCTGAAGAGAGAGGAGGATAAACTGTTCACCCAGTTCCTGCCCCATTTTCTACTATTGAGCTTGATAATAGAAGAGTCAATGAAGGAGGTAGTATTCAAAAACTTATATTATTTATTCGAGGAAAGGCTATATCAGGGGCTCCTAATATTAAAGGAACTAATCAATTTCAAAATTCCCTCCCA

>LA_BH_13F4

GTGGGTCAGATCAGAATAAGTGTTGGTATAAAATAGGATCTCCTCCTCCGATTGGATCAAAGAAAGATGTATTTAAGTTTCGGTCTGTTAATAACATAGTAATAGCTCCAGCTAAAACAGGAAGAGAAAGAAGTAATAAGATAGCTGTAATAACTACAGATCAAACAAATAAAGGTAGTCGATCTAAAGTAATTCCTGACGATCGTATATTAATTACAGTTGTAATAAAATTTACTGCCCCTAAAATTGAGGAAATTCCAGCTAAATGAAGAGAAAAAATAGCTAAATCAACAGAAGCTCCAGCATGAGCTGTTCCTGAAGAGAGAGGAGGATAAACTGTTCACCCAGTTCCTGCTCCATTTTCTACTATTGAGCTTGATAATAGAAGAGTCAATGAAGGAGGTAGTATTCAAAAACTTATATTATTTATTCGAGGAAAGGCTATATCAGGGGCTCCTAATATTAAAGGAACTAATCAAAAACAAAATTCCCTCCA

>LA_BH_13F5

GGGGGGTCAGATCAGATAAGTGTTGGTATAAAATAGGATCTCCTCCTCCGATTGGATCGAAGAAAGATGTATTTAAGTTTCGGTCTGTTAATAACATAGTAATAGCTCCAGCTAAAACAGGAAGAGAAAGAAGTAATAAGATAGCTGTAATAACTACAGATCAAACAAATAAAGGTAGTCGGTCTAAAGTAATTCCTGACGATCGTATATTAATTACAGTTGTAATAAAATTTACTGCCCCTAAAATTGAGGAAATTCCAGCTAAATGAAGAGAAAAAATAGCTAAATCAACAGAAGCTCCAGCATGAGCTGTTCCTGAAGAGAGAGGAGGATACACTGTTCACCCAGTTCCTGCCCCATTTTCTACTATTGAGCTTGATAATAGAAGAGTCAATGAAGGAGGTAGTATTCAAAAACTTATATTATTTATTCGAGGAAAGGCTATATCAGGGGCTCCTAATATTAAAGGAACTAATCAATTTCCAAAATCCCTCCCA

>LA_BHT_14F1

GTGGGGTCAAGATCAGATAAGTGTTGGTATAAAATAGGATCTCCTCCTCCGATTGGATCAAAGAAAGATGTATTTAAGTTTCGGTCTGTTAATAACATAGTAATAGCTCCAGCTAAAACAGGAAGAGAAAGAAGTAATAAGATAGCTGTAATAACTACAGATCAAACAAATAAAGGTAGTCGATCTAAAGTAATTCCTGACGATCGTATATTAATTACAGTTGTAATAAAATTTACTGCCCCTAAAATTGAGGAAATTCCAGCTAAATGAAGAGAAAAAATAGCTAAATCAACAGAAGCTCCAGCATGAGCTGTTCCTGAAGAGAGAGGAGGATAAACTGTTCACCCAGTTCCTGCTCCATTTTCTACTATTGAGCTTGATAATAGAAGAGTCAATGAAGGAGGTAGTATTCAAAAACTTATATTATTTATTCGAGGAAAGGCTATATCAGGGGCTCCTAATATTAAAGGAACTAATCAATTTCCAAATTCCTCCA

>LA_BHT_14F2

GATGCTTACGACATCACAATTAATCGTTGCATATCAAATAGGGTCTCCCCCTCCAATTGGATCAAAAAAAGATGTATTTAAATTTCGGTCTGTTAATAATATAGTAATAGCTCCGGCTAATACGGGTAGAGAAAGAAGTAATAAAATAGCTGTAATTACTACTGATCACACAAATAAAGGTAGTCGATCAAGAGTAATACCAGCTGATCGTATATTAATTACAGTTGTAATAAAATTTACTGCTCCTAAAATAGATGAGATTCCCGCTAAATGTAAAGAAAAAATTGCTAAATCAACTGAAGCCCCAGCATGAGCTGTTCCAGAAGAAAGGGGAGGATAAACCGTTCACCCTGTTCCAGCTCCGTTTTCTACTATAGAACTAGAAAGCAGCAGTGTTAAAGAGGGGGGTAATATTCAAAAACTTATATTATTTATTCGAGGAAAAGCTATATCAGGGGCTCCTAGTATTAAGGGAACTAATCAATTTCCAAAATTCCCCCCA

>LA_BHT_14F3

GTGGGGCAGTAATCAGAATAAGTGTTGGTATAAAATAGGATCTCCTCCTCCGATTGGATCAAAGAAAGATGTATTTAAGTTTCGGTCTGTTAATAACATAGTAATAGCTCCAGCTAAAACAGGAAGAGAAAGAAGTAATAAGATAGCTGTAATAACTACAGATCAAACAAATAAAGGTAGTCGATCTAAAGTAATTCCTGACGATCGTATATTAATTACAGTTGTAATAAAATTTACTGCCCCTAAAATTGAGGAAATTCCAGCTAAATGAAGAGAAAAAATAGCTAAATCAACAGAAGCTCCAGCATGAGCTGTTCCTGAAGAGAGAGGAGGATAAACTGTTCACCCAGTTCCTGCTCCATTTTCTACTATTGAGCTTGATAATAGAAGAGTCAATGAAGGAGGTAGTATTCAAAAACTTATATTATTTATTCGAGGAAAGGCTATATCAGGGGCTCCTAATATTAAAGGAACTAATCAATTTCAAATTCCCTCCCA

>LA_BHT_14F4

CTGGGGTCAGTATCACGATTAACGTAGTTCCGTCTAAAATAGGGTCTCCTCCTCCGATTGGATCAAAGAAAGATGTATTTAAGTTTCGGTCTGTTAATAACATAGTAATAGCTCCAGCTAAAACAGGAAGAGAAAGAAGTAATAAGATAGCTGTAATAACTACAGATCAAACAAATAAAGGTAGTCGRTCTAAAGTAATTCCTGACGATCGTATATTAATTACAGTTGTAATAAAATTTACTGCCCCTAAAATTGAGGAAATTCCAGCTAAATGAAGAGAAAAAATAGCTAAATCAACAGAAGCTCCAGCATGAGCTGTTCCTGAAGAGAGAGGAGGATAAACTGTTCACCCAGTTCCTGCCCCATTTTCTACTATTGAGCTTGATAATAGAAGAGTCAATGAAGGAGGTAGTATTCAAAAACTTATATTATTTATTCGAGGAAAGGCTATATCAGGGGCTCCTAATATTAAAGGAACTAATCAATTTCCAAAATTCCCCCCA

>LA_BHT_14F5

GTGGGGTCAAGATCAGAATAAGTGTTGGTATAAAATAGGATCTCCTCCTCCGATTGGATCAAAGAAAGATGTATTTAAGTTTCGGTCTGTTAATAACATAGTAATAGCTCCAGCTAAAACAGGAAGAGAAAGAAGTAATAAGATAGCTGTAATAACTACAGATCAAACAAATAAAGGTAGTCGATCTAAAGTAATTCCTGACGATCGTATATTAATTACAGTTGTAATAAAATTTACTGCCCCTAAAATTGAGGAAATTCCAGCTAAATGAAGAGAAAAAATAGCTAAATCAACAGAAGCTCCAGCATGAGCTGTTCCTGAAGAGAGAGGAGGATAAACTGTTCACCCAGTTCCTGCTCCATTTTCTACTATTGAGCTTGATAATAGAAGAGTCAATGAAGGAGGTAGTATTCAAAAACTTATATTATTTATTCGAGGAAAGGCTATATCAGGGGCTCCTAATATTAAAGGAACTAATCAATTTCCAAAATCCCCCCA

>LA_BHT_14F6

GTCCGTTAAGTAATGAGATTTACGTGTTCCGTATAAAATAGGATCTCCTCCTCCGATTGGATCGAAGAAAGATGTATTTAAGTTTCGGTCTGTTAATAACATAGTAATAGCTCCAGCTAAAACAGGAAGAGAAAGAAGTAATAAGATAGCTGTAATAACTACAGATCAAACAAATAAAGGTAGTCGGTCTAAAGTAATTCCTGACGATCGTATATTAATTACAGTTGTAATAAAATTTACTGCCCCTAAAATTGAGGAAATTCCAGCTAAATGAAGAGAAAAAATAGCTAAATCAACAGAAGCTCCAGCATGAGCTGTTCCTGAAGAGAGAGGAGGATACACTGTTCACCCAGTTCCTGCCCCATTTTCTACTATTGAGCTTGATAATAGAAGAGTCAATGAAGGAGGTAGTATTCAAAAACTTATATTATTTATTCGAGGAAAGGCTATATCAGGGGCTCCTAATATTAAAGGAACTAATCAATTTCAAAATTCCCCCCA

>LA_BHT_14F7

GTAGGGCCAGTATCAGATAAGTGTTGGTATAAAATAGGATCTCCTCCTCCGATTGGATCAAAGAAAGATGTATTTAAGTTTCGGTCTGTTAATAACATAGTAATAGCTCCGCTAAAACAGGAAGAGAAAGAAGTAATAAGATAGCTGTAATAACTACAGATCAAACAAATAAAGGTAGTCGATCTAAAGTAATTCCTGACGATCGTATATTAATTACAGTTGTAATAAAATTTACTGCCCCTAAAATTGAGGAAATTCCAGCTAAATGAAGAGAAAAAATAGCTAAATCAACAGAAGCTCCCGCATGAGCTGTTCCTGAAGAGAGAGGAGGATAAACTGTTCACCCAGTTCCTGCTCCATTTTCTACTATTGAGCTTGATAATAGAAGAGTCAATGAAGGAGGTAGTATTCAAAAACTTATATTATTTATTCGAGGAAAGGCTATATCAGGGGCTCCTAATATTAAAGGAACTAATCAATTTACAAATTCCCGCCCA

>LA_BHT_14F8

GTTGCTCCAGAATCAGAATAAGTGTTGGTATAAAATAGGATCTCCTCCTCCGATTGGATCAAAGAAAGATGTATTTAAGTTTCGGTCTGTTAATAACATAGTAATAGCTCCAGCTAAAACAGGAAGAGAAAGAAGTAATAAGATAGCTGTAATAACTACAGATCAAACAAATAAAGGTAGTCGATCTAAAGTAATTCCTGACGATCGTATATTAATTACAGTTGTAATAAAATTTACTGCCCCTAAAATTGAGGAAATTCCAGCTAAATGAAGAGAAAAAATAGCTAAATCAACAGAAGCTCCAGCATGAGCTGTTCCTGAAGAGAGAGGAGGATAAACTGTTCACCCAGTTCCTGCCCCATTTTCTACTATTGAGCTCGATAATAGAAGAGTCAATGAAGGAGGTAGTATTCAAAAACTTATATTATTTATTCGAGGAAAGGCTATATCAGGGGCTCCTAATATTAAAGGAACTAATCAATTTCCAAAATTCCCCCCA

>LA_BHT_14F9

GGAGGTCAAGATCAGAATAAGTGTTGGTATAAAATAGGATCTCCTCCTCCGATTGGATCAAAGAAAGATGTATTTAAGTTTCGGTCTGTTAATAATATAGTAATAGCTCCAGCTAAAACAGGAAGAGAAAGAAGTAATAAGATAGCTGTAATAACTACAGATCAAACAAATAAAGGTAGTCGATCTAAAGTAATTCCTGACGATCGCATATTAATTACAGTTGTAATAAAATTTACTGCCCCTAAAATTGAGGAAATTCCAGCTAAATGAAGAGAAAAAATAGCTAAATCAACAGAAGCTCCAGCATGAGCTGTTCCTGAAGAGAGAGGAGGATAAACTGTTCACCCAGTTCCTGCCCCATTTTCTACTATTGAGCTTGATAATAGAAGAGTCAATGAAGGAGGTAGTATTCAAAAACTTATATTATTTATTCGAGGAAAGGCTATATCAGGGGCTCCTAATATTAAAGGAACTAATCAATTTCCAAAATTCCCCCCA

>LA_BHT_15F1

GGGGGGTTCAGATCAGAATAAGTGTTGGTATAAAATAGGATCTCCTCCTCCGATTGGATCGAAGAAAGATGTATTTAAGTTTCGGTCTGTTAATAACATAGTAATAGCTCCAGCTAAAACAGGAAGAGAAAGAAGTAATAAGATAGCTGTAATAACTACAGATCAAACAAATAAAGGTAGTCGGTCTAAAGTAATTCCTGACGATCGTATATTAATTACAGTTGTAATAAAATTTACTGCCCCTAAAATTGAGGAAATTCCAGCTAAATGAAGAGAAAAAATAGCTAAATCAACAGAAGCTCCAGCATGAGCTGTTCCTGAAGAGAGAGGAGGATACACTGTTCACCCAGTTCCTGCCCCATTTTCTACTATTGAGCTTGATAATAGAAGAGTCAATGAAGGAGGTAGTATTCAAAAACTTATATTATTTATTCGAGGAAAGGCTATATCAGGGGCTCCTAATATTAAAGGAACTAATCAATTTCAAATTCCCCCCA

>LA_BHT_15F2

GCTGCGGTTCAGTATCAGATTTAGTGTTCCGTATAAAATAGGATCTCCTCCTCCGATTGGATCGAAGAAAGATGTATTTAAGTTTCGGTCTGTTAATAACATAGTAATAGCTCCAGCTAAAACAGGAAGAGAAAGAAGTAATAAGATAGCTGTAATAACTACAGATCAAACAAATAAAGGTAGTCGGTCTAAAGTAATTCCTGACGATCGTATATTAATTACAGTTGTAATAAAATTTACTGCCCCTAAAATTGAGGAAATTCCAGCTAAATGAAGAGAAAAAATAGCTAAATCAACAGAAGCTCCAGCATGAGCTGTTCCTGAAGAGAGAGGAGGATACACTGTTCACCCAGTTCCTGCCCCATTTTCTACTATTGAGCTTGATAATAGAAGAGTCAATGAAGGAGGTAGTATTCAAAAACTTATATTATTTATTCGAGGAAAGGCTATATCAGGGGCTCCTAATATTAAAGGAACTAATCAATTTCAAAATTCCCCCCA

>LA_BHT_15F3

GTGGGTGCAAGTATCAGAATAAGTGTTGGTATAAAATAGGATCTCCTCCTCCGATTGGATCAAAGAAAGATGTATTTAAGTTTCGGTCTGTTAATAATATAGTAATAGCTCCAGCTAAAACAGGAAGAGAAAGAAGTGATAAGATAGCTGTAATAACTACAGATCAAACAAATAAAGGTAGTCGATCTAAAGTAATTCCTGACGATCGCATATTAATTACAGTTGTAATAAAATTTACTGCCCCTAAAATTGAGGAAATTCCAGCTAAATGAAGAGAAAAAATAGCTAAATCAACAGAAGCTCCAGCATGAGCTGTTCCTGAAGAGAGAGGAGGATAAACTGTTCACCCAGTTCCTGCCCCATTTTCTACTATTGAGCTTGATAATAGAAGAGTCAATGAAGGAGGTAGTATTCAAAAACTTATATTATTTATTCGAGGAAAGGCTATATCAGGGGCTCCTAATATTAAAGGAACTAATCAATTTCAAAATTCCCCCCA

>LA_BHT_15F4

GTGGGGGTCAGATCAGAATAAGTGTTGGTATAAAATAGGATCTCCTCCTCCGATTGGATCGAAGAAAGATGTATTTAAGTTTCGGTCTGTTAATAACATAGTAATAGCTCCAGCTAAAACAGGAAGAGAAAGAAGTAATAAGATAGCTGTAATAACTACAGATCAAACAAATAAAGGTAGTCGGTCTAAAGTAATTCCTGACGATCGTATATTAATTACAGTTGTAATAAAATTTACTGCCCCTAAAATTGAGGAAATTCCAGCTAAATGAAGAGAAAAAATAGCTAAATCAACAGAAGCTCCAGCATGAGCTGTTCCTGAAGAGAGAGGAGGATACACTGTTCACCCAGTTCCTGCCCCATTTTCTACTATTGAGCTTGATAATAGAAGAGTCAATGAAGGAGGTAGTATTCAAAAACTTATATTATTTATTCGAGGAAAGGCTATATCAGGGGCTCCTAATATTAAAGGAACTAATCAATTTCCAAAATTCCCTCCCA

>LA_BHT_15F5

AGATCAGAATAAGTGTTGGTATAAAATAGGATCTCCTCCTCCGATTGGATCAAAGAAAGATGTATTTAAGTTTCGGTCTGTTAATAACATAGTAATAGCTCCAGCTAAAACAGGAAGAGAAAGAAGTAATAAGATAGCTGTAATAACTACAGATCAAACAAATAAAGGTAGTCGATCTAAAGTAATTCCTGACGATCGTATATTAATTACAGTTGTAATAAAATTTACTGCCCCTAAAATTGAGGAAATTCCAGCTAAATGAAGAGAAAAAATAGCTAAATCAACAGAAGCTCCAGCATGAGCTGTTCCTGAAGAGAGAGGAGGATAAACTGTTCACCCAGTTCCTGCCCCATTTTCTACTATTGAGCTTGATAATAGAAGAGTCAATGAAGGAGGTAGTATTCAAAAACTTATATTATTTATTCGAGGAAAGGCTATATCAGGGGCTCCTAATATTAAAGGAACTAATCAATTTCCAAATCCTCCAA

>LA_BHT_15F6

AGATCAGAATAAGTGTTGGTATAAAATAGGATCTCCTCCTCCGATTGGATCAAAGAAAGATGTATTTAAGTTTCGGTCTGTTAATAACATAGTAATAGCTCCAGCTAAAACAGGAAGAGAAAGAAGTAATAAGATAGCTGTAATAACTACAGATCAAACAAATAAAGGTAGTCGATCTAAAGTAATTCCTGACGATCGTATATTAATTACAGTTGTAATAAAATTTACTGCCCCTAAAATTGAGGAAATTCCAGCTAAATGAAGAGAAAAAATAGCTAAATCAACAGAAGCTCCAGCATGAGCTGTTCCTGAAGAGAGAGGAGGATAAACTGTTCACCCAGTTCCTGCTCCATTTTCTACTATTGAGCTTGATAATAGAAGAGTCAATGAAGGAGGTAGTATTCAAAAACTTATATTATTTATTCGAGGAAAGGCTATATCAGGGGCTCCTAATATTAAAGGAACTAATCAATTTCCAAATTCCTCCA

>LA_BHT_15F7

AGATCAGAATAAGTGTTGGTATAAAATAGGATCTCCTCCTCCGATTGGATCAAAGAAAGATGTATTTAAGTTTCGGTCTGTTAATAACATAGTAATAGCTCCAGCTAAAACAGGAAGAGAAAGAAGTAATAAGATAGCTGTAATAACTACAGATCAAACAAATAAAGGTAGTCGATCTAAAGTAATTCCTGACGATCGTATATTAATTACAGTTGTAATAAAATTTACTGCCCCTAAAATTGAGGAAATTCCAGCTAAATGAAGAGAAAAAATAGCTAAATCAACAGAAGCTCCAGCATGAGCTGTTCCTGAAGAGAGAGGAGGATAAACTGTTCACCCAGTTCCTGCCCCATTTTCTACTATTGAGCTTGATAATAGAAGAGTCAATGAAGGAGGTAGTATTCAAAAACTTATATTATTTATTCGAGGAAAGGCTATATCAGGGGCTCCTAATATTAAAGGAACTAATCAATTCCAAATTCCCTCCCA

>LA_BHT_15F8

AGATCAGAATAAGTGTTGGTATAAAATAGGATCTCCTCCTCCGATTGGATCAAAGAAAGATGTATTTAAGTTTCGGTCTGTTAATAACATAGTAATAGCTCCAGCTAAAACAGGAAGAGAAAGAAGTAATAAGATAGCTGTAATAACTACAGATCAAACAAATAAAGGTAGTCGATCTAAAGTAATTCCTGACGATCGTATATTAATTACAGTTGTAATAAAATTTACTGCCCCTAAAATTGAGGAAATTCCAGCTAAATGAAGAGAAAAAATAGCTAAATCAACAGAAGCTCCAGCATGAGCTGTTCCTGAAGAGAGAGGAGGATAAACTGTTCACCCAGTTCCTGCTCCATTTTCTACTATTGAGCTTGATAATAGAAGAGTCAATGAAGGAGGTAGTATTCAAAAACTTATATTATTTATTCGAGGAAAGGCTATATCAGGGGCTCCTAATATTAAAGGAACTAATCAATTCCAAATTCCCTCCA

>LA_BHT_15F9

AGATCAGAATAAGTGTTGGTATAAAATAGGATCTCCTCCTCCGATTGGATCAAAGAAAGATGTATTTAAGTTTCGGTCTGTTAATAACATAGTAATAGCTCCAGCTAAAACAGGAAGAGAAAGAAGTAATAAGATAGCTGTAATAACTACAGATCAAACAAATAAAGGTAGTCGATCTAAAGTAATTCCTGACGATCGTATATTAATTACAGTTGTAATAAAATTTACTGCCCCTAAAATTGAGGAAATTCCAGCTAAATGAAGAGAAAAAATAGCTAAATCAACAGAAGCTCCAGCATGAGCTGTTCCTGAAGAGAGAGGAGGATAAACTGTTCACCCAGTTCCTGCTCCATTTTCTACTATTGAGCTTGATAATAGAAGAGTCAATGAAGGAGGTAGTATTCAAAAACTTATATTATTTATTCGAGGAAAGGCTATATCAGGGGCTCCTAATATTAAAGGAACTAATCAATTTCCAAATTCCTCCA

>LA_BHT_15F10

CAGAATCAGATAAGTGTTGGTATAAAATAGGATCTCCTCCTCCGATTGGATCAAAGAAAGATGTATTTAAGTTTCGGTCTGTTAATAACATAGTAATAGCTCCAGCTAAAACAGGAAGAGAAAGAAGTAATAAGATAGCTGTAATAACTACAGATCAAACAAATAAAGGTAGTCGATCTAAAGTAATTCCTGACGATCGTATATTAATTACAGTTGTAATAAAATTTACTGCCCCTAAAATTGAGGAAATTCCAGCTAAATGAAGAGAAAAAATAGCTAAATCAACAGAAGCTCCAGCATGAGCTGTTCCTGAAGAGAGAGGAGGATAAACTGTTCACCCAGTTCCTGCTCCATTTTCTACTATTGAGCTTGATAATAGAAGAGTCAATGAAGGAGGTAGTATTCAAAAACTTATATTATTTATTCGAGGAAAGGCTATATCAGGGGCTCCTAATATTAAAGGAACTAATCAATTCCAAATTTCCTTCCA

>LA_BHT_16F1

GATCAGAATAAGTGTTGGTATAAAATAGGATCTCCTCCTCCGATTGGATCAAAGAAAGATGTATTTAAGTTTCGGTCTGTTAATAACATAGTAATAGCTCCAGCTAAAACAGGAAGAGAAAGAAGTAATAAGATAGCTGTAATAACTACAGATCAAACAAATAAAGGTAGTCGATCTAAAGTAATTCCTGACGATCGTATATTAATTACAGTTGTAATAAAATTTACTGCCCCTAAAATTGAGGAAATTCCAGCTAAATGAAGAGAAAAAATAGCTAAATCAACAGAAGCTCCAGCATGAGCTGTTCCTGAAGAGAGAGGAGGATAAACTGTTCACCCAGTTCCTGCTCCATTTTCTACTATTGAGCTTGATAATAGAAGAGTCAATGAAGGAGGTAGTATTCAAAAACTTATATTATTTATTCGAGGAAAGGCTATATCAGGGGCTCCTAATATTAAAGGAACTAATCAATTTCCAAATTTCCTCCA

>LA_BHT_16F2

GGTCGTTAGGTATAAAATAGGATCTCCTCCTCCGATTGGATCAAAGAAAGATGTATTTAAGTTTCGGTCTGTTAATAACATAGTAATAGCTCCAGCTAAAACAGGAAGAGAAAGAAGTAATAAGATAGCTGTAATAACTACAGATCAAACAAATAAAGGTAGTCGATCTAAAGTAATTCCTGACGATCGTATATTAATTACAGTTGTAATAAAATTTACTGCCCCTAAAATTGAGGAAATTCCAGCTAAATGAAGAGAAAAAATAGCTAAATCAACAGAAGCTCCAGCATGAGCTGTTCCTGAAGAGAGAGGAGGATAAACTGTTCACCCAGTTCCTGCTCCATTTTCTACTATTGAGCTTGATAATAGAAGAGTCAATGAAGGAGGTAGTATTCAAAAACTTATATTATTTATTCGAGGAAAGGCTATATCAGGGGCTCCTAATATTAAAGGAACTAATCAATTCCAAATTTCCTCCA

>LA_BHT_16F3

GATCAGATAAGTGTTGGTATAAAATAGGATCTCCTCCTCCGATTGGATCAAAGAAAGATGTATTTAAGTTTCGGTCTGTTAATAACATAGTAATAGCTCCAGCTAAAACAGGAAGAGAAAGAAGTAATAAGATAGCTGTAATAACTACAGATCAAACAAATAAAGGTAGTCGATCTAAAGTAATTCCTGACGATCGTATATTAATTACAGTTGTAATAAAATTTACTGCCCCTAAAATTGAGGAAATTCCAGCTAAATGAAGAGAAAAAATAGCTAAATCAACAGAAGCTCCAGCATGAGCTGTTCCTGAAGAGAGAGGAGGATAAACTGTTCACCCAGTTCCTGCTCCATTTTCTACTATTGAGCTTGATAATAGAAGAGTCAATGAAGGAGGTAGTATTCAAAAACTTATATTATTTATTCGAGGAAAGGCTATATCAGGGGCTCCTAATATTAAAGGAACTAATCAATTCCAAAATTCCCTCCCA

>LA_BHT_16F4

GATCAGAATAAGTGTTGGTATAAAATAGGATCTCCTCCTCCGATTGGATCAAAGAAAGATGTATTTAAGTTTCGGTCTGTTAATAACATAGTAATAGCTCCAGCTAAAACAGGAAGAGAAAGAAGTAATAAGATAGCTGTAATAACTACAGATCAAACAAATAAAGGTAGTCGATCTAAAGTAATTCCTGACGATCGTATATTAATTACAGTTGTAATAAAATTTACTGCCCCTAAAATTGAGGAAATTCCAGCTAAATGAAGAGAAAAAATAGCTAAATCAACAGAAGCTCCAGCATGAGCTGTTCCTGAAGAGAGAGGAGGATAAACTGTTCACCCAGTTCCTGCCCCATTTTCTACTATTGAGCTTGATAATAGAAGAGTCAATGAAGGAGGTAGTATTCAAAAACTTATATTATTTATTCGAGGAAAGGCTATATCAGGGGCTCCTAATATTAAAGGAACTAATCAATTCCAAAATTTCCTCCA

>LA_BHT_16F5

AGAATCAGAATAAGTGTTGGTAT

AAAATAGGATCTCCT

CCTCCGATTGGATCAAAGAAAGATGTATTTAAGTTTCGGTCTGTTAATAACATAGTAATAGCTCCAGCTAAAACAGGAAGAGAAAGAAGTAATAAGATAGCTGTAATAACTACAGATCAAACAAATAAAGGTAGTCGATCTAAAGTAATTCCTGACGATCGTATATTAATTACAGTTGTAATAAAATTTACTGCCCCTAAAATTGAGGAAATTCCAGCTAAATGAAGAGAAAAAATAGCTAAATCAACAGAAGCTCCAGCATGAGCTGTTCCTGAAGAGAGAGGAGGATAAACTGTTCACCCAGTTCCTGCTCCATTTTCTACTATTGAGCTTGATAATAGAAGAGTCAATGAAGGAGGTAGTATTCAAAAACTTATATTATTTATTCGAGGAAAGGCTATATCAGGGGCTCCTAATATTAAAGGAACTAATCAATTCCAAATTCCTCCA

>LA_BHT_16F6

AGATCAGATAAGTGTTGGTATAAAATAGGATCTCCTCCTCCGATTGGATCAAAGAAAGATGTATTTAAGTTTCGGTCTGTTAATAACATAGTAATAGCTCCAGCTAAAACAGGAAGAGAAAGAAGTAATAAGATAGCTGTAATAACTACAGATCAAACAAATAAAGGTAGTCGATCTAAAGTAATTCCTGACGATCGTATATTAATTACAGTTGTAATAAAATTTACTGCCCCTAAAATTGAGGAAATTCCAGCTAAATGAAGAGAAAAAATAGCTAAATCAACAGAAGCTCCAGCATGAGCTGTTCCTGAAGAGAGAGGAGGATAAACTGTTCACCCAGTTCCTGCTCCATTTTCTACTATTGAGCTTGATAATAGAAGAGTCAATGAAGGAGGTAGTATTCAAAAACTTATATTATTTATTCGAGGAAAGGCTATATCAGGGGCTCCTAATATTAAAGGAACTAATCAATTCCAAATCTCCTCCA

>LA_BHT_16F7

AGATAGAATAAGTGTTGGTATAAAATAGGATCTCCTCCTCCGATTGGATCAAAGAAAGATGTATTTAAGTTTCGGTCTGTTAATAACATAGTAATAGCTCCAGCTAAAACAGGAAGAGAAAGAAGTAATAAGATAGCTGTAATAACTACAGATCAAACAAATAAAGGTAGTCGATCTAAAGTAATTCCTGATGATCGTATATTAATTACAGTTGTAATAAAATTTACTGCCCCTAAAATTGAGGAAATTCCAGCTAAATGAAGAGAAAAAATAGCTAAATCAACAGAAGCTCCAGCATGAGCTGTTCCTGAAGAGAGAGGAGGATAAACTGTTCACCCAGTTCCTGCTCCATTTTCTACTATTGAGCTTGATAATAGAAGAGTCAATGAAGGAGGTAGTATTCAAAAACTTATATTATTTATTCGAGGAAAGGCTATATCAGGGGCTCCTAATATTAAAGGAACTAATCAATTTCCAAATTCCTCCA

>LA_BHT_16F8

GGTGGTTGGTAT

AAAATAGGGTCTCCCCCTCCGATTGGATCAAAGAAAGATGTATTTAAGTTTCGGTCTGTTAATAATATAGTAATAGCTCCAGCTAAAACAGGAAGAGAAAGAAGTAATAAGATAGCTGTAATAACTACAGATCAAACAAATAAGGGTAGTCGATCTAAAGTAATCCCTGACGATCGCATATTAATCACAGTTGTAATAAAATTTACTGCCCCTAAAATTGAGGAAATTCCAGCTAAATGAAGAGAAAAAATAGCTAAATCAACAGAAGCTCCAGCATGAGCTGTTCCTGAAGAGAGAGGAGGATAAACTGTTCACCCAGTTCCTGCCCCATTTTCTACTATTGAGCTTGATAATAGAAGAGTCAATGAAGGAGGTAGTATTCAAAAACTTATATTATTCATTCGAGGGAAAGCTATATCAGGGGCTCCTAATATTAAAGGAACTAATCAATTTCCAAATTCCTCCA

>LA_BHT_16F9

AGAATCAGAATAAGTGTTGGTATAAAATAGGATCTCCTCCTCCGATTGGATCAAAGAAAGATGTATTTAAGTTTCGGTCTGTTAATAACATAGTAATAGCTCCAGCTAAAACAGGAAGAGAAAGAAGTAATAAGATAGCTGTAATAACTACAGATCAAACAAATAAAGGTAGTCGATCTAAAGTAATTCCTGACGATCGTATATTAATTACAGTTGTAATAAAATTTACTGCCCCTAAAATTGAGGAAATTCCAGCTAAATGAAGAGAAAAAATAGCTAAATCAACAGAAGCTCCAGCATGAGCTGTTCCTGAAGAGAGAGGAGGATAAACTGTTCACCCAGTTCCTGCTCCATTTTCTACTATTGAGCTTGATAATAGAAGAGTCAATGAAGGAGGTAGTATTCAAAAACTTATATTATTTATTCGAGGAAAGGCTATATCAGGGGCTCCTAATATTAAAGGAACTAATCAATTTCCAAATTCCTCCA

>LA_BHT_16F10

GATCAGAATAAGTGTTGGTATAAAATAGGATCTCCTCCTCCGATTGGATCAAAGAAAGATGTATTTAAGTTTCGGTCTGTTAATAACATAGTAATAGCTCCAGCTAAAACAGGAAGAGAAAGAAGTAATAAGATAGCTGTAATAACTACAGATCAAACAAATAAAGGTAGTCGATCTAAAGTAATTCCTGACGATCGTATATTAATTACAGTTGTAATAAAATTTACTGCCCCTAAAATTGAGGAAATTCCAGCTAAATGAAGAGAAAAAATAGCTAAATCAACAGAAGCTCCAGCATGAGCTGTTCCTGAAGAGAGAGGAGGATAAACTGTTCACCCAGTTCCTGCTCCATTTTCTACTATTGAGCTTGATAATAGAAGAGTCAATGAAGGAGGTAGTATTCAAAAACTTATATTATTTATTCGAGGAAAGGCTATATCAGGGGCTCCTAATATTAAAGGAACTAATCAATTTCCAAATTCCTCCCA

>LA_BHT_17F1

GAATCAGAATAAGTGTTGGTATAAAATAGGATCTCCTCCTCCGATTGGATCAAAGAAAGATGTATTTAAGTTTCGGTCTGTTAATAACATAGTAATAGCTCCAGCTAAAACAGGAAGAGAAAGAAGTAATAAGATAGCTGTAATAACTACAGATCAAACAAATAAAGGTAGTCGATCTAAAGTAATTCCTGACGATCGTATATTAATTACAGTTGTAATAAAATTTACTGCCCCTAAAATTGAGGAAATTCCAGCTAAATGAAGAGAAAAAATAGCTAAATCAACAGAAGCTCCAGCATGAGCTGTTCCTGAAGAGAGAGGAGGATAAACTGTTCACCCAGTTCCTGCCCCATTTTCTACTATTGAGCTTGATAATAGAAGAGTCAATGAAGGAGGTAGTATTCAAAAACTTATATTATTTATTCGAGGAAAGGCTATATCAGGGGCTCCTAATATTAAAGGAACTAATCAATTTCCAAATTCCTCCA

>LA_BHT_17F2

AGATCAGAATAAGTGTTGGTATAAAATAGGATCTCCTCCTCCGATTGGATCAAAGAAAGATGTATTTAAGTTTCGGTCTGTTAATAACATAGTAATAGCTCCAGCTAAAACAGGAAGAGAAAGAAGTAATAAGATAGCTGTAATAACTACAGATCAAACAAATAAAGGTAGTCGATCTAAAGTAATTCCTGACGATCGTATATTAATTACAGTTGTAATAAAATTTACTGCCCCTAAAATTGAGGAAATTCCAGCTAAATGAAGAGAAAAAATAGCTAAATCAACAGAAGCTCCAGCATGAGCTGTTCCTGAAGAGAGAGGAGGATAAACTGTTCACCCAGTTCCTGCCCCATTTTCTACTATTGAGCTTGATAATAGAAGAGTCAATGAAGGAGGTAGTATTCAAAAACTTATATTATTTATTCGAGGAAAGGCTATATCAGGGGCTCCTAATATTAAAGGAACTAATCAATTCCAAATTCCTCCA

>LA_BHT_17F3

GATCAGAATAAGTGTTGGTATAAAATAGGATCTCCTCCTCCGATTGGATCAAAGAAAGATGTATTTAAGTTTCGGTCTGTTAATAACATAGTAATAGCTCCAGCTAAAACAGGAAGAGAAAGAAGTAATAAGATAGCTGTAATAACTACAGATCAAACAAATAAAGGTAGTCGATCTAAAGTAATTCCTGACGATCGTATATTAATTACAGTTGTAATAAAATTTACTGCCCCTAAAATTGAGGAAATTCCAGCTAAATGAAGAGAAAAAATAGCTAAATCAACAGAAGCTCCAGCATGAGCTGTTCCTGAAGAGAGAGGAGGATAAACTGTTCACCCAGTTCCTGCTCCATTTTCTACTATTGAGCTTGATAATAGAAGAGTCAATGAAGGAGGTAGTATTCAAAAACTTATATTATTTATTCGAGGAAAGGCTATATCAGGGGCTCCTAATATTAAAGGAACTAATCAATTTCCAAATTTCCTCCA

>LA_BHT_17F4

ATCGTTGATAC

CAAATAGGGTCTCCCCCTCCAATTGGATCAAAAAAAGATGTATTTAAATTTCGGTCTGTTAATAATATAGTAATAGCTCCGGCTAATACGGGTAGAGAAAGAAGTAATAAAATAGCTGTAATTACTACTGATCACACAAATAAAGGTAGTCGATCAAGAGTAATACCAGMTGATCGTATATTAATTACAGTTGTAATAAAATTTACTGCTCCTAAAATAGATGAGATTCCCGCTAAATGTAAAGAAAAAATTGCTAAATCAACTGAAGCCCCAGCATGAGCTGTTCCAGAAGAAAGGGGAGGATAAACCGTTCACCCTGTTCCAGCTCCRTTTTCTACTATAGAACTAGAAAGCAGCAGAGTTAAAGAGGGAGGTAATATTCAAAAACTTATATTATTTATTCGAGGAAAAGCTATATCAGGGGCTCCTAGTATTAAGGGAACTAATCAATTCCAAATTTCCTCCA

>LA_BHT_17F5

GATCAGATAAGTGTTGGTATAAAATAGGATCTCCTCCTCCGATTGGATCAAAGAAAGATGTATTTAAGTTTCGGTCTGTTAATAACATAGTAATAGCTCCAGCTAAAACAGGAAGAGAAAGAAGTAATAAGATAGCTGTAATAACTACAGATCAAACAAATAAAGGTAGTCGATCTAAAGTAATTCCTGATGATCGTATATTAATTACAGTTGTAATAAAATTTACTGCCCCTAAAATTGAGGAAATTCCAGCTAAATGAAGAGAAAAAATAGCTAAATCAACAGAAGCTCCAGCATGAGCTGTTCCTGAAGAGAGAGGAGGATAAACTGTTCACCCAGTTCCTGCTCCATTTTCTACTATTGAGCTTGATAATAGAAGAGTCAATGAAGGAGGTAGTATTCAAAAACTTATATTATTTATTCGAGGAAAGGCTATATCAGGGGCTCCTAATATTAAAGGAACTAATCAATTCCAAATTCCCTCCA

>LA_BHT_17F6

ATTTTCGTTTGATACCAAATAGGGTCTCCCCCTCCAATTGGATCAAAAAAAGATGTATTTAAATTTCGGTCTGTTAATAATATAGTAATAGCTCCGGCTAATACGGGTAGAGAAAGAAGTAATAAAATAGCTGTAATTACTACTGATCACACAAATAAAGGTAGTCGATCAAGAGTAATACCAGCTGATCGTATATTAATTACAGTTGTAATAAAATTTACTGCTCCTAAAATAGATGAGATTCCCGCTAAATGTAAAGAAAAAATTGCTAAATCAACTGAAGCCCCAGCATGAGCTGTTCCAGAAGAAAGGGGAGGATAAACCGTTCACCCTGTTCCAGCTCCGTTTTCTACTATAGAACTAGAAAGCAGCAGTGTTAAAGAGGGAGGTAATATTCAAAAACTTATATTATTTATTCGAGGAAAAGCTATATCAGGGGCTCCTAGTATTAAGGGAACTAATCAATTCCAAATTCCCTCCA

>LA_BHT_17F7

ATCAGAATAAGTGTTGGTATAAAATAGGATCTCCTCCTCCGATTGGATCAAAGAAAGATGTATTTAAGTTTCGGTCTGTTAATAACATAGTAATAGCTCCAGCTAAAACAGGAAGAGAAAGAAGTAATAAGATAGCTGTAATAACTACAGATCAAACAAATAAAGGTAGTCGATCTAAAGTAATTCCTGACGATCGTATATTAATTACAGTTGTAATAAAATTTACTGCCCCTAAAATTGAGGAAATTCCAGCTAAATGAAGAGAAAAAATAGCTAAATCAACAGAAGCTCCAGCATGAGCTGTTCCTGAAGAGAGAGGAGGATAAACTGTTCACCCAGTTCCTGCCCCATTTTCTACTATTGAGCTTGATAATAGAAGAGTCAATGAAGGAGGTAGTATTCAAAAACTTATATTATTTATTCGAGGAAAGGCTATATCAGGGGCTCCTAATATTAAAGGAACTAATCAATTCCAAAATTTCCCTCCA

>LA_BHT_17F8

GATAGAATAAGTGTTGGTATAAAATAGGATCTCCTCCTCCGATTGGATCAAAGAAAGATGTATTTAAGTTTCGGTCTGTTAATAACATAGTAATAGCTCCAGCTAAAACAGGAAGAGAAAGAAGTAATAAGATAGCTGTAATAACTACAGATCAAACAAATAAAGGTAGTCGATCTAAAGTAATTCCTGACGATCGTATATTAATTACAGTTGTAATAAAATTTACTGCCCCTAAAATTGAGGAAATTCCAGCTAAATGAAGAGAAAAAATAGCTAAATCAACAGAAGCTCCAGCATGAGCTGTTCCTGAAGAGAGAGGAGGATAAACTGTTCACCCAGTTCCTGCTCCATTTTCTACTATTGAGCTTGATAATAGAAGAGTCAATGAAGGAGGTAGTATTCAAAAACTTATATTATTTATTCGAGGAAAGGCTATATCAGGGGCTCCTAATATTAAAGGAACTAATCAATTTCCAAATTCCTCCA

>LA_BHT_17F9

ATCAGAATAAGTGTTGGTATAAAATAGGATCTCCTCCTCCGATTGGATCAAAGAAAGATGTATTTAAGTTTCGGTCTGTTAATAACATAGTAATAGCTCCAGCTAAAACAGGAAGAGAAAGAAGTAATAAGATAGCTGTAATAACTACAGATCAAACAAATAAAGGTAGTCGATCTAAAGTAATTCCTGACGATCGTATATTAATTACAGTTGTAATAAAATTTACTGCCCCTAAAATTGAGGAAATTCCAGCTAAATGAAGAGAAAAAATAGCTAAATCAACAGAAGCTCCAGCATGAGCTGTTCCTGAAGAGAGAGGAGGATAAACTGTTCACCCAGTTCCTGCCCCATTTTCTACTATTGAGCTTGATAATAGAAGAGTCAATGAAGGAGGTAGTATTCAAAAACTTATATTATTTATTCGAGGAAAGGCTATATCAGGGGCTCCTAATATTAAAGGAACTAATCAATTCCAAATTCCCTCCA

>LA_BHT_17F10

AGATCAGAATAAGTGTTGGTATAAAATAGGATCTCCTCCTCCGATTGGATCAAAGAAAGATGTATTTAAGTTTCGGTCTGTTAATAACATAGTAATAGCTCCAGCTAAAACAGGAAGAGAAAGAAGTAATAAGATAGCTGTAATAACTACAGATCAAACAAATAAAGGTAGTCGGTCTAAAGTAATTCCTGACGATCGTATATTAATTACAGTTGTAATAAAATTTACTGCCCCTAAAATTGAGGAAATTCCAGCTAAATGAAGAGAAAAAATAGCTAAATCAACAGAAGCTCCAGCATGAGCTGTTCCTGAAGAGAGAGGAGGATAAACTGTTCACCCAGTTCCTGCCCCATTTTCTACTATTGAGCTTGATAATAGAAGAGTCAATGAAGGAGGTAGTATTCAAAAACTTATATTATTTATTCGAGGAAAGGCTATATCAGGGGCTCCTAATATTAAAGGAACTAATCAATTCCAAAATTCCCTCCCA

>LA_BHT_18F1

ATCCAAATAGGGTCTCCCCCTCCCATTCCGATCAAAAAAAGATGTATTTWAATTTMGGTCTGTTAATAATATAGTAATAGCTCCGGCTAATACGGGTAGAGAAAGAAGTAATAAAATAGCTGTAATTACTACTGATCACACAAATAAAGGTAGTCGATCAAGAGTAATACCAGCTGATCGTATATTAATTACAGTTGTAATAAAATTTACTGCTCCTAAAATAGATGAGATTCCCGCTAAATGTAAAGAAAAAATTGCTAAATCAACTGAAGCCCCAGCATGAGCTGTTCCAGAAGAAAGGGGAGGATAAACCGTTCACCCTGTTCCAGCTCCGTTTTCTACTATAGAACTAGAAAGCAGCAGTGTTAAAGAGGGAGGTAATATTCAAAAACTTATATTATTTATTCGAGGAAAAGCTATATCAGGGGCTCCTAGTATTAAGGGAACTAATCAATTTCCAAAATTCCTTCCA

>LA_BHT_18F2

GATAAGTGTTGGTATAAAATAGGATCTCCTCCTCCGATTGGATCAAAGAAAGATGTATTTAAGTTTCGGTCTGTTAATAACATAGTAATAGCTCCAGCTAAAACAGGAAGAGAAAGAAGTAATAAGATAGCTGTAATAACTACAGATCAAACAAATAAAGGTAGTCGATCTAAAGTAATTCCTGACGATCGTATATTAATTACAGTTGTAATAAAATTTACTGCCCCTAAAATTGAGGAAATTCCAGCTAAATGAAGAGAAAAAATAGCTAAATCAACAGAAGCTCCAGCATGAGCTGTTCCTGAAGAGAGAGGAGGATAAACTGTTCACCCAGTTCCTGCTCCATTTTCTACTATTGAGCTTGATAATAGAAGAGTCAATGAAGGAGGTAGTATTCAAAAACTTATATTATTTATTCGAGGAAAGGCTATATCAGGGGCTCCTAATATTAAAGGAACTAATCAATTTCCAAATTTCCTCCA

>LA_BHT_19M1

GATAAGTGTTGGTATAAAATAGGATCTCCTCCTCCGATTGGATCAAAGAAAGATGTATTTAAGTTTCGGTCTGTTAATAACATAGTAATAGCTCCGGCTAAAACAGGAAGAGAAAGAAGTAATAAGATAGCTGTAATAACTACAGATCAAACAAATAAAGGTAGTCGATCTAAAGTAATTCCTGACGATCGTATATTAATTACAGTTGTAATAAAATTTACTGCCCCTAAAATTGAGGAAATTCCAGCTAAATGAAGAGAAAAAATAGCTAAATCAACAGAAGCTCCAGCATGAGCTGTTCCTGAAGAGAGAGGAGGATAAACTGTTCACCCAGTTCCTGCTCCATTTTCTACTATTGAGCTTGATAATAGAAGAGTCAATGAAGGAGGTAGTATTCAAAAACTTATATTATTTATTCGAGGAAAGGCTATATCAGGGGCTCCTAATATTAAAGGAACTAATCAATTTCCAAAATTCCTCCA

>LA_BHT_19M2

GAATAAGTGTTGGTATAAAATAGGATCTCCTCCTCCGATTGGATCAAAGAAAGATGTATTTAAGTTTCGGTCTGTTAATAACATAGTAATAGCTCCAGCTAAAACAGGAAGAGAAAGAAGTAATAAGATAGCTGTAATAACTACAGATCAAACAAATAAAGGTAGTCGATCTAAAGTAATTCCTGACGATCGTATATTAATTACAGTTGTAATAAAATTTACTGCCCCTAAAATTGAGGAAATTCCAGCTAAATGAAGAGAAAAAATAGCTAAATCAACAGAAGCTCCAGCATGAGCTGTTCCTGAAGAGAGAGGAGGATAAACTGTTCACCCAGTTCCTGCTCCATTTTCTACTATTGAGCTTGATAATAGAAGAGTCAATGAAGGAGGTAGTATTCAAAAACTTATATTATTTATTCGAGGAAAGGCTATATCAGGGGCTCCTAATATTAAAGGAACTAATCAATTTCCAAATCCTCCA

>LA_BHT_19M3

AATAAGTAGTTGGTATAAAATAGGATCTCCTCCTCCGATTGGATCAAAGAAAGATGTATTTTAAGTTTCGGTCTGTTAATAACATAGTAATAGCTCCAGCTAAAACAGGAAGAGAAAGAAGTAATAAGATAGCTGTAATAACTACAGATCAAACAAATAAAGGTAGTCGATCTAAAGTAATTCCTGACGATCGTATATTAATTACAGTTGTAATAAAATTTACTGCCCCTAAAATTGAGGAAATTCCAGCTAAATGAAGAGAAAAAATAGCTAAATCAACAGAAGCTCCAGCATGAGCTGTTCCTGAAGAGAGAGGAGGATAAACTGTTCACCCAGTTCCTGCCCCATTTTCTACTATTGAGCTTGATAATAGAAGAGTCAATGAAGGAGGTAGTATTCAAAAACTTATATTATTTATTCGAGGAAAGGCTATATCAGGGGCTCCTAATATTAAAGGAACTAATCAATTTCCAAATTCCCTCCA

>LA_BHT_19M4

GATAAGTGTTGGTATAAAATAGGATCTCCTCCTCCGATTGGATCAAAGAAAGATGTATTTAAGTTTCGGTCTGTTAATAACATAGTAATAGCTCCAGCTAAAACAGGAAGAGAAAGAAGTAATAAGATAGCTGTAATAACTACAGATCAAACAAATAAAGGTAGTCGGTCTAAAGTAATTCCTGACGATCGTATATTAATTACAGTTGTAATAAAATTTACTGCCCCTAAAATTGAGGAAATTCCAGCTAAATGAAGAGAAAAAATAGCTAAATCAACAGAAGCTCCAGCATGAGCTGTTCCTGAAGAGAGAGGAGGATAAACTGTTCACCCAGTTCCTGCCCCATTTTCTACTATTGAGCTTGATAATAGAAGAGTCAATGAAGGAGGTAGTATTCAAAAACTTATATTATTTATTCGAGGAAAGGCTATATCAGGGGCTCCTAATATTAAAGGAACTAATCAATTTCCAAATTTCCTCCA

>LA_BHT_19M5

AGTGTTGGTATAAAATAGGGTCTCCCCCTCCGATTGGATCAAAGAAAGATGTATTTAAGTTTCGGTCTGTTAATAATATAGTAATAGCTCCAGCTAAAACAGGAAGAGAAAGAAGTAATAAGATAGCTGTAATAACTACAGATCAAACAAATAAGGGTAGTCGATCTAAAGTAATCCCTGACGATCGCATATTAATCACAGTTGTAATAAAATTTACTGCCCCTAAAATTGAGGAAATTCCAGCTAAATGAAGAGAAAAAATAGCTAAATCAACAGAAGCTCCAGCATGAGCTGTTCCTGAAGAGAGAGGAGGATAAACTGTTCACCCAGTTCCTGCCCCATTTTCTACTATTGAGCTTGATAATAGAAGAGTCAATGAAGGAGGTAGTATTCAAAAACTTATATTATTCATTCGAGGGAAAGCTATATCAGGGGCTCCTAATATTAAAGGAACTAATCAATTTCCAAATTCCCTCCCA

>LA_BHT_19M6

GATAAGTGTTGGTATAAAATAGGATCTCCTCCTCCGATTGGATCAAAGAAAGATGTATTTAAGTTTCGGTCTGTTAATAACATAGTAATAGCTCCAGCTAAAACAGGAAGAGAAAGAAGTAATAAGATAGCTGTAATAACTACAGATCAAACAAATAAAGGTAGTCGATCTAAAGTAATTCCTGACGATCGTATATTAATTACAGTTGTAATAAAATTTACTGCCCCTAAAATTGAGGAAATTCCAGCTAAATGAAGAGAAAAAATAGCTAAATCAACAGAAGCTCCAGCATGAGCTGTTCCTGAAGAGAGAGGAGGATAAACTGTTCACCCAGTTCCTGCTCCATTTTCTACTATTGAGCTTGATAATAGAAGAGTCAATGAAGGAGGTAGTATTCAAAAACTTATATTATTTATTCGAGGAAAGGCTATATCAGGGGCTCCTAATATTAAAGGAACTAATCAATTCCAAATTTCCTCCA

>LA_BHT_19M7

GATAAGTGTTGGTATAAAATAGGATCTCCTCCTCCGATTGGATCAAAGAAAGATGTATTTAAGTTTCGGTCTGTTAATAACATAGTAATAGCTCCAGCTAAAACAGGAAGAGAAAGAAGTAATAAGATAGCTGTAATAACTACAGATCAAACAAATAAAGGTAGTCGATCTAAAGTAATTCCTGACGATCGTATATTAATTACAGTTGTAATAAAATTTACTGCCCCTAAAATTGAGGAAATTCCAGCTAAATGAAGAGAAAAAATAGCTAAATCAACAGAAGCTCCAGCATGAGCTGTTCCTGAAGAGAGAGGAGGATAAACTGTTCACCCAGTTCCTGCCCCATTTTCTACTATTGAGCTTGATAATAGAAGAGTCAATGAAGGAGGTAGTATTCAAAAACTTATATTATTTATTCGAGGAAAGGCTATATCAGGGGCTCCTAATATTAAAGGAACTAATCAATTTCCAAATTTCCTCCA

>LA_BHT_19M8

CCGACTAGTCAATTTCCAAATCCTCCGAACTARTCAATTTCCAAATCCTCCACGCTCATCAATTTCCATATCCTCCACTCTAGTCAATTGCCGAAGCCTCCGAAGTAATAAGATAGCTGATCTACCTACAGATCAAACTTATAAAGGGAGTCGATCTAAAGTTTTTCCTGACGATCGTATATTAATTACRGTTGTAATAAAATTTACTGCCCCTAAAATTGAGGAAATTCCAGCTAAATGAAGAGAGTTATAGCTAAATCAGCAGAAGCTCCARCATGAGCTGTTCCTGAAGAGAGAGGAGGATRAACTGTTCACCCAGYTCCTGCCGCCTTTTCTACTATTGAGCTTGATAATAGAAGAGTCAATGAAGGAGGTAGTATTCAAAAACTTATATTATTYATTCGAGGGGAGGCTGTGTCAGGGGCTCCTAATATGAAAGGAACTAATCATTTTCTCAATCCTCCA

>LA_BHT_19M9

GAATAAGTGTTGGTATAAAATAGGATCTCCTCCTCCGATTGGATCAAAGAAAGATGTATTTAAGTTTCGGTCTGTTAATAACATAGTAATAGCTCCAGCTAAAACAGGAAGAGAAAGAAGTAATAAGATAGCTGTAATAACTACAGATCAAACAAATAAAGGTAGTCGATCTAAAGTAATTCCTGACGATCGTATATTAATTACAGTTGTAATAAAATTTACTGCCCCTAAAATTGAGGAAATTCCAGCTAAATGAAGAGAAAAAATAGCTAAATCAACAGAAGCTCCAGCATGAGCTGTTCCTGAAGAGAGAGGAGGATAAACTGTTCACCCAGTTCCTGCCCCATTTTCTACTATTGAGCTTGATAATAGAAGAGTCAATGAAGGAGGTAGTATTCAAAAACTTATATTATTTATTCGAGGAAAGGCTATATCAGGGGCTCCTAATATTAAAGGAACTAATCAATTTCCAAATTTCCTCCA

>LA_BHT_19M10

AGTGTTGGTATAAAATAGGATCTCCTCCTCCGATTGGATCAAAGAAAGATGTATTTAAGTTTCGGTCTGTTAATAACATAGTAATAGCTCCAGCTAAAACAGGAAGAGAAAGAAGTAATAAGATAGCTGTAATAACTACAGATCAAACAAATAAAGGTAGTCGGTCTAAAGTAATTCCTGACGATCGTATATTAATTACAGTTGTAATAAAATTTACTGCCCCTAAAATTGAGGAAATTCCAGCTAAATGAAGAGAAAAAATAGCTAAATCAACAGAAGCTCCAGCATGAGCTGTTCCTGAAGAGAGAGGAGGATAAACTGTTCACCCAGTTCCTGCCCCATTTTCTACTATTGAGCTTGATAATAGAAGAGTCAATGAAGGAGGTAGTATTCAAAAACTTATATTATTTATTCGAGGAAAGGCTATATCAGGGGCTCCTAATATTAAAGGAACTAATCAATTTCCAAATTCCCTCCCA

>LA_BHT_19M11

TTCAGTATCAGATAGTGTTGGTATAAAATAGGGTCTCCCCCTCCGATTGGATCAWAGAAAGATGTATTTAAGTTTCGGTCTGTTAATAATATAGTAATAGCTCCAGCTAAAACAGGAAGAGAAAGAAGTAATAAGATAGCTGTAATAACTACAGATCAAACAAATAAGGGTAGTCGATCTAAAGTAATCCCTGACGATCGCATATTAATCACAGTTGTAATAAAATTTACTGCCCCTAAAATTGAGGAAATTCCAGCTAAATGAAGAGAAAAAATAGCTAAATCAACAGAAGCTCCAGCATGAGCTGTTCCTGAAGAGAGAGGAGGATAAACTGTTCACCCAGTTCCTGCCCCATTTTCTACTATTGAGCTTGATAATAGAAGAGTCAATGAAGGAGGTAGTATTCAAAAACTTATATTATTCATTCGAGGGAAAGCTATATCAGGGGCTCCTAATATTAAAGGAACTAATCATTTCCAAAATCCCCCCACATAA

>LA_BHT_19M12

ATGTGTTGCGTATAAAATAGGGTCTCCCCCTCCGATTGGGTCAAAGAAAGATGTATTTAAGTTTCGGTCTGTTAATAATATAGTAATAGCTCCAGCTAAAACAGGAAGAGAAAGAAGTAATAAGATAGCTGTAATAACTACAGATCAAACAAATAAGGGTAGTCGATCTAAAGTAATTCCTGACGATCGTATATTAATTACAGTTGTAATAAAATTTACTGCCCCTAAAATTGAGGAAATTCCAGCTAAATGAAGAGAAAAAATAGCTAAATCAACAGAAGCTCCAGCATGAGCTGTTCCTGAAGAGAGAGGAGGATAAACTGTTCACCCAGTTCCTGCCCCATTTTCTACTATTGAGCTTGATAATAGAAGAGTCAATGAAGGAGGTAGTATTCAAAAACTTATATTATTCATTCGAGGGAAAGCTATATCAGGGGCTCCTAATATTAAAGGAACTAATCAACTCCCAATTCCCCCCCCA

>LA_BHT_19M13

CGTATGCATACCTATAGGGTCTCCCCCTACAATTGCGATACATAAAAAGTTGTATTTTACTTTCGGTCTGTTAATAATATAGTAATAGCTCCGGCTAATACGGGTAGAGAAAGAAGTAATAAAATAGCTGTAATTACTACTGATCACACAAATAAAGGTAGTCGATCAAGAGTAATACCAGCTGATCGTATATTAATTACAGTTGTAATAAAATTTACTGCTCCTAAAATAGATGAGATTCCCGCTAAATGTAAAGAAAAAATTGCTAAATCAACTGAAGCCCCAGCATGAGCTGTTCCAGAAGAAAGGGGAGGATAAACCGTTCACCCTGTTCCAGCTCCGTTTTCTACTATAGAACTAGAAAGCAGCAGTGTTAAAGAGGGGGGTAATATTCAAAAACTTATATTATTTATTCGAGGAAAAGCTATATCAGGGGCTCCTAGTATTAAGGGAACTAATCAATTTACAAATTCCCCCAA

>LA_BHT_19M14

AGATAAGTGTTGGTATAAAATAGGATCTCCTCCTCCGATTGGATCAAAGAAAGATGTATTTAAGTTTCGGTCTGTTAATAACATAGTAATAGCTCCAGCTAAAACAGGAAGAGAAAGAAGTAATAAGATAGCTGTAATAACTACAGATCAAACAAATAAAGGTAGTCGATCTAAAGTAATTCCTGATGATCGTATATTAATTACAGTTGTAATAAAATTTACTGCCCCTAAAATTGAGGAAATTCCAGCTAAATGAAGAGAAAAAATAGCTAAATCAACAGAAGCTCCAGCATGAGCTGTTCCTGAAGAGAGAGGAGGATAAACTGTTCACCCAGTTCCTGCCCCATTTTCTACTATTGAGCTTGATAATAGAAGAGTCAATGAAGGAGGTAGTATTCAAAAACTTATATTATTTATTCGAGGAAAGGCTATATCAGGGGCTCCTAATATTAAAGGAACTAATCAATTCCAAATCCCCCCAA

>LA_BHT_19M15

CTAATCAATTTCCAATCCTCCAAACTAATCAATTTCCAAATCCTCCCAAGAATGTTTTTCCATATCCTCAGCTCTTGGTAAAAGAGGAAGAGAAAGAAGTAATAAGATAGCTGTAATAACTACAGATCAAACAAATGAAGGTAGTCGATCTAAAGTAATTCCTGATGATCGTATATTAATTACAGTTGTAATAAAATTTACTGCCCCTAAAATTGAGGAAATTCCAGCTAAATGAAGAGAAAAAATAGCTTAATCAACAGAAGCTCCAGCATGAGCTGTTCCTGAARAGAGAGGAGGATAAACTGTTCACCCAGTTCCTGCTCCATTTTCTACTATTGAGCTTGATAATAGAAGAGTCAATGARGGAGGTAGTATTCAAAAACTTATATTATTTATTCGAGGAAAGGCTATATCAGGGGCTCCTAATATTAAAGGAACTAATCAATTTCCAAATCCTCCAAA

>LA_BHT_19M16

AGATAAGTGTTGGTATAAAATAGGATCTCCTCCTCCGATTGGATCAAAGAAAGATGTATTTAAGTTTCGGTCTGTTAATAACATAGTAATAGCTCCAGCTAAAACAGGAAGAGAAAGAAGTAATAAGATAGCTGTAATAACTACAGATCAAACAAATAAAGGTAGTCGATCTAAAGTAATTCCTGATGATCGTATATTAATTACAGTTGTAATAAAATTTACTGCCCCTAAAATTGAGGAAATTCCAGCTAAATGAAGAGAAAAAATAGCTAAATCAACAGAAGCTCCAGCATGAGCTGTTCCTGAAGAGAGAGGAGGATAAACTGTTCACCCAGTTCCTGCCCCATTTTCTACTATTGAGCTTGATAATAGAAGAGTCAATGAAGGAGGTAGTATTCAAAAACTTATATTATTTATTCGAGGAAAGGCTATATCAGGGGCTCCTAATATTAAAGGAACTAATCATTTCCAAATTCCCCCCCA

>LA_BHT_19M17

TCAGTTTCCTGTTCCGTATAAAATAGGATCTCCTCCTCCGATTGGATCAAAGAAAGATGTATTTAAGTTTCGGTCTGTTAATAACATAGTAATAGCTCCAGCTAAAACAGGAAGAGAAAGAAGTAATAAGATAGCTGTAATAACTACAGATCAAACAAATAAAGGTAGTCGATCTAAAGTAATTCCTGACGATCGTATATTAATTACAGTTGTAATAAAATTTACTGCCCCTAAAATTGAGGAAATTCCAGCTAAATGAAGAGAAAAAATAGCTAAATCAACAGAAGCTCCAGCATGAGCTGTTCCTGAAGAGAGAGGAGGATAAACTGTTCACCCAGTTCCTGCTCCATTTTCTACTATTGAGCTTGATAATAGAAGAGTCAATGAAGGAGGTAGTATTCAAAAACTTATATTATTTATTCGAGGAAAGGCTATATCAGGGGCTCCTAATATTAAAGGAACTAATCAATTTCCAATTCCTCCA

>LA_BHT_19M18

GTATCAGTTTCGTGTTCCGTATAAAATAGGATCTCCTCCTCCGATTGGATCAAAGAAAGATGTATTTAAGTTTCGGTCTGTTAATAACATAGTAATAGCTCCAGCTAAAACAGGAAGAGAAAGAAGTAATAAGATAGCTGTAATAACTACAGATCAAACAAATAAAGGTAGTCGATCTAAAGTAATTCCTGACGATCGTATATTAATTACAGTTGTAATAAAATTTACTGCCCCTAAAATTGAGGAAATTCCAGCTAAATGAAGAGAAAAAATAGCTAAATCAACAGAAGCTCCAGCATGAGCTGTTCCTGAAGAGAGAGGAGGATAAACTGTTCACCCAGTTCCTGCCCCATTTTCTACTATTGAGCTTGATAATAGAAGAGTCAATGAAGGAGGTAGTATTCAAAAACTTATATTATTTATTCGAGGAAAGGCTATATCAGGGGCTCCTAATATTAAAGGAACTAATCAATTCCAAAATTTCCTCCCA

>LA_BHT_19M19

AGATAAGTGTTGGTATAAAATAGGATCTCCTCCTCCGATTGGATCAAAGAAAGATGTATTTAAGTTTCGGTCTGTTAATAACATAGTAATAGCTCCAGCTAAAACAGGAAGAGAAAGAAGTAATAAGATAGCTGTAATAACTACAGATCAAACAAATAAAGGTAGTCGATCTAAAGTAATTCCTGACGATCGTATATWAATTACAGTTGTAATAAAATTTACTGCCCCTAAAATTGAGGAAATTCCAGCTAAATGAAGAGAAAAAATAGCTAAATCAACAGAAGCTCCAGCATGAGCTGTTCCTGAAGAGAGAGGAGGATAAACTGTTCACCCAGTTCCTGCTCCATTTTCTACTATTGAGCTTGATAATAGAAGAGTCAATGAAGGAGGTAGTATTCAAAAACTTATATTATTTATTCGAGGAAAGGCTATATCAGGGGCTCCTAATATTAAAGGAACTAATCATCACCAATTTCCCCCCA

>LA_BHT_19M20

TTCGGTATAAAATAGGATCTCCTCCTCCGATTGGATCARAGAAAGATGTATTTAAGTTTCGGTCTGTTAATAACATAGTAATAGCTCCAGCTAAAACAGGAAGAGAAAGAAGTAATAAGATAGCTGTAATAACTACGGATAAAACAAATAAGGGTAGTCGATCTAAGGTAATTCCGGACGATCGTATATTAATTACAGTTGTAATAAAATTTACTGCCCCGAAAATTGAGGAAATTCCGGCTAAATGAAGAGAAAAAATAGCTTAGTCAACAGAAGCTCCCGCATGAGCTGTTCCTGAAGAGAGAGGAGGATAAACTGTTCACCCAGTTCCTGCTCCAKTTTCTACTATGGAGCTTGATCATAGAAGAGTCAATGAAGGAGGTAGTATTCAGAAACTTATATAATTTATTCGAGGAAAGGCTATATGAGGGGCTCCGAAAATGAGGGGAACTAAGCATTTTCCAAATCCCCCA

>LA_BHT_19M21

GTGTTCGGTATAAAATAGGATCTCCTCCTCCGATTGGATCAAAGAAAGATGTATTTAAGTTTCGGTCTGTTAATAACATAGTAATAGCTCCAGCTAAAACAGGAAGAGAAAGAAGTAATAAGATAGCTGTAATAACTACAGATCAGACAAATAAGGGTAGTCGGTCTAAAGTAATTCCTGACGATCGTATATGAATTACAGTTGTAATAAAATTTACTGCCCCTGAAATTGAGGAAATTCCAGCTAAATGAAGAGAAAAAATAGCTAAATCAACAGAAGCTCCAGCATGAGCTGTTCCTGAAGAGAGAGGAGGATAAACTGTTCACCCAGTTCCTGCCCCATTTTCTACTATCGAGCTTGATAATAGAAGAGTCAATGAAGGAGGTAGTATTCAGGAACTTAAATTATTTATCCGAGGAAAGGGTATTTGAGGGGCTCCGGATATTAGGGGAACTGGGCACTTTCCAGATCCGCCC

>LA_BHT_19M22

CAGTTGGGTCTGACCTCCCCTGGATGGAAGAGAATAATTATTTTCTTTTTGTTCTGTTAAAAATATTAGATTCGGTCCATATGGGTAGAGAAGAAATAATAAATTATCTTTGTTACACCTAATCGACATTTGGGTGGGTAATCGAATGAGCCTTCTAAAGATTTATTTACTACTTTGTAATTTCGGCTCCCCCACAAAAAAGCCTGCTCTTGTAGTAAAATTGCAGTCCCCACTACCTCGCCGAGCTGTACAGGGGGAAAAGATTTCGCCTTCCTCCCGCCCTTTTCCTTGGCTGGAGATCTAAAGTGGGGGGCGATATATTTATTTTCAGAGGGTTGTGGGGCTGGGGTCGCGGGAGGACTTTATTGCCCCCCCCCCCG

>LA_BHT_19M23

CGTGTTCGCGTATAAAATAGGATCTCCTCCTCCGATTGGATGAAAGAAAGATGTATTTAAGTTTCGGTCTGTTAATAACATAGTAATAGCTCCCGCTAAAACAGGAGGAGAAAGAAGTAATAAGATAGCTGTAAGAACTACAGATSAAACAAATAARGGTAGTCGATCTAAAGTAATTCCTGACGATTGTATGTAAATTACAGTTGTAATAAAATTTACTGCCCCTAAAATTGAGGAAATTCCTGCTAAATGAAGAGAAAAAATAGCTAAATCAACAGAAGCTCCCGCAAGAGCGGTTCCTGAAGAGAGAGGAGGATAAACTGTTCACCCAGTTCCTGCTCCATTTTCTACTATCGAGCTTGATTAGAGAAGAGTCAATGAAGGAGGGAGTATTCAAAAACTTATCTAATTTATTCGAGGAAGGCTATGTTAGGGGCTCCGGACATGATAGGAACTGA

>LA_BHT_19M24

TCAGAGAGGGTCTCCCCCTCCCCTTGGATAGAAGAAGAAATAATTTATTTTCGGTGTGTTATGTAAATAATAAAAGCTCCGGTCCATACGGGTAGGAAGAAAGGATAAAATAGCTCTCTTACGACTGATCACACCAATTAAGGTAGTCTATGGAAAGTAATACCATCCCATAGTATGTTAATTACTTTTGTGTTAAAATTTACTGCTCCGCCCCTGATCAGATCCCTGCTCAATTTACAGCAAAAATAGCTATCTCAACTTGCTCCCCCGCCAGAGCTTGCCGTGAACGCTGGGAAAGAGAGAGCGTTAACGCTCTCCGTCCSCCGCCTCCCTGCCGAATTTGCTGGTAGAGGGCTTAATGGGGAGGGAGGATGTAGAAGACGAAATCTTCATACGTTAAACTATTTATGCGGGTGAGGATCTCCATGGAGCTCCTGAATTTACCGGATCTTCACCCTTCCCAGGAG

>LA_BHT_19M25

GGTGTTGGTATAAAATAGGATCTCCTCCTCCGATTGGATGAGAGAAGATGTATTTTAGTTTCGGTCTGTTAATAACATAGTAATAGGTCCGGCTTGACAGGGGAGGAAGAAGTAATAAGATAGCTTTGATAACTACAGATAAGACAAATGAGGGTGGGGGATATGATTGATTCGCCGAGAAGAGTATATTAATTACAGTTGTGATAAAATTTACCGCGCCGCCATTGAGGAGTTCCAGCTCAATGAAGAGAGGAATAGCTAACTCATCATAAGCTCCGGCATGAGCTGGTCCTGAAGAGGGAGGAGGAGAACTGTTACCCAGTTGCTGCCGCCTTTTCTGCTATGAGGTTGATGATGGAAGAGTCGATAATGGAGGTAGTAGTCAAAACTTATTAATTATTCGAGGATGGGGGCTGGCGGGGTGCCGGGATCGAGGGAACGAAGGAACTTCCTCATCCTCC

>LA_BHT_19M26

AATATAGGATGTCCTCCTCCTATTGGATGAAAGAAGGATGTATTTAATTTTCGGTCTGTTGATAACAAAGTAGTAGCTCCTTCCGACAAAGGCGGAGAAAGAAGTAGTAAAATAGCTGCCTAACTACCTACATACAAATCATGGTGGGGTATCTATCTAATTCCTTCGATATATCTTATTACTTTTCTGATGTTTTTACGGCCCTGCCCCTGAGTTGTTCCATTCCTTCAGGAGAAGAGTAGCTATATCTCCATCACCTCCAGCTCCAGCTTTTGCTGTTCATGGAGAGAAAGAACGGTGTCTGTTCACCGGCTTCCTGTCCCATTTTCTACTTTTGGGCTAGAAATAGACGAGGCGAGGAGGGAGGTTCAAACTCATATACTTTATCATTTAGACGGGGTATGGCGGGCTCCCGGGCTCCCAAGATCACAGGAACTTTCCAACTCCCCCACACC

>LA_BHT_19M27

GTGTTCGCGTATAAAATAGGATCTCCTCCTCCGATTGGATCAGAGAAAGATGTATTTAAGTTTCGGTCTGTTAATAACATAGTAATAGCTCCAGCTAAAACAGGAAGAGAAAGAAGTAATAAGATAGCTGTAATAACTACAGATCAAACAAATAAAGGTAGTCGATCTAAAGTAATTCCTGACGATCGTATATTAATTACAGTTGTAATAAAATTTACTGCCCCTAAAATTGAGGAAATTCCAGCTAAATGAAGAGAAAAAATAGCTAAATCAACAGAAGCTCCAGCATGAGCTGTTCCTGAAGAGAGAGGAGGATAAACTGTTCACCCAGTTCCTGCCCCATTTTCTACTATTGAGCTTGATAATAGAAGAGTCAATGAAGGAGGTAGTATTCAAAAACTTATATTATTTATTCGAGGAAAGGCTATATTAGGGGCTCCTAATATTAAAGGAACTAATCACTTACCCAATCCACCC

>LA_BHT_19M29

GTGTTCGGTATAAAATAGGATCTCCTCCTCCGATTGGATTAAAGAAGGATGTATTTAAGTTTCGGTCTGTTAATAAGGGAGTAGTAGCTCCGGCTAAGCAGGGAGAGAAAGAAGTAATAAGATAGCTGTAATAACTACAGATGAGACAGATGGGGTAGTCGATCTGAGTAATTCCGGAGGATAGGATATTAGTTACTGTTGTAATAAAATTTACTGCCCCCAAATTGAGGAAATTCCTGCTAATTGAAGAGGAGAAATAGCTGATTCAACAGAAGCTCCGGCATGAGCTGTTCCAGAAGAGAGGGGAGGAGAACTGTTGGCCCGGGTTCCTGCCCCCTTTACTACCATGGAGCTGGATAAGGAAGAGTCAGTGGAGGAGGGAGTATTCAGAAACTTAGTTGAGGTTGTTCGAGGAGGGGCGAGGTTATGGGCGGCGCAGGTTATAGGGG

>LA_BHT_19M30

GTGTTGGTATAAAATAGGATCTCCTCCTCCGATTGGATCAGAGAAAGATGTATTTAAGTTTCGGTCTGTTAATAACATAGTAATAGCTCCAGCTAAGACAGGAAGAGAAAGAAGTAATAAGATAGCTGTAATAACTACCGATCAAACAAATGAAGGTAGTCGATCTGAAGTAATTCCTGACGATGGTATATTAATTACAGTTGTAATAAAATTTACTGCCCCTCAGATTGAGGAAATTCCCGCTAAATGAAGAGAAGAAATAGCTGAATCAACAGAAGCTCCAGCAGGAGCTGTTCCTGAAGAGAGAGGAGGAKAAACTGTTGACCCAGTTCCTGCTCCAATTTCTACTATGGAGTTTGATGATAGAAGAGTCGATGAAGGAGGTAGTATTCATAAACTTATATTATTTATTCGAGGAAGGGC

>LA_BHT_19M31

GTGTTGCGTATAAAATAGGATCTCCTCCTCCGATTGGATCAGAGAAAGATGTATTTAAGTTTCGGTCTGTTAATAACATAGTAATAGCTCCAGCTAAAACAGGAAGAGAAAGAAGTAATAAGATAGCTGTAATAACTACAGATCAAACAAATAAAGGTAGTCGATCTAAAGTAATTCCTGACGATCGTATATTAATTACAGTTGTAATAAAATTTACTGCCCCTAAAATTGAGGAAATTCCAGCTAAATGAAGAGAAAAAATAGCTAAATCAACAGAAGCTCCAGCATGAGCTGTTCCTGAAGAGAGAGGAGGATAAACTGTTCACCCAGTTCCTGCTCCATTTTCTACTATTGAGCTTGATAATAGAAGAGTCAATGAAGGAGGTAGTATTCAAAAACTTATATTATTTATTCGAGGAAAGGCTATATAAGGGGCTCCTAATATTAAAGGAACTAATCAATTTCAAAATCCCGCCA

>LA_BHT_19M32

TTGTGGCAGTTTTCGTTTAAGATAGGAATCCTGGGCCTATCGGGAGGAGAGGAGGTAGATGGTTTCGGTCTGTTAGGAGAGAGTATAGCTCCGCTCGGACCGGATAGGAAGAAGTATAATATGCTTGATAACTACGAGAGCGAGGAGGGTAGTCGATCTGAGTAATTCCTGACGATGGGAATATTACGTTGTATAAATTTACGCCCCGTATTGAGGATTCCGCTATAAAGAAATAGCTATAACAAACTCCGCAGAGCTGTTCCGAAAGGAGAGGAGAAACTGTTAACCCGTTCCGCTCCTTTTCACATGATTGATATGAAGAGTCATGAGGGAGGTCGTAGTCGAAACTTAATTATTATCTAGGATGGCTATTGGGGGCGCCGATATGGGGGACTGTCATTCCGGATTCCCCTCCG

>LA_BHT_19M33

TACATCAGATAGATAAGTGTTGGTATAAAATAGGATCTCCTCCTCCGATTGGATCAAAGAAAGATGTATTTTACTTTCGGTCTGTTAATAACAGAGTATAGCTCCCCTGAAGAGGAAGAGAAAGAAGTTTAAGATAGCTACGTTACTACGATAAGGGCATCTGGTAGTCGGTTCCTGACTTCCTGATATAGTTAATCATTACGTTTTCAGCACTCTGCCCCGCTATGAGGACTTACTGATTTAACAGGTACTATTTCCGGACGAGCGTCCAGGCATCCGAGAGAGCGGGAGCTGTCCCCTCTTTTGCTACGTTCGTAAGTGATAGTGAATGTCGAGCAGAGTGTTAATTCTATCGGGGGGGGAGTCGGTGCGGCTCAGATCCCTTTCTCCCCGCGACGGA

>LA_BHT_19M34

AGGGGGTAAAGGAGTATGAGTAAGTATCTGATAGTGTTGGGATAACTAGGATCTCCTGCTCCGATTGGATCAAGATGATGTATTTTAGTTTCGGTCTGTTAATAACGTTGTACTGCTCCGGCTGAACAGGAGAGAAAGAAGTCATTAGATAGCTCTTATAACTACCGATCAGGCAAATGATGGTAGTCCATCTCAGATAATTCCTGACGATTGGATATTAATTACAGTTGTTATAAAATTTACCGCCCCCAATATTGAAGAAATTCCCGCTAAATTAAGAAAAATAATAACTTACTCCACCAAAACCCCCGCCTGAACAGGTCCAGAAAAAAGAAGAAGAAAAACTGGTCAACCCGTTCCCGCTCCCTTTTTTGATATTGGGATAGATTATAAAAGAAGCAATGAACGAAGGTGTATTCAAAACTTATATTAATTCTTCCAGGAGAGGCTCTTTTATGGGCTCCCAAAATTAATCGGAACTTTCATTTCCCAAATCCTCCGA

>LA_BH_20M1

AATTCGTGTATCAGATAAGTGTTGGTATAAAATAGGATCTCCTCCTCCGATTGGATCAAAGAAAGATGTATTTAAGTTTCGGTCTGTTAATAACATAGTAATAGCTCCTGCTAAAACAGGAAGAGAAAGAAGTAATAAGATTGCTGTAATAACTACGGATCAAACAAATAAGGGTAGTCGATCTAAAGTAATTCCTGACGATGGTATATTAATTACAGTTGTAATAAAATTTACTGCCCCTAAAATTGAGGAAATTCCAGCTAAATAAAGAGAAAAAATAGCTAAATCAACAGAAGCTCCTGCATGAGCTGTTCCTGAAGAGAGAGGAGGATAAACTGTTCACCCAGTTCCTGCCCCATTTTCTACTATGGAGCTTGATAATAGAAGAGTCAATGAAGGAGGTAGTATTCAAAAACTTATATTATTTATTCGAGGAAAGGCTATATGAGGGGCTCCTAATATGAGAGGAACTAATCACCATCCTAATCCACCA

>LA_BH_20M2

CGTGTTGGTATAAAATAGGATCTCCTCCTCCGATTGGATCAGAGAAAGATGTATTTAAGTTTCGGTCTGTTAATAACATAGTAATAGCTCCAGCTAAAACAGGAAGAGAAAGAAGTAATAAGATAGCTGTAATAACTACCGATCAGACAAATAAAGGTAGTCGATCTAAAGTAATTCCTGACGATGGTATATGAATTACAGTTGTAATAAAATTTACGGCCCCTGATATTGAGGAAATTCCAGCTAATTGAAGAGAAAAAATAGCTAATTCAACAGAAGCTCCGGCATGAGCTGTTCCTGAAGAGAGAGGAGGATAAACTGTTCACCCAGTTCCTGCTCCATTTTCTACTATTGAGCTTGATGATAGAAAAGTCAATGAAGGAGGTGGTAGTCGGAAACTTATATTATTTATTCGAGGAAGGGCGATGTCAGGGGCTCCGGAGATCA

>LA_BH_20M3

CGTAAGTGTTGGTATAACATAGGATCTCCGCCTCCCATCGGATCAGAGAGGATGTATTTGAGTTTCGGTCTGTTAATAACATAGTAGTAGCTCCCGCTAGAGGGAAGAGGAGGAAGTAATAAGATGGCTGTAAGAACTACCGATGAGACGGATAAAGGTGGTCGATCTCAGGTAATTCCCGACCATCGTACATTAGTTACTGTTGTAATAAAATTTACCGCCCCACAGATTGAGGAAATTCCCGCTTAATCAAGAGAAAAAATAGCTTAATCAACATAAGCTCCCGCATGAGCTGTTCCAGAACAGCCCGGAAGAAGAACTGTTCACCCCGTTGCCCCCCCACCCGCTACCCTTTCCCTTGATGGAGTACAATCCTTGAACGAGCTTGTAAGGCAAAACTTACTTTAATTCTT

>LA_BH_20M4

ATTAACCGTAGTTCGCGTATAAAATAGGATCTCCTCCTCCGATTGGATCAAAGAAAGATGTATTTAAGTTTCGGTCTGTTAATAACATAGTAATAGCTCCAGCTAAAACAGGAAGAGAAAGAAGTAATAAGATAGCTGTAATAACTACGGATCAAACAAATAAAGGTAGTCGATCTAAAGTAATTCCTGATGATCGTATATTAATTACAGTTGTAATAAAATTTACTGCCCCTAAGATTGAGGAAATTCCAGCTAAATGAAGAGAAAAAATAGCTAAATCAACAGAAGCTCCCGCATGAGCTGTTCCTGAAGAGAGAGGAGGATAAACTGTTCACCCAGTTCCTGCCCCATTTTCTACTATTGAGCTTGATAATAGAAGAGTCAATGAAGGAGGTAGTATTCAAAAACTTATATTATTTATTCGAGGAAAGGCTATATTAGGGGCTCCTAATATTAAAGGAACTAA

>LA_TT_22F3

TTAACGTGTTGGTATAAAATAGGATCTCCTCCTCCGATTGGATCAAAGAAAGATGTATTTAAGTTTCGGTCTGTTAATAATATAGTAATAGCTCCAGCTAAAACAGGAAGAGAAAGAAGTAATAAGATAGCTGTAATAACTACAGATCAAACAAATAAAGGTAGTCGATCTAAAGTAATTCCTGACGATCGCATATTAATTACAGTTGTAATAAAATTTACTGCCCCTAAAATTGAGGAAATTCCAGCTAAATGAAGAGAAAAAATAGCTAAATCAACAGAAGCTCCAGCATGAGCTGTTCCTGAAGAGAGAGGAGGATAAACTGTTCACCCAGTTCCTGCCCCATTTTCTACTATGGAGCTTGATAATAGAAGAGTCAATGAAGGAGGTAGTATTCAAAAACTTATATTATTTATTCGAGGAAAGGCTATATCAGGGGCTCCTAATATTAAAGGAACTAATCAATCTCCAAATCCTCCA

>LA_TT_22F4

TAGTGATGGTAGTTGTTTGAATCCGCCCTCCTCCGGAATGGGATTAAAGAAAGATGTATTCAGTTTCGGTTTGTTAATAACTACACACTCCAGCTGGAAGGAGAAGTAAGAAGAAGCTATACTCTACAATACACATAAACGGTGTCGAAGTCAATCTTTCCTGTTCTGAAATATATACATTGCAAAGATTACATTTACGGCCCCGATATTACCAGTTCCTCTATAAAAAATAATCCTCATAGCCCCACCTGAGCTGTGCCTGATTACGAGAGGAGAAGGTTCACCCTTTCCCGCTCCCTGCCCCTTTGACTTGGAATTGGATATCATGAGGGATTAGGGAGCGGAACTCAATAATTTATTCAAGTAAGGCGAATGGGGTCTCCGGGGTCCAGACAAGGTTCCGCCCTTTCCCACTCCGCCA

>LA_TT_22F5

ATAGGGTCTCCTCCTCCACTTGGATCAAAAAAAGATGTATTTAAATTCAGGTCTGTTAATAATATAGTAATAGCTCCGGCTAATACGGGTAGAGAAAGAAGTAATAAAATAGCTGTAATTACTACTGATCACACAAATAAAGGTAGTCGATCAAGAGTAATACCAGCTGATCGTATATTAATTACAGTTGTAATAAAATTTACTGCTCCTAAAATAGATGAGATTCCCGCTAAATGTAAAGAAAAAATTGCTAAATCAACTGAAGCCCCAGCATGAGCTGTTCCAGAAGAAAGGGGAGGATAAACCGTTCACCCTGTTCCAGCTCCGTTTTCTACTATAGAACTAGAAAGCAGCAGTGTTAAAGAGGGGGGTAATATTCAAAAACTTATATTATTTATTCGAGGAAAAGCTATATCAGGGGCTCCTAGTATTAAGGGAACTAATCAATTCCAAAATTCCCCCCA

>LA_TT_22F6

AATGTAAACGTGTTCGGTATAAAATAGGATCTCCTCCTCCGATTGGATCAAAGAAAGATGTATTTAAGTTTCGGTCTGTTAATAACATAGTAATAGCTCCAGCTAAAACAGGAAGAGAAAGAAGTAATAAGATAGCTGTAATAACTACAGATCAAACAAATAAAGGTAGTCGATCTAAAGTAATTCCTGACGATCGTATATTAATTACAGTTGTAATAAAATTTACTGCCCCTAAAATTGAGGAAATTCCAGCTAAATGAAGAGAAAAAATAGCTAAATCAACAGAAGCTCCAGCATGAGCTGTTCCTGAAGAGAGAGGAGGATAAACTGTTCACCCAGTTCCTGCTCCATTTTCTACTATTGAGCTTGATAATAGAAGAGTCAATGAAGGAGGTAGTATTCAAAAACTTATATTATTTATTCGAGGAAAGGCTATATCAGGGGCTCCTAATATTAAAGGAACTAATCAATTTCTCAAATCCTCCAAAAA

>LA_TT_22F7

AGGGTAGTTCCGTATAAAATAGGATCTCCTCCTCCGATTGGATCAGAGAGGGATGTGTTTGAGTTTCGGTCTGTTAATGGGGGGGTAATAGCTCCCGCTGACAGGGGAAGAGAAGGAAGTAATAAGATCGCTGTCACAACTACCGATTAGAGGATGAGGGTTGTCGATTTGATGTATTTCCGGAAGATGGGATGTGGGTTACGGTTGTAAAAATATTTACTGCCCCTATATTGAGGATTTTCCCTCTCGTAAAGAGAAAAAATAGCTTACTCAACAGAATCTCCCGCGTGAGCTGTTCCTGAAGAGAGGGGAAGARAAACTGTTCACCCTTTCCCGGCCCCGTTTTCCACTATAGAGCTAGAGTGAGGAGAGTCTGTGAGGGGGGTGGTGGTCATACTCTTATTTTATTTATTCGAGGAGGGGGTATGTTGGTGGGCCCGGGATTTTAGGAGAGCGGGGTTTCTTCCTGGCCCGAGCAGCCCGGGG

>LA_TT_22F8

GTAGTTCGGTATAAAATAGGATCTCCTCCTCCGATTGGATCAAAGAAAGATGTATTTAAGTTTCGGTCTGTTAATAACATAGTAATAGCTCCAGCTAAAACAGGAAGAGAAAGAAGTAATAAGATAGCTGTAATAACTACAGATCAAACAAATAARGGTAGTCGATCTAAAGTAATTCCTGACGATCGTATATTAATTACAGTTGTAATAAAATTTACTGCCCCTAAAATTGAGGAAATTCCAGCTAAATGAAGAGAAAAAATAGCTAAATCAACAGAAGCTCCAGCATGAGCTGTTCCTGAAGAGAGAGGAGGATAAACTGTTCACCCAGTTCCTGCCCCATTTTCTACTATTGAGCTTGATAATAGAAGAGTCAATGAAGGAGGTAGTATTCAAAAACTTATATTATTTATTCGAGGAAAGGCTATATCAGGGGCTCCTAATATTAAAGGAACTAATCAATTTCAAAATTCCTCCAA

>LA_TT_22F9

GTACCGTGTTCGGTATAAAATAGGATCTCCTCCTCCGATTGGATCAAAGAAAGATGTATTTAAGTTTCGGTCTGTTAATAACATAGTAATAGCTCCAGCTAAAACAGGAAGAGAAAGAAGTAATAAGATAGCTGTAATAACTACAGATCAAACAAATAAAGGTAGTCGATCTAAAGTAATTCCTGACGATCGTATATTAATTACAGTTGTAATAAAATTTACTGCCCCTAAAATTGAGGAAATTCCAGCTAAATGAAGAGAAAAAATAGCTAAATCAACAGAAGCTCCAGCATGAGCTGTTCCTGAAGAGAGAGGAGGATAAACTGTTCACCCAGTTCCTGCTCCATTTTCTACTATTGAGCTTGATAATAGAAGAGTCAATGAAGGAGGTAGTATTCAAAAACTTATATTATTTATTCGAGGAAAGGCTATATCAGGGGCTCCTAATATTAAAGGAACTAATCAATTTACAAAATCCGCCCA

>LA_TT_22F10

GCGTATAAAATAGGATCTCCTCCTCCGATTGGATCAAAGAAAGATGTATTTAAGTTTCGGTCTGTTAATAACATAGTAATAGCTCCAGCTAAAACAGGAAGAGAAAGAAGTAATAAGATAGCTGTAATAACTACAGATCAAACAAATAAAGGTAGTCGATCTAAAGTAATTCCTGACGATCGTATATTAATTACAGTTGTAATAAAATTTACTGCCCCTAAAATTGAGGAAATTCCAGCTAAATGAAGAGAAAAAATAGCTAAATCAACAGAAGCTCCAGCATGAGCTGTTCCTGAAGAGAGAGGAGGATAAACTGTTCACCCAGTTCCTGCTCCATTTTCTACTATTGAGCTTGATAATAGAAGAGTCAATGAAGGAGGTAGTATTCAAAAACTTATATTATTTATTCGAGGAAAGGCTATATCAGGGGCTCCTAATATTAAAGGAACTAATCAATTTACAATTCCTCCCA

>LA_TT_23F1

TAACGTGTTGGTATAAAATAGGATCTCCTCCTCCGATTGGATCAAAGAAAGATGTATTTRAGTTTCGGTCTGTTAATAACATAGTATTAGCTCCTGCTAAAACAGGAAGAGAAAGAAGTAATAAGATAGCTGTAATAACTACTGATCAGACGAATAAGGGTAGTCGATCTTAAGTAATTCCTGACGATAGTATATTAATTACAGTTGTAATAAAATTTACTGCCCCTAATATTGAGGAAATTCCAGCTAAATGAAGAGAAAAAATAGCTAAATCAACAGAAGCTCCAGCATGAGCTGTTCCTGAAGAGAGAGGAGGATAAACTGTTCACCCAGTTCCTGCCCCATTTTCTACTATTGAGCTTGATAATAGAAGAGTCAATGAAGGAGGTAGTATTCAGAAACTTATTTTATTTATTCGAGGAAAGGCTATTTAAGGGGCTCCTAA

>LA_TT_23F2

ATAAGTGTTGGTATAAAATAGGATCTCCTCCTCCGATTGGATCAAAGAAAGATGTATTTAAGTTTCGGTCTGTTAATAACATAGTAATAGCTCCAGCTAAAACAGGAAGAGAAAGAAGTAATAAGATAGCTGTAATAACTACAGATCAGACAAATAAAGGTAGTCGATCTAAAGTAATTCCTGACGATCGTATATTAATTACAGTTGTAATAAAATTTACTGCCCCTAAAATTGAGGAAATTCCAGCTAAATGAAGAGAAAAAATAGCTAAATCAACAGAAGCTCCAGCATGAGCTGTTCCTGAAGAGAGAGGAGGATAAACTGTTCACCCAGTTCCTGCCCCATTTTCTACTATTGAGCTTGATAATAGAAGAGTCAATGAAGGAGGTAGTATTCAAAAACTTATATTATTTATTCGAGGAAAGGCTATATCAGGGGCTCCTAATATTAAAGGAACTAATCAATTTCCAAATCCTCCA

>LA_TT_23F3

ATCAGAATAAGTGTTGGTATAAAATAGGATCTCCTCCTCCGATTGGATCAAAGAAAGATGTATTTAAGTTTCGGTCTGTTAATAACATAGTAATAGCTCCAGCTAAAACAGGAAGAGAAAGAAGTAATAAGATAGCTGTAATAACTACAGATCAAACAAATAAAGGTAGTCGATCTAAAGTAATTCCTGACGATCGTATATTAATTACAGTTGTAATAAAATTTACTGCCCCTAAAATTGAGGAAATTCCAGCTAAATGAAGAGAAAAAATAGCTAAATCAACAGAAGCTCCAGCATGAGCTGTTCCTGAAGAGAGAGGAGGATAAACTGTTCACCCAGTTCCTGCCCCATTTTCTACTATTGAGCTTGATAATAGAAGAGTCAATGAAGGAGGTAGTATTCAAAAACTTATATTATTTATTCGAGGAAAGGCTATATCAGGGGCTCCTAATATTAAAGGAACTAATCAATACACAATCTCCCCCCCA

>LA_TT_23F4

ATCAGAATAAGTGTTGGTATAAAATAGGATCTCCTCCTCCGATTGGATCAAAGAAAGATGTATTTAAGTTTCGGTCTGTTAATAACATAGTAATAGCTCCAGCTAAAACAGGAAGAGAAAGAAGTAATAAGATAGCTGTAATAACTACAGATCAAACAAATAAAGGTAGTCGATCTAAAGTAATTCCTGACGATCGTATATTAATTACAGTTGTAATAAAATTTACTGCCCCTAAAATTGAGGAAATTCCAGCTAAATGAAGAGAAAAAATAGCTAAATCAACAGAAGCTCCAGCATGAGCTGTTCCTGAAGAGAGAGGAGGATAAACTGTTCACCCAGTTCCTGCCCCATTTTCTACTATTGAGCTTGATAATAGAAGAGTCAATGAAGGAGGTAGTATTCAAAAACTTATATTATTTATTCGAGGAAAGGCTATATCAGGGGCTCCTAATATTAAAGGAACTAATCAATC

>LA_TT_23F5

ATCAGATAAGTGTTGGTATAAAATAGGATCTCCTCCTCCGATTGGATCAAAGAAAGATGTATTTAAGTTTCGGTCTGTTAATAACATAGTAATAGCTCCAGCTAAAACAGGAAGAGAAAGAAGTAATAAGATAGCTGTAATAACTACAGATCAGACAAATAAAGGTAGTCGATCTAAAGTAATTCCTGACGATCGTATATTAATTACAGTTGTAATAAAATTTACTGCCCCTAAAATTGAGGAAATTCCAGCTAAATGAAGAGAAAAAATAGCTAAATCAACAGAAGCTCCAGCATGAGCTGTTCCTGAAGAGAGAGGAGGATAAACTGTTCACCCAGTTCCTGCCCCATTTTCTACTATTGAGCTTGATAATAGAAGAGTCAATGAAGGAGGTAGTATTCAAAAACTTATATTATTTATTCGAGGAAAGGCTATATCAGGGGCTCCTAATATTAAAGGAACTAATCAATTTACCAAATTCCCCCCCA

>LA_TT_23F6

ATCAGAATAAGTGTTGGTATAAAATAGGATCTCCTCCTCCGATTGGATCAAAGAAAGATGTATTTAAGTTTCGGTCTGTTAATAACATAGTAATAGCTCCAGCTAAAACAGGAAGAGAAAGAAGTAATAAGATAGCTGTAATAACTACAGATCAAACAAATAAAGGTAGTCGATCTAAAGTAATTCCTGACGATCGTATATTAATTACAGTTGTAATAAAATTTACTGCCCCTAAAATTGAGGAAATTCCAGCTAAATGAAGAGAAAAAATAGCTAAATCAACAGAAGCTCCAGCATGAGCTGTTCCTGAAGAGAGAGGAGGATAAACTGTTCACCCAGTTCCTGCCCCATTTTCTACTATTGAGCTTGATAATAGAAGAGTCAATGAAGGAGGTAGTATTCAAAAACTTATATTATTTATTCGAGGAAAGGCTATATCAGGGGCTCCTAATATTAAAGGAACTAATCAATTTCCCAAATTCCCTCCCA

>LA_TT_23F7

ACCTAATTCCGYMAAAATAGGATCTCCTCCTCCGATTGGATCAAAGAAAGATGTATTTAAGTTTCGGTCTGTTAATAACATAGTAATAGCTCCAGCTAAAACAGGAAGAGAAAGAAGTAATAAGATAGCTGTAATAACTACAGATCAAACAAATAAAGGTAGTCGATCTAAAGTAATTCCTGACGATCGTATATTAATTACAGTTGTAATAAAATTTACTGCCCCTAAAATTGAGGAAATTCCAGCTAAATGAAGAGAAAAAATAGCTAAATCAACAGAAGCTCCAGCATGAGCTGTTCCTGAAGAGAGAGGAGGATAAACTGTTCACCCAGTTCCTGCTCCATTTTCTACTATTGAGCTTGATAATAGAAGAGTCAATGAAGGAGGTAGTATTCAAAAACTTATATTATTTATTCGAGGAAAGGCTATATCAGGGGCTCCTAATATTAAAGGAACTAATCAATTTACAAATTCCCTCCCA

>LA_TT_23F8

TAGTTGGTATAAAATAGGATCTCCTCCTCCGATTGGATCAAAGAAAGATGTATTTAAGTTTCGGTCTGTTAATAACATAGTAATAGCTCCAGCTAAAACAGGAAGAGAAAGAAGTAATAAGATAGCTGTAATAACTACAGATCAAACAAATAAAGGTAGTCGATCTAAAGTAATTCCTGACGATCGTATATTAATTACAGTTGTAATAAAATTTACTGCCCCTAAAATTGAGGAAATTCCAGCTAAATGAAGAGAAAAAATAGCTAAATCAACAGAAGCTCCAGCATGAGCTGTTCCTGAAGAGAGAGGAGGATAAACTGTTCACCCAGTTCCTGCTCCATTTTCTACTATTGAGCTTGATAATAGAAGAGTCAATGAAGGAGGTAGTATTCAAAAACTTATATTATTTATTCGAGGAAAGGCTATATCAGGGGCTCCTAATATTAAAGGAACTAATCAATTTCCAAAATCCTCCA

>LA_TT_23F9

GTAATCAGAATAAGTGTTGGTATAAAATAGGATCTCCTCCTCCGATTGGATCAAAGAAAGATGTATTTAAGTTTCGGTCTGTTAATAACATAGTAATAGCTCCAGCTAAAACAGGAAGAGAAAGAAGTAATAAGATAGCTGTAATAACTACAGATCAAACAAATAAAGGTAGTCGATCTAAAGTAATTCCTGACGATCGTATATTAATTACAGTTGTAATAAAATTTACTGCCCCTAAAATTGAGGAAATTCCAGCTAAATGAAGAGAAAAAATAGCTAAATCAACAGAAGCTCCAGCATGAGCTGTTCCTGAAGAGAGAGGAGGATAAACTGTTCACCCAGTTCCTGCCCCATTTTCTACTATTGAGCTTGATAATAGAAGAGTCAATGAAGGAGGTAGTATTCAAAAACTTATATTATTTATTCGAGGAAAGGCTATATCAGGGGCTCCTAATATTAAAGGAACTAATCAATTTCCAAAATTCCCTCCCA

>LA_TT_23F10

GTTGGTATAAAATAGGATCTCCTCCTCCGATTGGATCAAAGAAAGATGTATTTAAGTTTCGGTCTGTTAATAACATAGTAATAGCTCCAGCTAAAACAGGAAGAGAAAGAAGTAATAAGATAGCTGTAATAACTACAGATCAGACAAATAAAGGTAGTCGATCTAAAGTAATTCCTGACGATCGTATATTAATTACAGTTGTAATAAAATTTACTGCCCCTAAAATTGAGGAAATTCCAGCTAAATGAAGAGAAAAAATAGCTAAATCAACAGAAGCTCCAGCATGAGCTGTTCCTGAAGAGAGAGGAGGATAAACTGTTCACCCAGTTCCTGCCCCATTTTCTACTATTGAGCTTGATAATAGAAGAGTCAATGAAGGAGGTAGTATTCAAAAACTTATATTATTTATTCGAGGAAAGGCTATATCAGGGGCTCCTAATATTAAAGGAACTAATCAATTTCCAATTCCTCCCA

>LA_TT_24F1

AATCAGAATAAGTGTTGGTATAAAATAGGATCTCCTCCTCCGATTGGATCAAAGAAAGATGTATTTAAGTTTCGGTCTGTTAATAACATAGTAATAGCTCCAGCTAAAACAGGAAGAGAAAGAAGTAATAAGATAGCTGTAATAACTACAGATCAAACAAATAAAGGTAGTCGATCTAAAGTAATTCCTGACGATCGTATATTAATTACAGTTGTAATAAAATTTACTGCCCCTAAAATTGAGGAAATTCCAGCTAAATGAAGAGAAAAAATAGCTAAATCAACAGAAGCTCCAGCATGAGCTGTTCCTGAAGAGAGAGGAGGATAAACTGTTCACCCAGTTCCTGCCCCATTTTCTACTATTGAGCTTGATAATAGAAGAGTCAATGAAGGAGGTAGTATTCAAAAACTTATATTATTTATTCGAGGAAAGGCTATATCAGGGGCTCCTAATATTAAAGGAACTAATCAATTCCAAATTTCCCCCA

>LA_TT_24F2

ATCAGTTTCCTGTTCCGTATAAAATAGGATCTCCTCCTCCGATTGGATCAAAGAAAGATGTATTTAAGTTTCGGTCTGTTAATAACATAGTAATAGCTCCAGCTAAAACAGGAAGAGAAAGAAGTAATAAGATAGCTGTAATAACTACAGATCAAACAAATAAAGGTAGTCGATCTAAAGTAATTCCTGACGATCGTATATTAATTACAGTTGTAATAAAATTTACTGCCCCTAAAATTGAGGAAATTCCAGCTAAATGAAGAGAAAAAATAGCTAAATCAACAGAAGCTCCAGCATGAGCTGTTCCTGAAGAGAGAGGAGGATAAACTGTTCACCCAGTTCCTGCTCCATTTTCTACTATTGAGCTTGATAATAGAAGAGTCAATGAAGGAGGTAGTATTCAAAAACTTATATTATTTATTCGAGGAAAGGCTATATCAGGGGCTCCTAATATTAAAGGAACTAATCAATACAAAATCTCCCCCCA

>LA_TT_24F3

GAATCAGAATAAGTGTTGGTATAAAATAGGATCTCCTCCTCCGATTGGATCAAAGAAAGATGTATTTAAGTTTCGGTCTGTTAATAACATAGTAATAGCTCCAGCTAAAACAGGAAGAGAAAGAAGTAATAAGATAGCTGTAATAACTACAGATCAAACAAATAAAGGTAGTCGATCTAAAGTAATTCCTGACGATCGTATATTAATTACAGTTGTAATAAAATTTACTGCCCCTAAAATTGAGGAAATTCCAGCTAAATGAAGAGAAAAAATAGCTAAATCAACAGAAGCTCCAGCATGAGCTGTTCCTGAAGAGAGAGGAGGATAAACTGTTCACCCAGTTCCTGCCCCATTTTCTACTATTGAGCTTGATAATAGAAGAGTCAATGAAGGAGGTAGTATTCAAAAACTTATATTATTTATTCGAGGAAAGGCTATATCAGGGGCTCCTAATATTAAAGGAACTAATCAATTACCAATTTCCCCCAA

>LA_TT_24F4

GTATCAGAATAAGTGTTGGTATAAAATAGGATCTCCTCCTCCGATTGGATCAAAGAAAGATGTATTTAAGTTTCGGTCTGTTAATAATATAGTAATAGCTCCAGCTAAAACAGGAAGAGAAAGAAGTAATAAGATAGCTGTAATAACTACAGATCAAACAAATAAAGGTAGTCGATCTAAAGTAATTCCTGACGATCGCATATTAATTACAGTTGTAATAAAATTTACTGCCCCTAAAATTGAGGAAATTCCAGCTAAATGAAGAGAAAAAATAGCTAAATCAACAGAAGCTCCAGCATGAGCTGTTCCTGAAGAGAGAGGAGGATAAACTGTTCACCCAGTTCCTGCCCCATTTTCTACTATTGAGCTTGATAATAGAAGAGTCAATGAAGGAGGTAGTATTCAAAAACTTATATTATTTATTCGAGGAAAGGCTATATCAGGGGCTCCTAATATTAAAGGAACTAATCAATTCCCCAATTTCCCCACA

>LA_TT_24F5

ATCAGAATAACGTGTTCCGTATAAAATAGGATCTCCTCCTCCGATTGGATCAAAGAAAGATGTATTTAAGTTTCGGTCTGTTAATAACATAGTAATAGCTCCAGCTAAAACAGGAAGAGAAAGAAGTAATAAGATAGCTGTAATAACTACAGATCAAACAAATAAAGGTAGTCGATCTAAAGTAATTCCTGACGATCGTATATTAATTACAGTTGTAATAAAATTTACTGCCCCTAAAATTGAGGAAATTCCAGCTAAATGAAGAGAAAAAATAGCTAAATCAACAGAAGCTCCAGCATGAGCTGTTCCTGAAGAGAGAGGAGGATAAACTGTTCACCCAGTTCCTGCTCCATTTTCTACTATTGAGCTTGATAATAGAAGAGTCAATGAAGGAGGTAGTATTCAAAAACTTATATTATTTATTCGAGGAAAGGCTATATCAGGGGCTCCTAATATTAAAGGAACTAATCAATTCCAAAATTTCCCCCCA

>LA_TT_24F6

GTAATGAGAATAAGTGTTGGTATAAAATAGGATCTCCTCCTCCGATTGGATCAAAGAAAGATGTATTTAAGTTTCGGTCTGTTAATAACATAGTAATAGCTCCAGCTAAAACAGGAAGAGAAAGAAGTAATAAGATAGCTGTAATAACTACAGATCAAACAAATAAAGGTAGTCGATCTAAAGTAATTCCTGACGATCGTATATTAATTACAGTTGTAATAAAATTTACTGCCCCTAAAATTGAGGAAATTCCAGCTAAATGAAGAGAAAAAATAGCTAAATCAACAGAAGCTCCAGCATGAGCTGTTCCTGAAGAGAGAGGAGGATAAACTGTTCACCCAGTTCCTGCCCCATTTTCTACTATTGAGCTTGATAATAGAAGAGTCAATGAAGGAGGTAGTATTCAAAAACTTATATTATTTATTCGAGGAAAGGCTATATCAGGGGCTCCTAATATTAAAGGAACTAATCAATTACCCAATTTCCCCCCA

>LA_TT_24F7

TCAGAATAAGTGTTGGTATAAAATAGGATCTCCTCCTCCGATTGGATCAAAGAAAGATGTATTTAAGTTTCGGTCTGTTAATAATATAGTAATAGCTCCAGCTAAAACAGGAAGAGAAAGAAGTAATAAGATAGCTGTAATAACTACAGATCAAACAAATAAAGGTAGTCGATCTAAAGTAATTCCTGACGATCGCATATTAATTACAGTTGTAATAAAATTTACTGCCCCTAAAATTGAGGAAATTCCAGCTAAATGAAGAGAAAAAATAGCTAAATCAACAGAAGCTCCAGCATGAGCTGTTCCTGAAGAGAGAGGAGGATAAACTGTTCACCCAGTTCCTGCCCCATTTTCTACTATTGAGCTTGATAATAGAAGAGTCAATGAAGGAGGTAGTATTCAAAAACTTATATTATTTATTCGAGGAAAGGCTATATCAGGGGCTCCTAATATTAAAGGAACTAATCAATTACAAATTCCCCCCA

>LA_TT_24F8

AGAATAAGTGTTGGTATAAAATAGGATCTCCTCCTCCGATTGGATCAAAGAAAGATGTATTTAAGTTTCGGTCTGTTAATAACATAGTAATAGCTCCAGCTAAAACAGGAAGAGAAAGAAGTAATAAGATAGCTGTAATAACTACAGATCAAACAAATAAAGGTAGTCGATCTAAAGTAATTCCTGACGATCGTATATTAATTACAGTTGTAATAAAATTTACTGCCCCTAAAATTGAGGAAATTCCAGCTAAATGAAGAGAAAAAATAGCTAAATCAACAGAAGCTCCAGCATGAGCTGTTCCTGAAGAGAGAGGAGGATAAACTGTTCATCCAGTTCCTGCCCCATTTTCTACTATTGAGCTTGATAATAGAAGAGTCAATGAAGGAGGTAGTATTCAAAAACTTATATTATTTATTCGAGGAAAGGCTATATCAGGGGCTCCTAATATTAAAGGAACTAATCAATTCCCAATTTCCCCCA

>LA_TT_24F9

AATCAGAATAAGTGTTGGTATAAAATAGGATCTCCTCCTCCGATTGGATCAAAGAAAGATGTATTTAAGTTTCGGTCTGTTAATAACATAGTAATAGCTCCAGCTAAAACAGGAAGAGAAAGAAGTAATAAGATAGCTGTAATAACTACAGATCAAACAAATAAAGGTAGTCGATCTAAAGTAATTCCTGACGATCGTATATTAATTACAGTTGTAATAAAATTTACTGCCCCTAAAATTGAGGAAATTCCAGCTAAATGAAGAGAAAAAATAGCTAAATCAACAGAAGCTCCAGCATGAGCTGTTCCTGAAGAGAGAGGAGGATAAACTGTTCATCCAGTTCCTGCCCCATTTTCTACTATTGAGCTTGATAATAGAAGAGTCAATGAAGGAGGTAGTATTCAAAAACTTATATTATTTATTCGAGGAAAGGCTATATCAGGGGCTCCTAATATTAAAGGAACTAATCATTCCCAAATTCCCCCCCA

>LA_TT_24F10

GTATCAGATAAGTGTTGGTATAAAATAGGATCTCCTCCTCCGATTGGATCAAAGAAAGATGTATTTAAGTTTCGGTCTGTTAATAATATAGTAATAGCTCCAGCTAAAACAGGAAGAGAAAGAAGTAATAAGATAGCTGTAATAACTACAGATCAAACAAATAAAGGTAGTCGATCTAAAGTAATTCCTGACGATCGCATATTAATTACAGTTGTAATAAAATTTACTGCCCCTAAAATTGAGGAAATTCCAGCTAAATGAAGAGAAAAAATAGCTAAATCAACAGAAGCTCCAGCATGAGCTGTTCCTGAAGAGAGAGGAGGATAAACTGTTCACCCAGTTCCTGCCCCATTTTCTACTATTGAGCTTGATAATAGAAGAGTCAATGAAGGAGGTAGTATTCAAAAACTTATATTATTTATTCGAGGAAAGGCTATATCAGGGGCTCCTAATATTAAAGGAACTAATCAACCCCAAATTTCCCCCCCA

>LA_TT_25F1

GTATCAAGAATTAGTGTTGGTATAAAATAGGATCTCCTCCTCCGATTGGATCAAAGAAAGATGTATTTAAGTTTCGGTCTGTTAATAACATAGTAATAGCTCCAGCTAAAACAGGAAGAGAAAGAAGTAATAAGATAGCTGTAATAACTACAGATCAGACAAATAAAGGTAGTCGATCTAAAGTAATTCCTGACGATCGTATATTAATTACAGTTGTAATAAAATTTACTGCCCCTAAAATTGAGGAAATTCCAGCTAAATGAAGAGAAAAAATAGCTAAATCAACAGAAGCTCCAGCATGAGCTGTTCCTGAAGAGAGAGGAGGATAAACTGTTCACCCAGTTCCTGCCCCATTTTCTACTATTGAGCTTGATAATAGAAGAGTCAATGAAGGAGGTAGTATTCAAAAACTTATATTATTTATTCGAGGAAAGGCTATATCAGGGGCTCCTAATATTAAAGGAACTAATCAATTCACAATATCCCCAATA

>LA_TT_25F2

GTAATCAGAATAAGTGTTGGTATAAAATAGGATCTCCTCCTCCGATTGGATCAAAGAAAGATGTATTTAAGTTTCGGTCTGTTAATAACATAGTAATAGCTCCAGCTAAAACAGGAAGAGAAAGAAGTAATAAGATAGCTGTAATAACTACAGATCAAACAAATAAAGGTAGTCGATCTAAAGTAATTCCTGACGATCGTATATTAATTACAGTTGTAATAAAATTTACTGCCCCTAAAATTGAGGAAATTCCAGCTAAATGAAGAGAAAAAATAGCTAAATCAACAGAAGCTCCAGCATGAGCTGTTCCTGAAGAGAGAGGAGGATAAACTGTTCACCCAGTTCCTGCCCCATTTTCTACTATTGAGCTTGATAATAGAAGAGTCAATGAAGGAGGTAGTATTCAAAAACTTATATTATTTATTCGAGGAAAGGCTATATCAGGGGCTCCTAATATTAAAGGAACTAATCAATTTCCAAATTTCCCCCGA

>LA_TT_25F3

GTTGGTATAAAATAGGATCTCCTCCTCCGATTGGATCAAAGAAAGATGTATTTAAGTTTCGGTCTGTTAATAACATAGTAATAGCTCCAGCTAAAACAGGAAGAGAAAGAAGTAATAAGATAGCTGTAATAACTACAGATCAAACAAATAAAGGTAGTCGATCTAAAGTAATTCCTGACGATCGTATATTAATTACAGTTGTAATAAAATTTACTGCCCCTAAAATTGAGGAAATTCCAGCTAAATGAAGAGAAAAAATAGCTAAATCAACAGAAGCTCCAGCATGAGCTGTTCCTGAAGAGAGAGGAGGATAAACTGTTCACCCAGTTCCTGCTCCATTTTCTACTATTGAGCTTGATAATAGAAGAGTCAATGAAGGAGGTAGTATTCAAAAACTTATATTATTTATTCGAGGAAAGGCTATATCAGGGGCTCCTAATATTAAAGGAACTAATCAATCACCAAATTTCCCCC

>LA_TT_25F4

ATCAGAATAAGTGTTGGTATAAAATAGGATCTCCTCCTCCGATTGGATCAAAGAAAGATGTATTTAAGTTTCGGTCTGTTAATAACATAGTAATAGCTCCAGCTAAAACAGGAAGAGAAAGAAGTAATAAGATAGCTGTAATAACTACAGATCAAACAAATAAAGGTAGTCGATCTAAAGTAATTCCTGACGATCGTATATTAATTACAGTTGTAATAAAATTTACTGCCCCTAAAATTGAGGAAATTCCAGCTAAATGAAGAGAAAAAATAGCTAAATCAACAGAAGCTCCAGCATGAGCTGTTCCTGAAGAGAGAGGAGGATAAACTGTTCACCCAGTTCCTGCCCCATTTTCTACTATTGAGCTTGATAATAGAAGAGTCAATGAAGGAGGTAGTATTCAAAAACTTATATTATTTATTCGAGGAAAGGCTATATCAGGGGCTCCTAATATTAAAGGAACTAATCAATCACAAAATTTCCCCCCA

>LA_TT_25F5

ATCAGAATAAGTGTTGGTATAAAATAGGATCTCCTCCTCCGATTGGATCAAAGAAAGATGTATTTAAGTTTCGGTCTGTTAATAACATAGTAATAGCTCCAGCTAAAACAGGAAGAGAAAGAAGTAATAAGATAGCTGTAATAACTACAGATCAGACAAATAAAGGTAGTCGATCTAAAGTAATTCCTGACGATCGTATATTAATTACAGTTGTAATAAAATTTACTGCCCCTAAAATTGAGGAAATTCCAGCTAAATGAAGAGAAAAAATAGCTAAATCAACAGAAGCTCCAGCATGAGCTGTTCCTGAAGAGAGAGGAGGATAAACTGTTCACCCAGTTCCTGCCCCATTTTCTACTATTGAGCTTGATAATAGAAGAGTCAATGAAGGAGGTAGTATTCAAAAACTTATATTATTTATTCGAGGAAAGGCTATATCAGGGGCTCCTAATATTAAAGGAACTAATCAATTTCAAAATTTCCCCCCA

>LA_TT_25F6

GAATCAGAATAAGTGTTGGTATAAAATAGGATCTCCTCCTCCGATTGGATCAAAGAAAGATGTATTTAAGTTTCGGTCTGTTAATAACATAGTAATAGCTCCAGCTAAAACAGGAAGAGAAAGAAGTAATAAGATAGCTGTAATAACTACAGATCAAACAAATAAAGGTAGTCGATCTAAAGTAATTCCTGACGATCGTATATTAATTACAGTTGTAATAAAATTTACTGCCCCTAAAATTGAGGAAATTCCAGCTAAATGAAGAGAAAAAATAGCTAAATCAACAGAAGCTCCAGCATGAGCTGTTCCTGAAGAGAGAGGAGGATAAACTGTTCACCCAGTTCCTGCCCCATTTTCTACTATTGAGCTCGATAATAGAAGAGTCAATGAAGGAGGTAGTATTCAAAAACTTATATTATTTATTCGAGGAAAGGCTATATCAGGGGCTCCTAATATTAAAGGAACTAATCAACTCCAAATTTCCCCCCA

>LA_TT_25F7

AGAATAAGTGTTGGTATAAAATAGGATCTCCTCCTCCGATTGGATCAAAGAAAGATGTATTTAAGTTTCGGTCTGTTAATAACATAGTAATAGCTCCAGCTAAAACAGGAAGAGAAAGAAGTAATAAGATAGCTGTAATAACTACAGATCAAACAAATAAAGGTAGTCGATCTAAAGTAATTCCTGACGATCGTATATTAATTACAGTTGTAATAAAATTTACTGCCCCTAAAATTGAGGAAATTCCAGCTAAATGAAGAGAAAAAATAGCTAAATCAACAGAAGCTCCAGCATGAGCTGTTCCTGAAGAGAGAGGAGGATAAACTGTTCACCCAGTTCCTGCTCCATTTTCTACTATTGAGCTTGATAATAGAAGAGTCAATGAAGGAGGTAGTATTCAAAAACTTATATTATTTATTCGAGGAAAGGCTATATCAGGGGCTCCTAATATTAAAGGAACTAATCAATTTCAAATTTCCCCCCA

>LA_TT_25F8

AATCAGAATAAGTGTTGGTATAAAATAGGATCTCCTCCTCCGATTGGATCAAAGAAAGATGTATTTAAGTTTCGGTCTGTTAATAACATAGTAATAGCTCCAGCTAAAACAGGAAGAGAAAGAAGTAATAAGATAGCTGTAATAACTACAGATCAAACAAATAAAGGTAGTCGATCTAAAGTAATTCCTGACGATCGTATATTAATTACAGTTGTAATAAAATTTACTGCCCCTAAAATTGAGGAAATTCCAGCTAAATGAAGAGAAAAAATAGCTAAATCAACAGAAGCTCCAGCATGAGCTGTTCCTGAAGAGAGAGGAGGATAAACTGTTCACCCAGTTCCTGCTCCATTTTCTACTATTGAGCTTGATAATAGAAGAGTCAATGAAGGAGGTAGTATTCAAAAACTTATATTATTTATTCGAGGAAAGGCTATATCAGGGGCTCCTAATATTAAAGGAACTAATCAATTTCCAAATTTCCCCCCCA

>LA_TT_25F9

TCAGAATAAGTGTTGGTATAAAATAGGATCTCCTCCTCCGATTGGATCAAAGAAAGATGTATTTAAGTTTCGGTCTGTTAATAACATAGTAATAGCTCCAGCTAAAACAGGAAGAGAAAGAAGTAATAAGATAGCTGTAATAACTACAGATCAAACAAATAAAGGTAGTCGATCTAAAGTAATTCCTGACGATCGTATATTAATTACAGTTGTAATAAAATTTACTGCCCCTAAAATTGAGGAAATTCCAGCTAAATGAAGAGAAAAAATAGCTAAATCAACAGAAGCTCCAGCATGAGCTGTTCCTGAAGAGAGAGGAGGATAAACTGTTCACCCAGTTCCTGCCCCATTTTCTACTATTGAGCTTGATAATAGAAGAGTCAATGAAGGAGGTAGTATTCAAAAACTTATATTATTTATTCGAGGAAAGGCTATATCAGGGGCTCCTAATATTAAAGGAACTAATCAAAAACAAAATTCCCCCCCA

>LA_TT_25F10

TCAGAATAACCTGTTCCGTATAAAATAGGATCTCCTCCTCCGATTGGATCAAAGAAAGATGTATTTAAGTTTCGGTCTGTTAATAACATAGTAATAGCTCCAGCTAAAACAGGAAGAGAAAGAAGTAATAAGATAGCTGTAATAACTACAGATCAAACAAATAAAGGTAGTCGATCTAAAGTAATTCCTGACGATCGTATATTAATTACAGTTGTAATAAAATTTACTGCCCCTAAAATTGAGGAAATTCCAGCTAAATGAAGAGAAAAAATAGCTAAATCAACAGAAGCTCCAGCATGAGCTGTTCCTGAAGAGAGAGGAGGATAAACTGTTCACCCAGTTCCTGCCCCATTTTCTACTATTGAGCTTGATAATAGAAGAGTCAATGAAGGAGGTAGTATTCAAAAACTTATATTATTTATTCGAGGAAAGGCTATATCAGGGGCTCCTAATATTAAAGGAACTAATCAATTCCAAAATTCCCCCA

>LA_TT_26F1

GTTCGCGTATAAAATAGGATCTCCTCCTCCGATTGGATCAAAGAAAGATGTATTTAAGTTTCGGTCTGTTAATAACATAGTAATAGCTCCAGCTAAAACAGGAAGAGAAAGAAGTAATAAGATAGCTGTAATAACTACAGATCAAACAAATAAAGGTAGTCGATCTAAAGTAATTCCTGACGATCGTATATTAATTACAGTTGTAATAAAATTTACTGCCCCTAAAATTGAGGAAATTCCAGCTAAATGAAGAGAAAAAATAGCTAAATCAACAGAAGCTCCAGCATGAGCTGTTCCTGAAGAGAGAGGAGGATAAACTGTTCACCCAGTTCCTGCTCCATTTTCTACTATTGAGCTTGATAATAGAAGAGTCAATGAAGGAGGTAGTATTCAAAAACTTATATTATTTATTCGAGGAAAGGCTATATCAGGGGCTCCTAATATTAAAGGAACTAATCACTTTCAATTTCCCCCCA

>LA_TT_26F2

TATCAGAATTACCTAATTCCGTATAAAATAGGATCTCCTCCTCCGATTGGATCAAAGAAAGATGTATTTAAGTTTCGGTCTGTTAATAACATAGTAATAGCTCCAGCTAAAACAGGAAGAGAAAGAAGTAATAAGATAGCTGTAATAACTACAGATCAAACAAATAAAGGTAGTCGATCTAAAGTAATTCCTGACGATCGTATATTAATTACAGTTGTAATAAAATTTACTGCCCCTAAAATTGAGGAAATTCCAGCTAAATGAAGAGAAAAAATAGCTAAATCAACAGAAGCTCCAGCATGAGCTGTTCCTGAAGAGAGAGGAGGATAAACTGTTCACCCAGTTCCTGCTCCATTTTCTACTATTGAGCTTGATAATAGAAGAGTCAATGAAGGAGGTAGTATTCAAAAACTTATATTATTTATTCGAGGAAAGGCTATATCAGGGGCTCCTAATATTAAAGGAACTAATCAATTTCCAAAATCCCCCCCA

>LA_TT_26F3

ATCAGAATAAGTGTTGGTATAAAATAGGATCTCCTCCTCCGATTGGATCAAAGAAAGATGTATTTAAGTTTCGGTCTGTTAATAACATAGTAATAGCTCCAGCTAAAACAGGAAGAGAAAGAAGTAATAAGATAGCTGTAATAACTACAGATCAGACAAATAAAGGTAGTCGATCTAAAGTAATTCCTGACGATCGTATATTAATTACAGTTGTAATAAAATTTACTGCCCCTAAAATTGAGGAAATTCCAGCTAAATGAAGAGAAAAAATAGCTAAATCAACAGAAGCTCCAGCATGAGCTGTTCCTGAAGAGAGAGGAGGATAAACTGTTCACCCAGTTCCTGCCCCATTTTCTACTATTGAGCTTGATAATAGAAGAGTCAATGAAGGAGGTAGTATTCAAAAACTTATATTATTTATTCGAGGAAAGGCTATATCAGGGGCTCCTAATATTAAAGGAACTAATCAATTTCAAAAATCCCCCCCA

>LA_TT_26F4

ATCAGAATAAGTGTTGGTATAAAATAGGATCTCCTCCTCCGATTGGATCAAAGAAAGATGTATTTAAGTTTCGGTCTGTTAATAACATAGTAATAGCTCCAGCTAAAACAGGAAGAGAAAGAAGTAATAAGATAGCTGTAATAACTACAGATCAAACAAATAAAGGTAGTCGATCTAAAGTAATTCCTGACGATCGTATATTAATTACAGTTGTAATAAAATTTACTGCCCCTAAAATTGAGGAAATTCCAGCTAAATGAAGAGAAAAAATAGCTAAATCAACAGAAGCTCCAGCATGAGCTGTTCCTGAAGAGAGAGGAGGATAAACTGTTCACCCAGTTCCTGCCCCATTTTCTACTATTGAGCTTGATAATAGAAGAGTCAATGAAGGAGGTAGTATTCAAAAACTTATATTATTTATTCGAGGAAAGGCTATATCAGGGGCTCCTAATATTAAAGGAACTAATCAATTTCCAAAATTCCTCCCA

>LA_TT_26F5

AATAAGTGTTGGTATAAAATAGGATCTCCTCCTCCGATTGGATCAAAGAAAGATGTATTTAAGTTTCGGTCTGTTAATAACATAGTAATAGCTCCAGCTAAAACAGGAAGAGAAAGAAGTAATAAGATAGCTGTAATAACTACAGATCAAACAAATAAAGGTAGTCGATCTAAAGTAATTCCTGACGATCGTATATTAATTACAGTTGTAATAAAATTTACTGCCCCTAAAATTGAGGAAATTCCAGCTAAATGAAGAGAAAAAATAGCTAAATCAACAGAAGCTCCAGCATGAGCTGTTCCTGAAGAGAGAGGAGGATAAACTGTTCACCCAGTTCCTGCCCCATTTTCTACTATTGAGCTTGATAATAGAAGAGTCAATGAAGGAGGTAGTATTCAAAAACTTATATTATTTATTCGAGGAAAGGCTATATCAGGGGCTCCTAATATTAAAGGAACTAATCAATTTCCAAATTCCCTCCCA

>LA_TT_26F6

GGTATAAAATAGGATCTCCTCCTCCGATTGGATCAAAGAAAGATGTATTTAAGTTTCGGTCTGTTAATAACATAGTAATAGCTCCAGCTAAAACAGGAAGAGAAAGAAGTAATAAGATAGCTGTAATAACTACAGATCAAACAAATAAAGGTAGTCGATCTAAAGTAATTCCTGACGATCGTATATTAATTACAGTTGTAATAAAATTTACTGCCCCTAAAATTGAGGAAATTCCAGCTAAATGAAGAGAAAAAATAGCTAAATCAACAGAAGCTCCAGCATGAGCTGTTCCTGAAGAGAGAGGAGGATAAACTGTTCACCCAGTTCCTGCCCCATTTTCTACTATTGAGCTTGATAATAGAAGAGTCAATGAAGGAGGTAGTATTCAAAAACTTATATTATTTATTCGAGGAAAGGCTATATCAGGGGCTCCTAATATTAAAGGAACTAATCAATTTCCAAATTCCCTCCA

>LA_TT_26F7

AGTTCGCGTATAAAATAGGATCTCCTCCTCCGATTGGATCAAAGAAAGATGTATTTAAGTTTCGGTCTGTTAATAACATAGTAATAGCTCCAGCTAAAACAGGAAGAGAAAGAAGTAATAAGATAGCTGTAATAACTACAGATCAAACAAATAAAGGTAGTCGATCTAAAGTAATTCCTGACGATCGTATATTAATTACAGTTGTAATAAAATTTACTGCCCCTAAAATTGAGGAAATTCCAGCTAAATGAAGAGAAAAAATAGCTAAATCAACAGAAGCTCCAGCATGAGCTGTTCCTGAAGAGAGAGGAGGATAAACTGTTCACCCAGTTCCTGCCCCATTTTCTACTATTGAGCTTGATAATAGAAGAGTCAATGAAGGAGGTAGTATTCAAAAACTTATATTATTTATTCGAGGAAAGGCTATATCAGGGGCTCCTAATATTAAAGGAACTAATCAATTTCAAAATTTCCCCCCA

>LA_BT_28F1

GAATAAGTGTTGGTATAAAATAGGATCTCCTCCTCCGATTGGATCAGAGAAAGATGTATTTAAGTTTCGGTCTGTTAATAACATAGTAATAGCTCCGGCTAAAACAGGAAGAGAAAGAAGTAATAAGATAGCTGTAATAACTACAGATCAAACAAATAAAGGTAGTCGATCTAAAGTAATTCCTGACGATCGTATATTAATTACAGTTGTAATAAAATTTACTGCCCCTAAAATTGAGGAAATTCCAGCTAAATGAAGAGAAAAAATAGCTAAATCAACAGAAGCTCCAGCATGAGCTGTTCCTGAAGAGAGAGGAGGATAAACTGTTCACCCAGTTCCTGCTCCATTTTCTACTATTGAGCTTGATAATAGAAGAGTCAATGAAGGAGGTAGTATTCAAAAACTTATATTATTTATTCGAGGAAAGGCTATATCAGGGGCTCCTAATATTAAAGGAACTAATCAATTC

>LA_BT_28F2

AATGAGATAAGTGTTGGTATAAAATAGGATCTCCTCCTCCGATTGGATCAAAGAAAGATGTATTTAAGTTTCGGTCTGTTAATAACATAGTAATAGCTCCAGCTAAAACAGGAAGAGAAAGAAGTAATAAGATAGCTGTAATAACTACAGATCAAACAAATAAAGGTAGTCGATCTAAAGTAATTCCTGACGATCGTATATTAATTACAGTTGTAATAAAATTTACTGCCCCTAAAATTGAGGAAATTCCAGCTAAATGAAGAGAAAAAATAGCTAAATCAACAGAAGCTCCAGCATGAGCTGTTCCTGAAGAGAGAGGAGGATAAACTGTTCACCCAGTTCCTGCCCCATTTTCTACTATTGAGCTTGATAATAGAAGAGTCAATGAAGGAGGTAGTATTCAAAAACTTATATTATTTATTCGAGGAAAGGCTATATCAGGGGCTCCTAATATTAAAGGAACTAATCAATTTCCAATTTCCCCCCA

>LA_BT_28F3

ATCAGAATAAGTGTTGGTATAAAATAGGATCTCCTCCTCCGATTGGATCAAAGAAAGATGTATTTAAGTTTCGGTCTGTTAATAATATAGTAATAGCTCCAGCTAAAACAGGAAGAGAAAGAAGTAATAAGATAGCTGTAATAACTACAGATCAAACAAATAAAGGTAGTCGATCTAAAGTAATTCCTGACGATCGTATATTAATTACAGTTGTAATAAAATTTACTGCCCCTAAAATTGAGGAAATTCCAGCTAAATGAAGAGAAAAAATAGCTAAATCAACAGAAGCTCCAGCATGAGCTGTTCCTGAAGAGAGAGGAGGATAAACTGTTCACCCAGTTCCTGCCCCATTTTCTACTATTGAGCTTGATAATAGAAGAGTCAATGAAGGAGGTAGTATTCAAAAACTTATATTATTTATTCGAGGAAAGGCTATATCAGGGGCTCCTAATATTAAAGGAACTAATCAACTTCCAAATTCCCCCCCA

>LA_BT_28F4

ATCAGAATAAGTGTTGGTATAAAATAGGATCTCCTCCTCCGATTGGATCAAAGAAAGATGTATTTAAGTTTCGGTCTGTTAATAACATAGTAATAGCTCCAGCTAAAACAGGAAGAGAAAGAAGTAATAAGATAGCTGTAATAACTACAGATCAAACAAATAAAGGTAGTCGGTCTAAAGTAATTCCTGACGATCGTATATTAATTACAGTTGTAATAAAATTTACTGCTCCTAAAATTGAGGAAATTCCAGCTAAATGAAGAGAAAAAATAGCTAAATCAACAGAAGCTCCAGCATGAGCTGTTCCTGAAGAGAGAGGAGGATAAACTGTTCACCCAGTTCCTGCCCCATTTTCTACTATTGAGCTTGATAATAGAAGAGTCAATGAAGGAGGTAGTATTCAAAAACTTATATTATTTATTCGAGGAAAGGCTATATCAGGGGCTCCTAATATTAAAGGAACTAATCAATTCCAAAATTCCCCCCA

>LA_BT_28F5

TGTTGGTAT

AAAATAGGATCTCCTCCTCCGATTGGATCAAAGAAAGATGTATTTAAGTTTCGGTCTGTTAATAACATAGTAATAGCTCCAGCTAAAACAGGAAGAGAAAGAAGTAATAAGATAGCTGTAATAACTACAGATCAAACAAATAAAGGTAGTCGGTCTAAAGTAATTCCTGACGATCGTATATTAATTACAGTTGTAATAAAATTTACTGCCCCTAAAATTGAGGAAATTCCAGCTAAATGAAGAGAAAAAATAGCTAAATCAACAGAAGCTCCAGCATGAGCTGTTCCTGAAGAGAGAGGAGGATAAACTGTTCACCCAGTTCCTGCCCCATTTTCTACTATTGAGCTTGATAATAGAAGAGTCAATGAAGGAGGTAGTATTCAAAAACTTATATTATTTATTCGAGGAAAGGCTATATCAGGGGCTCCTAATATTAAAGGAACTAATAAATTTCCAAATTTCCTCCA

>LA_BT_28F6

TCTGGGGTCAGATCAGATAAGTGTTGGTATAAAATAGGATCTCCTCCTCCGATTGGATCAAAGAAAGATGTATTTAAGTTTCGGTCTGTTAATAACATAGTAATAGCTCCAGCTAAAACAGGAAGAGAAAGAAGTAATAAGATAGCTGTAATAACTACAGATCAAACAAATAAAGGTAGTCGATCTAAAGTAATTCCTGACGATCGTATATTAATTACAGTTGTAATAAAATTTACTGCCCCTAAAATTGAGGAAATTCCAGCTAAATGAAGAGAAAAAATAGCTAAATCAACAGAAGCTCCAGCATGAGCTGTTCCTGAAGAGAGAGGAGGATAAACTGTTCACCCAGTTCCTGCCCCATTTTCTACTATTGAGCTTGATAATAGAAGAGTCAATGAAGGAGGTAGTATTCAAAAACTTATATTATTTATTCGAGGAAAGGCTATATCAGGGGCTCCTAATATTAAAGGAACTAATCAATTTCCAAATTCCTCACA

>LA_BT_28F7

GTGGGGGCAAGGATCAGATAAGTGTTGGTATAAAATAGGATCTCCTCCTCCGATTGGATCAAAGAAAGATGTATTTAAGTTTCGGTCTGTTAATAATATAGTAATAGCTCCAGCTAAAACAGGAAGAGAAAGAAGTAATAAGATAGCTGTAATAACTACAGATCAAACAAATAAAGGTAGTCGATCTAAAGTAATTCCTGACGATCGTATATTAATTACAGTTGTAATAAAATTTACTGCCCCTAAAATTGAGGAAATTCCAGCTAAATGAAGAGAAAAAATAGCTAAATCAACAGAAGCTCCAGCATGAGCTGTTCCTGAAGAGAGAGGAGGATAAACTGTTCACCCAGTTCCTGCCCCATTTTCTACTATTGAGCTTGATAATAGAAGAGTCAATGAAGGAGGTAGTATTCAAAAACTTATATTATTTATTCGAGGAAAGGCTATATSAGGGGCTCCTAATATTAAAGGAACTAATCAATTTCCAAAACCCTCCGAGA

>LA_BT_28F8

CGGGGGGTCAGATCAGATAAGTGTTGGTATAAATAGGATCTCCTCCTCCGATTGGATCAAAGAAAGATGTATTTAAGTTTCGGTCTGTTAATAACATAGTAATAGCTCCMGCTAAAACAGGAAGAGAAAGAAGTAATAAGATAGCTGTAATAACTACAGATCAAACAAATAAAGGTAGTCGGTCTAAAGTAATTCCTGACGATCGTATATTAATTACAGTTGTAATAAAATTTACTGCCCCTAAAATTGAGGAAATTCCAGCTAAATGAAGAGAAAAAATAGCTTAATCAACAGAAGCTCCAGCATGAGCTGTTCCTGAAGAGAGAGGAGGATAAACTGTTCACCCAGTTCCTGCCCCATTTTCTACTATTGAGCTTGATAATAGAAGAGTCAATGAAGGAGGTAGTATTCAAAAACTTATATTATTTATTCGAGGAAAGGCTATATCAGGGGCTCCTAATATTAAAGGAACTAATCAATTTCCAAATCCTCCGAGA

>LA_BT_28F9

GMTGGGGTCAGGATCAGATAAGTGTTGGTATAAAATAGGATCTCCTCCTCCGATTGGATCAAAGAAAGATGTATTTAAGTTTCGGTCTGTTAATAACATAGTAATAGCTCCAGCTAAAACAGGAAGAGAAAGAAGTAATAAGATAGCTGTAATAACTACAGATCAAACAAATAAAGGTAGTCGATCTAAAGTAATTCCTGACGATCGTATATTAATTACAGTTGTAATAAAATTTACTGCCCCTAAAATTGAGGAAATTCCAGCTAAATGAAGAGAAAAAATAGCTAAATCAACAGAAGCTCCAGCATGAGCTGTTCCTGAAGAGAGAGGAGGATAAACTGTTCACCCAGTTCCTGCCCCATTTTCTACTATTGAGCTTGATAATAGAAGAGTCAATGAAGGAGGTAGTATTCAAAAACTTATATTATTTATTCGAGGAAAGGCTATATCAGGGGCTCCTAATATTAAAGGAACTAATCAATTACAAATTCCCCCCCACA

>LA_BT_28F10

GGTGGGGTCAGATCAGATAAGTGTTGGTATAAAATAGGATCTCCTCCTCCGATTGGATCAAAGAAAGATGTATTTAAGTTTCGGTCTGTTAATAACATAGTAATAGCTCCAGCTAAAACAGGAAGAGAAAGAAGTAATAAGATAGCTGTAATAACTACAGATCAAACAAATAAAGGTAGTCGATCTAAAGTAATTCCTGACGATCGTATATTAATTACAGTTGTAATAAAATTTACTGCCCCTAAAATTGAGGAAATTCCAGCTAAATGAAGAGAAAAAATAGCTAAATCAACAGAAGCTCCAGCATGAGCTGTTCCTGAAGAGAGAGGAGGATAAACTGTTCACCCAGTTCCTGCTCCATTTTCTACTATTGAGCTTGATAATAGAAGAGTCAATGAAGGAGGTAGTATTCAAAAACTTATATTATTTATTCGAGGAAAGGCTATATCAGGGGCTCCTAATATTAAAGGAACTAATCAATTTCCAAATTCCCTCCTAGCA

>LA_BT_29F1

GCTGCGTCAGTATCAGATAAGTGTTCCGTATAAAATAGGATCTCCTCCTCCGATTGGATCAAAGAAAGATGTATTTAAGTTTCGGTCTGTTAATAACATAGTAATAGCTCCAGCTAAAACAGGAAGAGAAAGAAGTAATAAGATAGCTGTAATAACTACAGATCAAACAAATAAAGGTAGTCGATCTAAAGTAATTCCTGACGATCGTATATTAATTACAGTTGTAATAAAATTTACTGCCCCTAAAATTGAGGAAATTCCAGCTAAATGAAGAGAAAAAATAGCTAAATCAACAGAAGCTCCAGCATGAGCTGTTCCTGAAGAGAGAGGAGGATAAACTGTTCACCCAGTTCCTGCCCCATTTTCTACTATTGAGCTTGATAATAGAAGAGTCAATGAAGGAGGTAGTATTCAAAAACTTATATTATTTATTCGAGGAAAGGCTATATCAGGGGCTCCTAATATTAAAGGAACTAATCAAAAACAAAATTCCCTCCCACA

>LA_BT_29F2

TGGGGTCAGTATCAGATAAGTGTTGGTATAAAATAGGATCTCCTCCTCCGATTGGATCAAAGAAAGATGTATTTAAGTTTCGGTCTGTTAATAATATAGTAATAGCTCCAGCTAAAACAGGAAGAGAAAGAAGTAATAAGATAGCTGTAATAACTACAGATCAAACAAATAAAGGTAGTCGATCTAAAGTAATTCCTGACGATCGTATATTAATTACAGTTGTAATAAAATTTACTGCCCCTAAAATTGAGGAAATTCCAGCTAAATGAAGAGAAAAAATAGCTAAATCAACAGAAGCTCCAGCATGAGCTGTTCCTGAAGAGAGAGGAGGATAAACTGTTCACCCAGTTCCTGCCCCATTTTCTACTATTGAGCTTGATAATAGAAGAGTCAATGAAGGAGGTAGTATTCAAAAACTTATATTATTTATTCGAGGAAAGGCTATATCAGGGGCTCCTAATATTAAAGGAACTAATCAATACCAAATTCCCTCCCATAGT

>LA_BT_29F3

GAGGGCAAGATCAGATGAGTGTTGGTATAAGATAGGATCTCCCCCTCCGGATTGGATCAAAGAAAGATGTATTTAAGTTTCGGTCTGTTAATAACATAGTAATAGCTCCAGCTAAGGCAGGAAGAGAAAGAAGTAATAAGATAGCTGTAATAACTACAGATCAAACAAATGAGGGTAGTCGATCTAAAGTAATTCCTGACGATCGTATATAATTACAGTTGTAATAAAATTTACTGCCCCTAAAATTGAGGAAATTCCAGCTAAATGAAGAGAAAAAATAGCTAAATCAACAGAAGCTCCAGCATGAGCTGTTCCTGAAGAGAGAGGAGGATAAACTGTTCACCCAGTTCCTGCCCCATTTTCTACTATGAGCTTGATAATAGAAGAGTCGATGAAGGAGGTAGTATTCAAAAACTTATATTATTTATTCGAGGAAAGGCGATGTCAGGGGCTCCTAAGATTAAAGGAACTAATCAATTTCCAAATCCTCCCA

>LA_BT_29F4

GGGGGGGTCAGATCAGATAAGTGTTGGTATAAAATAGGATCTCCTCCTCCGATTGGATCAAAGAAAGATGTATTTAAGTTTCGGTCTGTTAATAATATAGTAATAGCTCCAGCTAAAACAGGAAGAGAAAGAAGTAATAAGATAGCTGTAATAACTACAGATCAAACAAATAAAGGTAGTCGATCTAAAGTAATTCCTGACGATCGTATATTAATTACAGTTGTAATAAAATTTACTGCCCCTAAAATTGAGGAAATTCCAGCTAAATGAAGAGAAAAAATAGCTAAATCAACAGAAGCTCCAGCATGAGCTGTTCCTGAAGAGAGAGGAGGATAAACTGTTCACCCAGTTCCTGCCCCATTTTCTACTATTGAGCTTGATAATAGAAGAGTCAATGAAGGAGGTAGTATTCAAAAACTTATATTATTTATTCGAGGAAAGGCTATATCAGGGGCTCCTAATATTAAAGGAACTAATCAATTATAAAATTCCCTCCCACA

>LA_BT_29F5

GGGGGGTCAAGATCAGATAAGTGTTGGTATAAAATAGGATCTCCTCCTCCGATTGGATCAAAGAAAGATGTATTTAAGTTTCGGTCTGTTAATAACATAGTAATAGCTCCAGCTAAAACAGGAAGAGAAAGAAGTAATAAGATAGCTGTAATAACTACAGATCAAACAAATAAAGGTAGTCGATCTAAAGTAATTCCTGACGATCGTATATTAATTACAGTTGTAATAAAATTTACTGCCCCTAAAATTGAGGAAATTCCAGCTAAATGAAGAGAAAAAATAGCTAAATCAACAGAAGCTCCAGCATGAGCTGTTCCTGAAGAGAGAGGAGGATAAACTGTTCACCCAGTTCCTGCTCCATTTTCTACTATTGAGCTTGATAATAGAAGAGTCAATGAAGGAGGTAGTATTCAAAAACTTATATTATTTATTCGAGGAAAGGCTATATCAGGGGCTCCTAATATTAAAGGAACTAATCAATTACAAAATTCCCCCCAAGA

>LA_BT_29F6

GGGGGGTCAGATCAGATAAGTGTTGGTATAAAATAGGATCTCCTCCTCCGATTGGATCAAAGAAAGATGTATTTAAGTTTCGGTCTGTTAATAACATAGTAATAGCTCCAGCTAAAACAGGAAGAGAAAGAAGTAATAAGATAGCTGTAATAACTACAGATCAAACAAATAAAGGTAGTCGATCTAAAGTAATTCCTGACGATCGTATATTAATTACAGTTGTAATAAAATTTACTGCCCCTAAAATTGAGGAAATTCCAGCTAAATGAAGAGAAAAAATAGCTAAATCAACAGAAGCTCCAGCATGAGCTGTTCCTGAAGAGAGAGGAGGATAAACTGTTCACCCAGTTCCTGCTCCATTTTCTACTATTGAGCTTGATAATAGAAGAGTCAATGAAGGAGGTAGTATTCAAAAACTTATATTATTTATTCGAGGAAAGGCTATATCAGGGGCTCCTAATATTAAAGGAACTAATCAATCACAAAATTCCCCCCCACA

>LA_BT_29F7

GGTGGTCAGTATCAGATAAGTGTTGGTATAAAATAGGATCTCCTCCTCCGATTGGATCAAAGAAAGATGTATTTAAGTTTCGGTCTGTTAATAATATAGTAATAGCTCCAGCTAAAACAGGAAGAGAAAGAAGTAATAAGATAGCTGTAATAACTACAGATCAAACAAATAAAGGTAGTCGATCTAAAGTAATTCCTGACGATCGTATATTAATTACAGTTGTAATAAAATTTACTGCCCCTAAAATTGAGGAAATTCCAGCTAAATGAAGAGAAAAAATAGCTAAATCAACAGAAGCTCCAGCATGAGCTGTTCCTGAAGAGAGAGGAGGATAAACTGTTCACCCAGTTCCTGCCCCATTTTCTACTATTGAGCTTGATAATAGAAGAGTCAATGAAGGAGGTAGTATTCAAAAACTTATATTATTTATTCGAGGAAAGGCTATATCAGGGGCTCCTAATATTAAAGGAACTAATCAACTATAAATTCCCCCCGACA

>LA_BT_29F8

GTGGGTCAAGATCAGATAAGTGTTGGTATAAAATAGGATCTCCTCCTCCGATTGGATCAAAGAAAGATGTATTTAAGTTTCGGTCTGTTAATAACATAGTAATAGCTCCAGCTAAAACAGGAAGAGAAAGAAGTAATAAGATAGCTGTAATAACTACAGATCAAACAAATAAAGGTAGTCGATCTAAAGTAATTCCTGACGATCGTATATTAATTACAGTTGTAATAAAATTTACTGCCCCTAAAATTGAGGAAATTCCAGCTAAATGAAGAGAAAAAATAGCTAAATCAACAGAAGCTCCAGCATGAGCTGTTCCTGAAGAGAGAGGAGGATAAACTGTTCACCCAGTTCCTGCTCCATTTTCTACTATTGAGCTTGATAATAGAAGAGTCAATGAAGGAGGTAGTATTCAAAAACTTATATTATTTATTCGAGGAAAGGCTATATCAGGGGCTCCTAATATTAAAGGAACTAATCAATTTCAAAATTCCCCCCAAGA

>LA_BT_29F9

GTGGGGTCAGATCAGATAAGTGTTGGTATAAAATAGGATCTCCTCCTCCGATTGGATCAAAGAAAGATGTATTTAAGTTTCGGTCTGTTAATAACATAGTAATAGCTCCAGCTAAAACAGGAAGAGAAAGAAGTAATAAGATAGCTGTAATAACTACAGATCAAACAAATAAAGGTAGTCGATCTAAAGTAATTCCTGACGATCGTATATTAATTACAGTTGTAATAAAATTTACTGCCCCTAAAATTGAGGAAATTCCAGCTAAATGAAGAGAAAAAATAGCTAAATCAACAGAAGCTCCAGCATGAGCTGTTCCTGAAGAGAGAGGAGGATAAACTGTTCACCCAGTTCCTGCCCCATTTTCTACTATTGAGCTTGATAATAGAAGAGTCAATGAAGGAGGTAGTATTCAAAAACTTATATTATTTATTCGAGGAAAGGCTATATCAGGGGCTCCTAATATTAAAGGAACTAATCAATTTACAAATTCCCCCCCACA

>LA_BT_29F10

CTGGGTCAAGTATCAGATAGTGTTGGTATAAAATAGGATCTCCTCCTCCGATTGGATCAAAGAAAGATGTATTTAAGTTTCGGTCTGTTAATAATATAGTAATAGCTCCAGCTAAAACAGGAAGAGAAAGAAGTAATAAGATAGCTGTAATAACTACAGATCAAACAAATAAAGGTAGTCGATCTAAAGTAATTCCTGACGATCGTATATTAATTACAGTTGTAATAAAATTTACTGCCCCTAAAATTGAGGAAATTCCAGCTAAATGAAGAGAAAAAATAGCTAAATCAACAGAAGCTCCAGCATGAGCTGTTCCTGAAGAGAGAGGAGGATAAACTGTTCACCCAGTTCCTGCCCCATTTTCTACTATTGAGCTTGATAATAGAAGAGTCAATGAAGGAGGTAGTATTCAAAAACTTATATTATTTATTCGAGGAAAGGCTATATCAGGGGCTCCTAATATTAAAGGAACTAATCAATTCTACAAAATCCCTCCAACA

>LA_BT_30F1

TGGGTGCAGATCAGATAAGTGTTGGTATAAAATAGGATCTCCTCCTCCGATTGGATCAAAGAAAGATGTATTTAAGTTTCGGTCTGTTAATAATATAGTAATAGCTCCAGCTAAAACAGGAAGAGAAAGAAGTAATAAGATAGCTGTAATAACTACAGATCAAACAAATAAAGGTAGTCGATCTAAAGTAATTCCTGACGATCGTATATTAATTACAGTTGTAATAAAATTTACTGCCCCTAAAATTGAGGAAATTCCAGCTAAATGAAGAGAAAAAATAGCTAAATCAACAGAAGCTCCAGCATGAGCTGTTCCTGAAGAGAGAGGAGGATAAACTGTTCACCCAGTTCCTGCCCCATTTTCTACTATTGAGCTTGATAATAGAAGAGTCAATGAAGGAGGTAGTATTCAAAAACTTATATTATTTATTCGAGGAAAGGCTATATCAGGGGCTCCTAATATTAAAGGAACTAATCAATTTCCAAATTCCCTCCCAGA

>LA_BT_30F2

GGGGGGGTCAGATCAGATAAGTGTTGGTATAAAATAGGATCTCCTCCTCCGATTGGATCAAAGAAAGATGTATTTAAGTTTCGGTCTGTTAATAATATAGTAATAGCTCCAGCTAAAACAGGAAGAGAAAGAAGTAATAAGATAGCTGTAATAACTACAGATCAAACAAATAAAGGTAGTCGATCTAAAGTAATTCCTGACGATCGTATATTAATTACAGTTGTAATAAAATTTACTGCCCCTAAAATTGAGGAAATTCCAGCTAAATGAAGAGAAAAAATAGCTAAATCAACAGAAGCTCCAGCATGAGCTGTTCCTGAAGAGAGAGGAGGATAAACTGTTCACCCAGTTCCTGCCCCATTTTCTACTATTGAGCTTGATAATAGAAGAGTCAATGAAGGAGGTAGTATTCAAAAACTTATATTATTTATTCGAGGAAAGGCTATATCAGGGGCTCCTAATATTAAAGGAACTAATCAATTTACAAATTCCCTCCAGCA

>LA_BT_30F3

GGGGGGGCAGATCAGATAAGTGTTGGTATAAAATAGGATCTCCTCCTCCGATTGGATCAAAGAAAGATGTATTTAAGTTTCGGTCTGTTAATAATATAGTAATAGCTCCAGCTAAAACAGGAAGAGAAAGAAGTAATAAGATAGCTGTAATAACTACAGATCAAACAAATAAAGGTAGTCGATCTAAAGTAATTCCTGACGATCGTATATTAATTACAGTTGTAATAAAATTTACTGCCCCTAAAATTGAGGAAATTCCAGCTAAATGAAGAGAAAAAATAGCTAAATCAACAGAAGCTCCAGCATGAGCTGTTCCTGAAGAGAGAGGAGGATAAACTGTTCACCCAGTTCCTGCCCCATTTTCTACTATTGAGCTTGATAATAGAAGAGTCAATGAAGGAGGTAGTATTCAAAAACTTATATTATTTATTCGAGGAAAGGCTATATCAGGGGCTCCTAATATTAAAGGAACTAATCAATTTACAAATTCCCCCCAACA

>LA_BT_30F4

GTGGGTCAAGATCAGATAAGTGTTGGTATAAAATAGGGTCTCCCCCTCCGATTGGATCAAAGAAAGATGTATTTAAGTTTCGGTCTGTTAATAATATAGTAATAGCTCCAGCTAAAACAGGAAGAGAAAGAAGTAATAAGATAGCTGTAATAACTACAGATCAAACAAATAAGGGTAGTCGATCTAAAGTAATCCCTGACGATCGCATATTAATCACAGTTGTAATAAAATTTACTGCCCCTAAAATTGAGGAAATTCCAGCTAAATGAAGAGAAAAAATAGCTAAATCAACAGAAGCTCCAGCATGAGCTGTTCCTGAAGAGAGAGGAGGATAAACTGTTCACCCAGTTCCTGCCCCATTTTCTACTATTGAGCTTGATAATAGAAGAGTCAATGAAGGAGGTAGTATTCAAAAACTTATATTATTCATTCGAGGGAAAGCTATATCAGGGGCTCCTAATATTAAAGGAACTAATCAATCACAAAATTCCCTCCACAGA

>LA_BT_30F5

GTCAAGTATCAGATTCCTGTTCCGTATAAAATAGGATCTCCTCCTCCGATTGGATCAAAGAAAGATGTATTTAAGTTTCGGTCTGTTAATAACATAGTAATAGCTCCAGCTAAAACAGGAAGAGAAAGAAGTAATAAGATAGCTGTAATAACTACAGATCAAACAAATAAAGGTAGTCGGTCTAAAGTAATTCCTGACGATCGTATATTAATTACAGTTGTAATAAAATTTACTGCTCCTAAAATTGAGGAAATTCCAGCTAAATGAAGAGAAAAAATAGCTAAATCAACAGAAGCTCCAGCATGAGCTGTTCCTGAAGAGAGAGGAGGATAAACTGTTCACCCAGTTCCTGCCCCATTTTCTACTATTGAGCTTGATAATAGAAGAGTCAATGAAGGAGGTAGTATTCAAAAACTTATATTATTTATTCGAGGAAAGGCTATATCAGGGGCTCCTAATATTAAAGGAACTAATCAATTTCAAAATTCCCTCCCACA

>LA_BT_30F6

GTGGGGGTCAAGATCAGATAAGTGTTGGTATAAAATAGGATCTCCTCCTCCGATTGGATCAAAGAAAGATGTATTTAAGTTTCGGTCTGTTAATAACATAGTAATAGCTCCGGCTAAAACAGGAAGAGAAAGAAGTAATAAGATAGCTGTAATAACTACAGATCAAACAAATAAAGGTAGTCGATCTAAAGTAATTCCTGACGATCGTATATTAATTACAGTTGTAATAAAATTTACTGCCCCTAAAATTGAGGAAATTCCAGCTAAATGAAGAGAAAAAATAGCTAAATCAACAGAAGCTCCAGCATGAGCTGTTCCTGAAGAGAGAGGAGGATAAACTGTTCACCCAGTTCCTGCTCCATTTTCTACTATTGAGCTTGATAATAGAAGAGTCAATGAAGGAGGTAGTATTCAAAAACTTATATTATTTATTCGAGGAAAGGCTATATCAGGGGCTCCTAATATTAAAGGAACTAATCAATTTCAAAATTCCCCCCAAGA

>LA_BT_30F7

TTTGGGGTCAGATCAGATAAGTGTTGGTATAAAATAGGATCTCCTCCTCCGATTGGATCAAAGAAAGATGTATTTAAGTTTCGGTCTGTTAATAACATAGTAATAGCTCCAGCTAAAACAGGAAGAGAAAGAAGTAATAAGATAGCTGTAATAACTACAGATCAAACAAATAAAGGTAGTCGATCTAAAGTAATTCCTGACGATCGTATATTAATTACAGTTGTAATAAAATTTACTGCCCCTAAAATTGAGGAAATTCCAGCTAAATGAAGAGAAAAAATAGCTAAATCAACAGAAGCTCCAGCATGAGCTGTTCCTGAAGAGAGAGGAGGATAAACTGTTCACCCAGTTCCTGCCCCATTTTCTACTATTGAGCTTGATAATAGAAGAGTCAATGAAGGAGGTAGTATTCAAAAACTTATATTATTTATTCGAGGAAAGGCTATATCAGGGGCTCCTAATATTAAAGGAACTAATCAATTTCAAAATTCCCCCCCAGA

>LA_BT_30F8

CTGGGGTCAGTATCAGATAAGTGTTGGTATAAAATAGGATCTCCTCCTCCGATTGGATCAAAGAAAGATGTATTTAAGTTTCGGTCTGTTAATAATATAGTAATAGCTCCAGCTAAAACAGGAAGAGAAAGAAGTAATAAGATAGCTGTAATAACTACAGATCAAACAAATAAAGGTAGTCGATCTAAAGTAATTCCTGACGATCGTATATTAATTACAGTTGTAATAAAATTTACTGCCCCTAAAATTGAGGAAATTCCAGCTAAATGAAGAGAAAAAATAGCTAAATCAACAGAAGCTCCAGCATGAGCTGTTCCTGAAGAGAGAGGAGGATAAACTGTTCACCCAGTTCCTGCCCCATTTTCTACTATTGAGCTTGATAATAGAAGAGTCAATGAAGGAGGTAGTATTCAAAAACTTATATTATTTATTCGAGGAAAGGCTATATCAGGGGCTCCTAATATTAAAGGAACTAATCAATTTACAAAATTCCCCCCCGCA

>LA_BT_30F9

TGTGGGGTCAAGATCAGATAAGTGTTGGTATAAAATAGGATCTCCTCCTCCGATTGGATCAAAGAAAGATGTATTTAAGTTTCGGTCTGTTAATAACATAGTAATAGCTCCAGCTAAAACAGGAAGAGAAAGAAGTAATAAGATAGCTGTAATAACTACAGATCAAACAAATAAAGGTAGTCGATCTAAAGTAATTCCTGACGATCGTATATTAATTACAGTTGTAATAAAATTTACTGCCCCTAAAATTGAGGAAATTCCAGCTAAATGAAGAGAAAAAATAGCTAAATCAACAGAAGCTCCAGCATGAGCTGTTCCTGAAGAGAGAGGAGGATAAACTGTTCACCCAGTTCCTGCCCCATTTTCTACTATTGAGCTTGATAATAGAAGAGTCAATGAAGGAGGTAGTATTCAAAAACTTATATTATTTATTCGAGGAAAGGCTATATCAGGGGCTCCTAATATTAAAGGAACTAATCACTTTCAAAATTTCCCCCAAATA

>LA_BT_30F10

GCTGCCGTCAAGTTCAGTTTAGTGTTCCGTATAAAATAGGATCTCCTCCTCCGATTGGATCAAAGAAAGATGTATTTAAGTTTCGGTCTGTTAATAACATAGTAATAGCTCCAGCTAAAACAGGAAGAGAAAGAAGTAATAAGATAGCTGTAATAACTACAGATCAAACAAATAAAGGTAGTCGATCTAAAGTAATTCCTGACGATCGTATATTAATTACAGTTGTAATAAAATTTACTGCCCCTAAAATTGAGGAAATTCCAGCTAAATGAAGAGAAAAAATAGCTAAATCAACAGAAGCTCCAGCATGAGCTGTTCCTGAAGAGAGAGGAGGATAAACTGTTCACCCAGTTCCTGCTCCATTTTCTACTATTGAGCTTGATAATAGAAGAGTCAATGAAGGAGGTAGTATTCAAAAACTTATATTATTTATTCGAGGAAAGGCTATATCAGGGGCTCCTAATATTAAAGGAACTAATCAATTTAAAATCCCTCCGACAG

>LA_BT_31F1

GTGGGTCAGATCAGATAAGTGTTGGTATAAAATAGGATCTCCTCCTCCGATTGGATCAAAGAAAGATGTATTTAAGTTTCGGTCTGTTAATAACATAGTAATAGCTCCAGCTAAAACAGGAAGAGAAAGAAGTAATAAGATAGCTGTAATAACTACAGATCAAACAAATAAAGGTAGTCGATCTAAAGTAATTCCTGACGATCGTATATTAATTACAGTTGTAATAAAATTTACTGCCCCTAAAATTGAGGAAATTCCAGCTAAATGAAGAGAAAAAATAGCTAAATCAACAGAAGCTCCAGCATGAGCTGTTCCTGAAGAGAGAGGAGGATAAACTGTTCACCCAGTTCCTGCTCCATTTTCTACTATTGAGCTTGATAATAGAAGAGTCAATGAAGGAGGTAGTATTCAAAAACTTATATTATTTATTCGAGGAAAGGCTATATCAGGGGCTCCTAATATTAAAGGAACTAATCAATTTCAAATTCCCCCCAAGCA

>LA_BT_31F2

GTGGGTTCAAGATCAGATAAGTGTTGGTATAAAATAGGATCTCCTCCTCCGATTGGATCAAAGAAAGATGTATTTAAGTTTCGGTCTGTTAATAACATAGTAATAGCTCCAGCTAAAACAGGAAGAGAAAGAAGTAATAAGATAGCTGTAATAACTACAGATCAAACAAATAAAGGTAGTCGATCTAAAGTAATTCCTGACGATCGTATATTAATTACAGTTGTAATAAAATTTACTGCCCCTAAAATTGAGGAAATTCCAGCTAAATGAAGAGAAAAAATAGCTAAATCAACAGAAGCTCCAGCATGAGCTGTTCCTGAAGAGAGAGGAGGATAAACTGTTCACCCAGTTCCTGCCCCATTTTCTACTATTGAGCTTGATAATAGAAGAGTCAATGAAGGAGGTAGTATTCAAAAACTTATATTATTTATTCGAGGAAAGGCTATATCAGGGGCTCCTAATATTAAAGGAACTAATCAATTTCAAAATT

CCCCCCCAGA

>LA_BT_31F3

GTGGGGTCAGATCAGATAAGTGTTGGTATAAAATAGGGTCTCCCCCTCCGATTGGATCAAAGAAAGATGTATTTAAGTTTCGGTCTGTTAATAATATAGTAATAGCTCCAGCTAAAACAGGAAGAGAAAGAAGTAATAAGATAGCTGTAATAACTACAGATCAAACAAATAAGGGTAGTCGATCTAAAGTAATCCCTGACGATCGCATATTAATCACAGTTGTAATAAAATTTACTGCCCCTAAAATTGAGGAAATTCCAGCTAAATGAAGAGAAAAAATAGCTAAATCAACAGAAGCTCCAGCATGAGCTGTTCCTGAAGAGAGAGGAGGATAAACTGTTCACCCAGTTCCTGCCCCATTTTCTACTATTGAGCTTGATAATAGAAGAGTCAATGAAGGAGGTAGTATTCAAAAACTTATATTATTCATTCGAGGGAAAGCTATATCAGGGGCTCCTAATATTAAAGGAACTAATCAATTTCCAAAATTCCCCCCCACAGG

>LA_BT_31F4

ACGGTCAAGTATCAGATTACCTGTTCCGTATAAAATAGGATCTCCCCCTCCGATTGGATCAAAGAAAGATGTATTTAAGTTTCGGTCTGTTAATAACATAGTAATAGCTCCAGCTAAAACAGGAAGAGAAAGAAGTAATAAGATAGCTGTAATAACTACAGATCAAACAAATAAAGGTAGTCGATCTAAAGTAATTCCTGACGATCGTATATTAATTACAGTTGTAATAAAATTTACTGCCCCTAAAATTGAGGAAATTCCAGCTAAATGAAGAGAAAAAATAGCTAAATCAACAGAAGCTCCAGCATGAGCTGTTCCTGAAGAGAGAGGAGGATAAACTGTTCACCCAGTTCCTGCCCCATTTTCTACTATTGAGCTTGATAATAGAAGAGTCAATGAAGGAGGTAGTATTCAAAAACTTATATTATTTATTCGAGGAAAGGCTATATCAGGGGCTCCTAATATTAAAGGAACTAATCAATTTACCAAATTCCCTCCCA

>LA_BT_31F5

CTGGGGGTCAGATCAGATAAGTGTTGGTATAAAATAGGATCTCCTCCTCCGATTGGATCAAAGAAAGATGTATTTAAGTTTCGGTCTGTTAATAACATAGTAATAGCTCCAGCTAAAACAGGAAGAGAAAGAAGTAATAAGATAGCTGTAATAACTACAGATCAAACAAATAAAGGTAGTCGATCTAAAGTAATTCCTGACGATCGTATATTAATTACAGTTGTAATAAAATTTACTGCCCCTAAAATTGAGGAAATTCCAGCTAAATGAAGAGAAAAAATAGCTAAATCAACAGAAGCTCCAGCATGAGCTGTTCCTGAAGAGAGAGGAGGATAAACTGTTCACCCAGTTCCTGCCCCATTTTCTACTATTGAGCTTGATAATAGAAGAGTCAATGAAGGAGGTAGTATTCAAAAACTTATATTATTTATTCGAGGAAAGGCTATATCAGGGGCTCCTAATATTAAAGGAACTAATCAATTTACAAATTCCCCCCCACA

>LA_BT_31F6

GTGGGGCAGATCAGATAAGTGTTGGTATAAAATAGGATCTCCTCCTCCGATTGGATCAAAGAAAGATGTATTTAAGTTTCGGTCTGTTAATAATATAGTAATAGCTCCAGCTAAAACAGGAAGAGAAAGAAGTAATAAGATAGCTGTAATAACTACAGATCAAACAAATAAAGGTAGTCGATCTAAAGTAATTCCTGACGATCGTATATTAATTACAGTTGTAATAAAATTTACTGCCCCTAAAATTGAGGAAATTCCAGCTAAATGAAGAGAAAAAATAGCTAAATCAACAGAAGCTCCAGCATGAGCTGTTCCTGAAGAGAGAGGAGGATAAACTGTTCACCCAGTTCCTGCCCCATTTTCTACTATTGAGCTTGATAATAGAAGAGTCAATGAAGGAGGTAGTATTCAAAAACTTATATTATTTATTCGAGGAAAGGCTATATCAGGGGCTCCTAATATTAAAGGAACTAATCAATTTCAAAATTCCCCCCAAGA

>LA_BT_31F7

GGTAGGGTCAAGATCAGATAAGTGTTGGTATAAAATAGGATCTCCTCCTCCGATTGGATCAAAGAAAGATGTATTTAAGTTTCGGTCTGTTAATAACATAGTAATAGCTCCAGCTAAAACAGGAAGAGAAAGAAGTAATAAGATAGCTGTAATAACTACAGATCAAACAAATAAAGGTAGTCGATCTAAAGTAATTCCTGACGATCGTATATTAATTACAGTTGTAATAAAATTTACTGCCCCTAAAATTGAGGAAATTCCAGCTAAATGAAGAGAAAAAATAGCTAAATCAACAGAAGCTCCAGCATGAGCTGTTCCTGAAGAGAGAGGAGGATAAACTGTTCACCCAGTTCCTGCCCCATTTTCTACTATTGAGCTCGATAATAGAAGAGTCAATGAAGGAGGTAGTATTCAAAAACTTATATTATTTATTCGAGGAAAGGCTATATCAGGGGCTCCTAATATTAAAGGAACTAATCAATTTCAAATTCCCCCACAGA

>LA_BT_31F8

GTGGGTCAAGATCAGATAAGTGTTGGTATAAAATAGGATCTCCTCCTCCGATTGGATCAAAGAAAGATGTATTTAAGTTTCGGTCTGTTAATAACATAGTAATAGCTCCAGCTAAAACAGGAAGAGAAAGAAGTAATAAGATAGCTGTAATAACTACAGATCAAACAAATAAAGGTAGTCGATCTAAAGTAATTCCTGACGATCGTATATTAATTACAGTTGTAATAAAATTTACTGCCCCTAAAATTGAGGAAATTCCAGCTAAATGAAGAGAAAAAATAGCTAAATCAACAGAAGCTCCAGCATGAGCTGTTCCTGAAGAGAGAGGAGGATAAACTGTTCACCCAGTTCCTGCCCCATTTTCTACTATTGAGCTTGATAATAGAAGAGTCAATGAAGGAGGTAGTATTCAAAAACTTATATTATTTATTCGAGGAAAGGCTATATCAGGGGCTCCTAATATTAAAGGAACTAATCAATTTCAAAATCTCCCCCCCAAAA

>LA_BT_31F9

GTGGGTCAAGATCAGATAAGTGTTGGTATAAAATAGGATCTCCTCCTCCGATTGGATCAAAGAAAGATGTATTTAAGTTTCGGTCTGTTAATAACATAGTAATAGCTCCAGCTAAAACAGGAAGAGAAAGAAGTAATAAGATAGCTGTAATAACTACAGATCAAACAAATAAAGGTAGTCGGTCTAAAGTAATTCCTGACGATCGTATATTAATTACAGTTGTAATAAAATTTACTGCTCCTAAAATTGAGGAAATTCCAGCTAAATGAAGAGAAAAAATAGCTAAATCAACAGAAGCTCCAGCATGAGCTGTTCCTGAAGAGAGAGGAGGATAAACTGTTCACCCAGTTCCTGCCCCATTTTCTACTATTGAGCTTGATAATAGAAGAGTCAATGAAGGAGGTAGTATTCAAAAACTTATATTATTTATTCGAGGAAAGGCTATATCAGGGGCTCCTAATATTAAAGGAACTAATCAATTTCAAAACCCTCACACAGG

>LA_BT_31F10

GTGGGGTGCAGATCAGATAAGTGTTGGTATAAAATAGGATCTCCTCCTCCGATTGGATCAAAGAAAGATGTATTTAAGTTTCGGTCTGTTAATAATATAGTAATAGCTCCAGCTAAAACAGGAAGAGAAAGAAGTAATAAGATAGCTGTAATAACTACAGATCAAACAAATAAAGGTAGTCGATCTAAAGTAATTCCTGACGATCGTATATTAATTACAGTTGTAATAAAATTTACTGCCCCTAAAATTGAGGAAATTCCAGCTAAATGAAGAGAAAAAATAGCTAAATCAACAGAAGCTCCAGCATGAGCTGTTCCTGAAGAGAGAGGAGGATAAACTGTTCACCCAGTTCCTGCCCCATTTTCTACTATTGAGCTTGATAATAGAAGAGTCAATGAAGGAGGTAGTATTCAAAAACTTATATTATTTATTCGAGGAAAGGCTATATCAGGGGCTCCTAATATTAAAGGAACTAATCAATTTCCAAAATTCCCCCCCAGCA

>LA_BT_32F1

GTGGGTCAGATCAGATAAGTGTTGGTATAAAATAGGATCTCCTCCTCCGATTGGATCAAAGAAAGATGTATTTAAGTTTCGGTCTGTTAATAACATAGTAATAGCTCCAGCTAAAACAGGAAGAGAAAGAAGTAATAAGATAGCTGTAATAACTACAGATCAAACAAATAAAGGTAGTCGATCTAAAGTAATTCCTGACGATCGTATATTAATTACAGTTGTAATAAAATTTACTGCCCCTAAAATTGAGGAAATTCCAGCTAAATGAAGAGAAAAAATAGCTAAATCAACAGAAGCTCCAGCATGAGCTGTTCCTGAAGAGAGAGGAGGATAAACTGTTCACCCAGTTCCTGCCCCATTTTCTACTATTGAGCTTGATAATAGAAGAGTCAATGAAGGAGGTAGTATTCAAAAACTTATATTATTTATTCGAGGAAAGGCTATATCAGGGGCTCCTAATATTAAAGGAACTAATCAATTTACAAATTCCCCTCCCAAGA

>LA_BT_32F2

GKTGGGGGTTCAAGATCAAATAAGTGTTGGTATAAAATAGGGTCTCCCCCTCCGATTGGATCAAAGAAAGATGTATTTAAGTTTCGGTCTGTTAATAATATAGTAATAGCTCCAGCTAAAACAGGAAGAGAAAGAAGTAATAAGATAGCTGTAATAACTACAGATCAAACAAATAAGGGTAGTCGATCTAAAGTAATCCCTGACGATCGCATATTAATCACAGTTGTAATAAAATTTACTGCCCCTAAAATTGAGGAAATTCCAGCTAAATGAAGAGAAAAAATAGCTAAATCAACAGAAGCTCCAGCATGAGCTGTTCCTGAAGAGAGAGGAGGATAAACTGTTCACCCAGTCCCTGCCCCATTTTCTACTATTGAGCTTGATAATAGAAGAGTCAATGAAGGAGGTAGTATTCAAAAACTTATATTATTCATTCGAGGGAAAGCTATATCAGGGGCTCCTAATATTAAAGGAACTAATCAATTTCCAAAATCCTCCAACACT

>LA_BT_32F3

TGCGTTCAAGTATCAGATTACGTGTTCCGTATAAAATAGGATCTCCTCCTCCGATTGGATCAAAGAAAGATGTATTTAAGTTTCGGTCTGTTAATAACATAGTAATAGCTCCAGCTAAAACAGGAAGAGAAAGAAGTAATAAGATAGCTGTAATAACTACAGATCAAACAAATAAAGGTAGTCGATCTAAAGTAATTCCTGACGATCGTATATTAATTACAGTTGTAATAAAATTTACTGCCCCTAAAATTGAGGAAATTCCAGCTAAATGAAGAGAAAAAATAGCTAAATCAACAGAAGCTCCAGCATGAGCTGTTCCTGAAGAGAGAGGAGGATAAACTGTTCACCCAGTTCCTGCTCCATTTTCTACTATTGAGCTTGATAATAGAAGAGTCAATGAAGGAGGTAGTATTCAAAAACTTATATTATTTATTCGAGGAAAGGCTATATCAGGGGCTCCTAATATTAAAGGAACTAATCAATTTCAAATTCCCTCCAACA

>LA_BT_32F4

GTTGGGTCAAGATCAGATAAGTGTTGGTATAAAATAGGATCTCCTCCTCCGATTGGATCAAAGAAAGATGTATTTAAGTTTCGGTCTGTTAATAACATAGTAATAGCTCCAGCTAAAACAGGAAGAGAAAGAAGTAATAAGATAGCTGTAATAACTACAGATCAAACAAATAAAGGTAGTCGATCTAAAGTAATTCCTGACGATCGTATATTAATTACAGTTGTAATAAAATTTACTGCCCCTAAAATTGAGGAAATTCCAGCTAAATGAAGAGAAAAAATAGCTAAATCAACAGAAGCTCCAGCATGAGCTGTTCCTGAAGAGAGAGGAGGATAAACTGTTCACCCAGTTCCTGCCCCATTTTCTACTATTGAGCTTGATAATAGAAGAGTCAATGAAGGAGGTAGTATTCAAAAACTTATATTATTTATTCGAGGAAAGGCTATATCAGGGGCTCCTAATATTAAAGGAACTAATCAATTTCCAAAATTCCCTCATAGA

>LA_BT_32F5

GTGGGGTCAAGATCAGATAGTGTTGGTATAAAATAGGATCTCCTCCTCCGATTGGATCAAAGAAAGATGTATTTAAGTTTCGGTCTGTTAATAACATAGTAATAGCTCCAGCTAAAACAGGAAGAGAAAGAAGTAATAAGATAGCTGTAATAACTACAGATCAAACAAATAAAGGTAGTCGATCTAAAGTAATTCCTGACGATCGTATATTAATTACAGTTGTAATAAAATTTACTGCCCCTAAAATTGAGGAAATTCCAGCTAAATGAAGAGAAAAAATAGCTAAATCAACAGAAGCTCCAGCATGAGCTGTTCCTGAAGAGAGAGGAGGATAAACTGTTCACCCAGTTCCTGCCCCATTTTCTACTATTGAGCTTGATAATAGAAGAGTCAATGAAGGAGGTAGTATTCAAAAACTTATATTATTTATTCGAGGAAAGGCTATATCAGGGGCTCCTAATATTAAAGGAACTAATCAATTTCAAAATTCCCTCCCATA

>LA_BT_32F6

GGTCAGATCAGAATAAGTGTTGGTATAAAATAGGATCTCCTCCTCCGATTGGATCAAAGAAAGATGTATTTAAGTTTCGGTCTGTTAATAACATAGTAATAGCTCCAGCTAAAACAGGAAGAGAAAGAAGTAATAAGATAGCTGTAATAACTACAGATCAAACAAATAAAGGTAGTCGATCTAAAGTAATTCCTGACGATCGTATATTAATTACAGTTGTAATAAAATTTACTGCCCCTAAAATTGAGGAAATTCCAGCTAAATGAAGAGAAAAAATAGCTAAATCAACAGAAGCTCCAGCATGAGCTGTTCCTGAAGAGAGAGGAGGATAAACTGTTCACCCAGTTCCTGCCCCATTTTCTACTATTGAGCTTGATAATAGAAGAGTCAATGAAGGAGGTAGTATTCAAAAACTTATATTATTTATTCGAGGAAAGGCTATATCAGGGGCTCCTAATATTAAAGGAACTAATCAATTTCCAAATCCCTCCAA

>LA_BT_32F7

GGTCAGATCAGAATAAGTGTTGGTATAAAATAGGATCTCCTCCTCCGATTGGATCAAAGAAAGATGTATTTAAGTTTCGGTCTGTTAATAACATAGTAATAGCTCCAGCTAAAACAGGAAGAGAAAGAAGTAATAAGATAGCTGTAATAACTACAGATCAGACAAATAAAGGTAGTCGATCTAAAGTAATTCCTGACGATCGTATATTAATTACAGTTGTAATAAAATTTACTGCCCCTAAAATTGAGGAAATTCCAGCTAAATGAAGAGAAAAAATAGCTAAATCAACAGAAGCTCCAGCATGAGCTGTTCCTGAAGAGAGAGGAGGATAAACTGTTCACCCAGTTCCTGCCCCATTTTCTACTATTGAGCTTGATAATAGAAGAGTCAATGAAGGAGGTAGTATTCAAAAACTTATATTATTTATTCGAGGAAAGGCTATATCAGGGGCTCCTAATATTAAAGGAACTAATCAATTTCCAAAATCCTCCA

>LA_BT_32F8

GGGTCAGATCAGATAAGTGTTGGTATAAAATAGGATCTCCTCCTCCGATTGGATCAAAGAAAGATGTATTTAAGTTTCGGTCTGTTAATAACATAGTAATAGCTCCAGCTAAAACAGGAAGAGAAAGAAGTAATAAGATAGCTGTAATAACTACAGATCAAACAAATAAAGGTAGTCGGTCTAAAGTAATTCCTGACGATCGTATATTAATTACAGTTGTAATAAAATTTACTGCCCCTAAAATTGAGGAAATTCCAGCTAAATGAAGAGAAAAAATAGCTAAATCAATAGAAGCTCCAGCATGAGCTGTTCCTGAAGAGAGAGGAGGATAAACTGTTCACCCAGTTCCTGCCCCATTTTCTACTATTGAGCTTGATAATAGAAGAGTCAATGAAGGAGGTAGTATTCAAAAACTTATATTATTTATTCGAGGAAAGGCTATATCAGGGGCTCCTAATATTAAAGGAACTAATCAATTTCCAAAATCCCTCCA

>LA_BT_32F9

GGGGTCAGATCAGATAAGTGTTGGTATAAAATAGGATCTCCTCCTCCGATTGGATCAAAGAAAGATGTATTTAAGTTTCGGTCTGTTAATAACATAGTAATAGCTCCAGCTAAAACAGGAAGAGAAAGAAGTAATAAGATAGCTGTAATAACTACAGATCAAACAAATAAAGGTAGTCGATCTAAAGTAATTCCTGACGATCGTATATTAATTACAGTTGTAATAAAATTTACTGCCCCTAAAATTGAGGAAATTCCAGCTAAATGAAGAGAAAAAATAGCTAAATCAACAGAAGCTCCAGCATGAGCTGTTCCTGAAGAGAGAGGAGGATAAACTGTTCACCCAGTTCCTGCTCCATTTTCTACTATTGAGCTTGATAATAGAAGAGTCAATGAAGGAGGTAGTATTCAAAAACTTATATTATTTATTCGAGGAAAGGCTATATCAGGGGCTCCTAATATTAAAGGAACTAATCAATTTCCAAATCCTCCA

>LA_BT_32F10

GGGTCAGATCAGAATAAGTGTTGGTATAAAATAGGATCTCCTCCTCCGATTGGATCAAAGAAAGATGTATTTAAGTTTCGGTCTGTTAATAACATAGTAATAGCTCCAGCTAAAACAGGAAGAGAAAGAAGTAATAAGATAGCTGTAATAACTACAGATCAAACAAATAAAGGTAGTCGATCTAAAGTAATTCCTGACGATCGTATATTAATTACAGTTGTAATAAAATTTACTGCCCCTAAAATTGAGGAAATTCCAGCTAAATGAAGAGAAAAAATAGCTAAATCAACAGAAGCTCCAGCATGAGCTGTTCCTGAAGAGAGAGGAGGATAAACTGTTCACCCAGTTCCTGCCCCATTTTCTACTATTGAGCTTGATAATAGAAGAGTCAATGAAGGAGGTAGTATTCAAAAACTTATATTATTTATTCGAGGAAAGGCTATATCAGGGGCTCCTAATATTAAAGGAACTAATCAATTTCCAAAACCCTCCA

>LA_BT_32F11

GGGGTCAGATCAGATAAGTGTTGGTAT

AAAATAGGATCTCCTCCTCCGATTGGATCAAAGAAAGATGTATTTAAGTTTCGGTCTGTTAATAACATAGTAATAGCTCCAGCTAAAACAGGAAGAGAAAGAAGTAATAAGATAGCTGTAATAACTACAGATCAAACAAATAAAGGTAGTCGATCTAAAGTAATTCCTGACGATCGTATATTAATTACAGTTGTAATAAAATTTACTGCCCCTAAAATTGAGGAAATTCCAGCTAAATGAAGAGAAAAAATAGCTAAATCAACAGAAGCTCCAGCATGAGCTGTTCCTGAAGAGAGAGGAGGATAAACTGTTCACCCAGTTCCTGCTCCATTTTCTACTATTGAGCTTGATAATAGAAGAGTCAATGAAGGAGGTAGTATTCAAAAACTTATATTATTTATTCGAGGAAAGGCTATATCAGGGGCTCCTAATATTAAAGGAACTAATCAATTTCCAAATTCCTCCA

>LA_BT_33M1

ATCAGTATCAGATAACGTGTTCCGTATAAAATAGGATCTCCTCCTCCGATTGGATCAAAGAAAGATGTATTTAAGTTTCGGTCTGTTAATAACATAGTAATAGCTCCAGCTAAAACAGGAAGAGAAAGAAGTAATAAGATAGCTGTAATAACTACAGATCAAACAAATAAAGGTAGTCGATCTAAAGTAATTCCTGACGATCGTATATTAATTACAGTTGTAATAAAATTTACTGCCCCTAAAATTGAGGAAATTCCAGCTAAATGAAGAGAAAAAATAGCTAAATCAACAGAAGCTCCAGCATGAGCTGTTCCTGAAGAGAGAGGAGGATAAACTGTTCACCCAGTTCCTGCCCCATTTTCTACTATTGAGCTTGATAATAGAAGAGTCAATGAAGGAGGTAGTATTCAAAAACTTATATTATTTATTCGAGGAAAGGCTATATCAGGGGCTCCTAATATTAAAGGAACTAATCAATTTCCAAATTCCCCCCA

>LA_BT_33M2

GGGTCAGATCAGATAAGTGTTGGTATAAAATAGGGTCTCCCCCTCCGATTGGATCAAAGAAAGATGTATTTAAGTTTCGGTCTGTTAATAATATAGTAATAGCTCCAGCTAAAACAGGAAGAGAAAGAAGTAATAAGATAGCTGTAATAACTACAGATCAAACAAATAAGGGTAGTCGATCTAAAGTAATCCCTGACGATCGCATATTAATCACAGTTGTAATAAAATTTACTGCCCCTAAAATTGAGGAAATTCCAGCTAAATGAAGAGAAAAAATAGCTAAATCAACAGAAGCTCCAGCATGAGCTGTTCCTGAAGAGAGAGGAGGATAAACTGTTCACCCAGTTCCTGCCCCATTTTCTACTATTGAGCTTGATAATAGAAGAGTCAATGAAGGAGGTAGTATTCAAAAACTTATATTATTCATTCGAGGGAAAGCTATATCAGGGGCTCCTAATATTAAAGGAACTAATCAATTTCCAAATTCCTCCA

>LA_BT_33M3

GGTGCAAGTATCAGATAAGTGTTGGTATAAATAGGATCTCCTCCTCCGATTGGATCAAAGAAAGATGTATTTAAGTTTCGGTCTGTTAATAATATAGTAATAGCTCCAGCTAAAACAGGAAGAGAAAGAAGTAATAAGATAGCTGTAATAACTACAGATCAAACAAATAAAGGTAGTCGATCTAAAGTAATTCCTGACGATCGTATATTAATTACAGTTGTAATAAAATTTACTGCCCCTAAAATTGAGGAAATTCCAGCTAAATGAAGAGAAAAAATAGCTAAATCAACAGAAGCTCCAGCATGAGCTGTTCCTGAAGAGAGAGGAGGATAAACTGTTCACCCAGTTCCTGCCCCATTTTCTACTATTGAGCTTGATAATAGAAGAGTCAATGAAGGAGGTAGTATTCAAAAACTTATATTATTTATTCGAGGAAAGGCTATATCAGGGGCTCCTAATATTAAAGGAACTAATCAATTTCCAAATTCCCCCCA

>LA_BT_33M4

CGTCAGTATCAGATTAGTGTTGCGTATAAAATAGGATCTCCTCCTCCGATTGGATCAAAGAAAGATGTATTTAAGTTTCGGTCTGTTAATAACATAGTAATAGCTCCAGCTAAAACAGGAAGAGAAAGAAGTAATAAGATAGCTGTAATAACTACAGATCAAACAAATAAAGGTAGTCGATCTAAAGTAATTCCTGACGATCGTATATTAATTACAGTTGTAATAAAATTTACTGCCCCTAAAATTGAGGAAATTCCAGCTAAATGAAGAGAAAAAATAGCTAAATCAACAGAAGCTCCAGCATGAGCTGTTCCTGAAGAGAGAGGAGGATAAACTGTTCACCCAGTTCCTGCTCCATTTTCTACTATTGAGCTTGATAATAGAAGAGTCAATGAAGGAGGTAGTATTCAAAAACTTATATTATTTATTCGAGGAAAGGCTATATCAGGGGCTCCTAATATTAAAGGAACTAATCAATTTCAAATTCCCCCCA

>LA_BT_33M5

GTCAGTATCAGATTTACGTGTTCCGTATAAAATAGGATCTCCTCCTCCGATTGGATCAAAGAAAGATGTATTTAAGTTTCGGTCTGTTAATAACATAGTAATAGCTCCAGCTAAAACAGGAAGAGAAAGAAGTAATAAGATAGCTGTAATAACTACAGATCAAACAAATAAAGGTAGTCGATCTAAAGTAATTCCTGACGATCGTATATTAATTACAGTTGTAATAAAATTTACTGCCCCTAAAATTGAGGAAATTCCAGCTAAATGAAGAGAAAAAATAGCTAAATCAACAGAAGCTCCAGCATGAGCTGTTCCTGAAGAGAGAGGAGGATAAACTGTTCACCCAGTTCCTGCCCCATTTTCTACTATTGAGCTTGATAATAGAAGAGTCAATGAAGGAGGTAGTATTCAAAAACTTATATTATTTATTCGAGGAAAGGCTATATCAGGGGCTCCTAATATTAAAGGAACTAATCAAATTTCAAATTCCTCCA

>LA_BT_33M6

GGTCAGATCAGATAGTGTTGGTATAAAATAGGATCTCCTCCTCCGATTGGATCAAAGAAAGATGTATTTAAGTTTCGGTCTGTTAATAACATAGTAATAGCTCCAGCTAAAACAGGAAGAGAAAGAAGTAATAAGATAGCTGTAATAACTACAGATCAAACAAATAAAGGTAGTCGATCTAAAGTAATTCCTGACGATCGTATATTAATTACAGTTGTAATAAAATTTACTGCCCCTAAAATTGAGGAAATTCCAGCTAAATGAAGAGAAAAAATAGCTAAATCAACAGAAGCTCCAGCATGAGCTGTTCCTGAAGAGAGAGGAGGATAAACTGTTCACCCAGTTCCTGCTCCATTTTCTACTATTGAGCTTGATAATAGAAGAGTCAATGAAGGAGGTAGTATTCAAAAACTTATATTATTTATTCGAGGAAAGGCTATATCAGGGGCTCCTAATATTAAAGGAACTAATCAATTCCAAATTCCCCCCA

>LA_BT_33M7

CGTCAGTATCACGATTTACGTGTTCCGTATAAAATAGGATCTCCTCCTCCGATTGGATCAAAGAAAGATGTATTTAAGTTTCGGTCTGTTAATAACATAGTAATAGCTCCAGCTAAAACAGGAAGAGAAAGAAGTAATAAGATAGCTGTAATAACTACAGATCAAACAAATAAAGGTAGTCGATCTAAAGTAATTCCTGACGATCGTATATTAATTACAGTTGTAATAAAATTTACTGCCCCTAAAATTGAGGAAATTCCAGCTAAATGAAGAGAAAAAATAGCTAAATCAACAGAAGCTCCAGCATGAGCTGTTCCTGAAGAGAGAGGAGGATAAACTGTTCACCCAGTTCCTGCTCCATTTTCTACTATTGAGCTTGATAATAGAAGAGTCAATGAAGGAGGTAGTATTCAAAAACTTATATTATTTATTCGAGGAAAGGCTATATCAGGGGCTCCTAATATTAAAGGAACTAATCAATTACAAAATTCCCCCCA

>LA_BT_33M8

GGGTCAGATCAGAATAAGTGTTGGTATAAAATAGGATCTCCTCCTCCGATTGGATCAAAGAAAGATGTATTTAAGTTTCGGTCTGTTAATAACATAGTAATAGCTCCAGCTAAAACAGGAAGAGAAAGAAGTAATAAGATAGCTGTAATAACTACAGATCAAACAAATAAAGGTAGTCGATCTAAAGTAATTCCTGACGATCGTATATTAATTACAGTTGTAATAAAATTTACTGCCCCTAAAATTGAGGAAATTCCAGCTAAATGAAGAGAAAAAATAGCTAAATCAACAGAAGCTCCAGCATGAGCTGTTCCTGAAGAGAGAGGAGGATAAACTGTTCACCCAGTTCCTGCTCCATTTTCTACTATTGAGCTTGATAATAGAAGAGTCAATGAAGGAGGTAGTATTCAAAAACTTATATTATTTATTCGAGGAAAGGCTATATCAGGGGCTCCTAATATTAAAGGAACTAATCAATTTCAAATTCCCTCCA

>LA_BT_33M9

CGTCAAGCTATCACGATTAACSTGTTCCGTCCAAAATAGGATCTCCTCCTCCGATTGGATCAAAGAAAGATGTATTTAAGTTTCGGTCTGTTAATAACATAGTAATAGCTCCAGCTAAAACAGGAAGAGAAAGAAGTAATAAGATAGCTGTAATAACTACAGATCAAACAAATAAAGGTAGTCGATCTAAAGTAATTCCTGACGATCGTATATTAATTACAGTTGTAATAAAATTTACTGCCCCTAAAATTGAGGAAATTCCAGCTAAATGAAGAGAAAAAATAGCTAAATCAACAGAAGCTCCAGCATGAGCTGTTCCTGAAGAGAGAGGAGGATAAACTGTTCACCCAGTTCCTGCTCCATTTTCTACTATTGAGCTTGATAATAGAAGAGTCAATGAAGGAGGTAGTATTCAAAAACTTATATTATTTATTCGAGGAAAGGCTATATCAGGGGCTCCTAATATTAAAGGAACTAATCAATATCCAAATTCCCCCCA

>LA_BT_33M10

GGGTCAGATCAGATAAGTGTTGGTATAAAATAGGATCTCCTCCTCCGATTGGATCAAAGAAAGATGTATTTAAGTTTCGGTCTGTTAATAACATAGTAATAGCTCCGGCTAAAACAGGAAGAGAAAGAAGTAATAAGATAGCTGTAATAACTACMGATCAAACAAATAAAGGTAGTCGATCTAAAGTAATTCCTGACGATCGTATATTAATTACAGTTGTAATAAAATTTACTGCCCCTAAAATTGAGGAAATTCCAGCTAAATGAAGAGAAAAAATAGCTAAATCAACAGAAGCTCCAGCATGAGCTGTTCCTGAAGAGAGAGGAGGATAAACTGTTCACCCAGTTCCTGCTCCATTTTCTACTATTGAGCTTGATAATAGAAGAGTCAATGAAGGAGGTAGTATTCAAAAACTTATATTATTTATTCGAGGAAAGGCTATATCAGGGGCTCCTAATATTAAAGGAACTAATCCATTTCCAAAWTCCTCCA

>LA_BT_33M11

GGGTCAAGTATCAGATAAGTGTTGGTATAAAATAGGATCTCCTCCTCCGATTGGATCAAAGAAAGATGTATTTAAGTTTCGGTCTGTTAATAATATAGTAATAGCTCCAGCTAAAACAGGAAGAGAAAGAAGTAATAAGATAGCTGTAATAACTACAGATCAAACAAATAAAGGTAGTCGATCTAAAGTAATTCCTGACGATCGTATATTAATTACAGTTGTAATAAAATTTACTGCCCCTAAAATTGAGGAAATTCCAGCTAAATGAAGAGAAAAAATAGCTAAATCAACAGAAGCTCCAGCATGAGCTGTTCCTGAAGAGAGAGGAGGATAAACTGTTCACCCAGTTCCTGCCCCATTTTCTACTATTGAGCTTGATAATAGAAGAGTCAATGAAGGAGGTAGTATTCAAAAACTTATATTATTTATTCGAGGAAAGGCTATATCAGGGGCTCCTAATATTAAAGGAACTAATCATTTCCAAATTCCCCCCA

>LA_BT_34M1

GTTCAGTATCAGATAAGTGTTGGTATAAAATAGGATCTCCTCCTCCGATTGGATCAAAGAAAGATGTATTTAAGTTTCGGTCTGTTAATAACATAGTAATAGCTCCAGCTAAAACAGGAAGAGAAAGAAGTAATAAGATAGCTGTAATAACTACAGATCAAACAAATAAAGGTAGTCGATCTAAAGTAATTCCYGACGATCGTATATTAATTACAGTTGTAATAAAATTTACTGCCCCTAAAATTGAGGAAATTCCAGCTAAATGAAGAGAAAAAATAGCTAAATCAACAGAAGCTCCAGCATGAGCTGTTCCTGAAGAGAGAGGAGGATAAACTGTTCACCCAGTTCCTGCCCCATTTTCTACTATTGAGCTTGATAATAGAAGAGTCAATGAAGGAGGTAGTATTCAAAAACTTATATTATTTATTCGAGGAAAGGCTATATCAGGGGCTCCTAATATTAAAGGAACTAATCAATTCCAAATTCCCCCCA

>LA_BT_34M2

CGGTCAGATCAGATAAGTGTTGGTATAAAATAGGATCTCCTCCTCCGATTGGATCAAAGAAAGATGTATTTAAGTTTCGGTCTGTTAATAACATAGTAATAGCTCCGGCTAAAACAGGAAGAGAAAGAAGTAATAAGATAGCTGTAATAACTACAGATCAAACAAATAAAGGTAGTCGATCTAAAGTAATTCCTGACGATCGTATATTAATTACAGTTGTAATAAAATTTACTGCCCCTAAAATTGAGGAAATTCCAGCTAAATGAAGAGAAAAAATAGCTAAATCAACAGAAGCTCCAGCATGAGCTGTTCCTGAAGAGAGAGGAGGATAAACTGTTCACCCAGTTCCTGCTCCATTTTCTACTATTGAGCTTGATAATAGAAGAGTCAATGAAGGAGGTAGTATTCAAAAACTTATATTATTTATTCGAGGAAAGGCTATATCAGGGGCTCCTAATATTAAAGGAACTAATCAATMAMAAAATTCCCCCCA

>LA_BT_34M3

GGTCAAAGATCAGATAAGTGTTGGTATAAAATAGGATCTCCTCCTCCGATTGGATCAAAGAAAGATGTATTTAAGTTTCGGTCTGTTAATAACATAGTAATAGCTCCAGCTAAAACAGGAAGAGAAAGAAGTAATAAGATAGCTGTAATAACTACAGATCAAACAAATAAAGGTAGTCGATCTAAAGTAATTCCTGACGATCGTATATTAATTACAGTTGTAATAAAATTTACTGCCCCTAAAATTGAGGAAATTCCAGCTAAATGAAGAGAAAAAATAGCTAAATCAACAGAAGCTCCAGCATGAGCTGTTCCTGAAGAGAGAGGAGGATAAACTGTTCACCCAGTTCCTGCTCCATTTTCTACTATTGAGCTTGATAATAGAAGAGTCAATGAAGGAGGTAGTATTCAAAAACTTATATTATTTATTCGAGGAAAGGCTATATCAGGGGCTCCTAATATTAAAGGAACTAATCAATTTCAAAAATCCTCCA

>LA_BT_34M4

GTCAGTATCAGTTACGTGTTCCGTATAAAATAGGATCTCCTCCTCCGATTGGATCAAAGAAAGATGTATTTAAGTTTCGGTCTGTTAATAATATAGTAATAGCTCCAGCTAAAACAGGAAGAGAAAGAAGTAATAAGATAGCTGTAATAACTACAGATCAAACAAATAAAGGTAGTCGATCTAAAGTAATTCCTGACGATCGTATATTAATTACAGTTGTAATAAAATTTACTGCCCCTAAAATTGAGGAAATTCCAGCTAAATGAAGAGAAAAAATAGCTAAATCAACAGAAGCTCCAGCATGAGCTGTTCCTGAAGAGAGAGGAGGATAAACTGTTCACCCAGTTCCTGCCCCATTTTCTACTATTGAGCTTGATAATAGAAGAGTCAATGAAGGAGGTAGTATTCAAAAACTTATATTATTTATTCGAGGAAAGGCTATATCAGGGGCTCCTAATATTAAAGGAACTAATCAATTTCAAAATTCCCCCCA

>LA_BT_34M5

CGTCAAGATCAGATAAGTGTTGGTATAAAATAGGATCTCCTCCTCCGATTGGATCAAAGAAAGATGTATTTAAGTTTCGGTCTGTTAATAACATAGTAATAGCTCCCGCTAAAACAGGAAGAGAAAGAAGTAATAAGATAGCTGTAATAACTACAGATCAAACAAATAAAGGTAGTCGATCTAAAGTAATTCCTGACGATCGTATATTAATTACAGTTGTAATAAAATTTACTGCCCCTAAAATTGAGGAAATTCCAGCTAAATGAAGAGAAAAAATAGCTAAATCAACAGAAGCTCCAGCATGAGCTGTTCCTGAAGAGAGAGGAGGATAAACTGTTCACCCAGTTCCTGCTCCATTTTCTACTATTGAGCTTGATAATAGAAGAGTCAATGAAGGAGGTAGTATTCAAAAACTTATATTATTTATTCGAGGAAAGGCTATATCAGGGGCTCCTAATATTAAAGGAACTAATCAATTCCAAATTCCCCCCCA

>LA_BT_34M6

CGTCAAGTATCCGATWSTGATCCGTACAAAATAGGGTCTCCCCCTCCGATTGGATCAAAGAAAGATGTATTTAAGTTTCGGTCTGTTAATAATATAGTAATAGCTCCAGCTAAAACAGGAAGAGAAAGAAGTAATAAGATAGCTGTAATAACTACAGATCAAACAAATAAGGGTAGTCGATCTAAAGTAATCCCTGACGATCGCATATTAATCACAGTTGTAATAAAATTTACTGCCCCTAAAATTGAGGAAATTCCAGCTAAATGAAGAGAAAAAATAGCTAAATCAACAGAAGCTCCAGCATGAGCTGTTCCTGAAGAGAGAGGAGGATAAACTGTTCACCCAGTTCCTGCCCCATTTTCTACTATTGAGCTTGATAATAGAAGAGTCAATGAAGGAGGTAGTATTCAAAAACTTATATTATTCATTCGAGGGAAAGCTATATCAGGGGCTCCTAATATTAAAGGAACTAATCAATTTCAAATTCCCCCCA

>LA_BT_34M7

GGTCAGATCAGATAAGTGTTGGTATAAAATAGGATCTCCTCCTCCGATTGGATCAAAGAAAGATGTATTTAAGTTTCGGTCTGTTAATAACATAGTAATAGCTCCAGCTAAAACAGGAAGAGAAAGAAGTAATAAGATAGCTGTAATAACTACAGATCAAACAAATAARGGTAGTCGATCTAAAGTAATTCCTGACGATCGTATATTAATTACAGTTGTAATAAAATTTACTGCCCCTAAAATTGAGGAAATTCCAGCTAAATGAAGAGAAAAAATAGCTAAATCAACAGAAGCTCCAGCATGAGCTGTTCCTGAAGAGAGAGGAGGATAAACTGTTCACCCAGTTCCTGCCCCATTTTCTACTATKGAGCTTGATAATAGAAGAGTCAATGAAGGAGGTAGTATTCAAAAACTTATATTATTTATTCGAGGAAAGGCTATATCAGGGGCTCCTAATATTAAAGGAACTAATCAATTTCCAAAATCCTCCA

>LA_BT_34M8

GGGTCGGATCAGAATAGTGTTGGTATAAAATAGGATCTCCTCCTCCGATTGGATCAAAGAAAGATGTATTTAAGTTTCGGTCTGTTAATAACATAGTAATAGCTCCAGCTAAAACAGGAAGAGAAAGAAGTAATAAGATAGCTGTAATAACTACAGATCAAACAAATAAAGGTAGTCGATCTAAAGTAATTCCTGACGATCGTATATTAATTACAGTTGTAATAAAATTTACTGCCCCTAAAATTGAGGAAATTCCAGCTAAATGAAGAGAAAAAATAGCTAAATCAACAGAAGCTCCAGCATGAGCTGTTCCTGAAGAGAGAGGAGGATAAACTGTTCACCCAGTTCCTGCTCCATTTTCTACTATTGAGCTTGATAATAGAAGAGTCAATGAAGGAGGTAGTATTCAAAAACTTATATTATTTATTCGAGGAAAGGCTATATCAGGGGCTCCTAATATTAAAGGAACTAATCAATTTCCAAATTCCACCAACC

>LA_BT_34M9

CGTCATGATCAGATAAGTGTTGGTATAAAATAGGATCTCCTCCTCCGATTGGATCAAAGAAAGATGTATTTAAGTTTCGGTCTGTTAATAACATAGTAATAGCTCCGGCTAAAACAGGAAGAGAAAGAAGTAATAAGATAGCTGTAATAACTACAGATCAAACAAATAAAGGTAGTCGATCTAAAGTAATTCCTGACGATCGTATATTAATTACAGTTGTAATAAAATTTACTGCCCCTAAAATTGAGGAAATTCCAGCTAAATGAAGAGAAAAAATAGCTAAATCAACAGAAGCTCCAGCATGAGCTGTTCCTGAAGAGAGAGGAGGATAAACTGTTCACCCAGTTCCTGCTCCATTTTCTACTATTGAGCTTGATAATAGAAGAGTCAATGAAGGAGGTAGTATTCAAAAACTTATATTATTTATTCGAGGAAAGGCTATATCAGGGGCTCCTAATATTAAAGGAACTAATCAATTAACAAATTCCCCCCCA

>LA_BT_34M10

GGGTCAGATCAGATAAGTGTTGGTATAAAATAGGATCTCCTCCTCCGATTGGATCAAAGAAAGATGTATTTAAGTTTCGGTCTGTTAATAACATAGTAATAGCTCCAGCTAAAACAGGAAGAGAAAGAAGTAATAAGATAGCTGTAATAACTACAGATCAAACAAATAAAGGTAGTCGATCTAAAGTAATTCCTGACGATCGTATATTAATTACAGTTGTAATAAAATTTACTGCCCCTAAAATTGAGGAAATTCCAGCTAAATGAAGAGAAAAAATAGCTAAATCAACAGAAGCTCCAGCATGAGCTGTTCCTGAAGAGAGAGGAGGATAAACTGTTCACCCAGTTCCTGCTCCATTTTCTACTATTGAGCTTGATAATAGAAGAGTCAATGAAGGAGGTAGTATTCAAAAACTTATATTATTTATTCGAGGAAAGGCTATATCAGGGGCTCCTAATATTAAAGGAACTAATCAATTCCAAATTCCTCCA

>LA_BT_34M11

CTGCAGATCAGATAAGTGTTGGTATAAATAGGATCTCCTCCTCCGATTGGATCAAAGAAAGATGTATTTAAGTTTCGGTCTGTTAATAATATAGTAATAGCTCCAGCTAAAACAGGAAGAGAAAGAAGTAATAAGATAGCTGTAATAACTACAGATCAAACAAATAAAGGTAGTCGATCTAAAGTAATTCCTGACGATCGTATATTAATTACAGTTGTAATAAAATTTACTGCCCCTAAAATTGAGGAAATTCCAGCTAAATGAAGAGAAAAAATAGCTAAATCAACAGAAGCTCCAGCATGAGCTGTTCCTGAAGAGAGAGGAGGATAAACTGTTCACCCAGTTCCTGCCCCATTTTCTACTATTGAGCTTGATAATAGAAGAGTCAATGAAGGAGGTAGTATTCAAAAACTTATATTATTTATTCGAGGAAAGGCTATATCAGGGGCTCCTAATATTAAAGGAACTAATCAATTTCAAAATTCCCCCCA

>LA_BT_34M12

GGTCATGTATCAGAATAAGTGTTGGTATAAAATAGGATCTCCTCCTCCGATTGGATCAAAGAAAGATGTATTTAAGTTTCGGTCTGTTAATAACATAGTAATAGCTCCAGCTAAAACAGGAAGAGAAAGAAGTAATAAGATAGCTGTAATAACTACAGATCAAACAAATAAAGGTAGTCGATCTAAAGTAATTCCTGACGATCGTATATTAATTACAGTTGTAATAAAATTTACTGCCCCTAAAATTGAGGAAATTCCAGCTAAATGAAGAGAAAAAATAGCTAAATCAACAGAAGCTCCMGCATGAGCTGTTCCTGAAGAGAGAGGAGGATAAACTGTTCACCCAGTTCCTGCCCCATTTTCTACTATTGAGCTCGATAATAGAAGAGTCAATGAAGGAGGTAGTATTCAAAAACTTATATTATTTATTCGAGGAAAGGCTATATCAGGGGCTCCTAATATTAAAGGAACTAATCAATTTCAAAATTCCCCCCA

>LA_BT_34M13

GTCAGTATCACGATTAACCTGATCCGTCCAAAATAGGATCTCCTCCTCCGATTGGATCAAAGAAAGATGTATTTAAGTTTCGGTCTGTTAATAACATAGTAATAGCTCCGGCTAAAACAGGAAGAGAAAGAAGTAATAAGATAGCTGTAATAACTACAGATCAAACAAATAAAGGTAGTCGATCTAAAGTAATTCCTGACGATCGTATATTAATTACAGTTGTAATAAAATTTACTGCCCCTAAAATTGAGGAAATTCCAGCTAAATGAAGAGAAAAAATAGCTAAATCAACAGAAGCTCCAGCATGAGCTGTTCCTGAAGAGAGAGGAGGATAAACTGTTCACCCAGTTCCTGCTCCATTTTCTACTATTGAGCTTGATAATAGAAGAGTCAATGAAGGAGGTAGTATTCAAAAACTTATATTATTTATTCGAGGAAAGGCTATATCAGGGGCTCCTAATATTAAAGGAACTAATCAATTTCAAATTCCCTCCCA

>LA_BT_34M14

GGTGCAGTATCAGATAAGTGTTGGTATAAAATAGGATCTCCTCCTCCGATTGGATCAAAGAAAGATGTATTTAAGTTTCGGTCTGTTAATAATATAGTAATAGCTCCCGCTAAAACAGGAAGAGAAAGAAGTAATAAGATAGCTGTAATAACTACAGATCAAACAAATAAAGGTAGTCGATCTAAAGTAATTCCTGACGATCGTATATTAATTACAGTTGTAATAAAATTTACTGCCCCTAAAATTGAGGAAATTCCAGCTAAATGAAGAGAAAAAATAGCTAAATCAACAGAAGCTCCAGCATGAGCTGTTCCTGAAGAGAGAGGAGGATAAACTGTTCACCCAGTTCCTGCCCCATTTTCTACTATTGAGCTTGATAATAGAAGAGTCAATGAAGGAGGTAGTATTCAAAAACTTATATTATTTATTCGAGGAAAGGCTATATCAGGGGCTCCTAATATTAAAGGAACTAATCATTTCCAAATCCCTCCA

>HT_PG_36F1

TAGGTAACTACTCAATTTCCATATCATACCTAATTGGGTCTCCTCCCTTTCCTTGCAATCTAAAAAGATGTCTTTTCCTTTCGGTCTGTTAATAATATAGTAATAGCTCCGGCTAATACGGGTAGAGAAAGAAGTAATAAAATAGCTGTAATTACTACTGATCACACAAATAAAGGTAGTCGATCAAGAGTAATACCAGCTGATCGTATATTAATTACAGTTGTAATAAAATTTACTGCTCCTAAAATAGATGAGATTCCCGCTAAATGTAAAGAAAAAATTGCTAAATCAACTGAAGCCCCAGCATGAGCTGTTCCAGAAGAAAGGGGAGGATAAACCGTTCACCCTGTTCCAGCTCCGTTTTCTACTATAGAACTAGAAAGCAGCAGTGTTAAAGAGGGGGGTAATATTCAAAAACTTATATTATTTATTCGAGGAAAAGCTATATCAGGGGCTCCTAGTATTAAGGGAACTAATCAATTTCCAAAATCCTCCAA

>HT_PG_36F2

ATACTACTCATTTCAGAATGCATACCCAATTGGGTCTCCTCCTCCCCTTCGCGATCAAAAAAAGATGTATTTTAATTTAGGTCTGTTAATAATATAGTAATAGCTCCGGCTAATACGGGTAGAGAAAGAAGTAATAAAATAGCTGTAATTACTACTGATCACACAAATAAAGGTAGTCGATCAAGAGTAATACCAGCTGATCGTATATTAATTACAGTTGTAATAAAATTTACTGCTCCTAAAATAGATGAGATTCCCGCTAAATGTAAAGAAAAAATTGCTAAATCAACTGAAGCCCCAGCATGAGCTGTTCCAGAAGAAAGGGGAGGATAAACCGTTCACCCTGTTCCAGCTCCGTTTTCTACTATAGAACTAGAAAGCAGCAGTGTTAAAGAGGGGGGTAATATTCAAAAACTTATATTATTTATTCGAGGAAAAGCTATATCAGGGGCTCCTAGTATTAAGGGAACTAATCAATTTCCAAATTCCTCCAA

>HT_PG_36F3

CGAACTAACTCAATTTCCAAATCCTACCCAACTAGTGACTTCTCCTATCCTTCGCAAACTAATCAAGATTCCTTTTCCTTTCGGTCTGTTAATAATATAGTAATAGCTCCGGCTAATACGGGTAGAGAAARAAGTAATAAAATAGCTGTAATTACTACTGATCACACAAATAAAGGTAGTCGATCTAGAGTAATTCCAGCTGATCGTATATTAATTACAGTTGTAATAAAATTTACTGCTCCTAAAATAGATGAGATTCCCGCTAAATGTAAAGAAAAAATTGCTAAATCAACTGAAGCCCCAGCATGAGCTGTTCCAGAAGAAAGGGGAGGATAAACTGTTCACCCTGTTCCAGCTCCGTTTTCTACTATAGAACTAGAAAGTAGCAGAGTTAAAGAGGGGGGTAATATTCAAAAACTTATATTATTTATTCGAGGAAAAGCTATATCAGGGGCTCCTAGTATTAAGGGAACTAATCAATTTCAAAATCCTCCA

>HT_Hv_37F1

AACGAACTACTCAATTTACCAAACGCCTACCCAACAGGGTCTCCCCCTCCCCTTCGCAATCAAAAAAAGATGCATTTGAATTTMGGTCWGTTAATAATATAGTAATAGCTCCGGCTAATACGGGTAGAGAAAGAAGTAATAAAATAGCTGTAATTACTACTGATCACACAAATAAAGGTAGTCGATCAAGAGTAATACCAGCTGATCGTATATTAATTACAGTTGTAATAAAATTTACTGCTCCTAAAATAGATGAGATTCCCGCTAAATGTAGAGAAAAAATTGCTAAATCAACTGAAGCCCCAGCATGAGCTGTTCCAGAAGAAAGGGGAGGATAAACCGTTCACCCTGTTCCAGCTCCGTTTTCTACTATAGAACTAGAAAGCAGCAGWGTTAAAGAGGGGGGTAATATTCAAAAACTTATATTATTTATTCGAGGAAAAGCTATATCAGGGGCTCCTAGTATTAAGGGAACTAATCAATTTCAAAATCCTCCAA

>HT_Hv_37F2

CTAAGAGCTAATCAATTTACAAACACTCCCAATTCGGTCTTCTCCTTTCCTTCGCAATACAAAAAAGATGTATTTTCCTTTCGGTCTGTTAATAATATAGTAATAGCTCCGGCTAATACGGGTAGAGAAAGAAGTAATAAAATAGCTGTAATTACTACTGATCACACAAATAAAGGTAGTCGATCAAGAGTAATACCAGCTGATCGTATATTAATTACAGTTGTAATAAAATTTACTGCTCCTAAAATAGATGAGATTCCCGCTAAATGTAAAGAAAAAATTGCTAAATCAACTGAAGCCCCAGCATGAGCTGTTCCAGAAGAAAGGGGAGGATAAACCGTTCACCCTGTTCCAGCTCCGTTTTCTACTATAGAACTAGAAAGCAGCAGTGTTAAAGAGGGGGGTAATATTCAAAAACTTATATTATTTATTCGAGGAAAAGCTATATCAGGGGCTCCTAGTATTAAGGGAACTAATCAATTTCCAATCCTCCA

>TTH_L_38M1

ACTACTCATTTCCATATCCTACCAAACAGGTGTCTTCTCCCTCACCTTGCGATCTAAAAAAGATGTATTTAAATTTCGGTCTGTTAATAATATAGTAATAGCTCCGGCTAATACGGGTAGAGAAAGAAGTAATAAAATAGCTGTAATTACTACTGATCACACAAATAAAGGTAGTCGATCAAGAGTAATACCAGCTGATCGTATATTAATTACAGTTGTAATAAAATTTACTGCTCCTAAAATAGATGAGATTCCCGCTAAATGTAAAGAAAAAATTGCTAAATCAACTGAAGCCCCAGCATGAGCTGTTCCAGAAGAAAGGGGAGGATAAACCGTTCACCCTGTTCCAGCTCCGTTTTCTACTATAGAACTAGAAAGCAGCAGTGTTAAAGAGGGGGGTAATATTCAAAAACTTATATTATTTATTCGAGGAAAAGCTATATCAGGGGCTCCTAGTATTAAGGGAACTAATCAATTTCAAAATCCTCCAA

>TTH_L_38M2

GCGTACTATCAATTTCAAATCCAACCAACTAGCCACTTTTCCTCTCCTTCGCGAACTAATAATGATGTATTTCCTTTTCGATCTGTTAATAATATAGTAATAGCTCCTGCTAATACTGGTAATGATAAAAGTAATAAAATAGCTGTAATTACTACAGATCAGACAAATAAAGGTAAACGATCTAAAGTAATTCCTGCTGATCGTATATTAATTACAGTAGTAATAAAATTTACTGCTCCTAAAATTGAAGAAATCCCTGCTAAATGAAGAGAAAAAATAGCTAAATCAACTGATGCACCAGCATGAGCAGTTCCTGAAGATAGAGGAGGATAAACTGTTCAACCTGTTCCTGCTCCATTTTCTACTATAGAACTAGAAAGTAGTAGAGTTAATGAAGGAGGTAATATTCAAAAACTTATATTATTTATTCGAGGGAAAGCTATATCAGGAGCTCCTAATATCAAAGGAACTAATCAATTTCAAAATCCTCCA

>TTH_L_38M3

CAAAACTAATCAATTTCCTATCCTAACTACTAATAATTTTCCCAATCCTCGAAACTAATAATTTTCCATTTCCACCTCGATCTGTTAATAACATAGTAATAGCTCCTGCTAATACTGGTAATGATAAAAGTAATAGAATAGCTGTAATTACTACAGATCAGACAAATAAAGGTAAACGATCTAAAGTAATTCCTGCTGATCGTATATTAATTACAGTAGTAATAAAATTTACTGCTCCTAAAATTGAAGAAATCCCTGCTAAATGAAGAGAAAAAATAGCTAAATCAACTGATGCACCAGCATGAGCAGTTCCAGAAGATAGAGGAGGATAAACTGTTCAACCTGTTCCTGCTCCATTTTCTACTATAGAACTAGAAAGTAGTAGTGTTAATGAAGGAGGTAATATTCAAAAACTTATATTATTTATTCGAGGGAAAGCTATATCAGGAGCTCCTAATATCAGAGGAACTAATCAATTTCAAAATCCTCCAA

>TTH_L_38M4

AGTAACACTCAATTTACCATATGCCTATCCAATAGGGTCTCCTCCTCCAATTGCGATCAAAAAAAGATGTATTTAAATTTCGGTCTGTTAATAATATAGTAATAGCTCCGGCTAATACGGGTAGAGAAAGAAGTAATAAAATAGCTGTAATTACTACTGATCACACAAATAAAGGTAGTCGATCAAGAGTAATACCAGCTGATCGTATATTAATTACAGTTGTAATAAAATTTACTGCTCCTAAAATAGATGAGATTCCCGCTAAATGTAAAGAAAAAATTGCTAAATCAACTGAAGCCCCAGCATGAGCTGTTCCAGAAGAAAGGGGAGGATAAACCGTTCACCCTGTTCCAGCTCCGTTTTCTACTATAGAACTAGAAAGCAGCAGTGTTAAAGAGGGGGGTAATATTCAAAAACTTATATTATTTATTCGAGGAAAAGCTATATCAGGGGCTCCTAGTATTAAGGGAACTAATCAATTTCAAAAATCCTCCAA

>TTH_L_39F1

ATTGGCTCCTCTTTTATACAATCCCACTGTAATTTCCAATCCTCCGAACTAGTCATGTTTCCAAATCCATCCTCGATCTGTTAATAATATAGTAATAACTCCTGCTAATACTGGTAATGATAAAAGTAATAAAATAGCTGTAATTACTACAGATCAGACAAATAAAGGTAAACGATCTAAAGTAATTCCTGCTGATCGTATATTAATTACAGTAGTAATAAAATTTACTGCTCCTAAAATTGAAGAAATCCCTGCTAAATGAAGAGAAAAAATAGCTAAATCAACTGATGCTCCAGCATGAGCAGTTCCAGAAGATAGAGGAGGATAAACTGTTCACCCTGTTCCTGCTCCGTTTTCTACTATAGAACTAGAAAGTAGTAGTGTTAATGAAGGAGGTAATATTCAAAAACTTATATTATTTATTCGAGGGAAAGCTATATCAGGAGCTCCTAATATCAAAGGAACTAATCAATTTCCAAATCCTCCCA

>TTH_L_39F2

GTGGGCTAAGGGATAAAATTTTCTGCCCAAAATAGGACTCCCCCCCCCATGGGAGAAGAAGAATTTTTTTTTTCTTCTTTTAAAAGTAAATGCTCCCCCAAAGGAAAGAAAAATTTAAAATAGCTCTTAAACTACCAAAAACCATTGGGGGGTCTATATTAGAATTTCCGAGTATAGTGTATTTTACAGTGGTGAATTATTTTTTGCGCCCCTGATTGAGGAGATATTCCCTCTATATAAAGAAAAAATATCTCT

TCAACACACACTCTCCGGAGCTCTGTTCCAAGAGAAGAGAGGGGATCTGCCGTTCCCCCTGCTGCCGCTCTTTCTTCTATTGTGCTGGTTGATAGTAGAGTAGTTGAGGGGGGGGGTAGTCTTCAACTTATTTTTATCTATTATAGGGAGGAGCGGTGTTTGTGCGCGTCGCTCCTAGAATTGGAAGGTAATCTCCTTTCCCTTCTCCCCCCCTCCAGCCAGG

>TTH_L_39F3

CTTGGCTCTTATTTCTATCATCCGAACTGGCAATTTCCCAATCCTCCAAAGTAGTAATGTTCCATATCCACCTCCATCAGTTATTAATATAGTAATAACTCCTGCTAATACTGGTAATGATAAAAGTAATAAAATAGCTGTAATTACAACAGATCAAACAAATAAAGGTAAACGATCTAAAGTAATTCCTGCTGATCGTATATTAATTACAGTAGTAATAAAATTTACTGCTCCTAAAATTGAAGAAATTCCTGCTAAATGAAGAGAAAAAATAGCTAAATCAACTGATGCACCAGCATGAGCAGTTCCAGAAGATAGAGGAGGATAAACTGTTCAGCCTGTTCCTGCTCCGTTTTCTACTATAGAACTAGAAAGTAGTAGTGTTAATGAAGGAGGTAATATTCAAAAACTTATATTATTTATTCGAGGGAAAGCTATATCAGGAGCTCCTAATATCAAAGGAACTAATCAATTTCCAAATCCTCCAA

>TTH_L_39F4

TTAACCAGATACTCGCACTATCCCAATAGGGTCTCCCCCTCCCATTCGCGATCAAAAAAAGATGTATTTAAATTTCGGTCTGTTAATAATATAGTAATAGCTCCGGCTAATACGGGTAGAGAAAGAAGTAATAAAATAGCTGTAATTACTACTGATCACACAAATAAAGGTAGTCGATCAAGAGTAATACCAGCTGATCGTATATTAATTACAGTTGTAATAAAATTTACTGCTCCTAAAATAGATGAGATTCCCGCTAAATGTAAAGAAAAAATTGCTAAATCAACTGAAGCCCCAGCATGAGCTGTTCCAGAAGAAAGGGGAGGATAAACCGTTCACCCTGTTCCAGCTCCGTTTTCTACTATAGAACTAGAAAGCAGCAGTGTTAAAGAGGGGGGTAATATTCAAAAACTTATATTATTTATTCGAGGAAAAGCTATATCAGGGGCTCCTAGTATTAAGGGAACTAATCAATTTCCAAAATCCTCCCA

>TTH_L_39F5

ACTGCTTCGTCTAATCACTTTCCAAATCCTCCGACTATCATTTTCCCAATCCTCCAAAGTAATCATTTTCCATTTCCTCCACCTCCATGTTTATAATATATTCATCACTCCCGCTAAAACGGGAAGAGAAAGAAGTAATAAGATAGCTGTAATAACTACAGATCAAACAAATAAAGGTAGTCGATCTAAAGTAATTCCTGACGATCGTATATTAATTACAGTTGTAATAAAATTTACTGCCCCTAAAATTGAGGAAATTCCTGCTAAATGAAGAGAAAAAATAGCTTAATCAACAGAAGCTCCAGCATGAGCTGTTCCTGAAGAGAGAGGAGGAAAAACTGTTCACCCTGTTCCTGCCCCATTTTCTACTATTGAGCTTGATAATAGAAGAGTTAATGAAGGAGGTAGTATTCAAAAACTTATATTATTTATTCGAGGAGAGGCTATATGGGGGGCTCCTAATATTAAAGGAACTAATCCATTTCCAAATCCTCCA

>TTH_L_39F6

ATTCTCGTAGCTACTCAATTTCCACATCCTACCGAACAGGGTCTCCCCCATCTCACTTCGCAATCAAAAAAAGATGTATTTAAATTTCGGTCTGTTAATAATATAGTAATAGCTCCGGCTAATACGGGTAGAGAAAGAAGTAATAAAATAGCTGTAATTACTACTGATCACACAAATAAAGGTAGTCGATCAAGAGTAATACCAGCTGATCGTATATTAATTACAGTTGTAATAAAATTTACTGCTCCTAAAATAGATGAGATTCCCGCTAAATGTAAAGAAAAAATTGCTAAATCAACTGAAGCCCCAGCATGAGCTGTTCCAGAAGAAAGGGGAGGATAAACCGTTCACCCTGTTCCAGCTCCGTTTTCTACTATAGAACTAGAAAGCAGCAGTGTTAAAGAGGGGGGTAATATTCAAAAACTTATATTATTTATTCGAGGAAAAGCTATATCAGGGGCTCCTAGTATTAAGGGAACTAATCAATTTCCAAAATCCTCCAA

>TTH_P_44F3

ACTGCCTAAATACACTCAATTTATCGTATGCATATCAAATAGGGTCTCCCCCTCCCATTGGATCAAAAAAAGATGTATTTAAATTTCGGTCTGTTAATAATATAGTAATAGCTCCGGCTAATACGGGTAGAGAAAGAAGTAATAAAATAGCTGTAATTACTACTGATCACACAAATAAAGGTAGTCGATCAAGAGTAATACCAGCTGATCGTATATTAATTACAGTTGTAATAAAATTTACTGCTCCTAAAATAGATGAGATTCCCGCTAAATGTAAAGAAAAAATTGCTAAATCAACTGAAGCCCCAGCATGAGCTGTTCCAGAAGAAAGGGGAGGATAAACCGTTCACCCTGTTCCAGCTCCGTTTTCTACTATAGAACTAGAAAGCAGCAGTGTTAAAGAGGGGGGTAATATTCAAAAACTTATATTATTTATTCGAGGAAAAGCTATATCAGGGGCTCCTAGTATTAAGGGAACTAATCAATTTCCAAAATCCTCC

>TTH_P_44F4

ACTGCTCCAACACTCAATTTCCATATCCTACCAAATAGGATCTCCCCCTCCAATTGAATCAAAAAAAGATGTATTTAAATTTAGGTCTGTTAATAATATAGTAATAGCTCCGGCTAATACGGGTAGAGAAAGAAGTAATAAAATAGCTGTAATTACTACTGATCACACAAATAAAGGTAGTCGATCAAGAGTAATACCAGCTGATCGTATATTAATTACAGTTGTAATAAAATTTACTGCTCCTAAAATAGATGAGATTCCCGCTAAATGTAGAGAAAAAATTGCTAAATCAACTGAAGCCCCAGCATGAGCTGTTCCAGAAGAAAGGGGAGGATAAACCGTTCACCCTGTTCCAGCTCCGTTTTCTACTATAGAACTAGAAAGCAGCAGTGTTAAAGAGGGGGGTAATATTCAAAAACTTATATTATTTATTCGAGGAAAAGCTATATCAGGGGCTCCTAGTATTAAGGGAACTAATCAATTTCCAAAATTCCTCCAA

>TTH_P_44F5

ACGCAAAGAAACAAATCAATTTATCGTATGATATCAAATAGGGTCTCCCCCTCCAATTGGATCAAAAAAAGATGTATTTAAATTTCGGTCTGTTAATAATATAGTAATAGCTCCGGCTAATACGGGTAGAGAAAGAAGTAATAAAATAGCTGTAATTACTACTGATCACACAAATAAAGGTAGTCGATCAAGAGTAATACCAGCTGATCGTATATTAATTACAGTTGTAATAAAATTTACTGCTCCTAAAATAGATGAGATTCCCGCTAAATGTAAAGAAAAAATTGCTAAATCAACTGAAGCCCCAGCATGAGCTGTTCCAGAAGAAAGGGGAGGATAAACCGTTCACCCTGTTCCAGCTCCGTTTTCTACTATAGAACTAGAAAGCAGCAGTGTTAAAGAGGGGGGTAATATTCAAAAACTTATATTATTTATTCGAGGAAAAGCTATATCAGGGGCTCCTAGTATTAAGGGAACTAATCAATTTCCAAAATCCTCCAA

>TTH_P_44F6

TATGGGTAAGTCAATTTACCTAACACTACTGAACTATCAATATTCCAAATCCTCCGAACTAATCAATTTCCAAATCCTCCGACCTCAACTATTTCCAAATCATCCGCTCTAGTTAATTTCGGTATCCACCGAAGTAATAAAATTGCTGTTATTACAACTGATCATACAAATAAAGGTAATCGGTCTAGAGTAATTCCTGCTGAACGGATATTAATTACTGTTGTAATAAAATTTACTGCTCCTAAAATTGACGAAATTCCTGCCAGATGTAAAGAAAAAATAGCTAAGTCAACAGAAGCCCCAGCATGAGCTGTTCCTGAAGAAGGAGGTGGGTAAACTGTTCATCCTGTACCAGCCCCATTTTCTACTATGGAGCTAGAAAGGAGAAGTGTTAATGAGGGGGGAGGTATTCAAAAACTTATATTATTTATTCGAGGAGAAGCTATATCAGGGGCCCCTAATATAAGGGGAACTAATCAATTTCCTAATCCTCCCCCCA

>TTH_KT_45M1

CTCTACGAACACTCAATTTCCGAATGCCTACCAAATAGGGTCTCCTCCTCCAATTGGATCAAAAAAAGATGTATTTAAATTTCGGTCTGTTAATAATATAGTAATAGCTCCGGCTAATACGGGTAGAGAAAGAAGTAATAAAATAGCTGTAATTACTACTGATCACACAAATAAAGGTAGTCGATCAAGAGTAATACCAGCTGATCGCATATTAATTACAGTTGTAATAAAATTTACTGCTCCTAAAATAGATGAGATTCCCGCTAAATGTAAAGAAAAAATTGCTAAATCAACTGAAGCCCCAGCATGAGCTGTTCCAGAAGAAAGGGGAGGATAAACCGTTCACCCTGTTCCAGCTCCGTTTTCTACTATAGAACTAGAAAGCAGCAGTGTTAAAGAGGGGGGTAATATTCAAAAACTTATATTATTTATTCGAGGAAAAGCTATATCAGGGGCTCCTAGTATTAAGGGAACTAATCAATTTCCAAAATCCTCCAA

>TTH_KT_45M2

CTCTACGACTACTCATTTCCAAATCCTACCCAACAGGGTCTCCCCCTCCCCTTGCAATCAAAAAAAGATGTATTTAAATTTCGGTCTGTTAATAATATAGTAATAGCTCCGGCTAATACGGGTAGAGAAAGAAGTAATAAAATAGCTGTAATTACTACTGATCACACAAATAAAGGTAGTCGATCAAGAGTAATACCAGCTGATCGTATATTAATTACAGTTGTAATAAAATTTACTGCTCCTAAAATAGATGAGATTCCCGCTAAATGTAAAGAAAAAATTGCTAAATCAACTGAAGCCCCAGCATGAGCTGTTCCAGAAGAAAGGGGAGGATAAACCGTTCACCCTGTTCCAGCTCCGTTTTCTACTATAGAACTAGAAAGCAGCAGTGTTAAAGAGGGGGGTAATATTCAAAAACTTATATTATTTATTCGAGGAAAAGCTATATCAGGGGCTCCTAGTATTAAGGGAACTAATCAATTTCAAAAATCCTCCCA

>TTH_KT_45M3

AAGCTCGTACTACTCAATTTATCATACAATATCCAATAGGGTCTCCCCCTCCCATTGCAATCAAAAAAAGATGTATTTAAATTTAGGTCTGTTAATAATATAGTAATAGCTCCGGCTAATACGGGTAGAGAAAGAAGTAATAAAATAGCTGTAATTACTACTGATCACACAAATAAAGGTAGTCGATCAAGAGTAATACCAGCTGATCGTATATTAATTACAGTTGTAATAAAATTTACTGCTCCTAAAATAGATGAGATTCCCGCTAAATGTAAAGAAAAAATTGCTAAATCAACTGAAGCCCCAGCATGAGCTGTTCCAGAAGAAAGGGGAGGATAAACCGTTCACCCTGTTCCAGCTCCGTTTTCTACTATAGAACTAGAAAGCAGCAGTGTTAAAGAGGGGGGTAATATTCAAAAACTTATATTATTTATTCGAGGAAAAGCTATATCAGGGGCTCCTAGTATTAAGGGAACTAATCAATTTCAAAAATCCTCCCA

>TTH_KT_45M4

AATCGTCGTTCTACTCACCCCATCTCCGATAATAACTAGTGTCTTTTCCAAATCCTTCGCGAACTAATCAAGTATTCCTTTTACTCCCGGTCTGTTAATAATATAGTAATAGCTCCGGCTAATACGGGTAGAGAAAGAAGTAATAAAATAGCTGTAATTACTACTGATCACACAAATAAAGGTAGTCGATCAAGAGTAATACCAGCTGATCGTATATTAATTACAGTTGTAATAAAATTTACTGCTCCTAAAATAGATGAGATTCCCGCTAAATGTAAAGAAAAAATTGCTAAATCAACTGAAGCCCCAGCATGAGCTGTTCCAGAAGAAAGGGGAGGATAAACCGTTCACCCTGTTCCAGCTCCGTTTTCTACTATAGAACTAGAAAGCAGCAGTGTTAAAGAGGGGGGTAATATTCAAAAACTTATATTATTTATTCGAGGAAAAGCTATATCAGGGGCTCCTAGTATTAAGGGAACTAATCAATTTCCAAATCCTCCCA

>TTH_KT_46F1

ATGCGTACTACTACTCATTTCCGTATCCTACCCAATAGGGTCTCCCCCTCCAATTGGATCAAAAAAAGATGTATTTAAATTTAGGTCTGTTAATAATATAGTAATAGCTCCGGCTAATACGGGTAGA

GAAAGAAGTAATAAAATAGCTGTAATTACTACTGATCACACAAATAAAGGTAGTCGATCAAGAGTAATACCAGCTGATCGTATATTAATTACAGTTGTAATAAAATTTACTGCTCCTAAAATAGATGAGATTCCCGCTAAATGTAAAGAAAAAATTGCTAAATCAACTGAAGCCCCAGCATGAGCTGTTCCAGAAGAAAGGGGAGGATAAACCGTTCACCCTGTTCCAGCTCCGTTTTCTACTATAGAACTAGAAAGCAGCAGTGTTAAAGAGGGGGGTAATATTCAAAAACTTATATTATTTATTCGAGGAAAAGCTATATCAGGGGCTCCTAGTATTAAGGGAACTAATCAATTTCAAAATCCTCCCA

>TTH_KT_46F2

ACTCTAGACTACTCAATTTCCATATGCCTACCAAATAGGGTCTCCCCCTCCAATTGGATCAAAAAAAGATGTATTTAAATTTCGGTCTGTTAATAATATAGTAATAGCTCCGGCTAATACGGGTAGAGAAAGAAGTAATAAAATAGCTGTAATTACTACTGATCACACAAATAAAGGCAGTCGATCAAGAGTAATACCAGCTGATCGTATATTAATTACAGTTGTAATAAAATTTACTGCTCCTAAAATAGATGAGATTCCCGCTAAATGTAAAGAAAAAATTGCTAAATCAACTGAAGCCCCAGCATGAGCTGTTCCAGAAGAAAGGGGAGGATAAACCGTTCACCCTGTTCCAGCTCCGTTTTCTACTATAGAACTAGAAAGCAGCAGTGTTAAAGAGGGGGGTAATATTCAAAAACTTATATTATTTATTCGAGGAAAAGCTATATCAGGGGCTCCTAGTATTAAGGGAACTAATCAATTTCCAAATCCTCC

>TTH_KT_46F3

TATGCGTCAGTATACGATTACCTAGATCCGTCATCAAATAGGATCTCCTCCTCCGATTGGATCAAAGAAAGATGTATTTAAGTTTCGGTCTGTTAATAACATAGTAATAGCTCCAGCTAAAACAGGAAGAGAAAGAAGTAATAAGATAGCTGTAATAACTACAGATCAAACAAATAAAGGTAGTCGATCTAAAGTAATTCCTGACGATCGTATATTAATTACAGTTGTAATAAAATTTACTGCCCCTAAAATTGAGGAAATTCCAGCTAAATGAAGAGAAAAAATAGCTAAATCAACAGAAGCTCCAGCATGAGCTGTTCCTGAAGAGAGAGGAGGATAAACTGTTCACCCAGTTCCTGCCCCATTTTCTACTATTGAGCTTGATAATAGAAGAGTCAATGAAGGAGGTAGTATTCAAAAACTTATATTATTTATTCGAGGAAAGGCTATATCAGGGGCTCCTAATATTAAAGGAACTAATCAATTTCCAAAATTCCCTCCAA

>TTH_KT_46F4

ATGCTAGTACTACTCATTTCCAAATCCTACCCAATAGGGTCTCCCCCTCCCATTGGATCAAAAAAAGATGTATTTAAATTTAGGTCTGTTAATAATATAGTAATAGCTCCGGCTAATACGGGTAGA

GAAAGAAGTAATAAAATAGCTGTAATTACTACTGATCACACAAATAAAGGTAGTCGATCAAGAGTAATACCAGCTGATCGTATATTAATTACAGTTGTAATAAAATTTACTGCTCCTAAAATAGATGAGATTCCCGCTAAATGTAAAGAAAAAATTGCTAAATCAACTGAAGCCCCAGCATGAGCTGTTCCAGAAGAAAGGGGAGGATAAACCGTTCACCCTGTTCCAGCTCCGTTTTCTACTATAGAACTAGAAAGCAGCAGTGTTAAAGAGGGGGGTAATATTCAAAAACTTATATTATTTATTCGAGGAAAAGCTATATCAGGGGCTCCTAGTATTAAGGGAACTAATCAATTTCCAAAATCCTCCCA

>TTH_KT_46F5

GCACTATCCGAATAGGGTCTCCCCCTCCAATTCGGATCAAAAAAAGATGTATTTAAATTTCGGTCTGTTAATAATATAGTAATAGCTCCGGCTAATACGGGTAGAGAAAGAAGTAATAAAATAGCTGTAATTACTACTGATCACACAAATAAAGGTAGTCGATCAAGAGTAATACCAGCTGATCGTATATTAATTACAGTTGTAATAAAATTTACTGCTCCTAAAATAGATGAGATTCCCGCTAAATGTAAAGAAAAAATTGCTAAATCAACTGAAGCCCCAGCATGAGCTGTTCCAGAAGAAAGGGGAGGATAAACCGTTCACCCTGTTCCAGCTCCGTTTTCTACTATAGAACTAGAAAGCAGCAGTGTTAAAGAGGGGGGTAATATTCAAAAACTTATATTATTTATTCGAGGAAAAGCTATATCAGGGGCTCCTAGTATTAAGGGAACTAATCAATTTCCAAATCCTCCCA

>TTH_KT_46F6

TCAAGATCAATAAGTGTTGGTATAAAATAGGGTCTCCCCCTCCGATTGGATCAAAGAAAGATGTATTTAAGTTTCGGTCTGTTAATAATATAGTAATAGCTCCCGCTAAAACAGGAAGAGAAAGAAGTAATAAGATAGCTGTAATAACTACAGATCAAACAAATAAGGGTAGTCGATCTAAAGTAATCCCTGACGATCGCATATTAATCACAGTTGTAATAAAATTTACTGCCCCTAAAATTGAGGAAATTCCAGCTAAATGAAGAGAAAAAATAGCTAAATCAACAGAAGCTCCAGCATGAGCTGTTCCTGAAGAGAGAGGAGGATAAACTGTTCACCCAGTTCCTGCCCCATTTTCTACTATGAGCTTGATAATAGAAGAGTCAATGAAGGAGGTAGTATTCAAAAACTTATATTATTCATTCGAGGGAAAGCTATATCAGGGGCTCCTAATATTAAAGGAACTAATCATTTCCAAATTCCCCCCAACA

>TTH_KT_46F7

CTCCTACGTACACTCAATTTCCGTATCCTAYCCAATAGGGTCTCCTCCTCCCATTGGATCAAAAAAAGATGTATTTAAATTTAGGTCTGTTAATAATATAGTAATAGCTCCGGCTAATACGGGTAGAGAAAGAAGTAATAAAATAGCTGTAATTACTACTGATCACACAAATAAAGGTAGTCGATCAAGAGTAATACCAGCTGATCGTATATTAATTACAGTTGTAATAAAATTTACTGCTCCTAAAATAGATGAGATTCCCGCTAAATGTAAAGAAAAAATTGCTAAATCAACTGAAGCCCCAGCATGAGCTGTTCCAGAAGAAAGGGGAGGATAAACCGTTCACCCTGTTCCAGCTCCGTTTTCTACTATAGAACTAGAAAGCAGCAGTGTTAAAGAGGGGGGTAATATTCAAAAACTTATATTATTTATTCGAGGAAAAGCTATATCAGGGGCTCCTAGTATTAAGGGAACTAATCAATTTCCAAAATCCTCCCA

>TTH_KT_47F2

TGCTAGCTACTCAATTTCCAAATSCGACCCAATAGGGTCTCCCCCTCCCCATCGAGATCAAAGAAAGATGTATTTCCGTTACGGTCTGTTAATAATATAGTAATAGCTCCCGCTAAAACAGGAAGAGAAAGAAGTAATAAGATAGCTGTAATAACTACCGATCAAACAAATAAGGGTAGTCGATCTAAAGTAATCCCTGACGATCGCATATTAATCACAGTTGTAATAAAATTTACTGCCCCTAAAATTGAGGAAATTCCAGCTAAATGAAGAGAAAAAATAGCTAAATCAACAGAAGCTCCAGCATGAGCTGTTCCTGAAGAGAGAGGAGGATAAACTGTTCACCCAGTTCCTGCCCCATTTTCTACTATTGAGCTTGATAATAGAAGAGTCAATGAAGGAGGTAGTATTCAAAAACTTATATTATTCATTCGAGGGAAAGCTATATCAGGGGCTCCTAATATTAAAGGAACTAATCAATTTCCAAATCCTCCAAA

>TTH_KT_47F3

CTCCTTCAACTATTCTAATTCTCCTGATGCGTCTCAATAGGGTCTCCCCCTCCGATTGAGATCAAAGAAAGATGTATTTAAGTTTCGGTCTGTTAATAATATAGTAATAGCTCCAGCTAAAACAGGAAGAGAAAGAAGTAATAAGATAGCTGTAATAACTACAGATCAAACAAATAAGGGTAGTCGATCTAAAGTAATCCCTGACGATCGCATATTAATCACAGTTGTAATAAAATTTACTGCCCCTAAAATTGAGGAAATTCCAGCTAAATGAAGAGAAAAAATAGCTAAATCAACAGAAGCTCCAGCATGAGCTGTTCCTGAAGAGAGAGGAGGATAAACTGTTCACCCAGTTCCTGCCCCATTTTCTACTATTGAGCTTGATAATAGAAGAGTCAATGAAGGAGGTAGTATTCAAAAACTTATATTATTCATTCGAGGGAAAGCTATATCAGGGGCTCCTAATATTAAAGGAACTAATCAATTTCCAAAATCCTCCAA

>TTH_L_49F1

ATGCTAGTACACTCAATTTACCGTATGCCTACCAAATAGGGTCTCCCCCTCCCATTGCGATCAAAAAAAGATGTATTTAAATTTAGGTCTGTTAATAATATAGTAATAGCTCCGGCTAATACGGGTAGAGAAAGAAGTAATAAAATAGCTGTAATTACTACTGATCACACAAATAAAGGTAGTCGATCAAGAGTAATACCAGCTGATCGTATATTAATTACAGTTGTAATAAAATTTACTGCTCCTAAAATAGATGAGATTCCCGCTAAATGTAGAGAAAAAATTGCTAAATCAACTGAAGCCCCAGCATGAGCTGTTCCAGAAGAAAGGGGAGGATAAACCGTTCACCCTGTTCCAGCTCCGTTTTCTACTATAGAACTAGAAAGCAGCAGTGTTAAAGAGGGGGGTAATATTCAAAAACTTATATTATTTATTCGAGGAAAAGCTATATCAGGGGCTCCTAGTATTAAGGGAACTAATCAATTTCCAAAATCCTCCCA

>TTH_L_49F2

ATCTCGTCTACTCAATTTCCAAATGCCTACCCAATAGGGTCTCCCCCTCCCATTGCGATCATAAAAAGATGTATTTAAATTTAGGTCTGTTAATAATATAGTAATAGCTCCGGCTAATACGGGTAGAGAAAGAAGTAATAAAATAGCTGTAATTACTACTGATCACACAAATAAAGGTAGTCGATCAAGAGTAATACCAGCTGATCGTATATTAATTACAGTTGTAATAAAATTTACTGCTCCTAAAATAGATGAGATTCCCGCTAAATGTAAAGAAAAAATTGCTAAATCAACTGAAGCCCCAGCATGAGCTGTTCCAGAAGAAAGGGGAGGATAAACCGTTCACCCTGTTCCAGCTCCGTTTTCTACTATAGAACTAGAAAGCAGCAGTGTTAAAGAGGGGGGTAATATTCAAAAACTTATATTATTTATTCGAGGAAAAGCTATATCAGGGGCTCCTAGTATTAAGGGAACTAATCAATTTCCAAAATCCTCCCA

>TTH_L_49F3

AATCTACGTGCTACTCATCTACGTATGATAACCAACTAGTGCTTTCTCCATATCCTTCGCGATACTAATCAAGTATGTCTTTAAATTCCGGTCTGTTAATAATATAGTAATACACTCCGGCTAATACGGGTAGAGAAAGAAGTAATAAAATAGCTGTAATTACTACTGATCACACAAATAAAGGTAGTCGATCAAGAGTAATACCAGCTGATCGTATATTAATTACAGTTGTAATAAAATTTACTGCTCCTAAAATAGATGAGATTCCCGCTAAATGTAAAGAAAAAATTGCTAAATCAACTGAAGCTCCAGCATGAGCTGTTCCAGAAGAAAGGGGAGGATAAACCGTTCACCCTGTTCCAGCTCCGTTTTCTACTATAGAACTAGAAAGCAGCAGTGTTAAAGAGGGGGGTAATATTCAAAAACTTATATTATTTATTCGAGGAAAAGCTATATCAGGGGCTCCTAGTATTAAGGGAACTAATCAATTTCCAAAATCCTCCCA

>TTH_L_49F4

CTACGTACTACTCAATTTCCAAATGCATACCGAACTAGTGACTTCTCCATATCCTTCGCGATCTAATCAGTATGTATTTAAATTTCGGTCTGTTAATAATATAGTAATAGCTCCGGCTAATACGGGTAGAGAAARAAGTAATAAAATAGCTGTAATTACTACTGATCACACAAATAAAGGTAGTCGATCAAGAGTAATACCAGCTGATCGTATATTAATTACAGTTGTAATAAAATTTACTGCTCCTAAAATAGATGAGATTCCCGCTAAATGTAAAGAAAAAATTGCTAAATCAACTGAAGCTCCAGCATGAGCTGTTCCAGAAGAAAGGGGAGGATAAACCGTTCACCCTGTTCCAGCTCCGTTTTCTACTATAGAACTAGAAAGCAGCAGTGTTAAAGAGGGGGGTAATATTCAAAAACTTATATTATTTATTCGAGGAAAAGCTATATCAGGGGCTCCTAGTATTAAGGGAACTAATCAATTTCAAAAATCCTCCCA

>TTH_L_49F5

ATGCTACGTACTACTCAATTTCCAATCCTACCGAATAGGGTCTCCTCCTCCCCTTGAATCAAAAAAAGATGTATTTAAATTTCGGTCTGTTAATAATATAGTAATAGCTCCGGCTAATACGGGTAGAGAAAGAAGTAATAAAATAGCTGTAATTACTACTGATCACACAAATAAAGGTAGTCGATCAAGAGTAATACCAGCTGATCGTATATTAATTACAGTTGTAATAAAATTTACTGCTCCTAAAATAGATGAGATTCCCGCTAAATGTAAAGAAAAAATTGCTAAATCAACTGAAGCCCCAGCATGAGCTGTTCCAGAAGAAAGGGGAGGATAAACCGTTCACCCTGTTCCAGCTCCGTTTTCTACTATAGAACTAGAAAGCAGCAGTGTTAAAGAGGGGGGTAATATTCAAAAACTTATATTATTTATTCGAGGAAAAGCTATATCAGGGGCTCCTAGTATTAAGGGAACTAATCAATTTCCAAAATCCTCCCA

>TTH_L_49F6

AAGCCTACAGACTACTCATCCTATCGTATGCATATCCAATAGGGTCTCCCCCTCCCATTGGATCAAAAAAAGATGTATTTAAATTTCGGTCTGTTAATAATATAGTAATAGCTCCGGCTAATACGGGTAGAGAAAGAAGTAATAAAATAGCTGTAATTACTACTGATCACACAAATAAAGGTAGTCGATCAAGAGTAATACCAGCTGATCGTATATTAATTACAGTTGTAATAAAATTTACTGCTCCTAAAATAGATGAGATTCCCGCTAAATGTAAAGAAAAAATTGCTAAATCAACTGAAGCTCCAGCATGAGCTGTTCCAGAAGAAAGGGGAGGATAAACCGTTCACCCTGTTCCAGCTCCGTTTTCTACTATAGAACTAGAAAGCAGCAGTGTTAAAGAGGGGGGTAATATTCAAAAACTTATATTATTTATTCGAGGAAAAGCTATATCAGGGGCTCCTAGTATTAAGGGAACTAATCAATTTCAAAAATCCTCCCA

>TTH_L_49F7

ACTCGCTGAAGACTTATAAACATCAACCACCCAGGAAGGGCCTTCCCCCCCCTTGAGGAAAAAAATATGTCTTATTCTCTGTGGGTAAAAAAAAAAAAACTCCCGCTAAACGGGAAAAGAAAAATAAAAAAAACTTTTTATAGTTTTGACGATAAGGTGTGATCAAAAAAACCCCCTGGCATTTATTAAATTGGATAAATTTTGGCTCCAAAAAAAAAAATTCCCCAAATGAAAAAAATTTGCTAAACTCTGACCCAAGATAACCTTTCTAAAGAGGGGAAAAAAGTTTCCCCCTTCCCCTTTTTCTTTTTAATTAAAAAAATTGTAGAAAGGTGAGATTCATATTATTTATTTCCGGAAAACTATTGGGGGCGCTTTTGGGGGATATCTTTCCTCTCCCCAACC

>TTH_L_49F8

ATCTACGTGCTAACTCAATCCTCCTCCAATCCCAATTAGGACTTCTCCAAATCCTCGCAATACTAATCATTATTCCATATCCTCCCGGTCAGTTAATAATATAGTAATAGACTCCGGCTAATACGGGTAGAGAAAGAAGTAATAAAATAGCTGTAATTACTACTGATCACACAAATAAAGGTAGTCGATCAAGAGTAATACCAGCTGATCGTATATTAATTACAGTTGTAATAAAATTTACTGCTCCTAAAATAGATGAGATTCCCGCTAAATGTAAAGAAAAAATTGCTAAATCAACTGAAGCTCCAGCATGAGCTGTTCCAGAAGAAAGGGGAGGATAAACCGTTCACCCTGTTCCAGCTCCGTTTTCTACTATAGAACTAGAAAGCAGCAGTGTTAAAGAGGGGGGTAATATTCAAAAACTTATATTATTTATTCGAGGAAAAGCTATATCAGGGGCTCCTAGTATTAAGGGAACTAATCAATTTCCAAAATCCTCCCA

>TTH_L_49F9

ATCTACAACTACTCAATTTCCATATCCTACCCAATAGGGTCTCCCCCTCCAATTGGATCAAAAAAAGATGTATTTAAATTTAGGTCTGTTAATAATATAGTAATAGCTCCGGCTAATACGGGTAGAGAAAGAAGTAATAAAATAGCTGTAATTACTACTGATCACACAAATAAAGGTAGTCGATCAAGAGTAATACCAGCTGATCGTATATTAATTACAGTTGTAATAAAATTTACTGCTCCTAAAATAGATGAGATTCCCGCTAAATGTAAAGAAAAAATTGCTAAATCAACTGAAGCTCCAGCATGAGCTGTTCCAGAAGAAAGGGGAGGATAAACCGTTCACCCTGTTCCAGCTCCGTTTTCTACTATAGAACTAGAAAGCAGCAGTGTTAAAGAGGGGGGTAATATTCAAAAACTTATATTATTTATTCGAGGAAAAGCTATATCAGGGGCTCCTAGTATTAAGGGAACTAATCAATTTCAAAAATCCTCCCA

>TTH_L_49F10

AAGCGTCGTTCTAATCATTTCCAAATCCTACCCAACTAGTGAATTTCCAAATCCTCGCGAACTAATCAGATTCCTTATCCTCCCGGTCTGTTAATAATATAGTAATAGCTCCGGCTAATACGGGTAGAGAAAGAAGTAATAAAATAGCTGTAATTACTACTGATCACACAAATAAAGGTAGTCGATCAAGAGTAATACCAGCTGATCGTATATTAATTACAGTTGTAATAAAATTTACTGCTCCTAAAATAGATGAGATTCCCGCTAAATGTAAAGAAAAAATTGCTAAATCAACTGAAGCTCCAGCATGAGCTGTTCCAGAAGAAAGGGGAGGATAAACCGTTCACCCTGTTCCAGCTCCGTTTTCTACTATAGAACTAGAAAGCAGCAGTGTTAAAGAGGGGGGTAATATTCAAAAACTTATATTATTTATTCGAGGAAAAGCTATATCAGGGGCTCCTAGTATTAAGGGAACTAATCAATTTCCAAATCCTCCCAAA

>TTH_L_49F11

CTGCTACGAACTACTCATTTCCGTATCCTACCCAATAGGGTCTCCTCCTCCAATTGGATCAAAAAAAGATGTATTTAAATTTAGGTCTGTTAATAATATAGTAATAGCTCCGGCTAATACGGGTAGAGAAAGAAGTAATAAAATAGCTGTAATTACTACTGATCACACAAATAAAGGTAGTCGATCAAGAGTAATACCAGCTGATCGTATATTAATTACAGTTGTAATAAAATTTACTGCTCCTAAAATAGATGAGATTCCCGCTAAATGTAAAGAAAAAATTGCTAAATCAACTGAAGCCCCAGCATGAGCTGTTCCAGAAGAAAGGGGGGGATAAACCGTTCACCCTGTTCCAGCTCCGTTTTCTACTATAGAACTAGAAAGCAGCAGTGTTAAAGAGGGGGGTAATATTCAAAAACTTATATTATTTATTCGAGGAAAAGCTATATCAGGGGCTCCTAGTATTAAGGGAACTAATCAATTTCCAAAATCCTCCCA

>TTH_L_49F12

ATCGTACGTACTACTCAATTTACCAAATGCATATCCAATAGGGTCTCCCCCTCCCCTTCGCGATCATAAAAAGATGTATTTAAATTTAGGTCTGTTAATAATATAGTAATAGCTCCGGCTAATACGGGTAGAGAAAGAAGTAATAAAATAGCTGTAATTACTACTGATCACACAAATAAAGGTAGTCGATCAAGAGTAATACCAGCCGATCGTATATTAATTACAGTTGTAATAAAATTTACTGCTCCTAAAATAGATGAGATTCCCGCTAAATGTAAAGAAAAAATTGCTAAATCAACTGAAGCCCCAGCATGAGCTGTTCCAGAAGAAAGGGGAGGATAAACCGTTCACCCTGTTCCAGCTCCGTTTTCTACTATAGAACTAGAAAGCAGCAGTGTTAAAGAGGGGGGTAATATTCAAAAACTTATATTATTTATTCGAGGAAAAGCTATATCAGGGGCTCCTAGTATTAAGGGAACTAATCAATTTCCAAAATCCTCCCA

>TTH_L_49F13

GTCCTTTCCCAATGTGTACTCTGGTTCTTTCCAAATCCTTCAATCTAATCAGATTTCTTTAAATTCCGGTCTGTTAATAATATAGTAATAGCTCCGGCTAATACGGGTAGAGAAAGAAGTAATAAAATAGCTGTAATTACTACTGATCACACAAATAAAGGTAGTCGATCAAGAGTAATACCAGCTGATCGTATATTAATTACAGTTGTAATAAAATTTACTGCTCCTAAAATAGATGAGATTCCCGCTAAATGTAAAGAAAAAATTGCTAAATCAACTGAAGCCCCAGCATGAGCTGTTCCAGAAGAAAGGGGAGGATAAACCGTTCACCCTGTTCCAGCTCCGTTTTCTACTATAGAACTAGAAAGCAGCAGTGTTAAAGAGGGGGGTAATATTCAAAAACTTATATTATTTATTCGAGGAAAAGCTATATCAGGGGCTCCTAGTATTAAGGGAACTAATCAATTTCCAAATCCTCCCA

>TTH_L_50M1

ACGCTTAGTACTACTTATCCACTCCGCTTACAATAGGGTCTCCCCCTCCCCTTGCGATCAAAAAAAGATGTATTTAAATTTCGGTCTGTTAATAATATAGTAATAGCTCCGGCTAATACGGGTAGAGAAAGAAGTAATAAAATAGCTGTAATTACTACTGATCACACAAATAAAGGTAGTCGATCAAGAGTAATACCAGCTGATCGTATATTAATTACAGTTGTAATAAAATTTACTGCTCCTAAAATAGATGAGATTCCCGCTAAATGTAAAGAAAAAATTGCTAAATCAACTGAAGCTCCAGCATGAGCTGTTCCAGAAGAAAGGGGAGGATAAACCGTTCACCCTGTTCCAGCTCCGTTTTCTACTATAGAACTAGAAAGCAGCAGTGTTAAAGAGGGGGGTAATATTCAAAAACTTATATTATTTATTCGAGGAAAAGCTATATCAGGGGCTCCTAGTATTAAGGGAACTAATCAATTTCCAAATCCTCCCA

>TTH_L_50M2

ATGCTCGTACACTCATTAATCATATGCATATCCAATAGGGTCTCCCCCTCCAATTGGATCAAAAAAAGATGTATTTAAATTTCGGTCTGTTAATAATATAGTAATAGCTCCGGCTAATACGGGTAGAGAAAGAAGTAATAAAATAGCTGTAATTACTACTGATCACACAAATAAAGGTAGTCGATCAAGAGTAATACCAGCTGATCGTATATTAATTACAGTTGTAATAAAATTTACTGCTCCTAAAATAGATGAGATTCCCGCTAAATGTAAAGAAAAAATTGCTAAATCAACTGAAGCTCCAGCATGAGCTGTTCCAGAAGAAAGGGGAGGATAAACCGTTCACCCTGTTCCAGCTCCGTTTTCTACTATAGAACTAGAAAGCAGCAGTGTTAAAGAGGGGGGTAATATTCAAAAACTTATATTATTTATTCGAGGAAAAGCTATATCAGGGGCTCCTAGTATTAAGGGAACTAATCAATTTCCAAATTCCTCACA

>TTH_L_50M3

CTCTAGAACTAATCATTTCCAAATCCTACCCAATAGGGTCTCCCCCTCCCATTGGATCAAAAAAAGATGTATTTAAATTTAGGTCTGTTAATAATATAGTAATAGCTCCGGCTAATACGGGTAGAGAAAGAAGTAATAAAATAGCTGTAATTACTACTGATCACACAAATAAAGGTAGTCGATCAAGAGTAATACCAGCCGATCGTATATTAATTACAGTTGTAATAAAATTTACTGCTCCTAAAATAGATGAGATTCCCGCTAAATGTAAAGAAAAAATTGCTAAATCAACTGAAGCCCCAGCATGAGCTGTTCCAGAAGAAAGGGGAGGATAAACCGTTCACCCTGTTCCAGCTCCGTTTTCTACTATAGAACTAGAAAGCAGCAGTGTTAAAGAGGGGGGTAATATTCAAAAACTTATATTATTTATTCGAGGAAAAGCTATATCAGGGGCTCCTAGTATTAAGGGAACTAATCAATTTCAAAAATCCTCCCA

>TTH_L_50M4

AGCGTAGAACTACTCAATTCACCGTACGATATCCAATAGGGTCTCCCCCTTCCCTTGGATCAAAAAAAGATGTATTTAACTTTCGGTCTGTTAATAATATAGTAATAGCTCCGGCTAATACGGGTAGAGAAAGAAGTAATAAAATAGCTGTAATTACTACTGATCACACAAATAAGGGTAGTCGATCAAGAGTAATACCAGCTGATCGTATATTAATTACAGTTGTAATAAAATTTACTGCTCCTAAAATAGATGAGATTCCCGCTAAATGTAAAGAAAAAATTGCTAAATCAACTGAAGCCCCAGCATGAGCTGTTCCAGAAGAAAGGGGAGGATAAACCGTTCACCCTGTTCCAGCTCCGTTTTCTACTATAGAACTAGAAAGCAGCAGTGTTAAAGAGGGGGGTAATATTCAAAAACTTATATTATTTATTCGAGGAAAAGCTATATCAGGGGCTCCTAGTATTAAGGGAACTAATCAATTTCAAAAATCCTCCCA

>TTH_L_50M5

CGCTAAGTACTACTCAATTTATCATATGCCTATCCAATAGGGTCTCCCCCTCCAATTGGATCAAAAAAAGATGTATTTAAATTTCGGTCTGTTAATAATATAGTAATAGCTCCGGCTAATACGGGTAGAGAAAGAAGTAATAAAATAGCTGTAATTACTACTGATCACACAAATAAAGGTAGTCGATCAAGAGTAATACCAGCCGATCGTATATTAATTACAGTTGTAATAAAATTTACTGCTCCTAAAATAGATGAGATTCCCGCTAAATGTAAAGAAAAAATTGCTAAATCAACTGAAGCCCCAGCATGAGCTGTTCCAGAAGAAAGGGGAGGATAAACCGTTCACCCTGTTCCAGCTCCGTTTTCTACTATAGAACTAGAAAGCAGCAGTGTTAAAGAGGGGGGTAATATTCAAAAACTTATATTATTTATTCGAGGAAAAGCTATATCAGGGGCTCCTAGTATTAAGGGAACTAATCAATTTCAAAATCCTCCCA

>TTH_L_50M6

ATGCGGCGTGCTACTCAATTCATCGTCCGCATTATAATAGGGTCTCCCCCTCCCCTTGCGATCATAAAAAGATGTATTTAAATTTCGGTCTGTTAATAATATAGTAATAGCTCCGGCTAATACGGGTAGAGAAAGAAGTAATAAAATAGCTGTAATTACTACTGATCACACAAATAAAGGTAGTCGATCAAGAGTAATACCAGCCGATCGTATATTAATTACAGTTGTAATAAAATTTACTGCTCCTAAAATAGATGAGATTCCCGCTAAATGTAAAGAAAAAATTGCTAAATCAACTGAAGCCCCAGCATGAGCTGTTCCAGAAGAAAGGGGAGGATAAACCGTTCACCCTGTTCCAGCTCCGTTTTCTACTATAGAACTAGAAAGCAGCAGTGTTAAAGAGGGGGGTAATATTCAAAAACTTATATTATTTATTCGAGGAAAACTATATCAGGGGCTCCTAGTATTAAGGGAACTAATCAATTTCAAAAATCCTCCCA

>TTH_L_50M7

AGCTCGTCTACTTATTCATCGTCCAATTATCAATAGGGTCTCCCCCTCCCATTGGATCAAAAAAAGATGTATTTAAATTTCGGTCTGTTAATAATATAGTAATAGCTCCGGCTAATACGGGTAGAGAAAGAAGTAATAAAATAGCTGTAATTACTACTGATCACACAAATAAGGGTAGTCGATCAAGAGTAATACCAGCTGATCGTATATTAATTACAGTTGTAATAAAATTTACTGCTCCTAAAATAGATGAGATTCCCGCTAAATGTAAAGAAAAAATTGCTAAATCAACTGAAGCCCCAGCATGAGCTGTTCCAGAAGAAAGGGGAGGATAAACCGTTCACCCTGTTCCAGCTCCGTTTTCTACTATAGAACTAGAAAGCAGCAGTGTTAAAGAGGGGGGTAATATTCAAAAACTTATATTATTTATTCGAGGAAAAGCTATATCAGGGGCTCCTAGTATTAAGGGAACTAATCAATTTCAAAATCCTC

>TTH_L_50M8

TCGTAGCTACTCAATTTCCAAATCATACCGGACTAATCAATTTCCAAATCCTCCAAACTAATCAATTTCCAAATCCTCCGAACTAATCAATTTCCAAATCCTCCGATCTAGTTAATCACGGTATACACAAAAGTAATAAAATTGCTGTTATTACAACTGATCACACAAATAGGGGTAGTCGGTCTAGAGTAATTCCTGCTGATCGTATATTAATTACTGGTGTAATAAAATTTACTGCCCCTAAAATTGATGAAATTCCTGCTAGATGTAAAGAAAAAATTGCTAAGTCAACTGAAGCCCCAGCATGAGCTGTTCCTGAAGAGAGGGGAGGGTAAACTGTTCATCCTGTACCAGCTCCGTTTTCTACTATGGAGCTAGAAAGGAGAAGTGTTAATGAGGGGGGAARTATTCAAAAACTTATATTATTTATTCGAGGAAAAGTTATATCAGGGGCTCCTAGTATAAAGGGAACTAATCAATTTCCAAATACATCCAAGGT

>TTH_L_50M9

CGTTCGAGGGAACTAAATCAATTAACCATACGCATACCGAATAGGGTCTCCCCCTCCAATTGGATCAAAAAAAGATGTATTTAAATTTMGGTCTGTTAATAATATAGTAATAGCTCCGGCTAATACGGGTAGAGAAAGAAGTAATAAAATAGCTGTAATTACTACTGATCACACAAATAAAGGTAGTCGATCAAGAGTAATACCAGCCGATCGTATATTAATTACAGTTGTAATAAAATTTACTGCTCCTAAAATAGATGAGATTCCCGCTAAATGTAAAGAAAAAATTGCTAAATCAACTGAAGCCCCAGCATGAGCTGTTCCAGAAGAAAGGGGAGGATAAACCGTTCACCCTGTTCCAGCTCCGTTTTCTACTATAGAACTAGAAAGCAGCAGTGTTAAAGAGGGGGGTAATATTCAAAAACTTATATTATTTATTCGAGGAAAAGCTATATCAGGGGCTCCTAGTATTAAGGGAACTAATCAATTTCCAAATCCT

>TTH_L_50M11

AGGCGTGCGTACTACTCAATTTCCGTATGATACCGAACTAGTGTCTYCYCCATCTCACTTCGCGATACATAAAAAGTATGTATTTTACTTTCGGTCAGTTAATAATATAGTAATAGCTCCGGCTAATACGGGTAGAGAAAGAAGTAATAAAATAGCTGTAATTACTACTGATCACACAAATAAAGGTAGTCGATCAAGAGTAATACCAGCCGATCGTATATTAATTACAGTTGTAATAAAATTTACTGCTCCTAAAATAGATGAGATTCCCGCTAAATGTAAAGAAAAAATTGCTAAATCAACTGAAGCCCCAGCATGAGCTGTTCCAGAAGAAAGGGGAGGATAAACCGTTCACCCTGTTCCAGCTCCGTTTTCTACTATAGAACTAGAAAGCAGCAGTGTTAAAGAGGGGGGTAATATTCAAAAACTTATATTATTTATTCGAGGAAAAGCTATATCAGGGGCTCCTAGTATTAAGGGAACTAATCAATTTCCAAAATCCTCCC

>TTH_L_50M12

CGGGACTATCCTCAATAGTGTACTCCCCCTCCCACTTCGCAATACAAAAAAAGATTGTATTTAAATTTACGGTCTGTTAATAATATAGTAATAGCTCCGGCTAATACGGGTAGAGAAAGAAGTAATAAAATAGCTGTAATTACTACTGATCACACAAATAAAGGTAGTCGATCAAGAGTAATACCAGCTGATCGTATATTAATTACAGTTGTAATAAAATTTACTGCCCCTAAAATAGATGAGATTCCCGCTAAATGTAAAGAAAAAATTGCTAAATCAACTGAAGCCCCAGCATGAGCTGTTCCAGAAGAAAGGGGAGGATAAACCGTTCACCCTGTTCCAGCTCCGTTTTCTACTATAGAACTAGAAAGCAGCAGTGTTAAAGAGGGGGGTAATATTCAAAAACTTATATTATTTATTCGAGGAAAAGCTATATCAGGGGCTCCTAGTATTAAGGGAACTAATCAATTTCAAAAATCCTCCCA

>TTH_L_50M13

AGCATACRACTACTCAATTTCCATATCCTACCCAATAGGGTCTCCCCCTCCCCTTGCAATCAAAAAAAGATGTATTTAAATTTCGGTCTGTTAATAATATAGTAATAGCTCCGGCTAATACGGGTAGAGAAAGAAGTAATAAAATAGCTGTAATTACTACTGATCACACAAATAAAGGTAGTCGATCAAGAGTAATACCAGCTGATCGTATATTAATTACAGTTGTAATAAAATTTACTGCTCCTAAAATAGATGAGATTCCCGCTAAATGTAAAGAAAAAATTGCTAAATCAACTGAAGCCCCAGCATGAGCTGTTCCAGAAGAAAGGGGAGGATAAACCGTTCACCCTGTTCCAGCTCCGTTTTCTACTATAGAACTAGAAAGCAGCAGTGTTAAAGAGGGGGGTAATATTCAAAAACTTATATTATTTATTCGAGGAAAAGCTATATCAGGGGCTCCTAGTATTAAGGGAACTAATCAATTTCCAAATCCTCCCA

>TTH_L_50M14

AGGGCATTCTACTTATCCACTCCGACTCCCAATAAGSACTACCCCTATCCTCGCAATCAAAAAAAGATGCCTTTAACTTTCGGTCTGTTAATAATATAGTAATAGCTCCGGCTAATACGGGTAGAGAAAGAAGTAATAAAATAGCTGTAATTACTACTGATCACACAAATAAAGGTAGTCGATCAAGAGTAATACCAGCTGATCGTATATTAATTACAGTTGTAATAAAATTTACTGCTCCTAAAATAGATGAGATTCCCGCTAAATGTAAAGAAAAAATTGCTAAATCAACTGAAGCTCCAGCATGAGCTGTTCCAGAAGAAAGGGGAGGATAAACCGTTCACCCTGTTCCAGCTCCGTTTTCTACTATAGAACTAGAAAGCAGCAGTGTTAAAGAGGGGGGTAATATTCAAAAACTTATATTATTTATTCGAGGAAAAGCTATATCAGGGGCTCCTAGTATTAAGGGAACTAATCAATTTCCAAATCCTCCCACA

>QT_LB_53M1

AATGCGGTCAAGTATCACGTTAACGTGTTCCGTCTAAAATAGGATCTCCTCCTCCGATTGAGATCAAAGAAAGATGTATTTAAGTTTCGGTCTGTTAATAACATAGTAATAGCTCCAGCTAAAACAGGAAGAGAAAGAAGTAATAAGATAGCTGTAATAACTACAGATCAAACAAATAAAGGTAGTCGATCTAAAGTAATTCCTGACGATCGTATATTAATTACAGTTGTAATAAAATTTACTGCCCCTAAAATTGAGGAAATTCCAGCTAAATGAAGAGAAAAAATAGCTAAATCAACAGAAGCTCCAGCATGAGCTGTTCCTGAAGAGAGAGGAGGATAAACTGTTCACCCAGTTCCTGCCCCATTTTCTACTATTGAGCTTGATAATAGAAGAGTCAATGAAGGAGGTAGTATTCAAAAACTTATATTATTTATTCGAGGAAAGGCTATATCAGGGGCTCCTAATATTAAAGGAACTAATCAATTCCAAATTCCTCCA

>QT_LB_53M2

GATGCGGTCAGTATCACGATTTACCTGTTCCGTCCAAATAGGATCTCCTCCTCCGATTGGATCAAAGAAAGATGTATTTAAGTTTCGGTCTGTTAATAACATAGTAATAGCTCCAGCTAAAACAGGAAGAGAAAGAAGTAATAAGATAGCTGTAATAACTACAGATCAAACAAATAAAGGTAGTCGATCTAAAGTAATTCCTGACGATCGTATATTAATTACAGTTGTAATAAAATTTACTGCCCCTAAAATTGAGGAAATTCCAGCTAAATGAAGAGAAAAAATAGCTAAATCAACAGAAGCTCCAGCATGAGCTGTTCCTGAAGAGAGAGGAGGATAAACTGTTCACCCAGTTCCTGCCCCATTTTCTACTATTGAGCTTGATAATAGAAGAGTCAATGAAGGAGGTAGTATTCAAAAACTTATATTATTTATTCGAGGAAAGGCTATATCAGGGGCTCCTAATATTAAAGGAACTAATCAATTTCCAAATTCCCCCCCA

>QT_LB_53M3

ACCTGCGTCAAGGTATCAGATTAACGTGTTCCGTATAAAATAGGATCTCCTCCTCCGATTGGATCAAAGAAAGATGTATTTAAGTTTCGGTCTGTTAATAACATAGTAATAGCTCCAGCTAAAACAGGAAGAGAAAGAAGTAATAAGATAGCTGTAATAACTACAGATCAAACAAATAAAGGTAGTCGATCTAAAGTAATTCCTGACGATCGTATATTAATTACAGTTGTAATAAAATTTACTGCCCCTAAAATTGAGGAAATTCCAGCTAAATGAAGAGAAAAAATAGCTAAATCAACAGAAGCTCCAGCATGAGCTGTTCCTGAAGAGAGAGGAGGATAAACTGTTCACCCAGTTCCTGCTCCATTTTCTACTATTGAGCTTGATAATAGAAGAGTCAATGAAGGAGGTAGTATTCAAAAACTTATATTATTTATTCGAGGAAAGGCTATATCAGGGGCTCCTAATATTAAAGGAACTAATCAATTTCCAAATTCCTCCA

>QT_LB_53M4

GCATGCCGTCAAGTTCAGATTAACCTGTTCCGTATAAAATAGGATCTCCTCCTCCGATTGGATCAAAGAAAGATGTATTTAAGTTTCGGTCTGTTAATAACATAGTAATAGCTCCAGCTAAAACAGGAAGAGAAAGAAGTAATAAGATAGCTGTAATAACTACAGATCAAACAAATAAAGGTAGTCGATCTAAAGTAATTCCTGACGATCGTATATTAATTACAGTTGTAATAAAATTTACTGCCCCTAAAATTGAGGAAATTCCAGCTAAATGAAGAGAAAAAATAGCTAAATCAACAGAAGCTCCAGCATGAGCTGTTCCTGAAGAGAGAGGAGGATAAACTGTTCACCCAGTTCCTGCCCCATTTTCTACTATTGAGCTTGATAATAGAAGAGTCAATGAAGGAGGTAGTATTCAAAAACTTATATTATTTATTCGAGGAAAGGCTATATCAGGGGCTCCTAATATTAAAGGAACTAATCAATTTCCAAATCCCTCCA

>QT_LB_53M5

CCTGCCGTCAGTATCAGATTACGTGTTCCGTATAAAATAGGATCTCCTCCTCCGATTGGATCAAAGAAAGATGTATTTAAGTTTCGGTCTGTTAATAACATAGTAATAGCTCCAGCTAAAACAGGAAGAGAAAGAAGTAATAAGATAGCTGTAATAACTACAGATCAAACAAATAAAGGTAGTCGATCTAAAGTAATTCCTGACGATCGTATATTAATTACAGTTGTAATAAAATTTACTGCCCCTAAAATTGAGGAAATTCCAGCTAAATGAAGAGAAAAAATAGCTAAATCAACAGAAGCTCCAGCATGAGCTGTTCCTGAAGAGAGAGGAGGATAAACTGTTCACCCAGTTCCTGCCCCATTTTCTACTATTGAGCTTGATAATAGAAGAGTCAATGAAGGAGGTAGTATTCAAAAACTTATATTATTTATTCGAGGAAAGGCTATATCAGGGGCTCCTAATATTAAAGGAACTAATCAATTTCCAAATTCCTCCA

>QT_LB_54F1

CCTTCCGTCAGTATCAGTTAACCTGATCCGTATAAATAGGATCTCCTCCTCCCATTGGATCAAAGAAAGATGTATTTAAGTTTCGGTCTGTTAATAACATAGTAATAGCTCCAGCTAAAACAGGAAGAGAAAGAAGTAATAAGATAGCTGTAATAACTACAGATCAAACAAATAAAGGTAGTCGATCTAAAGTAATTCCTGACGATCGTATATTAATTACAGTTGTAATAAAATTTACTGCCCCTAAAATTGAGGAAATTCCAGCTAAATGAAGAGAAAAAATAGCTAAATCAACAGAAGCTCCAGCATGAGCTGTTCCTGAAGAGAGAGGAGGATAAACTGTTCACCCAGTTCCTGCCCCATTTTCTACTATTGAGCTTGATAATAGAAGAGTCAATGAAGGAGGTAGTATTCAAAAACTTATATTATTTATTCGAGGAAAGGCTATATCAGGGGCTCCTAATATTAAAGGAACTAATCAATTTCCAAATTCCTCCA

>QT_LB_54F2

CGGTCAAGTATCACGTTAACCTGTTCCGTCATAAAATAGGATCTCCTCCTCCGATTGGATCAAAGAAAGATGTATTTAAGTTTCGGTCTGTTAATAACATAGTAATAGCTCCAGCTAAAACAGGAAGAGAAAGAAGTAATAAGATAGCTGTAATAACTACAGATCAAACAAATAAAGGTAGTCGATCTAAAGTAATTCCTGACGATCGTATATTAATTACAGTTGTAATAAAATTTACTGCCCCTAAAATTGAGGAAATTCCAGCTAAATGAAGAGAAAAAATAGCTAAATCAACAGAAGCTCCAGCATGAGCTGTTCCTGAAGAGAGAGGAGGATAAACTGTTCACCCAGTTCCTGCCCCATTTTCTACTATTGAGCTTGATAATAGAAGAGTCAATGAAGGAGGTAGTATTCAAAAACTTATATTATTTATTCGAGGAAAGGCTATATCAGGGGCTCCTAATATTAAAGGAACTAATCAATTTCAAATTCCCCCCA

>QT_LB_54F3

TCAGTATCACGATTACCTAGATGCGTCATAAATAGGATCTCCTCCTCCGATTGGATCAAAGAAAGATGTATTTAAGTTTCGGTCTGTTAATAACATAGTAATAGCTCCAGCTAAAACAGGAAGAGAAAGAAGTAATAAGATAGCTGTAATAACTACAGATCAAACAAATAAAGGTAGTCGATCTAAAGTAATTCCTGACGATCGTATATTAATTACAGTTGTAATAAAATTTACTGCCCCTAAAATTGAGGAAATTCCAGCTAAATGAAGAGAAAAAATAGCTAAATCAACAGAAGCTCCAGCATGAGCTGTTCCTGAAGAGAGAGGAGGATAAACTGTTCACCCAGTTCCTGCCCCATTTTCTACTATTGAGCTTGATAATAGAAGAGTCAATGAAGGAGGTAGTATTCAAAAACTTATATTATTTATTCGAGGAAAGGCTATATCAGGGGCTCCTAATATTAAAGGAACTAATCAATTTCCAAAATTCCCTCCA

>QT_LB_54F4

GTCAGTATCCGATAACCTGATCCGTCTAAAATAGGATCTCCTCCTCCGATTGAGATCAAAGAAAGATGTATTTCAGTTTCGGTCTGTTAATAACATAGTAATAGCTCCAGCTAAAACAGGAAGAGAAAGAAGTAATAAGATAGCTGTAATAACTACAGATCAAACAAATAAAGGTAGTCGATCTAAAGTAATTCCTGACGATCGTATATTAATTACAGTTGTAATAAAATTTACTGCCCCTAAAATTGAGGAAATTCCAGCTAAATGAAGAGAAAAAATAGCTAAATCAACAGAAGCTCCAGCATGAGCTGTTCCTGAAGAGAGAGGAGGATAAACTGTTCACCCAGTTCCTGCCCCATTTTCTACTATTGAGCTTGATAATAGAAGAGTCAATGAAGGAGGTAGTATTCAAAAACTTATATTATTTATTCGAGGAAAGGCTATATCAGGGGCTCCTAATATTAAAGGAACTAATCAATTTCCAAAATCCCTCCA

>QT_LB_54F5

CCGTCAGTATCAGATTAACGTGATCCGTATAAAATAGGATCTCCTCCTCCGATTGGATCAAAGAAAGATGTATTTAAGTTTCGGTCTGTTAATAACATAGTAATAGCTCCAGCTAAAACAGGAAGAGAAAGAAGTAATAAGATAGCTGTAATAACTACAGATCAAACAAATAAAGGTAGTCGATCTAAAGTAATTCCTGACGATCGTATATTAATTACAGTTGTAATAAAATTTACTGCCCCTAAAATTGAGGAAATTCCAGCTAAATGAAGAGAAAAAATAGCTAAATCAACAGAAGCTCCAGCATGAGCTGTTCCTGAAGAGAGAGGAGGATAAACTGTTCACCCAGTTCCTGCCCCATTTTCTACTATTGAGCTTGATAATAGAAGAGTCAATGAAGGAGGTAGTATTCAAAAACTTATATTATTTATTCGAGGAAAGGCTATATCAGGGGCTCCTAATATTAAAGGAACTAATCAATTTCCAAATTCCCTCCA

>QT_LB_54F6

CGGTCAGTATCAGATTACGTGTTCCGTATAAAATAGGATCTCCTCCTCCGATTGGATCAAAGAAAGATGTATTTAAGTTTCGGTCTGTTAATAACATAGTAATAGCTCCAGCTAAAACAGGAAGAGAAAGAAGTAATAAGATAGCTGTAATAACTACAGATCAAACAAATAAAGGTAGTCGATCTAAAGTAATTCCTGACGATCGTATATTAATTACAGTTGTAATAAAATTTACTGCCCCTAAAATTGAGGAAATTCCAGCTAAATGAAGAGAAAAAATAGCTAAATCAACAGAAGCTCCAGCATGAGCTGTTCCTGAAGAGAGAGGAGGATAAACTGTTCACCCAGTTCCTGCCCCATTTTCTACTATTGAGCTTGATAATAGAAGAGTCAATGAAGGAGGTAGTATTCAAAAACTTATATTATTTATTCGAGGAAAGGCTATATCAGGGGCTCCTAATATTAAAGGAACTAATCAATTTCCAAATTCCCTCCA

>QT_LB_54F7

GTCAAGTATACGTTAACGTGTTCCGTCTAAAATAGGATCTCCTCCTCCGATTGGATCAAAGAAAGATGTATTTAAGTTTCGGTCTGTTAATAACATAGTAATAGCTCCAGCTAAAACAGGAAGAGAAAGAAGTAATAAGATAGCTGTAATAACTACAGATCAAACAAATAAAGGTAGTCGATCTAAAGTAATTCCTGACGATCGTATATTAATTACAGTTGTAATAAAATTTACTGCCCCTAAAATTGAGGAAATTCCAGCTAAATGAAGAGAAAAAATAGCTAAATCAACAGAAGCTCCAGCATGAGCTGTTCCTGAAGAGAGAGGAGGATAAACTGTTCACCCAGTTCCTGCCCCATTTTCTACTATTGAGCTTGATAATAGAAGAGTCAATGAAGGAGGTAGTATTCAAAAACTTATATTATTTATTCGAGGAAAGGCTATATCAGGGGCTCCTAATATTAAAGGAACTAATCAATTTCCAAATTCCTCCA

>QT_LB_54F8

CGTCAGTATTACGATTACCTGATCCGTCTAAAATAGGATCTCCTCCTCCGATTGAGATCAAAGAAAGATGTATTTAAGTTTCGGTCTGTTAATAACATAGTAATAGCTCCAGCTAAAACAGGAAGAGAAAGAAGTAATAAGATAGCTGTAATAACTACAGATCAAACAAATAAAGGTAGTCGATCTAAAGTAATTCCTGACGATCGTATATTAATTACAGTTGTAATAAAATTTACTGCCCCTAAAATTGAGGAAATTCCAGCTAAATGAAGAGAAAAAATAGCTAAATCAACAGAAGCTCCAGCATGAGCTGTTCCTGAAGAGAGAGGAGGATAAACTGTTCACCCAGTTCCTGCCCCATTTTCTACTATTGAGCTTGATAATAGAAGAGTCAATGAAGGAGGTAGTATTCAAAAACTTATATTATTTATTCGAGGAAAGGCTATATCAGGGGCTCCTAATATTAAAGGAACTAATCAATTTCCAAATTCCTCCA

>QT_LB_55M1

CCTGCTACGTACATCACAATTTACCGTATGCATACCCAATAGGGTCTCCCCCTCCAATTGGATCAAAAAAAGATGTATTTAAATTTCGGTCTGTTAATAATATAGTAATAGCTCCGGCTAATACGGGTAGAGAAAGAAGTAATAAAATAGCTGTAATTACTACTGATCACACAAATAAAGGTAGTCGATCAAGAGTAATACCAGCTGATCGTATATTAATTACAGTTGTAATAAAATTTACTGCTCCTAAAATAGATGAGATTCCCGCTAAATGTAAAGAAAAAATTGCTAAATCAACTGAAGCCCCAGCATGAGCTGTTCCAGAAGAAAGGGGAGGATAAACCGTTCACCCTGTTCCAGCTCCGTTTTCTACTATAGAACTAGAAAGCAGCAGTGTTAAAGAGGGGGGTAATATTCAAAAACTTATATTATTTATTCGAGGAAAAGCTATATCAGGGGCTCCTAGTATTAAGGGAACTAATCAATTTCCAAATCCTCCCACA

>QT_LB_56F1

CGTGCAGCACAAAAAATCAATTTATCGTATCCTACCAAATAGGGTCTCCTCCTCCCCTTGGATCAAAAAAAGAAGTATTTAAATTTCGGTGTGTTAATAATATAGTAATAGCTCCGGCGAAAACGGGTAGAGAAAGAAGTAATAAAATAGCTGTTATTACTACTGATCACACAAATAAAGGTAGTCGATCAAGAAGAATACCACCTGATCGTATATTAATTACAGKTGTGATAAAATTTACTGGTCCCCAAAAAGATGAAAATCCCGCCTAATGTAAAGAAAAAATTGCTTATCAACTGAAGCCCCAGCATGAACTGTTCCCGAAAAAAAGAGGAGGAAAACCGTTCACCCCGTTCCCGCTCCCTTTTCTCTATAGAACTAGAAAGCAGAGTGTTAAGGAGGGGGGAGAATCCAAACTTATATTATTTTTTGAGGAAAGCTATATCAGGGCTCCCAGTATTAGGGAACTATTCAATTTCCATCCCCCAAAA

>QT_TL_59F1

GTCAGGTATCCGATTTCCTGATGCGTCTAAAATAGGATCTCCTCCTCCGCATTGAGATCAAAGAAAGATGTATTTCAGTTTCGGTCTGTTAATAACATAGTAATAGCTCCAGCTAAAACAGGAAGAGAAAGAAGTAATAAGATAGCTGTAATAACTACAGATCAAACAAATAAGGGTAGTCGATCTAAAGTAATTCCTGACGATCGTATATTAATTACAGTTGTAATAAAATTTACTGCCCCTAAAATTGAGGAAATTCCAGCTAAATGAAGAGAAAAAATAGCTTAATCAACAGAAGCTCCMGCATGAGCTGTTCCTGAAGAGAGAGGAGGATAAACTGTTCACCCAGTTCCTGCCCCATTTTCTACTATTGAGCTTGATAATAGAAGAGTCAATGAAGGAGGTAGTATTCAAAAACTTATATTATTTATTCGAGGAAAGGCTATATCAGGGGCTCCTAATATTAAAGGAACTAATCAATTTCCAAATTCCCCCCA

>QT_TL_59F2

CGTCAGTATCACATCAGCTGTTCCGTACAAAATAGGGATCTCCCCCTCCGATTGGATCAAAGAAAGATGTATTTAAGTTTCGGTCTGTTAATAAYATAGTAATAGCTCCAGCTAAAACAGGAAGAGAAAGAAGTAATAAGATAGCTGTAATAACTACAGATCAAACAAATAAGGGTAGTCGATCTAAAGTAATCCCTGACGATCGCATATTAATCACAGTTGTAATAAAATTTACTGCCCCTAAAATTGAGGAAATTCCAGCTAAATGAAGAGAAAAAATAGCTAAATCAACAGAAGCTCCAGCATGAGCTGTTCCTGAAGAGAGAGGAGGATAAACTGTTCACCCAGTTCCTGCCCCATTTTCTACTATTGAGCTTGATAATAGAAGAGTCAATGAAGGAGGTAGTATTCAAAAACTTATATTATTCATTCGAGGAAAAGCTATATCAGGGGCTCCTAATATTAAAGGAACTAATCAATTTCCAAATTCCCCCCA

>QT_TL_59F3

GTCAGTATCACGATTCCGTGATCCGTCTAAAATAGGATCTCCTCCTCCGATTGGATCAAAGAAAGATGTATTTAAGTTTCGGTCTGTTAATAACATAGTAATAGCTCCAGCTAAAACAGGAAGAGAAAGAAGTAATAAGATAGCTGTAATAACTACAGATCAAACAAATAAGGGTAGTCGATCTAAAGTAATTCCTGACGATCGTATATTAATTACAGTTGTAATAAAATTTACTGCCCCTAAAATTGAGGAAATTCCAGCTAAATGAAGAGAAAAAATAGCTAAATCAACAGAAGCTCCAGCATGAGCTGTTCCTGAAGAGAGAGGAGGATAAACTGTTCACCCAGTTCCTGCCCCATTTTCTACTATTGAGCTTGATAATAGAAGAGTCAATGAAGGAGGTAGTATTCAAAAACTTATATTATTTATTCGAGGAAAGGCTATATCAGGGGCTCCTAATATTAAAGGAACTAATCAAATTCCAAAATTCCTCCA

>QT_TL_59F4

GTTCAAGTATCACATAAGTGTTGGTACAAAATAGGGTCTCCCCCTCCGATTGGATCAAAGAAAGATGTATTTAAGTTTCGGTCTGTTAATAATATAGTAATAGCTCCAGCTAAAACAGGAAGAGAAAGAAGTAATAAGATAGCTGTAATAACTACAGATCAAACAAATAAGGGTAGTCGATCTAAAGTAATCCCTGACGATCGCATATTAATCACAGTTGTAATAAAATTTACTGCCCCTAAAATTGAGGAAATTCCAGCTAAATGAAGAGAAAAAATAGCTAAATCAACAGAAGCTCCMGCATGAGCTGTTCCTGAAGAGAGAGGAGGATAAACTGTTCACCCAGTTCCTGCCCCATTTTCTACTATTGAGCTTGATAATAGAAGAGTCAATGAAGGAGGTAGTATTCAAAAACTTATATTATTCATTCGAGGGAAAGCTATATCAGGGGCTCCTAATATTAAAGGAACTAATCAATTTCCAAATTCCTCCA

>QT_TT_61F1

GTTCAGGTATTCAATTAACCAGTTGCGTCCCAATAGGGTCTCCCCCTCCGATTGGATCAAAGAAAGATGTATTTAAGTTTCGGTCTGTTAATAATATAGTAATAGCTCCAGCTAAAACAGGAAGAGAAAGAAGTAATAAGATAGCTGTAATAACTACAGATCAAACAAATAAGGGTAGTCGATCTAAAGTAATCCCTGACGATCGCATATTAATCACAGTTGTAATAAAATTTACTGCCCCTAAAATTGAGGAAATTCCAGCTAAATGAAGAGAAAAAATAGCTAAATCAACAGAAGCTCCAGCATGAGCTGTTCCTGAAGAGAGAGGAGGATAAACTGTTCACCCAGTTCCTGCCCCATTTTCTACTATTGAGCTTGATAATAGAAGAGTCAATGAAGGAGGTAGTATTCAAAAACTTATATTATTCATTCGAGGGAAAGCTATATCAGGGGCTCCTAATATTAAAGGAACTAATCAATTTCCAAATTCCTCCA

>QT_TT_61F2

TTCAGCTATCAAATATCTAGTTGGTCTAAAATAGGGTCTCCCCCTCCGATTGGATCAAAGAAAGATGTATTTAAGTTTCGGTCTGTTAATAATATAGTAATAGCTCCAGCTAAAACAGGAAGAGAAAGAAGTAATAAGATAGCTGTAATAACTACAGATCAAACAAATAAGGGTAGTCGATCTAAAGTAATCCCTGACGATCGCATATTAATCACAGTTGTAATAAAATTTACTGCCCCTAAAATTGAGGAAATTCCAGCTAAATGAAGAGAAAAAATAGCTAAATCAACAGAAGCTCCAGCATGAGCTGTTCCTGAAGAGAGAGGAGGATAAACTGTTCACCCAGTTCCTGCCCCATTTTCTACTATTGAGCTTGATAATAGAAGAGTCAATGAAGGAGGTAGTATTCAAAAACTTATATTATTCATTCGAGGGAAAGCTATATCAGGGGCTCCTAATATTAAAGGAACTAATCAATTTCCAAAATTCCTCCCA

>QT_TT_61F3

GTCAGTATCACAATTGTAGTTCGTATAAAATAGGGTCTCCCCCTCCGATTGGATCAAAGAAAGATGTATTTAAGTTTCGGTCTGTTAATAATATAGTAATAGCTCCAGCTAAAACAGGAAGAGAAAGAAGTAATAAGATAGCTGTAATAACTACAGATCAAACAAATAAGGGTAGTCGATCTAAAGTAATCCCTGACGATCGCATATTAATCACAGTTGTAATAAAATTTACTGCCCCTAAAATTGAGGAAATTCCAGCTAAATGAAGAGAAAAAATAGCTAAATCAACAGAAGCTCCAGCATGAGCTGTTCCTGAAGAGAGAGGAGGATAAACTGTTCACCCAGTTCCTGCCCCATTTTCTACTATTGAGCTTGATAATAGAAGAGTCAATGAAGGAGGTAGTATTCAAAAACTTATATTATTCATTCGAGGGAAAGCTATATCAGGGGCTCCTAATATTAAAGGAACTAATCAATTTCCAAATTCCTCCA

>QT_TT_61F4

GGTCAGTTCCAATAAGTAGTTCCGTACAAAATAGGGTCTCCCCCTCCGATTGGATCAAAGAAAGATGTATTTAAGTTTCGGTCTGTTAATAATATAGTAATAGCTCCAGCTAAAACAGGAAGAGAAAGAAGTAATAAGATAGCTGTAATAACTACAGATCAAACAAATAAGGGTAGTCGATCTAAAGTAATCCCTGACGATCGCATATTAATCACAGTTGTAATAAAATTTACTGCCCCTAAAATTGAGGAAATTCCAGCTAAATGAAGAGAAAAAATAGCTAAATCAACAGAAGCTCCAGCATGAGCTGTTCCTGAAGAGAGAGGAGGATAAACTGTTCACCCAGTTCCTGCCCCATTTTCTACTATTGAGCTTGATAATAGAAGAGTCAATGAAGGAGGTAGTATTCAAAAACTTATATTATTCATTCGAGGGAAAGCTATATCAGGGGCTCCTAATATTAAAGGAACTAATCAATTTCCAAAATCCCTCCAA

>QT_TT_62M1

CGGTCAGTATCACGATAGCTGATCCGTACAAATAGGGATCTCCCCCTCCGATTGGATCAAAGAAAGATGTATTTAAGTTTCGGTCTGTTAATAACATAGTAATAGCTCCAGCTAAAACAGGAAGAGAAAGAAGTAATAAGATAGCTGTAATAACTACAGATCAAACAAATAAGGGTAGTCGATCTAAAGTAATTCCTGACGATCGCATATTAATYACAGTTGTAATAAAATTTACTGCCCCTAAAATTGAGGAAATTCCAGCTAAATGAAGAGAAAAAATAGCTAAATCAACAGAAGCTCCAGCATGAGCTGTTCCTGAAGAGAGAGGAGGATAAACTGTTCACCCAGTTCCTGCCCCATTTTCTACTATTGAGCTTGATAATAGAAGAGTCAATGAAGGAGGTAGTATTCAAAAACTTATATTATTCATTCGAGGGAAAGCTATATCAGGGGCTCCTAATATTAAAGGAACTAATCAATTTCCAAATTCCCTCCCA

>QT_TT_62M2

GGTCAGTATCCGATTAACGTGATCCGTCTAAAATAGGGTCTCCTCCTCCGATTGGATCAAAGAAAGATGTATTTAAGTTTCGGTCTGTTAATAACATAGTAATAGCTCCAGCTAAAACAGGAAGAGAAAGAAGTAATAAGATAGCTGTAATAACTACAGATCAAACAAATAAGGGTAGTCGATCTAAAGTAATTCCTGACGATCGTATATTAATTACAGTTGTAATAAAATTTACTGCCCCTAAAATTGAGGAAATTCCAGCTAAATGAAGAGAAAAAATAGCTAAATCAACAGAAGCTCCAGCATGAGCTGTTCCTGAAGAGAGAGGAGGATAAACTGTTCACCCAGTTCCTGCCCCATTTTCTACTATTGAGCTTGATAATAGAAGAGTCAATGAAGGAGGTAGTATTCAAAAACTTATATTATTTATTCGAGGAAAGGCTATATCAGGGGCTCCTAATATTAAAGGAACTAATCAATTTCCAAATTCCTCCCA

>QT_TT_62M3

CGTTCAGTATCCGATAGTGTTCCGTACAAATAGGGATCTCCCCCTCCGATTGGATCAAAGAAAGATGTATTTAAGTTTCGGTCTGTTAATAATATAGTAATAGCTCCAGCTAAAACAGGAAGAGAAAGAAGTAATAAGATAGCTGTAATAACTACAGATCAAACAAATAAGGGTAGTCGATCTAAAGTAATCCCTGACGATCGCATATTAATCACAGTTGTAATAAAATTTACTGCCCCTAAAATTGAGGAAATTCCAGCTAAATGAAGAGAAAAAATAGCTAAATCAACAGAAGCTCCAGCATGAGCTGTTCCTGAAGAGAGAGGAGGATAAACTGTTCACCCAGTTCCTGCCCCATTTTCTACTATTGAGCTTGATAATAGAAGAGTCAATGAAGGAGGTAGTATTCAAAAACTTATATTATTCATTCGAGGAAAAGCTATATCAGGGGCTCCTAATATTAAAGGAACTAATCAATTTCCAAATTCCCCCCA

>QT_TT_63F1

AAAAATACTCTCAATTTATCGTATGCATATCAAATAGGGTCTCCACCTCCCATAGGATCAAAGAAGGAAGTATTTAAATTTCGGTCAGTTAATAATATAGTAATTGCTCCAGCTAAAACTGGTAATGAAAGAAGTAAGAGAATTGCTGTAATAACAACAGATCAAACAAATAATGGTAATCGGTCTAATGTAATACCTGAAGATCGCATATTAATTACTGTGGTAATAAAATTTACAGCTCCTAGAATAGATGAAATTCCAGCTAAATGTAAAGAAAAAATAGCTAAATCAACTGATGCACCAGCATGAGCTGTTCCAGAAGATAGAGGTGGATAAACTGTTCAACCTGTTCCTGCTCCATTTTCTACTATTGAACTAGAAAGTAATAGGGTTAATGAAGGGGGAAGTATTCAAAAGCTTATATTATTTATTCGAGGAAATGCTATATCTGGTGCTCCTAATATTAATGGAACTAATCAATTTCCAAATTCCCTCCA

>QT_TT_64F1

CGAATGCCTACCGAATAGGGTCTCCCCCTCCCATTGCGATCAAAAAAAGATGTATTTAAATTTAGGTCTGTTAATAATATAGTAATAGCTCCGGCTAATACGGGTAGAGAAAGAAGTAATAAAATAGCTGTAATTACTACTGATCACACAAATAAAGGTAGTCGATCAAGAGTAATACCAGCTGATCGTATATTAATTACAGTTGTAATAAAATTTACTGCTCCTAAAATAGATGAGATTCCCGCTAAATGTAAAGAAAAAATTGCTAAATCAACTGAAGCCCCAGCATGAGCTGTTCCAGAAGAAAGGGGAGGATAAACCGTTCACCCTGTTCCAGCTCCGTTTTCTACTATAGAACTAGAAAGCAGCAGTGTTAAAGAGGGGGGTAATATTCAAAAACTTATATTATTTATTCGAGGAAAAGCTATATCAGGGGCTCCTAGTATTAAGGGAACTAATCAATTTCCAAAATCCTCCCA

>QT_TT_64F2

CGTCGAAATATCAATTTCCATATGCTACCCAATAGGGTCTCCTCCTCCTATTGGGTCAAAAAATGAAGTATTAAGATTTCGATCTGTTAATAATATAGTAATAGCTCCTGCTAAAACAGGGAGTGATAAAAGTAATAATACAGCAGTAATTACAACTGATCATACAAATAGAGGAAGTCGGTCTAAAGTAATTCCTGAAGATCGTATATTAATTACAGTAGTAATGAAATTTACTGCTCCTAAAATTGAAGAAATCCCAGCTAAATGAAGAGAAAAAATAGTTAGATCTACTGAGGCCCCTGCATGAGCAGTTCCTGAGGATAGTGGTGGGTAAACGGTTCATCCGGTACCAGCCCCATTTTCTACTATACTACTAGAAAGTAATAAAGTTAATGAAGGAGGTAATATTCAAAAACTTATATTATTTATTCGAGGAAAAGCTATATCAGGAGCTCCTAGTATTAAAGGAACTAATCAATTTCCAAAATCCTCCAA

>HT_GP_67M1

GTGGGGTCAGATCAGATAAGTGTTGGTATAAAATAGGATCTCCTCCTCCGATTGGATCAAAGAAAGATGTATTTAAGTTTCGGTCTGTTAATAACATAGTAATAGCTCCAGCTAAAACAGGAAGAGAAAGAAGTAATAAGATAGCTGTAATAACTACAGATCAAACAAATAAAGGTAGTCGATCTAAAGTAATTCCTGACGATCGTATATTAATTACAGTTGTAATAAAATTTACTGCCCCTAAAATTGAGGAAATTCCAGCTAAATGAAGAGAAAAAATAGCTAAATCAACAGAAGCTCCAGCATGAGCTGTTCCTGAAGAGAGAGGAGGATAAACTGTTCACCCAGTTCCTGCCCCATTTTCTACTATTGAGCTTGATAATAGAAGAGTCAATGAAGGAGGTAGTATTCAAAAACTTATATTATTTATTCGAGGAAAGGCTATATCAGGGGCTCCTAATATTAAAGGAACTAATCAATTTCCAAATTCCTCCAA

>HT_GP_67M2

GGGGGGGTTCAGATCAGATAAGTGTTGGTATAAATAGGATCTCCTCCTCCGATTGGATCAAAGAAAGATGTATTTAAGTTTCGGTCTGTTAATAACATAGTAATAGCTCCAGCTAAAACAGGAAGAGAAAGAAGTAATAAGATAGCTGTAATAACTACAGATCAAACAAATAAAGGTAGTCGGTCTAAAGTAATTCCTGACGATCGTATATTAATTACAGTTGTAATAAAATTTACTGCCCCTAAAATTGAGGAAATTCCAGCTAAATGAAGAGAAAAAATAGCTAAATCAACAGAAGCTCCAGCATGAGCTGTTCCTGAAGAGAGAGGAGGATAAACTGTTCACCCAGTTCCTGCCCCATTTTCTACTATTGAGCTTGATAATAGAAGAGTCAATGAAGGAGGTAGTATTCAAAAACTTATATTATTTATTCGAGGAAAGGCTATATCAGGGGCTCCTAATATTAAAGGAACTAAYCAATTTCCAAATTCCCCCCA

>HT_GP_67M3

GTGGGTCAAGATCAGATAAGTGTTGGTATAAAATAGGATCTCCTCCTCCGATTGGATCAAAGAAAGATGTATTTAAGTTTCGGTCTGTTAATAACATAGTAATAGCTCCAGCTAAAACAGGAAGAGAAAGAAGTAATAAGATAGCTGTAATAACTACAGATCAAACAAATAAAGGTAGTCGATCTAAAGTAATTCCTGACGATCGTATATTAATTACAGTTGTAATAAAATTTACTGCCCCTAAAATTGAGGAAATTCCAGCTAAATGAAGAGAAAAAATAGCTAAATCAACAGAAGCTCCAGCATGAGCTGTTCCTGAAGAGAGAGGAGGATAAACTGTTCACCCAGTTCCTGCCCCATTTTCTACTATTGAGCTTGATAATAGAAGAGTCAATGAAGGAGGTAGTATTCAAAAACTTATATTATTTATTCGAGGAAAGGCTATATCAGGGGCTCCTAATATTAAAGGAACTAATCAATTTCCAAATCCTCCA

>HT_GP_67M4

CTGGGGTCAGTATCAGATTAGTGTTGGTATAAAATAGGATCTCCTCCTCCGATTGGATCAAAGAAAGATGTATTTAAGTTTCGGTCTGTTAATAACATAGTAATAGCTCCAGCTAAAACAGGAAGAGAAAGAAGTAATAAGATAGCTGTAATAACTACAGATCAAACAAATAAAGGTAGTCGATCTAAAGTAATTCCTGACGATCGTATATTAATTACAGTTGTAATAAAATTTACTGCCCCTAAAATTGAGGAAATTCCAGCTAAATGAAGAGAAAAAATAGCTAAATCAACAGAAGCTCCAGCATGAGCTGTTCCTGAAGAGAGAGGAGGATAAACTGTTCACCCAGTTCCTGCCCCATTTTCTACTATTGAGCTTGATAATAGAAGAGTCAATGAAGGAGGTAGTATTCAAAAACTTATATTATTTATTCGAGGAAAGGCTATATCAGGGGCTCCTAATATTAAAGGAACTAATCAATTTCCAAATTCCTCCCAA

>HT_GP_67M5

GTGGGGTTAAAGATCAGATAAGTGTTGGTATAAATAGGATCTCCTCCTCCGATTGGATCAAAGAAAGATGTATTTAAGTTTCGGTCTGTTAATAACATAGTAATAGCTCCAGCTAAAACAGGAAGAGAAAGAAGTAATAAGATAGCTGTAATAACTACAGATCAAACAAATAAAGGTAGTCGATCTAAAGTAATTCCTGACGATCGTATATTAATTACAGTTGTAATAAAATTTACTGCCCCTAAAATTGAGGAAATTCCAGCTAAATGAAGAGAAAAAATAGCTAAATCAACAGAAGCTCCAGCATGAGCTGTTCCTGAAGAGAGAGGAGGATAAACTGTTCACCCAGTTCCTGCCCCATTTTCTACTATTGAGCTTGATAATAGAAGAGTCAATGAAGGAGGTAGTATTCAAAAACTTATATTATTTATTCGAGGAAAGGCTATATCAGGGGCTCCTAATATTAAAGGAACTAATCAATTTCCAAATCCTCCA

>HT_GP_67M6

GTGGGTCAGATCAGATAGTGTTGGTATAAAATAGGATCTCCTCCTCCGATTGGATCAAAGAAAGATGTATTTAAGTTTCGGTCTGTTAATAACATAGTAATAGCTCCAGCTAAAACAGGAAGAGAAAGAAGTAATAAGATAGCTGTAATAACTACAGATCAAACAAATAAAGGTAGTCGATCTAAAGTAATTCCTGACGATCGTATATTAATTACAGTTGTAATAAAATTTACTGCCCCTAAAATTGAGGAAATTCCAGCTAAATGAAGAGAAAAAATAGCTAAATCAACAGAAGCTCCAGCATGAGCTGTTCCTGAAGAGAGAGGAGGATAAACTGTTCACCCAGTTCCTGCCCCATTTTCTACTATTGAGCTTGATAATAGAAGAGTCAATGAAGGAGGTAGTATTCAAAAACTTATATTATTTATTCGAGGAAAGGCTATATCAGGGGCTCCTAATATTAAAGGAACTAATCAATTTCCAAAATCCTCCCA

>HT_GP_67M7

CTTGCGTCAAGTATCAGTTTCGTGTTCCGTATAAAATAGGATCTCCTCCTCCGATTGGATCAAAGAAAGATGTATTTAAGTTTCGGTCTGTTAATAACATAGTAATAGCTCCAGCTAAAACAGGAAGAGAAAGAAGTAATAAGATAGCTGTAATAACTACAGATCAAACAAATAAAGGTAGTCGATCTAAAGTAATTCCTGACGATCGTATATTAATTACAGTTGTAATAAAATTTACTGCCCCTAAAATTGAGGAAATTCCAGCTAAATGAAGAGAAAAAATAGCTAAATCAACAGAAGCTCCAGCATGAGCTGTTCCTGAAGAGAGAGGAGGATAAACTGTTCACCCAGTTCCTGCCCCATTTTCTACTATTGAGCTTGATAATAGAAGAGTCAATGAAGGAGGTAGTATTCAAAAACTTATATTATTTATTCGAGGAAAGGCTATATCAGGGGCTCCTAATATTAAAGGAACTAATCAATTTCCAAATCCTCCA

>HT_GP_67M8

TTGGGGGTCAGATCAGATAAGTGTTGGTATAAAATAGGATCTCCTCCTCCGATTGGATCAAAGAAAGATGTATTTAAGTTTCGGTCTGTTAATAACATAGTAATAGCTCCAGCTAAAACAGGAAGAGAAAGAAGTAATAAGATAGCTGTAATAACTACAGATCAAACAAATAAAGGTAGTCGATCTAAAGTAATTCCTGACGATCGTATATTAATTACAGTTGTAATAAAATTTACTGCCCCTAAAATTGAGGAAATTCCAGCTAAATGAAGAGAAAAAATAGCTAAATCAACAGAAGCTCCAGCATGAGCTGTTCCTGAAGAGAGAGGAGGATAAACTGTTCACCCAGTTCCTGCCCCATTTTCTACTATTGAGCTTGATAATAGAAGAGTCAATGAAGGAGGTAGTATTCAAAAACTTATATTATTTATTCGAGGAAAGGCTATATCAGGGGCTCCTAATATTAAAGGAACTAATCAATTTCCAAATCCTCCA

>HT_GP_67M9

TGGGGGGTCAGATCAGATAAGTGTTGGTATAAAATAGGATCTCCTCCTCCGATTGGATCAAAGAAAGATGTATTTAAGTTTCGGTCTGTTAATAACATAGTAATAGCTCCAGCTAAAACAGGAAGAGAAAGAAGTAATAAGATAGCTGTAATAACTACAGATCAAACAAATAAAGGTAGTCGGTCTAAAGTAATTCCTGACGATCGTATATTAATTACAGTTGTAATAAAATTTACTGCCCCTAAAATTGAGGAAATTCCAGCTAAATGAAGAGAAAAAATAGCTAAATCAACAGAAGCTCCAGCATGAGCTGTTCCTGAAGAGAGAGGAGGATAAACTGTTCACCCAGTTCCTGCCCCATTTTCTACTATTGAGCTTGATAATAGAAGAGTCAATGAAGGAGGTAGTATTCAAAAACTTATATTATTTATTCGAGGAAAGGCTATATCAGGGGCTCCTAATATTAAAGGAACTAATCAATTTCCAAATTCCTCCA

>HT_GP_68F1

TGTTCCGTATAATATAGGATCTCCTCCTCCGATTGGATCAAAGAAAGATGTATTTAAGTTTCGGTCTGTTAATAACATAGTAATAGCTCCGCTAAAACAGGAAGAGAAAGAAGTAATAAGATAGCTGTAATAACTACAGATCAAACAAATAAAGGTAGTCGATCTAAAGTAATTCCTGACGATCGTATATTAATTACAGTTGTAATAAAATTTACTGCCCCTAAAATTGAGGAAATTCCAGCTAAATGAAGAGAAAAAATAGCTAAATCAACAGAAGCTCCAGCATGAGCTGTTCCTGAAGAGAGAGGAGGATAAACTGTTCACCCAGTTCCTGCCCCATTTTCTACTATTGAGCTTGATAATAGAAGAGTCAATGAAGGAGGTAGTATTCAAAAACTTATATTATTTATTCGAGGAAAGGCTATATCAGGGGCTCCTAATATTAAAGGAACTAATCATTTACAAATTCCCTCCTA

>HT_GP_68F2

TTTACCTGTTCCGTATAAAATAGGATCTCCTCCTCCGATTGGATCAAAGAAAGATGTATTTAAGTTTCGGTCTGTTAATAACATAGTAATAGCTCCAGCTAAAACAGGAAGAGAAAGAAGTAATAAGATAGCTGTAATAACTACAGATCAAACAAATAAAGGTAGTCGATCTAAAGTAATTCCTGACGATCGTATATTAATTACAGTTGTAATAAAATTTACTGCCCCTAAAATTGAGGAAATTCCAGCTAAATGAAGAGAAAAAATAGCTAAATCAACAGAAGCTCCAGCATGAGCTGTTCCTGAAGAGAGAGGAGGATAAACTGTTCACCCAGTTCCTGCCCCATTTTCTACTATTGAGCTTGATAATAGAAGAGTCAATGAAGGAGGTAGTATTCAAAAACTTATATTATTTATTCGAGGAAAGGCTATATCAGGGGCTCCTAATATTAAAGGAACTAATCAATTTCAAACCCCCTCCTA

>HT_GP_68F3

GTGTTCCGTCCAAAATAGGATCTCCTCCTCCGATTGGATCAAAGAAAGATGTATTTAAGTTTCGGTCTGTTAATAACATAGTAATAGCTCCAGCTAAAACAGGAAGAGAAAGAAGTAATAAGATAGCTGTAATAACTACAGATCAAACAAATAAAGGTAGTCGATCTAAAGTAATTCCTGACGATCGTATATTAATTACAGTTGTAATAAAATTTACTGCCCCTAAAATTGAGGAAATTCCAGCTAAATGAAGAGAAAAAATAGCTAAATCAACAGAAGCTCCAGCATGAGCTGTTCCTGAAGAGAGAGGAGGATAAACTGTTCACCCAGTTCCTGCCCCATTTTCTACTATTGAGCTTGATAATAGAAGAGTCAATGAAGGAGGTAGTATTCAAAAACTTATATTATTTATTCGAGGAAAGGCTATATCAGGGGCTCCTAATATTAAAGGAACTAATCAATTTCAAAATTCCCCCCA

>HT_HK_70F1

TGATCCGTCTAAAATAGGATCTCCTCCTCCGATTGAGATCAAAGAAAGATGTATTTAAGTTTCGGTCTGTTAATAACATAGTAATAGCTCCAGCTAAAACAGGAAGAGAAAGAAGTAATAAGATAGCTGTAATAACTACAGATCAAACAAATAAAGGTAGTCGATCTAAAGTAATTCCTGACGATCGTATATTAATTACAGTTGTAATAAAATTTACTGCCCCTAAAATTGAGGAAATTCCAGCTAAATGAAGAGAAAAAATAGCTAAATCAACAGAAGCTCCAGCATGAGCTGTTCCTGAAGAGAGAGGAGGATAAACTGTTCACCCAGTTCCTGCCCCATTTTCTACTATTGAGCTTGATAATAGAAGAGTCAATGAAGGAGGTAGTATTCAAAAACTTATATTATTTATTCGAGGAAAGGCTATATCAGGGGCTCCTAATATTAAAGGAACTAATCAATTTCAAAATTCCCCCCCA

>HT_HK_70F2

GATTTACCTGTTCCGTATAAAATAGGATCTCCTCCTCCGATTGGATCAAAGAAAGATGTATTTAAGTTTCGGTCTGTTAATAACATAGTAATAGCTCCAGCTAAAACAGGAAGAGAAAGAAGTAATAAGATAGCTGTAATAACTACAGATCAAACAAATAAAGGTAGTCGATCTAAAGTAATTCCTGACGATCGTATATTAATTACAGTTGTAATAAAATTTACTGCCCCTAAAATTGAGGAAATTCCAGCTAAATGAAGAGAAAAAATAGCTAAATCAACAGAAGCTCCAGCATGAGCTGTTCCTGAAGAGAGAGGAGGATAAACTGTTCACCCAGTTCCTGCCCCATTTTCTACTATTGAGCTTGATAATAGAAGAGTCAATGAAGGAGGTAGTATTCAAAAACTTATATTATTTATTCGAGGAAAGGCTATATCAGGGGCTCCTAATATTAAAGGAACTAATCAATTTCAAAATTCCCCCCA

>HT_HK_70F3

ATAAGTGTTCCGTACAAAATAGGGTCTCCCCCTCCGATTGGATCAAAGAAAGATGTATTTAAGTTTCGGTCTGTTAATAATATAGTAATAGCTCCAGCTAAAACAGGAAGAGAAAGAAGTAATAAGATAGCTGTAATAACTACAGATCAAACAAATAAGGGTAGTCGATCTAAAGTAATCCCTGACGATCGCATATTAATCACAGTTGTAATAAAATTTACTGCCCCTAAAATTGAGGAAATTCCAGCTAAATGAAGAGAAAAAATAGCTAAATCAACAGAAGCTCCAGCATGAGCTGTTCCTGAAGAGAGAGGAGGATAAACTGTTCACCCAGTTCCTGCCCCATTTTCTACTATTGAGCTTGATAATAGAAGAGTCAATGAAGGAGGTAGTATTCAAAAACTTATATTATTCATTCGAGGGAAAGCTATATCAGGGGCTCCTAATATTAAAGGAACTAATCAATTTCAAATTCCCCCCAA

>HT_HK_70F4

TGATCCGTCTAAAATAGGATCTCCTCCTCCGATTGGATCAAAGAAAGATGTATTTAAGTTTCGGTCTGTTAATAACATAGTAATAGCTCCAGCTAAAACAGGAAGAGAAAGAAGTAATAAGATAGCTGTAATAACTACAGATCAAACAAATAAAGGTAGTCGATCTAAAGTAATTCCTGACGATCGTATATTAATTACAGTTGTAATAAAATTTACTGCCCCTAAAATTGAGGAAATTCCAGCTAAATGAAGAGAAAAAATAGCTAAATCAACAGAAGCTCCAGCATGAGCTGTTCCTGAAGAGAGAGGAGGATAAACTGTTCACCCAGTTCCTGCCCCATTTTCTACTATTGAGCTTGATAATAGAAGAGTCAATGAAGGAGGTAGTATTCAAAAACTTATATTATTTATTCGAGGAAAGGCTATATCAGGGGCTCCTAATATTAAAGGAACTAATCAATTTCAAAATTCCCTCCCA

>HT_HK_70F5

AAAATAGGATCTCCTCCTCCGATTGGATCAAAGAAAGATGTATTTAAGTTTCGGTCTGTTAATAACATAGTAATAGCTCCAGCTAAAACAGGAAGAGAAAGAAGTAATAAGATAGCTGTAATAACTACAGATCAAACAAATAAAGGTAGTCGGTCTAAAGTAATTCCTGACGATCGTATATTAATTACAGTTGTAATAAAATTTACTGCCCCTAAAATTGAGGAAATTCCAGCTAAATGAAGAGAAAAAATAGCTAAATCAACAGAAGCTCCAGCATGAGCTGTTCCTGAAGAGAGAGGAGGATAAACTGTTCACCCAGTTCCTGCCCCATTTTCTACTATTGAGCTTGATAATAGAAGAGTCAATGAAGGAGGTAGTATTCAAAAACTTATATTATTTATTCGAGGAAAGGCTATATCAGGGGCTCCTAATATTAAAGGAACTAATCAATTTCCAAAATTCCCTCCCA

>HT_HK_70F6

AACGTGTTCCGTATAAAATAGGATCTCCTCCTCCGATTGGATCAAAGAAAGATGTATTTAAGTTTCGGTCTGTTAATAACATAGTAATAGCTCCAGCTAAAACAGGAAGAGAAAGAAGTAATAAGATAGCTGTAATAACTACAGATCAAACAAATAAAGGTAGTCGGTCTAAAGTAATTCCTGACGATCGTATATTAATTACAGTTGTAATAAAATTTACTGCCCCTAAAATTGAGGAAATTCCAGCTAAATGAAGAGAAAAAATAGCTAAATCAACAGAAGCTCCAGCATGAGCTGTTCCTGAAGAGAGAGGAGGATAAACTGTTCACCCAGTTCCTGCCCCATTTTCTACTATTGAGCTTGATAATAGAAGAGTCAATGAAGGAGGTAGTATTCAAAAACTTATATTATTTATTCGAGGAAAGGCTATATCAGGGGCTCCTAATATTAAAGGAACTAATCATTTCCAAAATTCCCCCA

>HT_HK_70F7

TCTCGAGCTACTCAATTTCCTCCAGATCCCASTAGTCAATTTCCCAATCCTCCGGATCATGTAATGATCCATTTCCATCTCGGTCTGTTAATAACATAGTAATAGCTCCAGCTAAAACAGGAAGAGAAAGAAGTAATAAGATAGCTGTAATAACTACAGATCAAACAAATAAAGGTAGTCGATCTAAAGTAATTCCTGACGATCGTATATTAATTACAGTTGTAATAAAATTTACTGCCCCTAAAATTGAGGAAATTCCAGCTAAATGAAGAGAAAAAATAGCTAAATCAACAGAAGCTCCAGCATGAGCTGTTCCTGAAGAGAGAGGAGGATAAACTGTTCACCCAGTTCCTGCCCCATTTTCTACTATTGAGCTTGATAATAGAAGAGTCAATGAAGGAGGTAGTATTCAAAAACTTATATTATTTATTCGAGGAAAGGCTATATCAGGGGCTCCTAATATTAAAGGAACTAATCAATTTCCAAATCCTCCCAA

>HT_HK_70F8

AAAATAGGATCTCCTCCTCCGATTGGATCAAAGAAAGATGTATTTAAGTTTCGGTCTGTTAATAACATAGTAATAGCTCCAGCTAAAACAGGAAGAGAAAGAAGTAATAAGATAGCTGTAATAACTACAGATCAAACAAATAAAGGTAGTCGATCTAAAGTAATTCCTGACGATCGTATATTAATTACAGTTGTAATAAAATTTACTGCCCCTAAAATTGAGGAAATTCCAGCTAAATGAAGAGAAAAAATAGCTAAATCAACAGAAGCTCCAGCATGAGCTGTTCCTGAAGAGAGAGGAGGATAAACTGTTCACCCAGTTCCTGCCCCATTTTCTACTATTGAGCTTGATAATAGAAGAGTCAATGAAGGAGGTAGTATTCAAAAACTTATATTATTTATTCGAGGAAAGGCTATATCAGGGGCTCCTAATATTAAAGGAACTAATCAATTTCAAATTCCCTCCCA

>HT_HK_70F9

AAATAGGCATCTCCTCCTCTCGCATCCAGATCAAAGAAAGATGTATTTCAGTTTCGGTCTGTTAATAACATAGTAATAGCTCCAGCTAAAACAGGAAGAGAAAGAAGTAATAAGATAGCTGTAATAACTACAGATCAAACAAATAAAGGTAGTCGGTCTAAAGTAATTCCTGACGATCGTATATTAATTACAGTTGTAATAAAATTTACTGCCCCTAAAATTGAGGAAATTCCAGCTAAATGAAGAGAAAAAATAGCTAAATCAACAGAAGCTCCAGCATGAGCTGTTCCTGAAGAGAGAGGAGGATAAACTGTTCACCCAGTTCCTGCCCCATTTTCTACTATTGAGCTTGATAATAGAAGAGTCAATGAAGGAGGTAGTATTCAAAAACTTATATTATTTATTCGAGGAAAGGCTATATCAGGGGCTCCTAATATTAAAGGAACTAATCAATTTCAAATTCCTCACA

>HT_HK_70F10

AAAATAGGATCTCCTCCTCCGATTGAGATCAAAGAAAGATGTATTTAAGTTTCGGTCTGTTAATAACATAGTAATAGCTCCAGCTAAAACAGGAAGAGAAAGAAGTAATAAGATAGCTGTAATAACTACAGATCAAACAAATAAAGGTAGTCGGTCTAAAGTAATTCCTGACGATCGTATATTAATTACAGTTGTAATAAAATTTACTGCCCCTAAAATTGAGGAAATTCCAGCTAAATGAAGAGAAAAAATAGCTAAATCAACAGAAGCTCCAGCATGAGCTGTTCCTGAAGAGAGAGGAGGATAAACTGTTCACCCAGTTCCTGCCCCATTTTCTACTATTGAGCTTGATAATAGAAGAGTCAATGAAGGAGGTAGTATTCAAAAACTTATATTATTTATTCGAGGAAAGGCTATATCAGGGGCTCCTAATATTAAAGGAACTAATCAATTTCAAAATTCCCCCCCA

>HT_HK_70F11

CGCTACGTACTACTCAATTTCCGTATGCCTACCCAATAGGGTCTCCTCCTCCCATTGCGATCAAAAAAAGATGTATTTAAATTTAGGTCTGTTAATAATATAGTAATAGCTCCGGCTAATACGGGTAGAGAAAGAAGTAATAAAATAGCTGTAATTACTACTGATCACACAAATAAAGGTAGTCGATCAAGAGTAATACCAGCTGATCGTATATTAATTACAGTTGTAATAAAATTTACTGCTCCTAAAATAGATGAGATTCCCGCTAAATGTAAAGAAAAAATTGCTAAATCAACTGAAGCCCCAGCATGAGCTGTTCCAGAAGAAAGGGGAGGATAAACCGTTCACCCTGTTCCAGCTCCGTTTTCTACTATAGAACTAGAAAGCAGCAGTGTTAAAGAGGGGGGTAATATTCAAAAACTTATATTATTTATTCGAGGAAAAGCTATATCAGGGGCTCCTAGTATTAAGGGAACTAATCAATTTCCAAAATTCCTCCCA

>HT_HK_70F12

GATTTACGTGTTCCGTATAAAATAGGATCTCCTCCTCCGATTGGATCAAAGAAAGATGTATTTAAGTTTCGGTCTGTTAATAACATAGTAATAGCTCCAGCTAAAACAGGAAGAGAAAGAAGTAATAAGATAGCTGTAATAACTACAGATCAAACAAATAAAGGTAGTCGATCTAAAGTAATTCCTGACGATCGTATATTAATTACAGTTGTAATAAAATTTACTGCCCCTAAAATTGAGGAAATTCCAGCTAAATGAAGAGAAAAAATAGCTAAATCAACAGAAGCTCCAGCATGAGCTGTTCCTGAAGAGAGAGGAGGATAAACTGTTCACCCAGTTCCTGCCCCATTTTCTACTATTGAGCTTGATAATAGAAGAGTCAATGAAGGAGGTAGTATTCAAAAACTTATATTATTTATTCGAGGAAAGGCTATATCAGGGGCTCCTAATATTAAAGGAACTAATCAACTTTCAAATTCCCCCCAA

>HT_HK_70F13

GTTCCGTATAAAATAGGGTCTCCCCCTCCGATTGGATCAAAGAAAGATGTATTTAAGTTTCGGTCTGTTAATAATATAGTAATAGCTCCAGCTAAAACAGGAAGAGAAAGAAGTAATAAGATAGCTGTAATAACTACAGATCAAACAAATAAGGGTAGTCGATCTAAAGTAATCCCTGACGATCGCATATTAATCACAGTTGTAATAAAATTTACTGCCCCTAAAATTGAGGAAATTCCAGCTAAATGAAGAGAAAAAATAGCTAAATCAACAGAAGCTCCAGCATGAGCTGTTCCTGAAGAGAGAGGAGGATAAACTGTTCACCCAGTTCCTGCCCCATTTTCTACTATTGAGCTTGATAATAGAAGAGTCAATGAAGGAGGTAGTATTCAAAAACTTATATTATTCATTCGAGGGAAAGCTATATCAGGGGCTCCTAATATTAAAGGAACTAATCAATTTCAAAATTCCCCCCA

>HT_HK_70F14

GTTTACGTGTTCCGTATAAAATAGGATCTCCTCCTCCGATTGGATCAAAGAAAGATGTATTTAAGTTTCGGTCTGTTAATAACATAGTAATAGCTCCAGCTAAAACAGGAAGAGAAAGAAGTAATAAGATAGCTGTAATAACTACAGATCAAACAAATAAAGGTAGTCGGTCTAAAGTAATTCCTGACGATCGTATATTAATTACAGTTGTAATAAAATTTACTGCCCCTAAAATTGAGGAAATTCCAGCTAAATGAAGAGAAAAAATAGCTAAATCAACAGAAGCTCCAGCATGAGCTGTTCCTGAAGAGAGAGGAGGATAAACTGTTCACCCAGTTCCTGCCCCATTTTCTACTATTGAGCTTGATAATAGAAGAGTCAATGAAGGAGGTAGTATTCAAAAACTTATATTATTTATTCGAGGAAAGGCTATATCAGGGGCTCCTAATATTAAAGGAACTAATCATTTCCAAATTCCTCCGAGCA

>HT_HK_70F15

CGTGATCCGTCTAAAATAGGATCTCCTCCTCCGATTGAGATCAAAGAAAGATGTATTTAAGTTTCGGTCTGTTAATAACATAGTAATAGCTCCAGCTAAAACAGGAAGAGAAAGAAGTAATAAGATAGCTGTAATAACTACAGATCAAACAAATAAAGGTAGTCGGTCTAAAGTAATTCCTGACGATCGTATATTAATTACAGTTGTAATAAAATTTACTGCCCCTAAAATTGAGGAAATTCCAGCTAAATGAAGAGAAAAAATAGCTAAATCAACAGAAGCTCCAGCATGAGCTGTTCCTGAAGAGAGAGGAGGATAAACTGTTCACCCAGTTCCTGCCCCATTTTCTACTATTGAGCTTGATAATAGAAGAGTCAATGAAGGAGGTAGTATTCAAAAACTTATATTATTTATTCGAGGAAAGGCTATATCAGGGGCTCCTAATATTAAAGGAACTAATCAATTTCCAAATTCCTCCA

>HT_HK_71M1

ATTTACCTGTTCCGTACAAATAGGGTCTCCTCCTCCGATTGGATCAAAGAAAGATGTATTTAAGTTTCGGTCTGTTAATAACATAGTAATAGCTCCGCTAAAACAGGAAGAGAAAGAAGTAATAAGATAGCTGTAATAACTACAGATCAACAAATAAGGGTAGTCGGTCTAAAGTAATTCCTGACGATCGTATATTAATTACAGTTGTAATAAAATTTACTGCCCCTAAAATTGAGGAAATTCCAGCTAAATGAAGAGAAAAAATAGCTAAATCAACAGAAGCTCCAGCATGAGCTGTTCCTGAAGAGAGAGGAGGATAAACTGTTCACCCAGTTCCTGCCCCATTTTCTACTATTGAGCTTGATAATAGAAGAGTCAATGAAGGAGGTAGTATTCAAAAACTTATATTATTTATTCGAGGAAAGGCTATATCAGGGGCTCCTAATATTAAAGGAACTAATCAATTTCCAAATTCCTCCCTA

>HT_HK_71M2

ATTTCGTGTTCCGTATAAAATAGGATCTCCTCCTCCGATTGGATCAAAGAAAGATGTATTTAAGTTTCGGTCTGTTAATAACATAGTAATAGCTCCAGCTAAAACAGGAAGAGAAAGAAGTAATAAGATAGCTGTAATAACTACAGATCAAACAAATAARGGTAGTCGATCTAAAGTAATTCCTGACGATCGTATATTAATTACAGTTGTAATAAAATTTACTGCCCCTAAAATTGAGGAAATTCCAGCTAAATGAAGAGAAAAAATAGCTAAATCAACAGAAGCTCCAGCATGAGCTGTTCCTGAAGAGAGAGGAGGATAAACTGTTCACCCAGTTCCTGCCCCATTTTCTACTATTGAGCTTGATAATAGAAGAGTCAATGAAGGAGGTAGTATTCAAAAACTTATATTATTTATTCGAGGAAAGGCTATATCAGGGGCTCCTAATATTAAAGGAACTAATCAATTTACAAATTCCCCCCA

>HT_HK_71M3

TGATCCGTATAAAATAGGATCTCCTCCTCCGATTGGATCAAAGAAAGATGTATTTAAGTTTCGGTCTGTTAATAACATAGTAATAGCTCCAGCTAAAACAGGAAGAGAAAGAAGTAATAAGATAGCTGTAATAACTACAGATCAAACAAATAAAGGTAGTCGATCTAAAGTAATTCCTGACGATCGTATATTAATTACAGTTGTAATAAAATTTACTGCCCCTAAAATTGAGGAAATTCCAGCTAAATGAAGAGAAAAAATAGCTAAATCAACAGAAGCTCCAGCATGAGCTGTTCCTGAAGAGAGAGGAGGATAAACTGTTCACCCAGTTCCTGCCCCATTTTCTACTATTGAGCTTGATAATAGAAGAGTCAATGAAGGAGGTAGTATTCAAAAACTTATATTATTTATTCGAGGAAAGGCTATATCAGGGGCTCCTAATATTAAAGGAACTAATCAATTTCAAATTCCTCCA

>HT_HK_71M4

AAAATAGGATCTCCTCCTCCGATTGAGATCAAAGAAAGATGTATTTAAGTTTCGGTCTGTTAATAACATAGTAATAGCTCCAGCTAAAACAGGAAGAGAAAGAAGTAATAAGATAGCTGTAATAACTACAGATCAAACAAATAAAGGTAGTCGATCTAAAGTAATTCCTGACGATCGTATATTAATTACAGTTGTAATAAAATTTACTGCCCCTAAAATTGAGGAAATTCCAGCTAAATGAAGAGAAAAAATAGCTAAATCAACAGAAGCTCCAGCATGAGCTGTTCCTGAAGAGAGAGGAGGATAAACTGTTCACCCAGTTCCTGCCCCATTTTCTACTATTGAGCTTGATAATAGAAGAGTCAATGAAGGAGGTAGTATTCAAAAACTTATATTATTTATTCGAGGAAAGGCTATATCAGGGGCTCCTAATATTAAAGGAACTAATCAATTTCCAAATTCCCCCCAA

>HT_HK_71M5

TGTTCCGTCTAAATAGGATCTCCTCCTCCGATTGGATCAAAGAAAGATGTATTTAAGTTTCGGTCTGTTAATAACATAGTAATAGCTCCAGCTAAAACAGGAAGAGAAAGAAGTAATAAGATAGCTGTAATAACTACAGATCAAACAAATAARGGTAGTCGGTCTAAAGTAATTCCTGACGATCGTATATTAATTACAGTTGTAATAAAATTTACTGCCCCTAAAATTGAGGAAATTCCAGCTAAATGAAGAGAAAAAATAGCTAAATCAACAGAAGCTCCAGCATGAGCTGTTCCTGAAGAGAGAGGAGGATAAACTGTTCACCCAGTTCCTGCCCCATTTTCTACTATTGAGCTTGATAATAGAAGAGTCAATGAAGGAGGTAGTATTCAAAAACTTATATTATTTATTCGAGGAAAGGCTATATCAGGGGCTCCTAATATTAAAGGAACTAATCAATTTCCAAATTCCTCCA

>HT_HK_71M6

AGTGTTCCGTCCAAAATAGGATCTCCTCCTCCGATTGGATCAAAGAAAGATGTATTTAAGTTTCGGTCTGTTAATAACATAGTAATAGCTCCAGCTAAAACAGGAAGAGAAAGAAGTAATAAGATAGCTGTAATAACTACAGATCAAACAAATAARGGTAGTCGGTCTAAAGTAATTCCTGACGATCGTATATTAATTACAGTTGTAATAAAATTTACTGCCCCTAAAATTGAGGAAATTCCAGCTAAATGAAGAGAAAAAATAGCTTAATCAACAGAAGCTCCAGCATGAGCTGTTCCTGAAGAGAGAGGAGGATAAACTGTTCACCCAGTTCCTGCCCCATTTTCTACTATTGAGCTTGATAATAGAAGAGTCAATGAAGGAGGTAGTATTCAAAAACTTATATTATTTATTCGAGGAAAGGCTATATCAGGGGCTCCTAATATTAAAGGAACTAATCAATTTCCAAATTCCCCCCCA

>HT_HK_71M7

GTGTTCCGTCCAAAATAGGATCTCCTCCTCCGATTGGATCAAAGAAAGATGTATTTAAGTTTCGGTCTGTTAATAACATAGTAATAGCTCCAGCTAAAACAGGAAGAGAAAGAAGTAATAAGATAGCTGTAATAACTACAGATCAAACAAATAARGGTAGTCGGTCTAAAGTAATTCCTGACGATCGTATATTAATTACAGTTGTAATAAAATTTACTGCCCCTAAAATTGAGGAAATTCCAGCTAAATGAAGAGAAAAAATAGCTAAATCAACAGAAGCTCCAGCATGAGCTGTTCCTGAAGAGAGAGGAGGATAAACTGTTCACCCAGTTCCTGCCCCATTTTCTACTATTGAGCTTGATAATAGAAGAGTCAATGAAGGAGGTAGTATTCAAAAACTTATATTATTTATTCGAGGAAAGGCTATATCAGGGGCTCCTAATATTAAAGGAACTAATCAATTTCCAAATTCCCTCCA

>HT_HK_71M8

AAAATAGGATCTCCTCCTCCGATTGGATCAAAGAAAGATGTATTTAAGTTTCGGTCTGTTAATAACATAGTAATAGCTCCAGCTAAAACAGGAAGAGAAAGAAGTAATAAGATAGCTGTAATAACTACAGATCAAACAAATAAAGGTAGTCGATCTAAAGTAATTCCTGACGATCGTATATTAATTACAGTTGTAATAAAATTTACTGCCCCTAAAATTGAGGAAATTCCAGCTAAATGAAGAGAAAAAATAGCTAAATCAACAGAAGCTCCAGCATGAGCTGTTCCTGAAGAGAGAGGAGGATAAACTGTTCACCCAGTTCCTGCCCCATTTTCTACTATTGAGCTTGATAATAGAAGAGTCAATGAAGGAGGTAGTATTCAAAAACTTATATTATTTATTCGAGGAAAGGCTATATCAGGGGCTCCTAATATTAAAGGAACTAATCAATTTCCAAATTCCCTCCA

>HT_HK_71M9

TATCAGATTTACGTGTTCCGTATAAAATAGGATCTCCTCCTCCGATTGGATCAAAGAAAGATGTATTTAAGTTTCGGTCTGTTAATAACATAGTAATAGCTCCAGCTAAAACAGGAAGAGAAAGAAGTAATAAGATAGCTGTAATAACTACAGATCAAACAAATAARGGTAGTCGATCTAAAGTAATTCCTGACGATCGTATATTAATTACAGTTGTAATAAAATTTACTGCCCCTAAAATTGAGGAAATTCCAGCTAAATGAAGAGAAAAAATAGCTAAATCAACAGAAGCTCCAGCATGAGCTGTTCCTGAAGAGAGAGGAGGATAAACTGTTCACCCAGTTCCTGCCCCATTTTCTACTATTGAGCTTGATAATAGAAGAGTCAATGAAGGAGGTAGTATTCAAAAACTTATATTATTTATTCGAGGAAAGGCTATATCAGGGGCTCCTAATATTAAAGGAACTAATCATTTCCAAATTCCCCCCA

>HT_HK_71M10

AAAATAGGATCTCCTCCTCCGATTGAGATCAAAGAAAGTTGTATTTAAGTTTCGGTCTGTTAATAACATAGTAATAGCTCCAGCTAAAACAGGAAGAGAAAGAAGTAATAAGATAGCTGTAATAACTACAGATCAAACAAATAARGGTAGTCGATCTAAAGTAATTCCTGACGATCGTATATTAATTACAGTTGTAATAAAATTTACTGCCCCTAAAATTGAGGAAATTCCAGCTAAATGAAGAGAAAAAATAGCTTAATCAACAGAAGCTCCAGCATGAGCTGTTCCTGAAGAGAGAGGAGGATAAACTGTTCACCCAGTTCCTGCCCCATTTTCTACTATTGAGCTTGATAATAGAAGAGTCAATGAAGGAGGTAGTATTCAAAAACTTATATTATTTATTCGAGGAAAGGCTATATCAGGGGCTCCTAATATTAAAGGAACTAATCAATTTCCAAATTCCTCCA

>HT_HK_71M11

ATTTACGTGTTCCGTATAAAATAGGATCTCCTCCTCCGATTGGATCAAAGAAAGATGTATTTAAGTTTCGGTCTGTTAATAACATAGTAATAGCTCCGCTAAAACAGGAAGAGAAAGAAGTAATAAGATAGCTGTAATAACTACAGATCAAACAAATAARGGTAGTCGATCTAAAGTAATTCCTGACGATCGTATATTAATTACAGTTGTAATAAAATTTACTGCCCCTAAAATTGAGGAAATTCCAGCTAAATGAAGAGAAAAAATAGCTAAATCAACAGAAGCTCCAGCATGAGCTGTTCCTGAAGAGAGAGGAGGATAAACTGTTCACCCAGTTCCTGCCCCATTTTCTACTATTGAGCTTGATAATAGAAGAGTCAATGAAGGAGGTAGTATTCAAAAACTTATATTATTTATTCGAGGAAAGGCTATATCAGGGGCTCCTAATATTAAAGGAACTAATCAATTTCCAAAATCCTCCTA

>HT_HK_71M12

TTTACCTGTTCCGTATAAAATAGGATCTCCTCCTCCGATTGGATCAAAGAAAGATGTATTTAAGTTTCGGTCTGTTAATAACATAGTAATAGCTCCAGCTAAAACAGGAAGAGAAAGAAGTAATAAGATAGCTGTAATAACTACAGATCAAACAAATAAAGGTAGTCGGTCTAAAGTAATTCCTGACGATCGTATATTAATTACAGTTGTAATAAAATTTACTGCCCCTAAAATTGAGGAAATTCCAGCTAAATGAAGAGAAAAAATAGCTAAATCAACAGAAGCTCCAGCATGAGCTGTTCCTGAAGAGAGAGGAGGATAAACTGTTCACCCAGTTCCTGCCCCATTTTCTACTATTGAGCTTGATAATAGAAGAGTCAATGAAGGAGGTAGTATTCAAAAACTTATATTATTTATTCGAGGAAAGGCTATATCAGGGGCTCCTAATATTAAAGGAACTAATCAATTTCCAAATTCCTCCA

>HT_HK_71M13

TCAGATAACGTGTTCCGTATAAAATAGGATCTCCTCCTCCGATTGGATCAAAGAAAGATGTATTTAAGTTTCGGTCTGTTAATAACATAGTAATAGCTCCAGCTAAAACAGGAAGAGAAAGAAGTAATAAGATAGCTGTAATAACTACAGATCAAACAAATAAAGGTAGTCGATCTAAAGTAATTCCTGACGATCGTATATTAATTACAGTTGTAATAAAATTTACTGCCCCTAAAATTGAGGAAATTCCAGCTAAATGAAGAGAAAAAATAGCTAAATCAACAGAAGCTCCAGCATGAGCTGTTCCTGAAGAGAGAGGAGGATAAACTGTTCACCCAGTTCCTGCCCCATTTTCTACTATTGAGCTTGATAATAGAAGAGTCAATGAAGGAGGTAGTATTCAAAAACTTATATTATTTATTCGAGGAAAGGCTATATCAGGGGCTCCTAATATTAAAGGAACTAATCAATTTCCAAATTCCCCCCA

>HT_HK_72F1

CGCCTAATACTACTCAATTTACAAATGCCTACCCAATAGGGTCTCCTCCTCCCATTGGATCAAAAAAAGATGTATTTAAATTTCGGTCTGTTAATAATATAGTAATAGCTCCGGCTAATACGGGTAGAGAAAGAAGTAATAAAATAGCTGTAATTACTACTGATCACACAAATAAAGGTAGTCGATCAAGAGTAATACCAGCTGATCGTATATTAATTACAGTTGTAATAAAATTTACTGCTCCTAAAATAGATGAGATTCCCGCTAAATGTAAAGAAAAAATTGCTAAATCAACTGAAGCCCCAGCATGAGCTGTTCCAGAAGAAAGGGGAGGATAAACCGTTCACCCTGTTCCAGCTCCGTTTTCTACTATAGAACTAGAAAGCAGCAGTGTTAAAGAGGGGGGTAATATTCAAAAACTTATATTATTTATTCGAGGAAAAGCTATATCAGGGGCTCCTAGTATTAAGGGAACTAATCAATTTCCAAAATCCTCCCA

>HT_HK_72F2

ATCCTCGGTCTACTCAATTTATCGTATGATATCCAACAGGGTCTCCTTCCTCTCCTTGCAATCAAAAAAAAATGTATTTAAATTTCGGTCTGTTAATAATATAGTAATAGCTCCGGCTAATACGGGTAGAGAAAGAAGTAATAAAATAGCTGTAATTACTACTGATCACACAAATAAAGGTAGTCGATCAAGAGTAATACCAGCTGATCGTATATTAATTACAGTTGTAATAAAATTTACTGCTCCTAAAATAGATGAGATTCCCGCTAAATGTAAAGAAAAAATTGCTAAATCAACTGAAGCCCCAGCATGAGCTGTTCCAGAAGAAAGGGGAGGATAAACCGTTCACCCTGTTCCAGCTCCGTTTTCTACTATAGAACTTGAAAGCAGCAGTGTTAAAGAGGGGGGTAATATTCAAAAACTTATATTATTTATTCGAGGAAAAGCTATATCAGGGGCTCCTAGTATTAAGGGAACTAATCAATTTCCAAATCCTCCCA

>HT_HK_72F3

CGCTAGACTACTCAATTTCCAAATGCCTATCCAACAGGGTCTCCCCCTCCCCTTCGCAATCATAAAAAGATGTATTTTAATTTAGGTCTGTTAATAATATAGTAATAGCTCCGGCTAATACGGGTAGAGAAAGAAGTAATAAAATAGCTGTAATTACTACTGATCACACAAATAAAGGTAGTCGATCAAGAGTAATACCAGCTGATCGTATATTAATTACAGTTGTAATAAAATTTACTGCTCCTAAAATAGATGAGATTCCCGCTAAATGTAAAGAAAAAATTGCTAAATCAACTGAAGCTCCAGCATGAGCTGTTCCAGAAGAAAGGGGAGGATAAACCGTTCACCCTGTTCCAGCTCCGTTTTCTACTATAGAACTAGAAAGCAGCAGTGTTAAAGAGGGGGGTAATATTCAAAAACTTATATTATTTATTCGAGGAAAAGCTATATCAGGGGCTCCTAGTATTAAGGGAACTAATCAATTTCCAAAATCCTCCCA

>HT_HK_72F4

ATGCTTAAGTACTACTCAATTTCAGAATCCTATCGAATAGGGTCTCTCCCCTCCCATTCGCGATCAAAAAAAGATGTATTTAAATTTCGGTCTGTTAATAATATAGTAATAGCTCCGGCTAATACGGGTAGAGAAAGAAGTAATAAAATAGCTGTAATTACTACTGATCACACAAATAAAGGTAGTCGATCAAGAGTAATACCAGCTGATCGTATATTAATTACAGTTGTAATAAAATTTACTGCTCCTAAAATAGATGAGATTCCCGCTAAATGTAAAGAAAAAATTGCTAAATCAACTGAAGCCCCAGCATGAGCTGTTCCAGAAGAAAGGGGAGGATAAACCGTTCACCCTGTTCCAGCTCCGTTTTCTACTATAGAACTAGAAAGCAGCAGTGTTAAAGAGGGGGGTAATATTCAAAAACTTATATTATTTATTCGAGGAAAA

GCTATATCAGGGGCTCCTAGTATTAAGGGAACTAATCAATTTCCAAAATCCTCCCA

>HT_HK_72F5

ATCTAAGTCTACTCAATTTACCGTATGATACCCAACTAGTGTCTTCTCCATATCCTTCGCGATACTAATCAAGTATGTCTTTTCCTTCCGGTCTGTTAATAATATAGTAATAGCTCCGGCTAATACGGGTAGAGAAAGAAGTAATAAAATAGCTGTAATTACTACTGATCACACAAATAAAGGTAGTCGATCAAGAGTAATACCAGCTGATCGTATATTAATTACAGTTGTAATAAAATTTACTGCTCCTAAAATAGATGAGATTCCCGCTAAATGTAAAGAAAAAATTGCTAAATCAACTGAAGCCCCAGCATGAGCTGTTCCAGAAGAAAGGGGAGGATAAACCGTTCACCCTGTTCCAGCTCCGTTTTCTACTATAGAACTAGAAAGCAGCAGTGTTAAAGAGGGGGGTAATATTCAAAAACTTATATTATTTATTCGAGGAAAAGCTATATCAGGGGCTCCTAGTATTAAGGGAACTAATCAATTTCAAAATCCTCCCA

>HT_HK_72F6

TTCGCGTATCAAATAGGATCTCCTCCTCCGATTGAGATCAAAGAAAGATGTATTTAAGTTTCGGTCTGTTAATAACATAGTAATAGCTCCAGCTAAAACAGGAAGAGAAAGAAGTAATAAGATAGCTGTAATAACTACAGATCAAACAAATAAAGGTAGTCGATCTAAAGTAATTCCTGACGATCGTATATTAATTACAGTTGTAATAAAATTTACTGCCCCTAAAATTGAGGAAATTCCAGCTAAATGAAGAGAAAAAATAGCTAAATCAACAGAAGCTCCAGCATGAGCTGTTCCTGAAGAGAGAGGAGGATAAACTGTTCACCCAGTTCCTGCCCCATTTTCTACTATTGAGCTTGATAATAGAAGAGTCAATGAAGGAGGTAGTATTCAAAAACTTATATTATTTATTCGAGGAAAGGCTATATCAGGGGCTCCTAATATTAAAGGAACTAATCAATTTCCAAATTCCCTCCCA

>HT_HK_72F7

ACTCCTACGAACTACTCAATTTCCCTATCCTACCCAATAGGGTCTCCCCCTCCCATTGCGATCAAAAAAAGATGTATTTAAATTTAGGTCTGTTAATAATATAGTAATAGCTCCGGCTAATACGGGTAGAGAAAGAAGTAATAAAATAGCTGTAATTACTACTGATCACACAAATAAAGGTAGTCGATCAAGAGTAATACCAGCTGATCGTATATTAATTACAGTTGTAATAAAATTTACTGCTCCTAAAATAGATGAGATTCCCGCTAAATGTAAAGAAAAAATTGCTAAATCAACTGAAGCCCCAGCATGAGCTGTTCCAGAAGAAAGRGGAGGATAAACCGTTCACCCTGTTCCAGCTCCGTTTTCTACTATAGAACTAGAAAGCAGCAGTGTTAAAGAGGGRGGTAATATTCAAAAACTTATATTATTTATTCGAGGAAAAGCTATATCAGGGGCTCCTAGTATTAAGGGAACTAATCAATTTCAAAAATCCTCCCA

>HT_HK_73M1

ATCCTAGAACTACTCAATTTCCAAATCCTACCCAATAGGGTCTCCCCCTCCCCTTGCGATCATAAAAAGATGTATTTAAATTTCGGTCTGTTAATAATATAGTAATAGCTCCGGCTAATACGGGTAGAGAAAGAAGTAATAAAATAGCTGTAATTACTACTGATCACACAAATAAAGGTAGTCGATCAAGAGTAATACCAGCTGATCGTATATTAATTACAGTTGTAATAAAATTTACTGCTCCTAAAATAGATGAGATTCCCGCTAAATGTAAAGAAAAAATTGCTAAATCAACTGAAGCCCCAGCATGAGCTGTTCCAGAAGAAAGGGGAGGATAAACCGTTCACCCTGTTCCAGCTCCGTTTTCTACTATAGAACTAGAAAGCAGCAGTGTTAAAGAGGGGGGTAATATTCAAAAACTTATATTATTTATTCGAGGAAAAGCTATATCAGGGGCTCCTAGTATTAAGGGAACTAATCAATTTCAAAAATCCTCCCA

>HT_HK_73M2

CTCGTAC

AACTACTCATTTCCGTATCCTACCCAATCGGGTCTCCCCCTCCCATTGCGATCAAAAAAAGATGTATTTAAATTTAGGTCTGTTAATAATATAGTAATAGCTCCGGCTAATACGGGTAGAGAAAGAAGTAATAAAATAGCTGTAATTACTACTGATCACACAAATAAAGGTAGTCGATCAAGAGTAATACCAGCTGATCGTATATTAATTACAGTTGTAATAAAATTTACTGCTCCTAAAATAGATGAGATTCCCGCTAAATGTAAAGAAAAAATTGCTAAATCAACTGAAGCCCCAGCATGAGCTGTTCCAGAAGAAAGGGGAGGATAAACCGTTCACCCTGTTCCAGCTCCGTTTTCTACTATAGAACTAGAAAGCAGCAGTGTTAAAGAGGGGGGTAATATTCAAAAACTTATATTATTTATTCGAGGAAAAGCTATATCAGGGGCTCCTAGTATTAAGGGAACTAATCAATTTCAAAAATCCTCCCA

>HT_HK_73M4

CTCAGGCGATCTACTCAATTCACCTCCGATCCCAACTCGGTACTTCTCCATCTCCTACGCGATACTAATCATATTTCTTATCCTTCCGGTCTGTTAATAATATAGTAATAGCTCCGGCTAATACGGGTAGAGAAAGAAGTAATAAAATAGCTGTAATTACTACTGATCACACAAATAAAGGTAGTCGATCAAGAGTAATACCAGCTGATCGTATATTAATTACAGTTGTAATAAAATTTACTGCTCCTAAAATAGATGAGATTCCCGCTAAATGTAGAGAAAAAATTGCTAAATCAACTGAAGCCCCAGCATGAGCTGTTCCAGAAGAAAGGGGAGGATAAACCGTTCACCCTGTTCCAGCTCCGTTTTCTACTATAGAACTAGAAAGCAGCAGTGTTAAAGAGGGGGGTAATATTCAAAAACTTATATTATTTATTCGAGGAAAAGCTATATCAGGGGCTCCTAGTATTAAGGGAACTAATCAATTTCCAAATCCTCCCA

>HT_HK_73M5

ATCGTACGTACTACTCAATTTACCAAATGCATATCAAATAGGGTCTCCTCCTCCCCTTGCGATCAAAAAAAGATGTATTTAAATTTCGGTCTGTTAATAATATAGTAATAGCTCCGGCTAATACGGGTAGAGAAAGAAGTAATAAAATAGCTGTAATTACTACTGATCATACAAATAAAGGTAGTCGATCAAGAGTAATACCAGCTGATCGTATATTAATTACAGTTGTAATAAAATTTACTGCTCCTAAAATAGATGAGATTCCCGCTAAATGTAAAGAAAAAATTGCTAAATCAACTGAAGCCCCAGCATGAGCTGTTCCAGAAGAAAGGGGAGGATAAACCGTTCACCCTGTTCCAGCTCCGTTTTCTACTATAGAACTAGAAAGCAGCAGTGTTAAAGAGGGGGGTAATATTCAAAAACTTATATTATTTATTCGAGGAAAAGCTATATCAGGGGCTCCTAGTATTAAGGGAACTAATCAATTTCCAAATCCTCCCA
