# Supplementary material for: Incidence of dengue and chikungunya viruses in mosquitoes and human patients in border provinces of Vietnam
Source: Parasit Vectors. 2017 Nov 9;10:556. doi: 10.1186/s13071-017-2422-z (PMC5680899; doi:10.1186/s13071-017-2422-z)
Supplement: Supplementary file 3 — Distribution of haplotypes of captured adult mosquitoes (DOCX 31 kb) [file 13071_2017_2422_MOESM3_ESM.docx]

**Additional file 3: Table S3.** Distribution of haplotypes of captured adult mosquitoes

**Strain Species Haplotype (Accession number) ^a^ Country**

**DN_DL_01F1 *Aedes aegypti* Vietnam 5 (JQ926687) Vietnam**

**DN_DL_01F2 *Aedes aegypti* Cambodia 3 (JQ926690) Cambodia**

**DN_DL_01F3 *Aedes aegypti* Martinique 1 (JQ926696) Martinique (French Caribbean)**

**DN_DL_01F4 *Aedes aegypti* Martinique 1 (JQ926696) Martinique (French Caribbean)**

# DN_DL_01F5 *Aedes aegypti* BU-Zoo-Ae.a-31 (KT339683) India

**DN_DL_01F6 *Aedes aegypti* Martinique 1 (JQ926696) Martinique (French Caribbean)**

**DN_DL_01F7 *Aedes aegypti*  Vietnam 5 (JQ926687) Vietnam**

**DN_DL_01F8 *Culex vishnui* 564IRI2008 (AB738195) Unknown**

**DN_DL_01F9 *Aedes aegypti* TG13-Haplotype III (KT313648) Tonga**

**DN_DL_01F10 *Aedes aegypti*  Vietnam 5 (JQ926687) Vietnam**

**DN_DL_02F1 *Aedes aegypti* Martinique 1 (JQ926696) Martinique (French Caribbean)**

**DN_DL_02F2 *Aedes aegypti* Martinique 1 (JQ926696) Martinique (French Caribbean)**

**DN_DL_02F3 *Aedes aegypti*  Vietnam 5 (JQ926687) Vietnam**

**DN_DL_02F4 *Aedes aegypti* Martinique 1 (JQ926696) Martinique (French Caribbean)**

**DN_DMi_03F1 *Aedes aegypti* Martinique 1 (JQ926696) Martinique (French Caribbean)**

**DN_DMi_03F2 *Aedes aegypti* BU-Zoo-Ae.a-08 (KT339660) India**

**DN_DMi_03F3 *Aedes aegypti* Martinique 1 (JQ926696) Martinique (French Caribbean)**

**DN_DMi_03F4 *Aedes aegypti* Martinique 1 (JQ926696) Martinique (French Caribbean)**

**DN_DMi_03F5 *Aedes aegypti* Martinique 1 (JQ926696) Martinique (French Caribbean)**

**DN_DMi_03F6 *Aedes aegypti* Martinique 1 (JQ926696) Martinique (French Caribbean)**

**DN_DMi_03F7 *Aedes aegypti* Martinique 1 (JQ926696) Martinique (French Caribbean)**

**DN_DMi_03F8 *Aedes aegypti* Martinique 1 (JQ926696) Martinique (French Caribbean)**

**DN_DMi_03F9 *Aedes aegypti* Martinique 1 (JQ926696) Martinique (French Caribbean)**

**DN_DMi_04F1 *Aedes aegypti* Martinique 1 (JQ926696) Martinique (French Caribbean)**

**DN_DMi_04F2 *Aedes aegypti* Martinique 1 (JQ926696) Martinique (French Caribbean)**

**DN_DMi_04F3 *Aedes aegypti* Martinique 1 (JQ926696) Martinique (French Caribbean)**

**DN_DMi_04F4 *Aedes aegypti* Martinique 1 (JQ926696) Martinique (French Caribbean)**

**DN_DMi_04F5 *Aedes aegypti* Martinique 1 (JQ926696) Martinique (French Caribbean)**

**DN_DMi_04F6 *Aedes aegypti* Martinique 1 (JQ926696) Martinique (French Caribbean)**

**DN_DMi_04F7 *Aedes aegypti* Martinique 1 (JQ926696) Martinique (French Caribbean)**

**DN_DMi_04F8 *Aedes aegypti* Martinique 1 (JQ926696) Martinique (French Caribbean)**

**DN_DMi_05F1 *Aedes aegypti* BU-Zoo-Ae.a-31 (KT339683) India**

**DN_DMi_05M1 *Aedes aegypti* haplotype 62 (KM203201) Colombia**

**DN_DMi_06F1 *Aedes aegypti* Martinique 1 (JQ926696) Martinique (French Caribbean)**

**DN_DMi_06F2 *Aedes aegypti* Martinique 1 (JQ926696) Martinique (French Caribbean)**

**DN_DMi_06F3 *Aedes aegypti* Isolate 4 (JX456414) Brazil**

**DN_DMi_06F4 *Aedes aegypti* BU-Zoo-Ae.a-08 (KT339660) India**

**DN_DMi_06F5 *Aedes aegypti* Martinique 1 (JQ926696) Martinique (French Caribbean)**

**DN_DL_07M1 *Aedes aegypti* BU-Zoo-Ae.a-30 (KT339682) India**

**DN_DL_07M2 *Aedes aegypti* Martinique 1 (JQ926696) Martinique (French Caribbean)**

**DN_DL_07M3 *Aedes aegypti* Cambodia 3 (JQ926690) Cambodia**

**DN_DL_07M4 *Aedes aegypti* BU-Zoo-Ae.a-30 (KT339682) India**

**DN_DL_07M6 *Aedes albopictus* VAITC4665 (KU495082) Australia**

**DN_DL_07M7 *Aedes aegypti* Vietnam 5 (JQ926687) Vietnam**

**DN_DL_07M8 *Aedes aegypti* BU-Zoo-Ae.a-30 (KT339682) India**

**DN_DL_07M9 *Aedes aegypti* Martinique 1 (JQ926696) Martinique (French Caribbean)**

**DN_DL_07M10 *Aedes aegypti* Martinique 1 (JQ926696) Martinique (French Caribbean)**

**DN_DM_8F1 *Aedes aegypti* Unknown (AJ970971) Vietnam**

**DN_DM_8F2 *Aedes aegypti* BU-Zoo-Ae.a-08 (KT339660) India**

**DN_DM_8F3 *Aedes aegypti* Unknown (AJ970971) Vietnam**

**DN_DM_8F4 *Aedes aegypti* VAITC4689 (KU495081) Australia**

**DN_DM_8F5 *Aedes aegypti* VAITC4689 (KU495081) Australia**

**DN_DM_8F6** ***Aedes aegypti* VAITC4689 (KU495081) Australia**

**DN_DM_9M1 *Aedes aegypti* Martinique 1 (JQ926696) Martinique (French Caribbean)**

**DN_DM_9M2**  ***Aedes aegypti* VAITC4689 (KU495081) Australia**

**DN_DM_9M3**  ***Aedes aegypti* VAITC4689 (KU495081) Australia**

**DN_DM_9M4 *Aedes aegypti* TIN2 (KX227743) India**

**DN_DM_9M5 *Aedes aegypti* VAITC4689 (KU495081) Australia**

**LA_BH_13F1 *Aedes aegypti* Cambodia 3 (JQ926690) Cambodia**

**LA_BH_13F2 *Aedes aegypti* Cambodia 3 (JQ926690) Cambodia**

**LA_BH_13F3 *Aedes aegypti* BU-Zoo-Ae.a-30 (KT339682) India**

**LA_BH_13F4 *Aedes aegypti* Cambodia 3 (JQ926690) Cambodia**

**LA_BH_13F5 *Aedes aegypti* Unknown (AJ970971) Vietnam**

**LA_BHT_14F1 *Aedes aegypti* Cambodia 3 (JQ926690) Cambodia**

**LA_BHT_14F2 *Aedes albopictus*  Unknown (HF536717) Romania**

**LA_BHT_14F3 *Aedes aegypti*  haplotype 19 (KM203158) Colombia**

**LA_BHT_14F4 *Aedes aegypti* VAITC4689 (KU495081) Australia**

**LA_BHT_14F5 *Aedes aegypti* Cambodia 3 (JQ926690) Cambodia**

**LA_BHT_14F6 *Aedes aegypti* Unknown (AJ970971) Vietnam**

**LA_BHT_14F7 *Aedes aegypti* Cambodia 3 (JQ926690) Cambodia**

**LA_BHT_14F8 *Aedes aegypti* BU-Zoo-Ae.a-31 (KT339683) India**

**LA_BHT_14F9 *Aedes aegypti* BU-Zoo-Ae.a-31 (KT339683) India**

**LA_BHT_15F1 *Aedes aegypti* Unknown (AJ970971) Vietnam**

**LA_BHT_15F2 *Aedes aegypti* Unknown (AJ970971) Vietnam**

**LA_BHT_15F3 *Aedes aegypti* BU-Zoo-Ae.a-31 (KT339683) India**

**LA_BHT_15F4 *Aedes aegypti* Unknown (AJ970971) Vietnam**

**LA_BHT_15F5 *Aedes aegypti* BU-Zoo-Ae.a-31 (KT339683) India**

**LA_BHT_15F6 *Aedes aegypti* BU-Zoo-Ae.a-31 (KT339683) India**

**LA_BHT_15F7 *Aedes aegypti* BU-Zoo-Ae.a-31 (KT339683) India**

**LA_BHT_15F8 *Aedes aegypti* Cambodia 3 (JQ926690) Cambodia**

**LA_BHT_15F9 *Aedes aegypti* Cambodia 3 (JQ926690) Cambodia**

**LA_BHT_15F10 *Aedes aegypti* Cambodia 3 (JQ926690) Cambodia**

**LA_BHT_16F1 *Aedes aegypti* Cambodia 3 (JQ926690) Cambodia**

**LA_BHT_16F2 *Aedes aegypti* Cambodia 3 (JQ926690) Cambodia**

**LA_BHT_16F3 *Aedes aegypti* Cambodia 3 (JQ926690) Cambodia**

**LA_BHT_16F4 *Aedes aegypti* BU-Zoo-Ae.a-31 (KT339683) India**

**LA_BHT_16F5 *Aedes aegypti* Cambodia 3 (JQ926690) Cambodia**

**LA_BHT_16F6 *Aedes aegypti* Cambodia 3 (JQ926690) Cambodia**

**LA_BHT_16F7 *Aedes aegypti* Cambodia 3 (JQ926690) Cambodia**

**LA_BHT_16F8 *Aedes aegypti* Martinique 1 (JQ926696) Martinique (French Caribbean)**

**LA_BHT_16F9 *Aedes aegypti* Cambodia 3 (JQ926690) Cambodia**

**LA_BHT_16F10 *Aedes aegypti* Cambodia 3 (JQ926690) Cambodia**

**LA_BHT_17F1 *Aedes aegypti* BU-Zoo-Ae.a-31 (KT339683) India**

**LA_BHT_17F2 *Aedes aegypti* BU-Zoo-Ae.a-31 (KT339683) India**

**LA_BHT_17F3 *Aedes aegypti* Cambodia 3 (JQ926690) Cambodia**

**LA_BHT_17F4 *Aedes albopictus* Tez5 (KT260200) India**

**LA_BHT_17F5 *Aedes aegypti* Cambodia 3 (JQ926690) Cambodia**

**LA_BHT_17F6 *Aedes albopictus* None (HF536717) Romania**

**LA_BHT_17F7 *Aedes aegypti* BU-Zoo-Ae.a-31 (KT339683) India**

**LA_BHT_17F8 *Aedes aegypti* Cambodia 3 (JQ926690) Cambodia**

**LA_BHT_17F9 *Aedes aegypti* BU-Zoo-Ae.a-31 (KT339683) India**

**LA_BHT_17F10 *Aedes aegypti*  BU-Zoo-Ae.a-30 (KT339682) India**

**LA_BHT_18F1 *Aedes albopictus* Unknown (HF536717) Romania**

**LA_BHT_18F2 *Aedes aegypti* Cambodia 3 (JQ926690) Cambodia**

**LA_BHT_19M1 *Aedes aegypti* Cambodia 3 (JQ926690) Cambodia**

**LA_BHT_19M2 *Aedes aegypti* Cambodia 3 (JQ926690) Cambodia**

**LA_BHT_19M3 *Aedes aegypti*  BU-Zoo-Ae.a-31 (KT339683) India**

**LA_BHT_19M4 *Aedes aegypti*  BU-Zoo-Ae.a-30 (KT339682) India**

**LA_BHT_19M5 *Aedes aegypti* Martinique 1 (JQ926696) Martinique (French Caribbean)**

**LA_BHT_19M6 *Aedes aegypti* Cambodia 3 (JQ926690) Cambodia**

**LA_BHT_19M7 *Aedes aegypti*  BU-Zoo-Ae.a-31 (KT339683) India**

**LA_BHT_19M8 *Aedes aegypti*  VAITC4689 (KU495081) Australia**

**LA_BHT_19M9 *Aedes aegypti*  BU-Zoo-Ae.a-31 (KT339683) India**

**LA_BHT_19M10 *Aedes aegypti* aeg7 (KP843388) Thailand**

**LA_BHT_19M11 *Aedes aegypti* Martinique 1 (JQ926696) Martinique (French Caribbean)**

**LA_BHT_19M12 *Aedes aegypti* Vietnam 5 (JQ926687) Vietnam**

**LA_BHT_19M13 *Aedes albopictus* None (HF536717) Romania**

**LA_BHT_19M14 *Aedes aegypti*  BU-Zoo-Ae.a-31 (KT339683) India**

**LA_BHT_19M15 *Aedes aegypti* BEN361_1 (KX446417) Benin**

**LA_BHT_19M16 *Aedes aegypti*  BU-Zoo-Ae.a-31 (KT339683) India**

**LA_BHT_19M17 *Aedes aegypti* Haplotype 62 (KM203201) Colombia**

**LA_BHT_19M18 *Aedes aegypti*  BU-Zoo-Ae.a-31 (KT339683) India**

**LA_BHT_19M19 *Aedes aegypti* Cambodia 3 (JQ926690) Cambodia**

**LA_BHT_19M20 *Aedes aegypti* CDC11 (KM452747) Colombia**

**LA_BHT_19M21 *Aedes aegypti* aeg7 (KP843388) Thailand**

**LA_BHT_19M22 *Aedes aegypti* Unknown (NA) Unknown**

**LA_BHT_19M23 *Aedes aegypti* Haplotype 37 (KM203176) Colombia**

**LA_BHT_19M24 *Aedes aegypti* Unknown (NA) Unknown**

**LA_BHT_19M25 *Aedes aegypti* Haplotype 80 (KM203219) Colombia**

**LA_BHT_19M26 *Aedes aegypti* BUZOO-M-Aa (KR817731) India**

**LA_BHT_19M27 *Aedes aegypti*  BU-Zoo-Ae.a-31 (KT339683) India**

**LA_BHT_19M29 *Aedes aegypti* Guinea 1 (JQ926700) Guinea**

**LA_BHT_19M30 *Aedes aegypti* Cambodia 3 (JQ926690) Cambodia**

**LA_BHT_19M31 *Aedes aegypti* Cambodia 3 (JQ926690) Cambodia**

**LA_BHT_19M32 *Aedes aegypti*  Unknown (NA) Unknown**

**LA_BHT_19M33 *Aedes aegypti* BUZOO-M-Aa (KR81773) India**

**LA_BHT_19M34 *Aedes aegypti* Haplotype 68 (KM203207) Colombia**

**LA_BH_20M1 *Aedes aegypti* Haplotype 94 (KM203233) Colombia**

**LA_BH_20M2 *Aedes aegypti* Cambodia 3 (JQ926690) Cambodia**

**LA_BH_20M3 Unknown Unknown Unknown**

**LA_BH_20M4 *Aedes aegypti* Haplotype VI (KT313652) French Polynesia**

**LA_TT_22F3 *Aedes aegypti* BU-Zoo-Ae.a-31 (KT339683) India**

**LA_TT_22F4 Unknown Unknown Unknown**

**LA_TT_22F5 *Aedes albopictus* None (HF536717) Romania**

**LA_TT_22F6 *Aedes aegypti* Cambodia 3 (JQ926690) Cambodia**

**LA_TT_22F7 *Aedes aegypti* U10_7 (KX446442) Uganda**

**LA_TT_22F8 *Aedes aegypti* BU-Zoo-Ae.a-31 (KT339683) India**

**LA_TT_22F9 *Aedes aegypti* Cambodia 3 (JQ926690) Cambodia**

**LA_TT_22F10 *Aedes aegypti* Haplotype 62 (KM203201) Colombia**

**LA_TT_23F1 *Aedes aegypti* U10_16 (KX446448) Uganda**

**LA_TT_23F2 *Aedes aegypti* BU-Zoo-Ae.a-31 (KT339683) India**

**LA_TT_23F3 *Aedes aegypti* BU-Zoo-Ae.a-31 (KT339683) India**

**LA_TT_23F4 *Aedes aegypti* BU-Zoo-Ae.a-31 (KT339683) India**

**LA_TT_23F5 *Aedes aegypti* BU-Zoo-Ae.a-31 (KT339683) India**

**LA_TT_23F6 *Aedes aegypti* BU-Zoo-Ae.a-31 (KT339683) India**

**LA_TT_23F7 *Aedes aegypti* Cambodia 3 (JQ926690) Cambodia**

**LA_TT_23F8 *Aedes aegypti* Cambodia 3 (JQ926690) Cambodia**

**LA_TT_23F9 *Aedes aegypti* BU-Zoo-Ae.a-31 (KT339683) India**

**LA_TT_23F10 *Aedes aegypti* Haplotype 62 (KM203201) Colombia**

**LA_TT_24F1 *Aedes aegypti* BU-Zoo-Ae.a-31 (KT339683) India**

**LA_TT_24F2 *Aedes aegypti* Cambodia 3 (JQ926690) Cambodia**

**LA_TT_24F3 *Aedes aegypti* Haplotype VI (KT313652) French Polynesia**

**LA_TT_24F4 *Aedes aegypti* BU-Zoo-Ae.a-31 (KT339683) India**

**LA_TT_24F5 *Aedes aegypti* Cambodia 3 (JQ926690) Cambodia**

**LA_TT_24F6 *Aedes aegypti* BU-Zoo-Ae.a-31 (KT339683) India**

**LA_TT_24F7 *Aedes aegypti* Haplotype 62 (KM203201) Colombia**

**LA_TT_24F8 *Aedes aegypti* Haplotype 62 (KM203201) Colombia**

**LA_TT_24F9 *Aedes aegypti* BU-Zoo-Ae.a-31 (KT339683) India**

**LA_TT_24F10 *Aedes aegypti* Haplotype VI (KT313652) French Polynesia**

**LA_TT_25F1 *Aedes aegypti* BU-Zoo-Ae.a-31 (KT339683) India**

**LA_TT_25F2 *Aedes aegypti* BU-Zoo-Ae.a-31 (KT339683) India**

**LA_TT_25F3 *Aedes aegypti* Cambodia 3 (JQ926690) Cambodia**

**LA_TT_25F4 *Aedes aegypti* BU-Zoo-Ae.a-31 (KT339683) India**

**LA_TT_25F5 *Aedes aegypti* BU-Zoo-Ae.a-31 (KT339683) India**

**LA_TT_25F6 *Aedes aegypti* BU-Zoo-Ae.a-31 (KT339683) India**

**LA_TT_25F7 *Aedes aegypti* Cambodia 3 (JQ926690) Cambodia**

**LA_TT_25F8 *Aedes aegypti* Cambodia 3 (JQ926690) Cambodia**

**LA_TT_25F9 *Aedes aegypti* Haplotype VI (KT313652) French Polynesia**

**LA_TT_25F10 *Aedes aegypti* BU-Zoo-Ae.a-31 (KT339683) India**

**LA_TT_26F1 *Aedes aegypti* Cambodia 3 (JQ926690) Cambodia**

**LA_TT_26F2 *Aedes aegypti* Cambodia 3 (JQ926690) Cambodia**

**LA_TT_26F3 *Aedes aegypti* BU-Zoo-Ae.a-31 (KT339683) India**

**LA_TT_26F4 *Aedes aegypti* BU-Zoo-Ae.a-31 (KT339683) India**

**LA_TT_26F5 *Aedes aegypti* BU-Zoo-Ae.a-31 (KT339683) India**

**LA_TT_26F6 *Aedes aegypti* BU-Zoo-Ae.a-31 (KT339683) India**

**LA_TT_26F7 *Aedes aegypti* BU-Zoo-Ae.a-31 (KT339683) India**

**LA_BT_28F1 *Aedes aegypti* Cambodia 3 (JQ926690) Cambodia**

**LA_BT_28F2 *Aedes aegypti* Haplotype 62 (KM203201) Colombia**

**LA_BT_28F3 *Aedes aegypti* BU-Zoo-Ae.a-31 (KT339683) India**

**LA_BT_28F4 *Aedes aegypti*  BU-Zoo-Ae.a-30 (KT339682) India**

**LA_BT_28F5 *Aedes aegypti*  aeg7 (KP843388) Thailand**

**LA_BT_28F6 *Aedes aegypti* BU-Zoo-Ae.a-31 (KT339683) India**

**LA_BT_28F7 *Aedes aegypti* BU-Zoo-Ae.a-31 (KT339683) India**

**LA_BT_28F8 *Aedes aegypti*  BU-Zoo-Ae.a-30 (KT339682) India**

**LA_BT_28F9 *Aedes aegypti* Haplotype 62 (KM203201) Colombia**

**LA_BT_28F10 *Aedes aegypti* Cambodia 3 (JQ926690) Cambodia**

**LA_BT_29F1 *Aedes aegypti* Haplotype VI (KT313652) French Polynesia**

**LA_BT_29F2 *Aedes aegypti* BU-Zoo-Ae.a-31 (KT339683) India**

**LA_BT_29F3 *Aedes aegypti*  Unknown (AJ970966) Guadeloupe (French Caribbean)**

**LA_BT_29F4 *Aedes aegypti* Haplotype 62 (KM203201) Colombia**

**LA_BT_29F5 *Aedes aegypti*  Cambodia 3 (JQ926690) Cambodia**

**LA_BT_29F6 *Aedes aegypti* Cambodia 3 (JQ926690) Cambodia**

**LA_BT_29F7 *Aedes aegypti*  Haplotype VI (KT313652) French Polynesia**

**LA_BT_29F8 *Aedes aegypti* Cambodia 3 (JQ926690) Cambodia**

**LA_BT_29F9 *Aedes aegypti*  BU-Zoo-Ae.a-31 (KT339683) India**

**LA_BT_29F10 *Aedes aegypti* BU-Zoo-Ae.a-31 (KT339683) India**

**LA_BT_30F1 *Aedes aegypti* BU-Zoo-Ae.a-31 (KT339683) India**

**LA_BT_30F2 *Aedes aegypti*  BU-Zoo-Ae.a-31 (KT339683) India**

**LA_BT_30F3 *Aedes aegypti* BU-Zoo-Ae.a-31 (KT339683) India**

**LA_BT_30F4 *Aedes aegypti*  Martinique 1 (JQ926696) Martinique (French Caribbean)**

**LA_BT_30F5 *Aedes aegypti*  BU-Zoo-Ae.a-30 (KT339682) India**

**LA_BT_30F6 *Aedes aegypti*  Cambodia 3 (JQ926690) Cambodia**

**LA_BT_30F7 *Aedes aegypti* BU-Zoo-Ae.a-31 (KT339683) India**

**LA_BT_30F8 *Aedes aegypti*  BU-Zoo-Ae.a-31 (KT339683) India**

**LA_BT_30F9 *Aedes aegypti* BU-Zoo-Ae.a-31 (KT339683) India**

**LA_BT_30F10 *Aedes aegypti*  Cambodia 3 (JQ926690) Cambodia**

**LA_BT_31F1 *Aedes aegypti* Haplotype 19 (KM203158) Colombia**

**LA_BT_31F2 *Aedes aegypti*  BU-Zoo-Ae.a-31 (KT339683) India**

**LA_BT_31F3 *Aedes aegypti*  Martinique 1 (JQ926696) Martinique (French Caribbean)**

**LA_BT_31F4 *Aedes aegypti* BU-Zoo-Ae.a-31 (KT339683) India**

**LA_BT_31F5 *Aedes aegypti*  BU-Zoo-Ae.a-31 (KT339683) India**

**LA_BT_31F6 *Aedes aegypti* BU-Zoo-Ae.a-31 (KT339683) India**

**LA_BT_31F7 *Aedes aegypti* Haplotype 62 (KM203201) Colombia**

**LA_BT_31F8 *Aedes aegypti* Haplotype 51 (KM203190) Colombia**

**LA_BT_31F9 *Aedes aegypti* BU-Zoo-Ae.a-30 (KT339682) India**

**LA_BT_31F10 *Aedes aegypti* BU-Zoo-Ae.a-31 (KT339683) India**

**LA_BT_32F1 *Aedes aegypti*  BU-Zoo-Ae.a-31 (KT339683) India**

**LA_BT_32F2 *Aedes aegypti* Martinique 1 (JQ926696) Martinique (French Caribbean)**

**LA_BT_32F3 *Aedes aegypti* Haplotype 62 (KM203201) Colombia**

**LA_BT_32F4 *Aedes aegypti*  BU-Zoo-Ae.a-31 (KT339683) India**

**LA_BT_32F5 *Aedes aegypti* BU-Zoo-Ae.a-31 (KT339683) India**

**LA_BT_32F6 *Aedes aegypti* BU-Zoo-Ae.a-31 (KT339683) India**

**LA_BT_32F7 *Aedes aegypti*  BU-Zoo-Ae.a-31 (KT339683) India**

**LA_BT_32F8 *Aedes aegypti*  BU-Zoo-Ae.a-30 (KT339682) India**

**LA_BT_32F9 *Aedes aegypti* Cambodia 3 (JQ926690) Cambodia**

**LA_BT_32F10 *Aedes aegypti*  BU-Zoo-Ae.a-31 (KT339683) India**

**LA_BT_32F11 *Aedes aegypti* Cambodia 3 (JQ926690) Cambodia**

**LA_BT_33M1 *Aedes aegypti*  BU-Zoo-Ae.a-31 (KT339683) India**

**LA_BT_33M2 *Aedes aegypti* Martinique 1 (JQ926696) Martinique (French Caribbean)**

**LA_BT_33M3 *Aedes aegypti* BU-Zoo-Ae.a-31 (KT339683) India**

**LA_BT_33M4 *Aedes aegypti* Haplotype 19 (KM203158) Colombia**

**LA_BT_33M5 *Aedes aegypti*  Haplotype 62 (KM203201) Colombia**

**LA_BT_33M6 *Aedes aegypti* Cambodia 3 (JQ926690) Cambodia**

**LA_BT_33M7 *Aedes aegypti* Cambodia 3 (JQ926690) Cambodia**

**LA_BT_33M8 *Aedes aegypti*  Haplotype 19 (KM203158) Colombia**

**LA_BT_33M9 *Aedes aegypti* Cambodia 3 (JQ926690) Cambodia**

**LA_BT_33M10 *Aedes aegypti* Cambodia 3 (JQ926690) Cambodia**

**LA_BT_33M11 *Aedes aegypti*  BU-Zoo-Ae.a-31 (KT339683) India**

**LA_BT_34M1 *Aedes aegypti* BU-Zoo-Ae.a-31 (KT339683) India**

**LA_BT_34M2 *Aedes aegypti* Cambodia 3 (JQ926690) Cambodia**

**LA_BT_34M3 *Aedes aegypti* Cambodia 3 (JQ926690) Cambodia**

**LA_BT_34M4 *Aedes aegypti* BU-Zoo-Ae.a-31 (KT339683) India**

**LA_BT_34M5 *Aedes aegypti*  Cambodia 3 (JQ926690) Cambodia**

**LA_BT_34M6 *Aedes aegypti* BU-Zoo-Ae.a-08 (KT339660) India**

**LA_BT_34M7 *Aedes aegypti* BU-Zoo-Ae.a-31 (KT339683) India**

**LA_BT_34M8 *Aedes aegypti*  Cambodia 3 (JQ926690) Cambodia**

**LA_BT_34M9 *Aedes aegypti* Cambodia 3 (JQ926690) Cambodia**

**LA_BT_34M10 *Aedes aegypti* Cambodia 3 (JQ926690) Cambodia**

**LA_BT_34M11 *Aedes aegypti*  BU-Zoo-Ae.a-31 (KT339683) India**

**LA_BT_34M12 *Aedes aegypti* BU-Zoo-Ae.a-31 (KT339683) India**

**LA_BT_34M13 *Aedes aegypti* Haplotype 62 (KM203201) Colombia**

**LA_BT_34M14 *Aedes aegypti* BU-Zoo-Ae.a-31 (KT339683) India**

**HT_PG_36F1 *Aedes albopictus* Unknown (HF536717) Romania**

**HT_PG_36F2 *Aedes albopictus* VAITC4665 (KU495082) Australia**

**HT_PG_36F3 *Aedes albopictus* Tez 7 (KT260201) India**

**HT_Hv_37F1 *Aedes albopictus* Tez5 (KT260200) India**

**HT_Hv_37F2 *Aedes albopictus* None (HF536717) Romania**

**TTH_L_38M1 *Aedes albopictus* VAITC4665 (KU495082) Australia**

**TTH_L_38M2 *Aedes vittatus* 20974_AevitE09 (KU380451) Kenya**

**TTH_L_38M3 *Aedes vittatus* 20974_AevitE09 (KU380451) Kenya**

**TTH_L_38M4 *Aedes albopictus* Tez 7 (KT260201) India**

**TTH_L_39F1 *Aedes cogilli* NIBGE MOS-01588 (KF406621) Pakistan**

**TTH_L_39F2 Unknown Unknown Unknown**

**TTH_L_39F3 *Aedes cogilli* NIBGE MOS-01588 (KF406621) Pakistan**

**TTH_L_39F4 *Aedes albopictus* Unknown (HF536717) Romania**

**TTH_L_39F5 *Aedes aegypti*  VAITC4689 (KU495081) Australia**

**TTH_L_39F6 *Aedes albopictus* Unknown (HF536717) Romania**

**TTH_P_44F3 *Aedes albopictus* Unknown (HF536717) Romania**

**TTH_P_44F4 *Aedes albopictus* Unknown (HF536717) Romania**

**TTH_P_44F5 *Aedes albopictus* Unknown (HF536717) Romania**

**TTH_P_44F6 *Aedes w-albus* NIBGE MOS-00138 (KF406651) Pakistan**

**TTH_KT_45M1 *Aedes albopictus* VAITC4665 (KU495082) Australia**

**TTH_KT_45M2 *Aedes albopictus* Tez5 (KT260200) India**

**TTH_KT_45M3 *Aedes albopictus* Tez5 (KT260200) India**

**TTH_KT_45M4 *Aedes albopictus* VAITC4665 (KU495082) Australia**

**TTH_KT_46F1 *Aedes albopictus* Tez5 (KT260200) India**

**TTH_KT_46F2 *Aedes albopictus* AB1_5S15_08P (KM613097) Thailand**

**TTH_KT_46F3 *Aedes aegypti* BU-Zoo-Ae.a-31 (KT339683) India**

**TTH_KT_46F4 *Aedes albopictus* Unknown (HF536717) Romania**

**TTH_KT_46F5 *Aedes albopictus* AB1_5S15_08P (KM613097) Thailand**

**TTH_KT_46F6 *Aedes aegypti*  Martinique 1 (JQ926696) Martinique (French Caribbean)**

**TTH_KT_46F7 *Aedes albopictus* Tez 7 (KT260201) India**

**TTH_KT_47F2 *Aedes aegypti*  BU-Zoo-Ae.a-08 (KT339660) India**

**TTH_KT_47F3 *Aedes aegypti* BU-Zoo-Ae.a-08 (KT339660) India**

**TTH_L_49F1 *Aedes albopictus* Unknown (HF536717) Romania**

**TTH_L_49F2 *Aedes albopictus* Unknown (HF536717) Romania**

**TTH_L_49F3 *Aedes albopictus* Unknown (HF536717) Romania**

**TTH_L_49F4 *Aedes albopictus* Tez 7 (KT260201) India**

**TTH_L_49F5 *Aedes albopictus* Tez 7 (KT260201) India**

**TTH_L_49F6 *Aedes albopictus* Tez5 (KT260200) India**

**TTH_L_49F7 Unknown Unknown Unknown**

**TTH_L_49F8 *Aedes albopictus* Unknown (HF536717) Romania**

**TTH_L_49F9 *Aedes albopictus* Tez5 (KT260200) India**

**TTH_L_49F10 *Aedes albopictus* VAITC4665 (KU495082) Australia**

**TTH_L_49F11 *Aedes albopictus* Tez 7 (KT260201) India**

**TTH_L_49F12 *Aedes albopictus* Unknown (HF536717) Romania**

**TTH_L_49F13 *Aedes albopictus* VAITC4665 (KU495082) Australia**

**TTH_L_50M1 *Aedes albopictus* AB1_5S15_08P (KM613097) Thailand**

**TTH_L_50M2 *Aedes albopictus* Unknown (HF536717) Romania**

**TTH_L_50M3 *Aedes albopictus* Tez5 (KT260200) India**

**TTH_L_50M4 *Aedes albopictus* Tez5 (KT260200) India**

**TTH_L_50M5 *Aedes albopictus* Tez5 (KT260200) India**

**TTH_L_50M6 *Aedes albopictus* Tez5 (KT260200) India**

**TTH_L_50M7 *Aedes albopictus* Tez5 (KT260200) India**

**TTH_L_50M8 *Aedes albopictus* Unknown (HF536717) Romania**

**TTH_L_50M9 *Aedes albopictus* Unknown (HF536717) Romania**

**TTH_L_50M11 *Aedes albopictus* Unknown (HF536717) Romania**

**TTH_L_50M12 *Aedes albopictus* Tez5 (KT260200) India**

**TTH_L_50M13 *Aedes albopictus* AB1_5S15_08P (KM613097) Thailand**

**TTH_L_50M14 *Aedes albopictus* VAITC4665 (KU495082) Australia**

**QT_LB_53M1 *Aedes aegypti*  BU-Zoo-Ae.a-31 (KT339683) India**

**QT_LB_53M2 *Aedes aegypti* BU-Zoo-Ae.a-31 (KT339683) India**

**QT_LB_53M3 *Aedes aegypti*  Cambodia 3 (JQ926690) Cambodia**

**QT_LB_53M4 *Aedes aegypti* BU-Zoo-Ae.a-31 (KT339683) India**

**QT_LB_53M5 *Aedes aegypti*  BU-Zoo-Ae.a-31 (KT339683) India**

**QT_LB_54F1 *Aedes aegypti* BU-Zoo-Ae.a-31 (KT339683) India**

**QT_LB_54F2 *Aedes aegypti*  Haplotype 62 (KM203201) Colombia**

**QT_LB_54F3 *Aedes aegypti* BU-Zoo-Ae.a-31 (KT339683) India**

**QT_LB_54F4 *Aedes aegypti*  BU-Zoo-Ae.a-31 (KT339683) India**

**QT_LB_54F5 *Aedes aegypti* BU-Zoo-Ae.a-31 (KT339683) India**

**QT_LB_54F6 *Aedes aegypti*  BU-Zoo-Ae.a-31 (KT339683) India**

**QT_LB_54F7 *Aedes aegypti* BU-Zoo-Ae.a-31 (KT339683) India**

**QT_LB_54F8 *Aedes aegypti*  BU-Zoo-Ae.a-31 (KT339683) India**

**QT_LB_55M1 *Aedes albopictus* alb9 (KP843400) Thailand**

**QT_LB_56F1 *Aedes albopictus* Unknown (HF536717) Romania**

**QT_TL_59F1 *Aedes aegypti*  NIBGE DIP-00264 (KF406352) Pakistan**

**QT_TL_59F2 *Aedes aegypti* BU-Zoo-Ae.a-08 (KT339660) India**

**QT_TL_59F3 *Aedes aegypti* NIBGE DIP-00264 (KF406352) Pakistan**

**QT_TL_59F4 *Aedes aegypti*  Martinique 1 (JQ926696) Martinique (French Caribbean)**

**QT_TT_61F1 *Aedes aegypti*  BU-Zoo-Ae.a-08 (KT339660) India**

**QT_TT_61F2 *Aedes aegypti* Martinique 1 (JQ926696) Martinique (French Caribbean)**

**QT_TT_61F3 *Aedes aegypti* Martinique 1 (JQ926696) Martinique (French Caribbean)**

**QT_TT_61F4 *Aedes aegypti*  Martinique 1 (JQ926696) Martinique (French Caribbean)**

**QT_TT_62M1 *Aedes aegypti* BU-Zoo-Ae.a-08 (KT339660) India**

**QT_TT_62M2 *Aedes aegypti*  BEN359_1 (KX446415) Benin**

**QT_TT_62M3 *Aedes aegypti* BU-Zoo-Ae.a-08 (KT339660) India**

**QT_TT_63F1 *Aedes mcintoshi* SEN2 (KJ940756) Senegal**

**QT_TT_64F1 *Aedes albopictus* Unknown (HF536717) Romania**

**QT_TT_64F2 *Culex bitaeniorhynchus* isolate 39-1 (KT358430) South Korea**

**HT_GP_67M1 *Aedes aegypti*  BU-Zoo-Ae.a-31 (KT339683) India**

**HT_GP_67M2 *Aedes aegypti* BU-Zoo-Ae.a-30 (KT339682) India**

**HT_GP_67M3 *Aedes aegypti*  BU-Zoo-Ae.a-31 (KT339683) India**

**HT_GP_67M4 *Aedes aegypti* BU-Zoo-Ae.a-31 (KT339683) India**

**HT_GP_67M5 *Aedes aegypti*  BU-Zoo-Ae.a-31 (KT339683) India**

**HT_GP_67M6 *Aedes aegypti* BU-Zoo-Ae.a-31 (KT339683) India**

**HT_GP_67M7 *Aedes aegypti*  BU-Zoo-Ae.a-31 (KT339683) India**

**HT_GP_67M8 *Aedes aegypti* BU-Zoo-Ae.a-31 (KT339683) India**

**HT_GP_67M9 *Aedes aegypti*  BU-Zoo-Ae.a-30 (KT339682) India**

**HT_GP_68F1 *Aedes aegypti* BU-Zoo-Ae.a-31 (KT339683) India**

**HT_GP_68F2 *Aedes aegypti*  BU-Zoo-Ae.a-31 (KT339683) India**

**HT_GP_68F3 *Aedes aegypti* BU-Zoo-Ae.a-31 (KT339683) India**

**HT_HK_70F1 *Aedes aegypti*  BU-Zoo-Ae.a-31 (KT339683) India**

**HT_HK_70F2 *Aedes aegypti* BU-Zoo-Ae.a-31 (KT339683) India**

**HT_HK_70F3 *Aedes aegypti*  Martinique 1 (JQ926696) Martinique (French Caribbean)**

**HT_HK_70F4 *Aedes aegypti* BU-Zoo-Ae.a-31 (KT339683) India**

**HT_HK_70F5 *Aedes aegypti*  BU-Zoo-Ae.a-30 (KT339682) India**

**HT_HK_70F6 *Aedes aegypti* BU-Zoo-Ae.a-30 (KT339682) India**

**HT_HK_70F7 *Aedes aegypti*  VAITC4689 (KU495081) Australia**

**HT_HK_70F8 *Aedes aegypti* Haplotype 62 (KM203201) Colombia**

**HT_HK_70F9 *Aedes aegypti*  BU-Zoo-Ae.a-30 (KT339682) India**

**HT_HK_70F10 *Aedes aegypti* BU-Zoo-Ae.a-30 (KT339682) India**

**HT_HK_70F11 *Aedes albopictus* Tez 7 (KT260201) India**

**HT_HK_70F12 *Aedes aegypti*  Haplotype 62 (KM203201) Colombia**

**HT_HK_70F13 *Aedes aegypti* BU-Zoo-Ae.a-08 (KT339660) India**

**HT_HK_70F14 *Aedes aegypti* BU-Zoo-Ae.a-30 (KT339682) India**

**HT_HK_70F15 *Aedes aegypti*  BU-Zoo-Ae.a-30 (KT339682) India**

**HT_HK_71M1 *Aedes aegypti* BEN359_1 (KX446415) Benin**

**HT_HK_71M2 *Aedes aegypti*  BU-Zoo-Ae.a-31 (KT339683) India**

**HT_HK_71M3 *Aedes aegypti* Haplotype 62 (KM203201) Colombia**

**HT_HK_71M4 *Aedes aegypti*  BU-Zoo-Ae.a-31 (KT339683) India**

**HT_HK_71M5 *Aedes aegypti* BU-Zoo-Ae.a-30 (KT339682) India**

**HT_HK_71M6 *Aedes aegypti*  BU-Zoo-Ae.a-30 (KT339682) India**

**HT_HK_71M7 *Aedes aegypti* BU-Zoo-Ae.a-30 (KT339682) India**

**HT_HK_71M8 *Aedes aegypti*  BU-Zoo-Ae.a-31 (KT339683) India**

**HT_HK_71M9 *Aedes aegypti* BU-Zoo-Ae.a-31 (KT339683) India**

**HT_HK_71M10 *Aedes aegypti*  BU-Zoo-Ae.a-31 (KT339683) India**

**HT_HK_71M11 *Aedes aegypti* BU-Zoo-Ae.a-31 (KT339683) India**

**HT_HK_71M12 *Aedes aegypti*  BU-Zoo-Ae.a-30 (KT339682) India**

**HT_HK_71M13 *Aedes aegypti* BU-Zoo-Ae.a-31 (KT339683) India**

**HT_HK_72F1 *Aedes albopictus* Tez 7 (KT260201) India**

**HT_HK_72F2 *Aedes albopictus* VAITC4665 (KU495082) Australia**

**HT_HK_72F3 *Aedes albopictus* Unknown (HF536717) Romania**

**HT_HK_72F4 *Aedes albopictus* Unknown (HF536717) Romania**

**HT_HK_72F5 *Aedes albopictus* Tez 7 (KT260201) India**

**HT_HK_72F6 *Aedes aegypti* BU-Zoo-Ae.a-31 (KT339683) India**

**HT_HK_72F7 *Aedes albopictus* Unknown (HF536717) Romania**

**HT_HK_73M1 *Aedes albopictus* Tez5 (KT260200) India**

**HT_HK_73M2 *Aedes albopictus* Tez5 (KT260200) India**

**HT_HK_73M4 *Aedes albopictus* VAITC4665 (KU495082) Australia**

**HT_HK_73M5 *Aedes albopictus* VAITC4665 (KU495082) Australia**

a) Accession number of the haplotype sequences already described
